# Trends in the Burden of Headache Disorders in Europe, 1990–2021: A Systematic Analysis from the Global Burden of Disease Study 2021

**DOI:** 10.3390/jcm14196966

**Published:** 2025-10-01

**Authors:** Terry Jung, Yoonkyung Chang, Moon-Kyung Shin, Sohee Wang, Seyedehmahla Hosseini, Joonho Kim, Min Kyung Chu, Tae-Jin Song

**Affiliations:** 1 Department of Medicine, Ewha Womans University College of Medicine, Seoul 07804, Republic of Korea; terry428@naver.com (T.J.); smk0712@ewha.ac.kr (M.-K.S.); omg24202@ewha.ac.kr (S.W.); mahlalee94@ewha.ac.kr (S.H.); 2 Department of Neurology, Mokdong Hospital, Ewha Womans University College of Medicine, Seoul 07985, Republic of Korea; ykchang@ewha.ac.kr; 3 Department of Neurology, Severance Hospital, Yonsei University College of Medicine, Seoul 03722, Republic of Korea; joonho345@yuhs.ac; 4 Department of Neurology, Seoul Hospital, Ewha Womans University College of Medicine, Seoul 07804, Republic of Korea; 5 Graduate Program in System Health Science and Engineering, Ewha Womans University, Seoul 03763, Republic of Korea

**Keywords:** headache disorders, migraine, tension type headache, DALY, Europe

## Abstract

**Background/Objectives:** Headache disorders, including migraine and tension-type headache (TTH), are among the most prevalent and disabling neurological conditions globally. This study aimed to evaluate temporal changes, demographic disparities, and socio-geographic variation in the burden of headache disorders across European countries. **Methods:** We analyzed data from the Global Burden of Disease Study 2021, covering 45 European countries grouped into Western, Central, and Eastern regions. We examined age-standardized prevalence, incidence, and disability-adjusted life year (DALY) rates for headache disorders between 1990 and 2021. Analyses were stratified by sex, age group, region, and country-level socio-demographic index (SDI). All estimates were reported with 95 percent uncertainty intervals where relevant. Spearman correlation was used to assess associations between disease burden and SDI. **Results:** Between 1990 and 2021, the number of individuals with headache disorders in Europe rose from 345.0 to 370.6 million, although age-standardized prevalence remained stable. The burden of migraine slightly increased, with age-standardized DALY rates rising from 648.35 to 657.27 per 100,000 population. Conversely, TTH showed a minor decline in both prevalence and DALY rates. Women and individuals aged 30 to 44 years consistently exhibited the highest burden, particularly for migraine. Higher SDI scores were positively correlated with DALY rates for migraine (rho = 0.392, *p* = 0.008) but negatively correlated for TTH (rho = −0.466, *p* = 0.001). **Conclusions:** Headache disorders continue to pose a major and largely unmitigated health burden across Europe. Regionally targeted strategies are essential to reduce disability and improve outcomes across diverse European populations.

## 1. Introduction

Headache disorders, particularly migraine and tension-type headache (TTH), are among the most widespread neurological conditions worldwide. They contribute substantially to long-term illness and to functional impairment in daily life [1,2]. These disorders often coexist with other medical conditions and, in doing so, amplify clinical severity and social burden [3]. Migraine alone affects more than one billion people globally and remains a leading cause of disability in women during their reproductive and working years [1,2,4]. Likewise, TTH remains among the most frequently encountered headache types on a global scale [1,2,5]. Taken together, the extensive prevalence and impact of migraine and TTH underscore their significance as major public health concerns that warrant close attention [6,7,8].

The Global Burden of Disease (GBD) initiative has provided comprehensive assessments of the worldwide and country-level effects of headache disorders [1]. According to recent global evaluations, headache disorders were the second most prevalent condition worldwide in 2021, while migraine alone ranked as the fourth leading cause of years lived with disability [9]. Importantly, the impact of headache disorders varies considerably depending on geographic location and socio-demographic context, highlighting the importance of conducting analyses tailored to specific regions and populations [1,2].

In Europe, headache disorders remain leading contributors to disability [10,11]. A recent migraine epidemiology study across five European countries (France, Germany, United Kingdom, Italy, and Spain) found that 11.5% of the total population had migraine. Among these patients, 56% reported disability due to headaches, and medication overuse was observed [12]. In a cross-sectional internet-based survey of migraine conducted in Germany and Spain, 78.5% of all patients received professional medical care. However, only 50.8% had been diagnosed with migraine. Although 73.9% of patients met the criteria for preventive medication, only 17.7% had ever used such medication. Among those who had used preventive medication, 66.8% reported satisfaction with it [13]. The headache burden among pediatric and adolescent patients is also significant and shows an increasing trend [14,15,16]. Although migraine and tension-type headache are primarily diagnosed in adults, it is not uncommon for children to visit the emergency room with primary headaches. In the 2019 GBD data, migraine prevalence in Western Europe was higher than the global mean, while it was lower than the mean in Eastern Europe. For TTH, prevalence in Eastern Europe and Western Europe was higher than the global average. Migraine-associated DALYs per 100,000 population ranked highest overall in Western Europe, while TTH-associated DALYs per 100,000 population ranked highest overall in Eastern Europe [17]. While the disease burden of headache disorders is high in Europe, research on regional differences in the diseases and their association with SDI, along with individualized health policy strategies, is needed.

Accordingly, this study utilized comprehensive data from the GBD 2021 framework to analyze patterns in the health burden posed by headache disorders across Europe between 1990 and 2021. We further explored disparities across demographic factors such as sex and age, as well as across different countries and socio-demographic index (SDI) levels.

## 2. Materials and Methods

The GBD 2021 is a comprehensive and methodical analysis of global diseases. By utilizing the estimates from this study, the present global, regional, and national disease burdens can be compared and assessed [18]. The research adheres to the Guidelines for Accurate and Transparent Health Estimates Reporting (GATHER) [19]. The research, conducted by the Institute for Health Metrics and Evaluation (IHME) at the University of Washington, utilizes anonymized data, which eliminates the requirement for informed consent. The data of CVDs analyzed in this study were sourced from the GBD 2021, which offered the most recent epidemiological estimates for 371 diseases and injuries across 21 GBD regions and 204 countries and territories from 1990 to 2021. All data were freely available through the Global Health Data Exchange (https://ghdx.healthdata.org (accessed on 13 March 2025), [20] with comprehensive information on the data, methodologies, and statistical modeling available in previous reports [9,21]. The relevant data were anonymous and publicly accessible. European countries were classified into Central (Bulgaria, North Macedonia, Montenegro, Romania, Serbia, Bosnia and Herzegovina, Albania, Hungary, Slovakia, Croatia, Czechia, Slovenia, Poland), Eastern (Belarus, Estonia, Latvia, Lithuania, Republic of Moldova, Russian Federation, Ukraine), and Western Europe (Andorra, Austria, Belgium, Cyprus, Denmark, Finland, France, Germany, Greece, Iceland, Ireland, Israel, Italy, Luxembourg, Malta, Monaco, The Netherlands, Norway, Portugal, San Marino, Spain, Sweden, Switzerland, UK) for analysis based on the classification of GBD for European countries. Institutional Review Board approval with a waiver of informed consent was obtained (EUMC 2023-10-040), as the study used de-identified secondary data.

The IHME at the University of Washington conducted searches in various databases including Embase, PubMed, System for Information on Gray Literature in Europe, CAP abstracts, Medline, CINAHL, and the World Health Organization Library Information System to gather GBD data, regardless of age, language, or sex. The IHME applied stringent inclusion criteria, excluding sources such as studies with fewer than 150 participants, review articles, investigations not based on general population samples, and those lacking a clearly defined target population [22,23]. Eligible datasets were subsequently integrated into the Global Health Data Exchange platform and analyzed using DisMod-MR 2.1 (World Health Organization), a Bayesian meta-regression modeling tool designed for disease burden estimation [24]. For the GBD 2021 study, systematic reviews of migraine, TTH, and medication overuse headache (MOH) were updated building on the previous reviews conducted for GBD 2017. Literature research was conducted through September 2017 using condition-specific search strings, and inclusion criteria required population-representative surveys reporting prevalence. Medical claims data were excluded due to instability in adjustment methods. For disease modeling, standard DisMod-MR settings were applied, assuming no incidence or prevalence before age 5 and zero excess mortality. Separate models were run for definite, probable, and total categories of migraine and TTH, with remission bounds set at 0.1 and 0.5, respectively, and results subsequently scaled to total headache envelopes. Adjustments were made for earlier data reporting only definite diagnoses, and sex- or age-specific regression models were used to harmonize definite and total categories. To refine time symptomatic estimates, unit-record data from the Lift the Burden multi-country survey were incorporated and MR-BRT methods were applied, yielding proportions of 0.093 (definite migraine), 0.066 (probable migraine), 0.029 (definite TTH), and 0.021 (probable TTH). A comprehensive description of the search strategy and inclusion criteria can be found in the Supplementary Methods. This study received Institutional Review Board (IRB) approval with a waiver of informed consent due to the use of secondary, de-identified data (EUMC 2023-10-040).

A migraine is definitively diagnosed if a patient’s symptoms meet all five major diagnostic criteria set by the International Classification of Headache Disorders, 3rd edition (ICHD-3) [25]. The diagnostic process for TTH follows the same procedure, with a definitive diagnosis made if a patient’s symptoms meet all five major diagnostic criteria outlined by the ICHD-3. Regarding diagnostic codes for headache disorders based on International Classification of Diseases, 9th version (ICD-9) and 10th version (ICD-10), the codes 346.93 (ICD-9) and G43-G43.919 (ICD-10) were considered as migraine and 307.81 (ICD-19) and G44.2-G44.229, and G44.4-G44.41 (ICD-10) for TTH [9]. In GBD 2021, migraine included definite and probable migraine and TTH also included definite and probable TTH. Moreover, MOH was regarded as a sequela of either migraine or TTH. Consequently, the burden of MOH was incorporated into the overall burdens estimated for migraine or TTH [9].)

The Socio-demographic Index (SDI), developed by the IHME in 2015, is a comprehensive measure used to evaluate the development level of countries or regions, highlighting the link between social development and population health outcomes. It is calculated as the geometric mean of three indicators, each normalized to a scale of 0 to 1: the total fertility rate for individuals younger than 25 years, the mean education level for individuals aged 15 years and older, and the lag-distributed income per capita. For GBD 2021, SDI values were scaled from 0 to 1, where 0 signifies the lowest education and income levels and the highest fertility rate, while 1 represents the highest education and income levels and the lowest fertility rate (https://ghdx.healthdata.org/record/global-burden-disease-study-2021-gbd-2021-socio-demographic-index-sdi-1950%E2%80%932021 (accessed on 13 March 2025)). The 204 countries and territories were categorized into five SDI regions based on quintiles: low (<0.46), low-middle (0.46–0.60), middle (0.61–0.69), high-middle (0.70–0.81), and high (>0.81) [9,21].

DALYs are a standard measure for assessing disease burden, representing the total healthy years lost from the onset of a disease to death. This metric combines both years of life lost (YLLs) due to premature mortality and YLDs. Because the GBD estimates do not directly attribute deaths to migraines, YLLs are set to 0 in this study. Consequently, DALYs are equivalent to YLDs in our study.

All estimates were presented with 95% uncertainty intervals (UIs), derived by repeatedly sampling the data 500 times. The upper and lower bounds of these intervals were based on the 2.5th and 97.5th percentiles of the resulting uncertainty distribution [9,21]. In this study, we examined the impact of migraines and TTH on health outcomes. To evaluate the extent of this impact, we utilized various metrics, including incidence, prevalence, and YLDs, along with their respective rates. The disease burden was estimated and presented with 95% uncertainty intervals (UI) to ensure accuracy and reliability [9,21]. Spearman’s correlation was conducted to assess the relationship between SDI and the burden of headache disorders, using R (version 4.4.2).

## 3. Results

### 3.1. Change in the Rank of Headache Disorders from 1990 to 2021

According to the GBD 2021 study, the age-standardized prevalence rate of headache disorders ranked 2nd among level 3 causes in Europe in both 1990 and 2021. Similarly, the prevalence of TTH consistently ranked second among level 4 causes over the same period. In contrast, the prevalence ranking of migraine increased from 4th in 1990 to 3rd in 2021. Further details on the rank changes are provided in the Supplementary Results and Supplementary Figure S1.

### 3.2. Change in the Burden of Headache Disorders from 1990 to 2021

The number of prevalent headache disorder cases in Europe changed from 345.0 million (95% UI: 318.2–371.5) in 1990 to 370.6 million (95% UI: 340.9–397.4) in 2021. The age-standardized prevalence rate per 100,000 was 40,799.81 (95% UI: 37,726.20 to 44,018.84) in 1990 and 40,685.82 (95% UI: 37,620.80 to 43,852.02) in 2021. The incidence rate of headache disorders was 12,143.81 (95% UI: 10,689.35 to 13,495.53) in 1990 and 12,070.27 (95% UI: 10,635.74 to 13,416.36) in 2021. The DALY rate of headache disorders also showed a 0.00% (95% UI: −0.01 to 0.00) percentage change, slightly increasing from 693.03 (95% UI: 177.31 to 1449.58) in 1990 to 696.22 (95% UI: 173.05 to 1449.93) in 2021. Globally, the age-standardized prevalence rates of headache disorders remained relatively stable (Supplementary Table S1).

The burden of migraine minimally increased both globally and in Europe. The percentage change in incidence rate showed the same at 0.01% (95% UI: 0.01 to 0.02) globally and in Europe. In Europe, the prevalence rate of migraine was 16,783.83 (95% UI: 14,495.57 to 19,320.19) in 1990 and 16,916.64 (95% UI: 14,714.84 to 19,481.74) in 2021, representing an increase of 0.01% (95% UI: 0.00 to 0.02). Similarly, the DALY rate was 648.35 (95% UI: 133.14 to 1372.86) in 1990 and 657.27 (95% UI: 145.44 to 1381.22) in 2021. Globally, the percentage changes in the age-standardized prevalence and DALY rates were 0.02% (95% UI: 0.00 to 0.03), and 0.01% (95% UI: −0.04 to 0.03), respectively (Supplementary Table S1b).

The burden of TTH showed declining trends of percentage changes globally and in Europe. The prevalence and incidence rates of TTH in Europe showed the same percentage decreases of 0.01% (95% UI: −0.02 to 0.00). The DALY rate of TTH was 78.70 (95% UI: 23.92 to 257.25) in 1990 and 77.79 (95% UI: 23.49 to 256.54) in 2021. Similarly, the global prevalence rate of TTH slightly decreased by 0.01% (95% UI: −0.01 to 0.00), from 24,904.85 (95% UI: 21,960.05 to 28,038.80) in 1990 to 24,764.77 (95% UI: 21,863.62 to 27,954.74) in 2021. The DALY rate of TTH was 56.99 (95% UI: 16.79 to 186.13) in 1990 and 55.69 (95% UI: 16.13 to 185.07) in 2021. The incidence rate of TTH was 8960.34 (95% UI: 7815.06 to 10,074.35) in 1990 and 8931.31 (95% UI: 7788.21 to 10,020.83) in 2021 (Supplementary Table S1c). Additional information on burden estimates from 1990 to 2021 is provided in the Supplementary Results and Supplementary Tables S1 and S2, Supplementary Figures S2 and S3).

### 3.3. The Burden of Headache Disorders According to the European Countries

**Figure 1:**
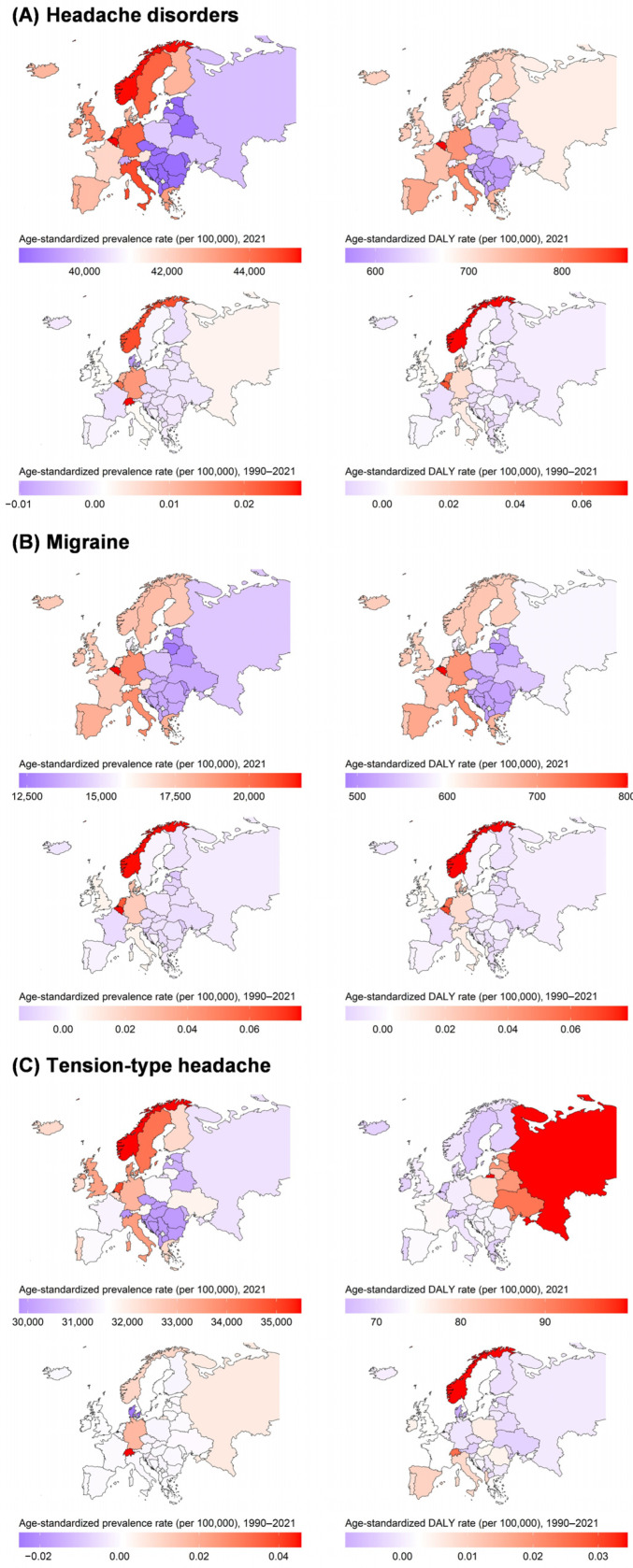
Age-standardized prevalence and DALY rates for (**A**) headache disorders, (**B**) migraine, and (**C**) tension-type headache in 2021, and percentage changes in age-standardized rate from 1990 to 2021.

Table 1 and Figure 1 show age-standardized prevalence, DALY, and incidence rates for headache disorders, migraine, and TTH in 2021 across European countries. Belgium (45,248.17, 95% UI: 41,636.71 to 48,749.14) had the highest prevalence rate per 100,000. In contrast, the country with the lowest prevalence rate was Estonia (38,419.23, 95% UI: 34,900.22 to 42,118.05). Similarly, Belgium was the country with the highest DALY rate (869.78, 95% UI: 135.03 to 1852.24). The country with the lowest DALY rate of headache disorders was Lithuania (568.25, 95% UI: 181.60 to 1159.12).

Belgium, Italy, and Germany ranked highest in prevalence and DALY rates for migraine. The prevalence rates of Belgium, Italy, and Germany were 21,751.47 (95% UI: 18,730.05 to 25,705.50), 19,244.27 (95% UI: 16,729.30 to 22,008.96), and 19,203.72 (95% UI: 16,546.88 to 22,548.90), respectively. Similarly, the DALY rates of Belgium, Italy, and Germany were 800.36 (95% UI: 91.99 to 1772.02), 717.20 (95% UI: 89.24 to 1568.08), and 707.40 (95% UI: 101.69 to 1567.80), respectively. Lithuania had the lowest prevalence, DALY, and incidence rates of migraine at 12,244.96 (95% UI: 10,458.50 to 14,260.23), 487.22 (95% UI: 121.55 to 1024.43), and 976.09 (95% UI: 830.75 to 1129.22), respectively (Table 1).

The country with the highest prevalence rate of TTH was Norway (35,492.44, 95% UI: 31,718.03 to 39,481.91), followed by the Netherlands (34,984.91, 95% UI: 30,123.30 to 39,942.65) and Sweden (34,297.27, 95% UI: 30,701.03 to 38,326.02). In contrast, the country with the lowest prevalence rate was Switzerland (29,829.20, 95% UI: 25,864.17 to 34,171.76). Switzerland also had the lowest DALY rate of TTH (66.36, 95% UI: 18.13 to 241.18). The country with the highest DALY rates was Russian Federation (99.72, 95% UI: 34.79 to 288.43). Norway, Sweden, and Italy ranked the highest in incidence rates for TTH. The incidence rates of Norway, Sweden, and Italy were 12,225.67 (95% UI: 10,673.08 to 13,778.10), 11,957.24 (95% UI: 10,503.70 to 13,393.96), and 11,907.30 (95% UI: 10,379.71 to 13,336.30), respectively (Table 1). Additional country-specific estimates and changes are detailed in the Supplementary Results.

### 3.4. The Burden of Headache Disorders by European Regions

**Figure 2:**
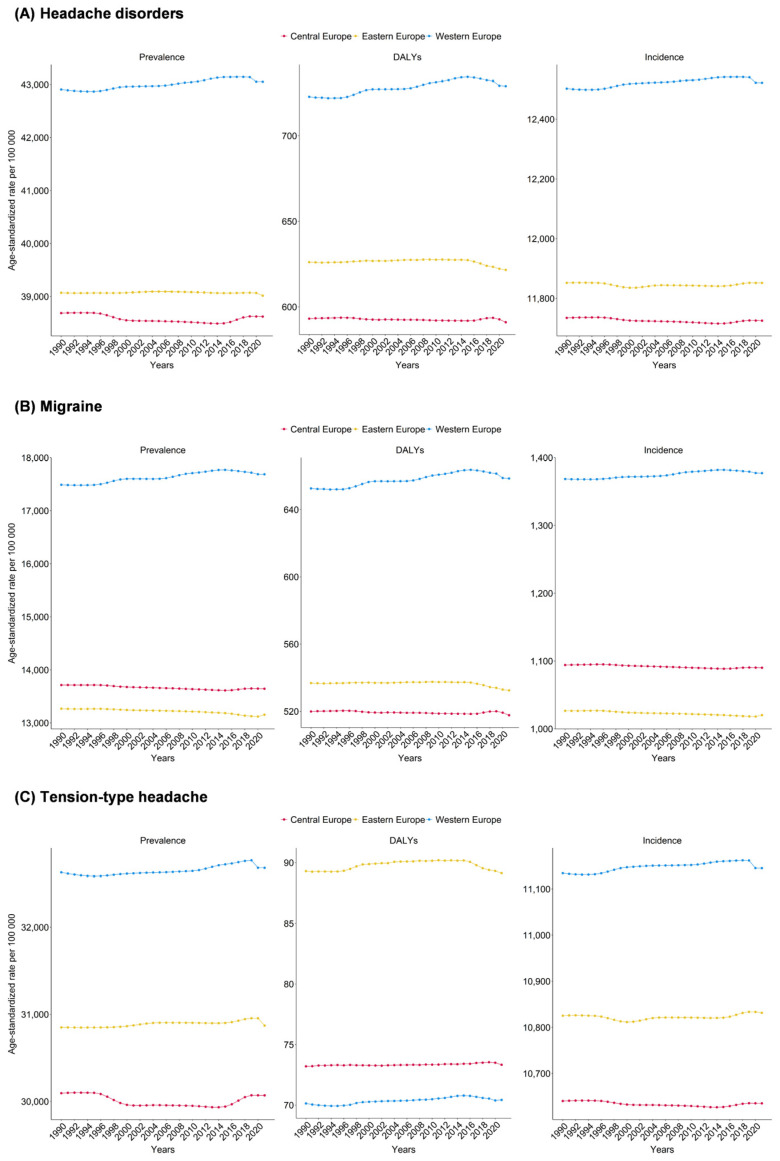
Age-standardized prevalence, DALYs and incidence rates for (**A**) headache disorders, (**B**) migraine, and (**C**) tension-type headache in Central, Eastern, and Western Europe, 1990–2021.

When dividing Europe into three regions—Central, Eastern, and Western Europe—Western Europe showed the highest age-standardized prevalence and incidence rates for headache disorders, migraine, and TTH. Central Europe had the lowest incidence rate of headache disorders, while Eastern Europe had the lowest migraine burden. Interestingly, Eastern Europe recorded the highest DALY rate for TTH. Across the three regions, only minor changes were observed in age-standardized prevalence, DALY, and incidence rates from 1990 to 2021 (Figure 2 and Figure S7). Additional region-specific data are presented in the Supplementary Results, Table S1, Figures S2 and S7.

### 3.5. Differences by Sex and Age

Sex- and age-based differences in the prevalence and burden of headache disorders, migraine, and TTH were apparent across European countries in GBD 2021, with consistently higher rates among females. Belgium had the highest age-standardized prevalence of headache disorders in females, while the Netherlands ranked highest among males. Belgium also had the highest DALY rates for headache disorders and migraine, whereas the Russian Federation had the highest DALY rate for TTH.

For both sexes, the peak age group for headache disorder prevalence was 30–34 years. Migraine prevalence peaked at 40–44 years, and TTH at 30–34 years. DALY rates peaked at 40–44 years for headache disorders and migraine, and at 45–49 years for TTH. Between 1990 and 2021, males and females exhibited similar trends in age-standardized prevalence, incidence, DALY, and YLD rates across all headache disorders, with only slight variations in absolute numbers. Age-specific distributions of prevalence, DALY, incidence, and YLD cases closely mirrored the age-standardized trends (Supplementary Tables S3–S5, Supplementary Figures S8–S11).

### 3.6. Association of Burden with the SDI

**Figure 3:**
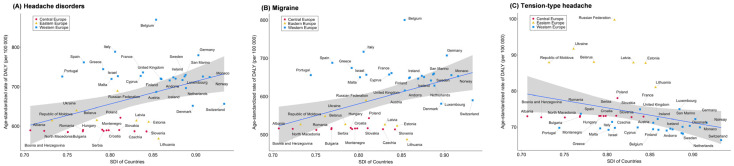
Age-standardized DALYs rates for (**A**) headache disorders, (**B**) migraine, and (**C**) tension-type headache in Central, Eastern, and Western Europe, by socio-demographic index (SDI).

Age-standardized DALY rates of headache disorders (rho = 0.389, *p* = 0.008) and migraine (rho = 0.392, *p* = 0.008) showed positive correlations with SDI. In contrast, the DALY rate of TTH (rho = −0.466, *p* = 0.001) showed a negative correlation with SDI (Supplementary Table S6). When categorized by countries, countries in Western Europe such as Germany, Monaco, and Norway had high SDI and high prevalence, DALY, incidence, and YLD rates of headache disorders and migraine. Conversely, countries in Central Europe such as Albania, Bosnia and Herzegovina, and North Macedonia showed low SDI and low rates of the two disorders. For TTH, Western European countries similarly had high SDI and high prevalence and incidence rates, whereas countries in Central Europe and Eastern Europe had relatively low SDI and low rates. However, countries in Western Europe such as Switzerland, Norway, and Monaco had high SDI but low DALY and YLD rates, representing negative correlations (Figure 3 and Figure S12a–c).

## 4. Discussion

Our key findings revealed that headache disorders, including migraine and TTH, remain a significant public health burden in Europe. From 1990 to 2021, the absolute number of people affected rose from approximately 345 million to 371 million, reflecting population growth and aging. However, the age-standardized prevalence and incidence rates remained essentially unchanged for over three decades. Notably, the age-standardized prevalence of migraine slightly increased, and its DALY rate also rose marginally, whereas TTH showed only small decreases in both metrics. These results align with global estimates indicating that headache disorders—especially migraine—continue to rank among the top contributors to disability worldwide [1,2]. Globally, migraine and TTH showed a high disease burden, accounting for over 98% of neurological disorders incidence in 2021 [26]. Despite their non-fatal nature, headache disorders have become relatively more prominent causes of disability in Europe, as reflected in migraine’s rise to the third highest DALY contributor among neurological disorders [27].

Importantly, our analysis revealed pronounced country-specific outliers. Belgium exhibited the highest prevalence and DALY rates for both headache disorders and migraine, far above the regional average—a finding supported by national analyses of the Belgian burden of disease [10]. In contrast, Lithuania reported the lowest DALY rates, which may partly reflect underdiagnosis or limited surveillance data rather than a truly low disease burden. For TTH, Norway stood out with the highest incidence rates, exceeding those of many Eastern European countries by more than twofold, possibly reflecting differences in diagnostic criteria or healthcare utilization. Recognizing such outliers is essential, as they highlight both best- and worst-case scenarios that could guide regional health strategies.

Regionally, Western Europe had the highest age-standardized prevalence and incidence rates of headache disorders and migraine, while Central Europe showed the lowest. Conversely, Eastern Europe recorded the highest DALY rates for TTH. Despite these differences in magnitude, all European regions exhibited relatively stable burden trajectories over time. Regional heterogeneity in headache burden is likely multifactorial. Differences in healthcare access—including availability of neurologists and primary care physicians familiar with ICHD criteria—may lead to higher reported burden in Western Europe compared with underdiagnosis in Central or Eastern Europe. Cultural attitudes toward reporting headache symptoms also vary; in some countries, headache may be normalized and under-reported, whereas in others, awareness campaigns and patient advocacy have heightened recognition. Furthermore, socioeconomic factors such as occupational stress, urbanization, and education levels may contribute to varying prevalence patterns. The timing and intensity of national headache awareness programs, as well as the adoption of ICHD diagnostic criteria, further shape these observed differences.

The highest burden of headache disorders is observed among young or middle individuals, reflecting significant implications for workforce productivity and quality of life. Particularly, this age group frequently experiences migraine, underscoring the economic and social impacts associated with peak productive ages [28,29]. Prevalence tends to decline in older age groups, suggesting potential age-related variations in headache manifestation or reporting behaviors [28,29]. Consistent with previous findings, the prevalence and burden of migraine and TTH remain higher among women than men. Previous studies suggested that approximately 21% of women reported migraine or severe headaches compared to 10.7% of men, highlighting the role of biological and hormonal factors in headache susceptibility [3,28,30,31,32]. Addressing these sex-specific differences is critical for targeted treatment and prevention programs.

We found positive correlations between SDI and DALY rates for migraine and combined headache disorders. We also found a negative correlation between SDI and TTH DALYs. In other words, more developed countries experienced higher recorded migraine disability but lower TTH-related disability. Although we are unable to provide clear evidence for the association, our findings likely reflect both increased healthcare access and awareness in high-SDI countries, leading to better migraine recognition, as well as more effective management of milder TTH symptoms [27,33,34]. At the same time, it is important to note that socioeconomic disadvantage within high-SDI countries remains a risk factor for severe headache outcomes. For example, individuals with lower income and education in high-income countries like Germany report higher headache-related disability and lower treatment rates [35,36,37]. This inverse correlation may help explain the negative correlations with SDI and DALY rate of TTH.

Our findings have important public health and policy implications. First, countries with a disproportionately high burden, such as Belgium and Norway, may benefit from intensified prevention strategies, including workplace interventions and stress management programs, given the concentration of burden in working-age adults. Second, countries with unexpectedly low reported burden, such as Lithuania, should strengthen surveillance systems and expand epidemiological surveys to avoid underestimation. Addressing sex-specific differences requires policies targeting women, who consistently report higher migraine prevalence and disability. Moreover, the high incidence of medication overuse headache highlights the need for careful prescribing practices, patient education, and regulation of over-the-counter analgesics. Finally, cross-national collaboration in Europe—for example, through shared guidelines, training initiatives, and awareness campaigns—may reduce disparities and ensure equitable access to effective headache care.

In the GBD 2021 study, limitations in data collection and analysis methods prevented the distinction between probable and definite diagnoses based on the ICHD criteria for migraine and TTH. Despite this, probable migraine carries a significant disease burden and disability similar to that of definite migraine [38,39]. The primary issue leading to a probable rather than definite diagnosis in past studies has often been the duration of the headache. Consequently, the GBD study’s approach of categorizing probable migraine within the broader category of migraine may be reasonable. This method may ensure a comprehensive understanding of the disease burden. A similar argument can be made for probable TTH, although the knowledge of this type of headache is significantly less developed. Additionally, the DALYs missed by excluding this headache type are fewer due to the considerably lower disability weight associated with TTH compared to migraine [8]. On the other hand, headache disorders are generally underdiagnosed, with a significant number of patients not seeking medical care. Furthermore, since headache intensity and burden are typically calculated based on patient reports, self-report variability can influence the results. While migraine and TTH diagnoses were classified according to the ICHD-3 diagnostic criteria, the fact that headache diagnostic criteria changed over the period is also a limitation that must be considered. Lastly, GBD estimates for headache disorders in European countries with limited primary data coverage. In several nations, especially those with scarce epidemiological surveys, the burden of headache was not derived from actual observed data but instead estimated through statistical modeling using the DisMod-MR framework. While this approach enables comprehensive regional comparisons, it inevitably introduces uncertainty, as modeled estimates may not fully reflect the true epidemiological patterns in settings where direct evidence is lacking. Therefore, the findings should be interpreted with caution, particularly for countries with lower data availability, where the accuracy of the reported burden may be constrained by the assumptions of the modeling process.

## 5. Conclusions

Headache disorders including migraine and TTH continue to impose considerable health burdens in Europe, with distinct disparities by sex, age, socio-demographic factors, and geography. Addressing these disparities requires targeted interventions, comprehensive public health strategies, and improved healthcare accessibility. Continued research and tailored interventions are critical to reducing the overall burden and improving quality of life for populations with headache disorders.

## Figures and Tables

**Table 1:** Prevalence, DALY, and incidence counts and age-standardized rates of headache disorders, migraine, and tension-type headache in 2021, and percentage changes in age-standardized rate from 1990 to 2021, by region.

	Prevalence (95% UI)			DALYs (95% UI)			Incidence (95% UI)		
	Absolute Number, 2021	Age-Standardized Rate, per 100,000 People, 2021	Percentage Change, 1990–2021	Absolute Number, 2021	Age-Standardized Rate, per 100,000 People, 2021	Percentage Change, 1990–2021	Absolute Number, 2021	Age-Standardized Rate, per 100,000 People, 2021	Percentage Change, 1990–2021
Headache Disorders									
Europe	370,591,630.52 (340,862,080.96 to 397,397,185.77)	40,685.82 (37,620.80 to 43,852.02)	0.00% (−0.01% to 0.00%)	6,345,063.09 (1,796,988.70 to 12,906,373.08)	696.22 (173.05 to 1449.93)	0.00% (−0.02% to 0.02%)	104,292,837.24 (92,399,738.42 to 115,349,658.98)	12,070.27 (10,635.74 to 13,416.36)	−0.01% (−0.01% to 0.00%)
Central Europe	48,986,031.61 (44,773,980.88 to 52,892,512.71)	39,073.11 (35,876.92 to 42,546.93)	0.00% (0.00% to 0.00%)	768,193.81 (238,261.14 to 1,575,941.56)	599.93 (163.52 to 1264.19)	0.00% (−0.01% to 0.02%)	14,086,702.42 (12,455,869.12 to 15,580,372.60)	11,943.96 (10,495.46 to 13,312.91)	0.00% (0.00% to 0.00%)
Eastern Europe	89,285,285.39 (82,617,768.80 to 95,996,350.07)	39,866.60 (36,979.81 to 43,073.94)	0.00% (−0.01% to 0.01%)	1,576,590.49 (612,730.26 to 3,028,642.64)	671.26 (241.79 to 1331.61)	0.00% (−0.01% to 0.01%)	25,940,760.78 (22,902,462.71 to 28,699,839.74)	12,263.83 (10,834.87 to 13,644.35)	0.00% (−0.01% to 0.02%)
Western Europe	201,163,898.69 (185,551,318.08 to 216,210,145.68)	43,409.83 (40,111.93 to 46,783.53)	0.00% (0.00% to 0.01%)	3,387,292.30 (764,627.48 to 6,980,774.17)	748.58 (144.83 to 1578.77)	0.01% (−0.01% to 0.02%)	55,477,379.37 (49,552,163.08 to 61,469,217.94)	12,661.01 (11,128.29 to 14,103.83)	0.00% (−0.01% to 0.01%)
Migraine									
Europe	143,542,170.14 (124,859,309.64 to 165,307,694.92)	16,128.60 (13,894.33 to 18,592.33)	0.01% (0.00% to 0.02%)	5,577,086.08 (1,234,081.57 to 11,719,998.01)	618.43 (116.07 to 1315.08)	0.01% (−0.02% to 0.02%)	9,057,610.76 (7,881,493.51 to 10,303,044.20)	1248.05 (1084.00 to 1419.21)	0.01% (0.01% to 0.02%)
Central Europe	17,302,363.75 (15,177,932.24 to 19,780,847.61)	13,822.04 (11,854.14 to 15,865.31)	0.00% (−0.01% to 0.00%)	666,284.65 (159,796.29 to 1,436,227.37)	525.24 (109.75 to 1158.95)	0.00% (−0.01% to 0.01%)	1,109,874.37 (965,027.11 to 1,271,786.91)	1101.88 (949.54 to 1260.69)	0.00% (0.00% to 0.00%)
Eastern Europe	32,050,565.68 (27,899,660.01 to 36,442,160.85)	14,077.09 (12,210.58 to 16,112.04)	0.00% (0.00% to 0.00%)	1,336,530.24 (424,522.73 to 2,695,926.70)	574.43 (164.13 to 1193.90)	0.00% (−0.01% to 0.01%)	2,009,050.44 (1,775,939.75 to 2,265,371.53)	1072.44 (944.27 to 1208.65)	0.00% (0.00% to 0.00%)
Western Europe	80,359,608.30 (70,005,541.78 to 93,109,621.14)	18,170.70 (15,724.92 to 21,091.55)	0.01% (−0.01% to 0.02%)	3,026,513.28 (527,630.22 to 6,473,769.21)	676.64 (96.68 to 1471.91)	0.01% (−0.01% to 0.03%)	4,913,868.33 (4,280,234.45 to 5,568,358.73)	1399.05 (1207.74 to 1596.87)	0.00% (−0.01% to 0.01%)
Tension-type headache									
Europe	282,494,328.80 (252,165,015.17 to 313,416,190.31)	30,810.85 (27,241.14 to 34,786.17)	−0.01% (−0.02% to 0.00%)	767,977.02 (245,879.07 to 2,405,182.64)	77.79 (23.49 to 256.54)	−0.01% (−0.06% to 0.02%)	95,235,226.48 (83,299,117.39 to 105,938,467.33)	10,822.21 (9430.78 to 12,181.47)	−0.01% (−0.02% to 0.00%)
Central Europe	38,175,566.57 (33,813,707.07 to 42,723,788.04)	30,488.91 (26,793.28 to 34,697.76)	0.00% (0.00% to 0.00%)	101,909.16 (32,757.21 to 310,711.10)	74.70 (22.49 to 248.04)	0.01% (−0.06% to 0.04%)	12,976,828.05 (11,395,486.60 to 14,469,564.91)	10,842.09 (9385.16 to 12,204.69)	0.00% (0.00% to 0.00%)
Eastern Europe	69,475,455.82 (62,206,601.56 to 76,924,171.47)	31,243.12 (27,766.56 to 34,948.02)	0.00% (−0.02% to 0.02%)	240,060.24 (86,665.76 to 660,402.84)	96.83 (33.12 to 286.15)	0.00% (−0.03% to 0.03%)	23,931,710.34 (20,936,072.75 to 26,643,791.37)	11,191.39 (9761.98 to 12,541.10)	0.00% (−0.02% to 0.02%)
Western Europe	153,919,251.89 (136,293,091.30 to 171,722,574.01)	32,770.54 (28,999.01 to 37,028.00)	0.00% (−0.01% to 0.01%)	360,779.02 (103,682.91 to 1,263,384.07)	71.93 (19.05 to 261.97)	0.00% (−0.06% to 0.04%)	50,563,511.05 (44,445,362.07 to 56,493,751.33)	11,261.96 (9744.87 to 12,737.18)	0.00% (−0.01% to 0.01%)

UI, Uncertainty interval; DALYs, disability-adjusted life-years.

## Data Availability

The GBD study is an open-source dataset that is freely accessible and available for use by anyone. The data in this study are available from the corresponding author upon reasonable request.

## Supplementary Materials

The following supporting information can be downloaded at: https://www.mdpi.com/article/10.3390/jcm14196966/s1, Figure S1a: Changes in top ranks of age-standardized (A) prevalence, (B) DALY, (C) incidence, and (D) YLD rates for cause level 3 conditions from 1990 to 2021; Figure S1b: Changes in top ranks of age-standardized (A) prevalence, (B) DALY, (C) incidence, and (D) YLD rates for cause level 4 conditions from 1990 to 2021; Figure S2a: Age-standardized (A) prevalence, (B) DALY, (C) incidence, and (D) YLD rates and counts of headache disorders by year, 1990–2021; Figure S2b: Age-standardized (A) prevalence, (B) DALY, (C) incidence, and (D) YLD rates and counts of migraine by year, 1990–2021; Figure S2c: Age-standardized (A) prevalence, (B) DALY, (C) incidence, and (D) YLD rates and counts of tension-type headache by year, 1990–2021; Figure S3a: Age-standardized (A) prevalence and DALY, and (B) incidence and YLD rates and counts of headache disorders by year, 1990–2021; Figure S3b: Age-standardized (A) prevalence and DALY, and (B) incidence and YLD rates and counts of migraine by year, 1990–2021; Figure S3c: Age-standardized (A) prevalence and DALY, and (B) incidence and YLD rates and counts of tension-type headache by year, 1990–2021; Figure S4a: Region-wise percentage changes in (A) prevalence, (B) DALY, (C) incidence, and (D) YLD in headache disorders burden, 1990–2021; Figure S4b: Region-wise percentage changes in (A) prevalence, (B) DALY, (C) incidence, and (D) YLD in migraine burden, 1990–2021; Figure S4c: Region-wise percentage changes in (A) prevalence, (B) DALY, (C) incidence, and (D) YLD in tension-type headache burden, 1990–2021; Figure S5a: Changes in age-standardized (A) prevalence, (B) DALY, (C) incidence, and (D) YLD rate for headache disorders, by country, 2000–2010 and 2010–2021; Figure S5b: Changes in age-standardized (A) prevalence, (B) DALY, (C) incidence, and (D) YLD rate for migraine, by country, 2000–2010 and 2010–2021; Figure S5c: Changes in age-standardized (A) prevalence, (B) DALY, (C) incidence, and (D) YLD rate for tension-type headache, by country, 2000–2010 and 2010–2021; Figure S6a: Changes in age-standardized (A) prevalence, (B) DALY, (C) incidence, and (D) YLD rate for headache disorders, by country, 2010–2019 and 2019–2021; Figure S6b: Changes in age-standardized (A) prevalence, (B) DALY, (C) incidence, and (D) YLD rate for migraine, by country, 2010–2019 and 2019–2021; Figure S6c: Changes in age-standardized (A) prevalence, (B) DALY, (C) incidence, and (D) YLD rate for tension-type headache, by country, 2010–2019 and 2019–2021; Figure S7a: Annual changes in (A) prevalence, (B) DALY, (C) incidence, and (D) YLD rates of headache disorders comparing pre-pandemic (2010–2019) to pandemic periods (2019–2021), stratified by age group and by region; Figure S7b: Annual changes in (A) prevalence, (B) DALY, (C) incidence, and (D) YLD rates of migraine comparing pre-pandemic (2010–2019) to pandemic periods (2019–2021), stratified by age group and by region; Figure S7c: Annual changes in (A) prevalence, (B) DALY, (C) incidence, and (D) YLD rates of tension-type headache comparing pre-pandemic (2010–2019) to pandemic periods (2019–2021), stratified by age group and by region; Figure S8a: Total (A) prevalence, (B) DALY, (C) incidence, and (D) YLD rates of headache disorders for both sexes, by age group and region, 2021; Figure S8b: Total (A) prevalence, (B) DALY, (C) incidence, and (D) YLD rates of migraine for both sexes, by age group and region, 2021; Figure S8c: Total (A) prevalence, (B) DALY, (C) incidence, and (D) YLD rates of tension-type headache for both sexes, by age group and region, 2021; Figure S9a: Age-standardized rates of (A) prevalence, (B) DALY, (C) incidence, and (D) YLD of headache disorders, by country and sex, 1990–2021; Figure S9b: Age-standardized rates of (A) prevalence, (B) DALY, (C) incidence, and (D) YLD of migraine, by country and sex, 1990–2021; Figure S9c: Age-standardized rates of (A) prevalence, (B) DALY, (C) incidence, and (D) YLD of tension-type headache, by country and sex, 1990–2021; Figure S10a: Age-standardized prevalence, DALY, and incidence rates and counts of headache disorders, by sex, 1990–2021; Figure S10b: Age-standardized prevalence, DALY, and incidence rates and counts of migraine, by sex, 1990–2021; Figure S10c: Age-standardized prevalence, DALY, and incidence rates and counts of tension-type headache, by sex, 1990–2021; Figure S11a: Counts and rates of (A) prevalence and DALY, and (B) incidence and YLD of headache disorders, by year, age group, and sex; Figure S11b: Counts and rates of (A) prevalence and DALY, and (B) incidence and YLD of migraine, by year, age group, and sex; Figure S11c: Counts and rates of (A) prevalence and DALY, and (B) incidence and YLD of tension-type headache, by year, age group, and sex; Figure S12a: Age-standardized (A) prevalence, (B) DALY, (C) incidence, and (D) YLD rates for headache disorders in Europe, by socio-demographic index (SDI), 2021; Figure S12b: Age-standardized (A) prevalence, (B) DALY, (C) incidence, and (D) YLD rates for migraine in Europe, by socio-demographic index (SDI), 2021; Figure S12c: Age-standardized (A) prevalence, (B) DALY, (C) incidence, and (D) YLD rates for tension-type headache in Europe, by socio-demographic index (SDI), 2021; Table S1a: Prevalence, DALY, incidence, and YLD counts and age-standardized rates of headache disorders in 1990 and 2021, and percentage changes of age-standardized rate from 1990 to 2021, in global and Europe; Table S1b: Prevalence, DALY, incidence, and YLD counts and age-standardized rates of migraine in 1990 and 2021, and percentage changes of age-standardized rate from 1990 to 2021, in global and Europe; Table S1c: Prevalence, DALY, incidence, and YLD counts and age-standardized rates of tension-type headache in 1990 and 2021, and percentage changes of age-standardized rate from 1990 to 2021, in global and Europe; Table S2a: Counts and age-standardized prevalence rates of headache disorders from 1990 to 2021, by sex; Table S2b: Counts and age-standardized DALY rates of headache disorders from 1990 to 2021, by sex; Table S2c: Counts and age-standardized incidence rates of headache disorders from 1990 to 2021, by sex; Table S2d: Counts and age-standardized YLD rates of headache disorders from 1990 to 2021, by sex; Table S2e: Counts and age-standardized prevalence rates of migraine from 1990 to 2021, by sex; Table S2f: Counts and age-standardized DALY rates of migraine from 1990 to 2021, by sex; Table S2g: Counts and age-standardized incidence rates of migraine from 1990 to 2021, by sex; Table S2h: Counts and age-standardized YLD rates of migraine from 1990 to 2021, by sex; Table S2i: Counts and age-standardized prevalence rates of tension-type headache from 1990 to 2021, by sex; Table S2j: Counts and age-standardized DALY rates of tension-type headache from 1990 to 2021, by sex; Table S2k: Counts and age-standardized incidence rates of tension-type headache from 1990 to 2021, by sex; Table S2l: Counts and age-standardized YLD rates of tension-type headache from 1990 to 2021, by sex; Table S3a: Counts and age-standardized prevalence rates of headache disorders in 2021, and percentage changes from 1990 to 2021, by country and sex; Table S3b: Counts and age-standardized DALY rates of headache disorders in 2021, and percentage changes from 1990 to 2021, by country and sex; Table S3c: Counts and age-standardized incidence rates of headache disorders in 2021, and percentage changes from 1990 to 2021, by country and sex; Table S3d: Counts and age-standardized YLD rates of headache disorders in 2021, and percentage changes from 1990 to 2021, by country and sex; Table S3e: Counts and age-standardized prevalence rates of migraine in 2021, and percentage changes from 1990 to 2021, by country and sex; Table S3f: Counts and age-standardized DALY rates of migraine in 2021, and percentage changes from 1990 to 2021, by country and sex; Table S3g: Counts and age-standardized incidence rates of migraine in 2021, and percentage changes from 1990 to 2021, by country and sex; Table S3h: Counts and age-standardized YLD rates of migraine in 2021, and percentage changes from 1990 to 2021, by country and sex; Table S3i: Counts and age-standardized prevalence rates of tension-type headache in 2021, and percentage changes from 1990 to 2021, by country and sex; Table S3j: Counts and age-standardized DALY rates of tension-type headache in 2021, and percentage changes from 1990 to 2021, by country and sex; Table S3k: Counts and age-standardized incidence rates of tension-type headache in 2021, and percentage changes from 1990 to 2021, by country and sex; Table S3l: Counts and age-standardized YLD rates of tension-type headache in 2021, and percentage changes from 1990 to 2021, by country and sex; Table S4a: Prevalence, DALY, and incidence rates of headache disorders, by age and sex, 2021; Table S4b: Prevalence, DALY, and incidence rates of migraine, by age and sex, 2021; Table S4c: Prevalence, DALY, and incidence rates of tension-type headache, by age and sex, 2021; Table S5a: Prevalence, DALY, incidence, and YLD rates of headache disorders for both sexes, by age, 2021; Table S5b: Prevalence, DALY, incidence, and YLD rates of migraine for both sexes, by age, 2021; Table S5c: Prevalence, DALY, incidence, and YLD rates of tension-type headache for both sexes, by age, 2021; Table S6a: Socio-demographic index (SDI) with prevalence, DALY, and incidence counts and age-standardized rates of headache disorders in 2021, and percentage changes from 1990 to 2021, by country; Table S6b: Socio-demographic index (SDI) with prevalence, DALY, and incidence counts and age-standardized rates of migraine in 2021, and percentage changes from 1990 to 2021, by country; Table S6c: Socio-demographic index (SDI) with prevalence, DALY, and incidence counts and age-standardized rates of tension-type headache in 2021, and percentage changes from 1990 to 2021, by country.

## Abbreviations

The following abbreviations are used in this manuscript:

TTH	tension-type headache
GBD	Global Burden of Disease
SDI	socio-demographic index
GATHER	Guidelines for Accurate and Transparent Health Estimates Reporting
IHME	Institute for Health Metrics and Evaluation
ICHD-3	International Classification of Headache Disorders, 3rd edition
ICD	International Classification of Diseases
MOH	medication overuse headache
DALYs	Disability-adjusted life years
YLLs	years of life lost
YLDs	years lived with disability
UI	uncertainty intervals
