# Supplementary material for: Trends in the Burden of Headache Disorders in Europe, 1990–2021: A Systematic Analysis from the Global Burden of Disease Study 2021
Source: J Clin Med. 2025 Oct 1;14(19):6966. doi: 10.3390/jcm14196966 (PMC12525519; doi:10.3390/jcm14196966)
Supplement: Supplementary file 1 [file jcm-14-06966-s001.zip › jcm-3810907-supplementary.pdf]

Supplementary materials

Supplementary methods 6

Supplementary Tables 8

Table S1a: Prevalence, DALY, incidence, and YLD counts and age-standardized rates of headache disorders in 1990 and 2021, and percentage changes of age-standardized rate from 1990 to 2021, in global and Europe *8*

Table S1b: Prevalence, DALY, incidence, and YLD counts and age-standardized rates of migraine in 1990 and 2021, and percentage changes of age-standardized rate from 1990 to 2021, in global and Europe *9*

Table S1c: Prevalence, DALY, incidence, and YLD counts and age-standardized rates of tension-type headache in 1990 and 2021, and percentage changes of age-standardized rate from 1990 to 2021, in global and Europe *10*

Table S2a: Counts and age-standardized prevalence rates of headache disorders from 1990 to 2021, by sex *11*

Table S2b: Counts and age-standardized DALY rates of headache disorders from 1990 to 2021, by sex *14*

Table S2c: Counts and age-standardized incidence rates of headache disorders from 1990 to 2021, by sex *17*

Table S2d: Counts and age-standardized YLD rates of headache disorders from 1990 to 2021, by sex *20*

Table S2e: Counts and age-standardized prevalence rates of migraine from 1990 to 2021, by sex *23*

Table S2f: Counts and age-standardized DALY rates of migraine from 1990 to 2021, by sex *26*

Table S2g: Counts and age-standardized incidence rates of migraine from 1990 to 2021, by sex *29*

Table S2h: Counts and age-standardized YLD rates of migraine from 1990 to 2021, by sex *32*

Table S2i: Counts and age-standardized prevalence rates of tension-type headache from 1990 to 2021, by sex *35*

Table S2j: Counts and age-standardized DALY rates of tension-type headache from 1990 to 2021, by sex *38*

Table S2k: Counts and age-standardized incidence rates of tension-type headache from 1990 to

2021, by sex 41

Table S2l: Counts and age-standardized YLD rates of tension-type headache from 1990 to 2021, by sex 44

Table S3a: Counts and age-standardized prevalence rates of headache disorders in 2021, and percentage changes from 1990 to 2021, by country and sex 47

Table S3b: Counts and age-standardized DALY rates of headache disorders in 2021, and percentage changes from 1990 to 2021, by country and sex 51

Table S3c: Counts and age-standardized incidence rates of headache disorders in 2021, and percentage changes from 1990 to 2021, by country and sex 55

Table S3d: Counts and age-standardized YLD rates of headache disorders in 2021, and percentage changes from 1990 to 2021, by country and sex 59

Table S3e: Counts and age-standardized prevalence rates of migraine in 2021, and percentage changes from 1990 to 2021, by country and sex 63

Table S3f: Counts and age-standardized DALY rates of migraine in 2021, and percentage changes from 1990 to 2021, by country and sex 67

Table S3g: Counts and age-standardized incidence rates of migraine in 2021, and percentage changes from 1990 to 2021, by country and sex 71

Table S3h: Counts and age-standardized YLD rates of migraine in 2021, and percentage changes from 1990 to 2021, by country and sex 75

Table S3i: Counts and age-standardized prevalence rates of tension-type headache in 2021, and percentage changes from 1990 to 2021, by country and sex 79

Table S3j: Counts and age-standardized DALY rates of tension-type headache in 2021, and percentage changes from 1990 to 2021, by country and sex 83

Table S3k: Counts and age-standardized incidence rates of tension-type headache in 2021, and percentage changes from 1990 to 2021, by country and sex 87

Table S3l: Counts and age-standardized YLD rates of tension-type headache in 2021, and percentage changes from 1990 to 2021, by country and sex 91

Table S4a: Prevalence, DALY, and incidence rates of headache disorders, by age and sex, 2021 95

Table S4b: Prevalence, DALY, and incidence rates of migraine, by age and sex, 2021 97

Table S4c: Prevalence, DALY, and incidence rates of tension-type headache, by age and sex, 2021 99

Table S5a: Prevalence, DALY, incidence, and YLD rates of headache disorders for both sexes, by age, 2021 101

Table S5b: Prevalence, DALY, incidence, and YLD rates of migraine for both sexes, by age, 2021 ...102

Table S5c: Prevalence, DALY, incidence, and YLD rates of tension-type headache for both sexes, by age, 2021...103

Table S6a: Socio-demographic index (SDI) with prevalence, DALY, and incidence counts and age-standardized rates of headache disorders in 2021, and percentage changes from 1990 to 2021, by country...104

Table S6b: Socio-demographic index (SDI) with prevalence, DALY, and incidence counts and age-standardized rates of migraine in 2021, and percentage changes from 1990 to 2021, by country...109

Table S6c: Socio-demographic index (SDI) with prevalence, DALY, and incidence counts and age-standardized rates of tension-type headache in 2021, and percentage changes from 1990 to 2021, by country...114

Supplementary Figures...119

Figure S1a: Changes in top ranks of age-standardized (A) prevalence, (B) DALY, (C) incidence, and (D) YLD rates for cause level 3 conditions from 1990 to 2021...119

Figure S1b: Changes in top ranks of age-standardized (A) prevalence, (B) DALY, (C) incidence, and (D) YLD rates for cause level 4 conditions from 1990 to 2021...120

Figure S2a: Age-standardized (A) prevalence, (B) DALY, (C) incidence, and (D) YLD rates and counts of headache disorders by year, 1990-2021...121

Figure S2b: Age-standardized (A) prevalence, (B) DALY, (C) incidence, and (D) YLD rates and counts of migraine by year, 1990-2021...122

Figure S2c: Age-standardized (A) prevalence, (B) DALY, (C) incidence, and (D) YLD rates and counts of tension-type headache by year, 1990-2021...123

Figure S3a: Age-standardized (A) prevalence and DALY, and (B) incidence and YLD rates and counts of headache disorders by year, 1990-2021...124

Figure S3b: Age-standardized (A) prevalence and DALY, and (B) incidence and YLD rates and counts of migraine by year, 1990-2021...125

Figure S3c: Age-standardized (A) prevalence and DALY, and (B) incidence and YLD rates and counts of tension-type headache by year, 1990-2021...126

Figure S4a: Region-wise percentage changes in (A) prevalence, (B) DALY, (C) incidence, and (D) YLD in headache disorders burden, 1990–2021...128

Figure S4b: Region-wise percentage changes in (A) prevalence, (B) DALY, (C) incidence, and (D) YLD in migraine burden, 1990–2021...130

Figure S4c: Region-wise percentage changes in (A) prevalence, (B) DALY, (C) incidence, and (D) YLD in tension-type headache burden, 1990–2021......132

Figure S5a: Changes in age-standardized (A) prevalence, (B) DALY, (C) incidence, and (D) YLD rate for headache disorders, by country, 2000-2010 and 2010-2021......134

Figure S5b: Changes in age-standardized (A) prevalence, (B) DALY, (C) incidence, and (D) YLD rate for migraine, by country, 2000-2010 and 2010-2021......136

Figure S5c: Changes in age-standardized (A) prevalence, (B) DALY, (C) incidence, and (D) YLD rate for tension-type headache, by country, 2000-2010 and 2010-2021......138

Figure S6a: Changes in age-standardized (A) prevalence, (B) DALY, (C) incidence, and (D) YLD rate for headache disorders, by country, 2010-2019 and 2019-2021......140

Figure S6b: Changes in age-standardized (A) prevalence, (B) DALY, (C) incidence, and (D) YLD rate for migraine, by country, 2010-2019 and 2019-2021......142

Figure S6c: Changes in age-standardized (A) prevalence, (B) DALY, (C) incidence, and (D) YLD rate for tension-type headache, by country, 2010-2019 and 2019-2021......144

Figure S7a: Annual changes in (A) prevalence, (B) DALY, (C) incidence, and (D) YLD rates of headache disorders comparing pre-pandemic (2010-2019) to pandemic periods (2019-2021), stratified by age group and by region......145

Figure S7b: Annual changes in (A) prevalence, (B) DALY, (C) incidence, and (D) YLD rates of migraine comparing pre-pandemic (2010-2019) to pandemic periods (2019-2021), stratified by age group and by region.......146

Figure S7c: Annual changes in (A) prevalence, (B) DALY, (C) incidence, and (D) YLD rates of tension-type headache comparing pre-pandemic (2010-2019) to pandemic periods (2019-2021), stratified by age group and by region......147

Figure S8a: Total (A) prevalence, (B) DALY, (C) incidence, and (D) YLD rates of headache disorders for both sexes, by age group and region, 2021......148

Figure S8b: Total (A) prevalence, (B) DALY, (C) incidence, and (D) YLD rates of migraine for both sexes, by age group and region, 2021......149

Figure S8c: Total (A) prevalence, (B) DALY, (C) incidence, and (D) YLD rates of tension-type headache for both sexes, by age group and region, 2021......150

Figure S9a: Age-standardized rates of (A) prevalence, (B) DALY, (C) incidence, and (D) YLD of headache disorders, by country and sex, 1990–2021......152

Figure S9b: Age-standardized rates of (A) prevalence, (B) DALY, (C) incidence, and (D) YLD of migraine, by country and sex, 1990–2021......154

Figure S9c: Age-standardized rates of (A) prevalence, (B) DALY, (C) incidence, and (D) YLD of

tension-type headache, by country and sex, 1990–2021..........156

Figure S10a: Age-standardized prevalence, DALY, and incidence rates and counts of headache disorders, by sex, 1990-2021..........157

Figure S10b: Age-standardized prevalence, DALY, and incidence rates and counts of migraine, by sex, 1990-2021..........158

Figure S10c: Age-standardized prevalence, DALY, and incidence rates and counts of tension-type headache, by sex, 1990-2021..........159

Figure S11a: Counts and rates of (A) prevalence and DALY, and (B) incidence and YLD of headache disorders, by year, age group, and sex..........161

Figure S11b: Counts and rates of (A) prevalence and DALY, and (B) incidence and YLD of migraine, by year, age group, and sex..........163

Figure S11c: Counts and rates of (A) prevalence and DALY, and (B) incidence and YLD of tension-type headache, by year, age group, and sex..........165

Figure S12a: Age-standardized (A) prevalence, (B) DALY, (C) incidence, and (D) YLD rates for headache disorders in Europe, by socio-demographic index (SDI), 2021..........166

Figure S12b: Age-standardized (A) prevalence, (B) DALY, (C) incidence, and (D) YLD rates for migraine in Europe, by socio-demographic index (SDI), 2021..........167

Figure S12c: Age-standardized (A) prevalence, (B) DALY, (C) incidence, and (D) YLD rates for tension-type headache in Europe, by socio-demographic index (SDI), 2021..........168

Supplementary methods

GBD 2021 classifies causes into a four-level hierarchy. Level 1 causes encompass aggregates of non-communicable diseases; injuries; and a combined category of maternal and neonatal disorders, infectious diseases, and nutritional deficiencies. At Level 2, there are 22 aggregated groupings of diseases and injuries, such as tuberculosis, cardiovascular diseases, respiratory infections, and transport injuries. Level 3 includes specific causes like stroke and road injuries. For some, Level 3 is the most detailed classification, while others have further detailed categories at Level 4, such as hemorrhagic stroke, latent tuberculosis infection, and pedestrian road injuries. Detailed levels of causes are explained at the GBD 2021 website (https://www.healthdata.org/research-analysis/diseases-injuries/factsheets-overview/about-disease-injury-impairment). Based on these criteria, headache disorders are classified as Level 3, while migraine and TTH are classified as Level 4.

Search Terms

The procedures for collecting data on non-fatal outcomes and mortality have been thoroughly explained in earlier research. Estimates for Disability-adjusted life years (DALYs) and years lived with disability (YLDs) related to headache disorders, migraine, and TTH were obtained from population-based data, survey microdata, registries, surveillance systems, and Systematic evaluations of reports, regardless of publication status. The IHME at the University of Washington conducted searches in various databases including Embase, PubMed, System for Information on Gray Literature in Europe, CAP abstracts, Medline, CINAHL, and the World Health Organization Library Information System to gather GBD data. The search string for migraine was: (("migraine disorders"[MeSH Terms] OR “migraine” [All Fields]) AND (“prevalence”[Title/Abstract] OR “incidence”[Title/Abstract] OR “remission”[Title/Abstract] OR epidemiology[Title/Abstract])). For TTH, the search

string was: (("headache"[MeSH Terms]) OR ("headache"[Title/Abstract] AND "tension" [Title/Abstract])) AND ("epidemiology"[Title/Abstract] OR "prevalence" [Title/Abstract] OR "incidence"[Title/Abstract] OR "remission"[Title/Abstract])). For medication overuse headache, the search string was: (("headache"[MeSH Terms] OR "headache"[Title/Abstract]) AND ("pharmaceutical preparations"[MeSH Terms] OR "pharmaceutical preparations" [Title/Abstract] OR "medication"[Title/Abstract]) AND ("epidemiology"[Title/Abstract] OR "prevalence"[Title/Abstract] OR "incidence" [Title/Abstract] OR "remission" [Title/Abstract])) without language restrictions. Estimates for medication overuse headache were combined with those for migraine and TTH.

Then, IHME excluded studies or datasets with small sample sizes (<150), review articles, non-population sample studies, or those lacking clear subpopulation definition. These datasets were then incorporated into the Global Health Data Exchange, where they were analyzed using DisMod-MR 2.1, a Bayesian meta-regression tool.

Supplementary Tables

Table S1a: Prevalence, DALY, incidence, and YLD counts and age-standardized rates of headache disorders in 1990 and 2021, and percentage changes of age-standardized rate from 1990 to 2021, in global and Europe

	Global					Europe				
	**Absolute number, 1990**	**Age-standardized rate, per 100 000 people, 1990**	**Absolute number, 2021**	**Age-standardized rate, per 100 000 people, 2021**	**Percentage Change, 1990-2021**	**Absolute number, 1990**	**Age-standardized rate, per 100 000 people, 1990**	**Absolute number, 2021**	**Age-standardized rate, per 100 000 people, 2021**	**Percentage Change, 1990-2021**
**Prevalence**	1787302945.09 (1648822201.72 to 1937346555.85)	34486.61 (31872.81 to 37187.63)	2808876481.80 (2599555367.51 to 3028767343.34)	34574.00 (32017.69 to 37318.55)	0.00% (0.00% to 0.01%)	345029404.43 (318156326.29 to 371460760.12)	40799.81 (37726.20 to 44018.84)	370591630.52 (340862080.96 to 397397185.77)	40685.82 (37620.80 to 43852.02)	0.00% (-0.01% to 0.00%)
**DALYs**	30260883.92 (5963391.97 to 64833434.46)	583.75 (122.49 to 1231.64)	47975675.06 (9800212.26 to 100667852.52)	588.39 (117.58 to 1245.36)	0.01% (-0.04% to 0.02%)	5891748.16 (1583812.42 to 12128651.80)	693.03 (177.31 to 1449.58)	6345063.09 (1796988.70 to 12906373.08)	696.22 (173.05 to 1449.93)	0.00% (-0.02% to 0.02%)
**Incidence**	533794823.90 (472464996.93 to 591430931.57)	10097.25 (8965.19 to 11186.25)	809226480.19 (717818771.04 to 895990201.46)	10084.51 (8956.48 to 11170.76)	0.00% (-0.01% to 0.00%)	100053721.33 (88528726.15 to 111403577.39)	12143.81 (10689.35 to 13495.53)	104292837.24 (92399738.42 to 115349658.98)	12070.27 (10635.74 to 13416.36)	-0.01% (-0.01% to 0.00%)
**YLDs**	30260883.92 (5963391.97 to 64833434.46)	583.75 (122.49 to 1231.64)	47975675.06 (9800212.26 to 100667852.52)	588.39 (117.58 to 1245.36)	0.01% (-0.04% to 0.02%)	5891748.16 (1583812.42 to 12128651.80)	693.03 (177.31 to 1449.58)	6345063.09 (1796988.70 to 12906373.08)	696.22 (173.05 to 1449.93)	0.00% (-0.02% to 0.02%)

Table S1b: Prevalence, DALY, incidence, and YLD counts and age-standardized rates of migraine in 1990 and 2021, and percentage changes of age-standardized rate from 1990 to 2021, in global and Europe

	Global					Europe				
	**Absolute number, 1990**	**Age-standardized rate, per 100 000 people, 1990**	**Absolute number, 2021**	**Age-standardized rate, per 100 000 people, 2021**	**Percentage Change, 1990-2021**	**Absolute number, 1990**	**Age-standardized rate, per 100 000 people, 1990**	**Absolute number, 2021**	**Age-standardized rate, per 100 000 people, 2021**	**Percentage Change, 1990-2021**
**Prevalence**	732564462.68 (624559243.92 to 847058436.26)	14027.65 (12063.37 to 16078.07)	1158432823.82 (995861966.38 to 1331312506.13)	14246.55 (12194.12 to 16378.70)	0.02% (0.00% to 0.03%)	134693058.36 (116329423.36 to 155047785.69)	16783.83 (14495.57 to 19320.19)	143542170.14 (124859309.64 to 165307694.92)	16916.64 (14714.84 to 19481.74)	0.01% (0.00% to 0.02%)
**DALYs**	27412196.29 (4076605.01 to 60325805.84)	526.76 (83.36 to 1145.92)	43378889.81 (6732642.21 to 95079454.09)	532.70 (80.57 to 1167.71)	0.01% (-0.04% to 0.03%)	5203117.75 (1068484.67 to 11017419.82)	648.35 (133.14 to 1372.86)	5577086.08 (1234081.57 to 11719998.01)	657.27 (145.44 to 1381.22)	0.01% (-0.02% to 0.02%)
**Incidence**	63496590.82 (55194751.45 to 72208003.40)	1136.90 (995.14 to 1287.76)	90183386.87 (78857600.46 to 101838162.48)	1153.20 (1006.07 to 1304.49)	0.01% (0.01% to 0.02%)	9470368.50 (8249961.74 to 10780895.79)	1180.08 (1028.01 to 1343.39)	9057610.76 (7881493.51 to 10303044.20)	1067.45 (928.84 to 1214.23)	0.01% (0.01% to 0.02%)
**YLDs**	27412196.29 (4076605.01 to 60325805.84)	526.76 (83.36 to 1145.92)	43378889.81 (6732642.21 to 95079454.09)	532.70 (80.57 to 1167.71)	0.01% (-0.04% to 0.03%)	5203117.75 (1068484.67 to 11017419.82)	648.35 (133.14 to 1372.86)	5577086.08 (1234081.57 to 11719998.01)	657.27 (145.44 to 1381.22)	0.01% (-0.02% to 0.02%)

Table S1c: Prevalence, DALY, incidence, and YLD counts and age-standardized rates of tension-type headache in 1990 and 2021, and percentage changes of age-standardized rate from 1990 to 2021, in global and Europe

	Global					Europe				
	**Absolute number, 1990**	**Age-standardized rate, per 100 000 people, 1990**	**Absolute number, 2021**	**Age-standardized rate, per 100 000 people, 2021**	**Percentage Change, 1990-2021**	**Absolute number, 1990**	**Age-standardized rate, per 100 000 people, 1990**	**Absolute number, 2021**	**Age-standardized rate, per 100 000 people, 2021**	**Percentage Change, 1990-2021**
**Prevalence**	1286366671.75 (1122503420.79 to 1467160187.52)	24904.85 (21960.05 to 28038.80)	2011612877.49 (1776544390.83 to 2270860638.85)	24764.77 (21863.62 to 27954.74)	-0.01% (-0.01% to 0.00%)	263085772.75 (234474303.61 to 296486651.56)	31057.91 (27533.49 to 35153.24)	282494328.80 (252165015.17 to 313416190.31)	30810.85 (27241.14 to 34786.17)	-0.01% (-0.02% to 0.00%)
**DALYs**	2848687.62 (820890.75 to 9563745.41)	56.99 (16.79 to 186.13)	4596785.25 (1347300.84 to 15012932.75)	55.69 (16.13 to 185.07)	-0.02% (-0.05% to 0.01%)	688630.41 (213826.80 to 2188953.53)	78.70 (23.92 to 257.25)	767977.02 (245879.07 to 2405182.64)	77.79 (23.49 to 256.54)	-0.01% (-0.06% to 0.02%)
**Incidence**	470298233.07 (408471892.00 to 527847536.34)	8960.34 (7815.06 to 10074.35)	719043093.32 (629219079.87 to 804949048.83)	8931.31 (7788.21 to 10020.83)	0.00% (-0.01% to 0.00%)	90583352.83 (78895825.05 to 101724244.72)	10911.18 (9459.09 to 12276.46)	95235226.48 (83299117.39 to 105938467.33)	10822.21 (9430.78 to 12181.47)	-0.01% (-0.02% to 0.00%)
**YLDs**	2848687.62 (820890.75 to 9563745.41)	56.99 (16.79 to 186.13)	4596785.25 (1347300.84 to 15012932.75)	55.69 (16.13 to 185.07)	-0.02% (-0.05% to 0.01%)	688630.41 (213826.80 to 2188953.53)	78.70 (23.92 to 257.25)	767977.02 (245879.07 to 2405182.64)	77.79 (23.49 to 256.54)	-0.01% (-0.06% to 0.02%)

Table S2a: Counts and age-standardized prevalence rates of headache disorders from 1990 to 2021, by sex

	Both		Male		Female	
	Absolute number	Age-standardized rate, per 100 000 people	Absolute number	Age-standardized rate, per 100 000 people	Absolute number	Age-standardized rate, per 100 000 people
**1990**	345029404.43 (318156326.29 to 371460760.12)	40799.81 (37726.20 to 44018.84)	151259857.61 (138369342.54 to 165391544.40)	36978.04 (33874.21 to 40379.84)	193769546.82 (180151061.59 to 206759578.38)	44454.94 (41361.06 to 47620.92)
**1991**	346626513.07 (319601998.41 to 373190305.10)	40761.64 (37702.06 to 43966.34)	151995279.01 (139061345.73 to 166145099.87)	36929.53 (33818.52 to 40344.72)	194631234.07 (180983551.62 to 207613181.77)	44432.82 (41341.92 to 47587.16)
**1992**	348249199.72 (321208313.63 to 374822145.32)	40726.71 (37678.48 to 43913.05)	152724606.65 (139804830.68 to 166929468.90)	36885.37 (33757.28 to 40298.30)	195524593.07 (181875046.64 to 208585781.37)	44411.97 (41320.02 to 47555.39)
**1993**	349788938.01 (322655595.41 to 376339064.08)	40694.70 (37635.86 to 43866.03)	153394923.14 (140506483.14 to 167729798.91)	36846.05 (33702.85 to 40261.43)	196394014.87 (182781374.93 to 209543753.50)	44390.39 (41290.26 to 47522.40)
**1994**	351050034.24 (324040073.15 to 377568788.58)	40667.11 (37595.96 to 43836.01)	153915340.43 (141052198.04 to 168245615.58)	36813.36 (33661.51 to 40235.35)	197134693.81 (183529615.26 to 210345783.99)	44369.52 (41261.75 to 47483.22)
**1995**	352213677.79 (325390779.63 to 378792899.91)	40643.67 (37558.85 to 43812.01)	154385616.01 (141351736.16 to 168670461.66)	36787.98 (33652.44 to 40237.99)	197828061.78 (184211880.46 to 211072466.62)	44349.04 (41238.85 to 47439.25)
**1996**	353427808.86 (326933525.04 to 379735445.55)	40625.94 (37604.62 to 43749.02)	154882180.87 (142046802.37 to 169117996.73)	36767.62 (33666.62 to 40195.55)	198545627.99 (185015893.82 to 211545034.80)	44335.90 (41275.52 to 47407.87)
**1997**	354679041.81 (328575889.11 to 380753811.40)	40612.70 (37659.75 to 43712.13)	155393762.64 (142700753.73 to 169437909.05)	36748.43 (33655.25 to 40133.08)	199285279.16 (185767516.69 to 212036019.54)	44331.66 (41335.57 to 47360.85)
**1998**	356005997.95 (329973494.04 to 381839602.95)	40598.34 (37714.29 to 43678.84)	155951197.79 (143323829.81 to 169711002.49)	36727.64 (33650.31 to 40065.42)	200054800.16 (186672548.18 to 212633823.57)	44327.49 (41360.23 to 47314.00)
**1999**	357260534.85 (331309130.80 to 382813015.19)	40581.09 (37754.70 to 43627.01)	156486021.89 (143919438.89 to 170039293.55)	36705.42 (33642.11 to 40010.46)	200774512.96 (187623288.38 to 213287790.34)	44319.25 (41383.26 to 47265.16)
**2000**	358424126.86 (332759115.50 to 383668425.53)	40562.53 (37779.67 to 43555.38)	156989992.24 (144453365.15 to 170357173.06)	36685.86 (33633.35 to 39956.38)	201434134.63 (188324999.56 to 214111518.08)	44305.30 (41385.62 to 47244.09)

**2001**	359577565.34 (333610619.93 to 384870887.88)	40548.80 (37774.20 to 43534.63)	157513023.17 (144891012.25 to 170903147.55)	36676.32 (33624.91 to 39946.24)	202064542.17 (188928108.59 to 214884639.83)	44291.08 (41372.61 to 47232.44)
**2002**	360705152.96 (334368669.41 to 386037381.97)	40545.23 (37778.64 to 43538.98)	158038949.89 (145318551.30 to 171489169.02)	36679.48 (33635.59 to 39950.75)	202666203.07 (189432594.15 to 215625707.02)	44284.17 (41388.31 to 47227.05)
**2003**	361737176.65 (335073905.29 to 387133458.74)	40545.16 (37775.08 to 43554.35)	158521923.86 (145635769.80 to 171992250.17)	36686.75 (33651.43 to 39939.04)	203215252.79 (189878621.86 to 216299672.12)	44279.55 (41398.86 to 47223.44)
**2004**	362711721.90 (335962698.18 to 388021628.59)	40543.82 (37760.88 to 43561.96)	158979899.15 (145915133.42 to 172480691.48)	36691.74 (33664.02 to 39926.05)	203731822.75 (190509000.88 to 216939040.24)	44275.11 (41387.26 to 47220.40)
**2005**	363581098.50 (336966859.53 to 388988263.11)	40539.15 (37740.55 to 43568.32)	159371237.83 (146135799.55 to 172906660.82)	36690.34 (33669.62 to 39935.58)	204209860.67 (191000944.66 to 217535967.86)	44270.56 (41374.87 to 47219.55)
**2006**	364240076.33 (337585234.65 to 389878511.90)	40533.66 (37734.50 to 43564.98)	159652385.56 (146363597.40 to 173209731.58)	36685.94 (33668.75 to 39923.87)	204587690.78 (191300746.49 to 217868837.65)	44267.22 (41371.75 to 47216.79)
**2007**	364780840.52 (338121161.69 to 390604845.94)	40531.77 (37731.76 to 43564.48)	159880562.45 (146538887.24 to 173384907.52)	36684.98 (33668.63 to 39914.39)	204900278.06 (191562558.26 to 218162296.98)	44267.38 (41371.45 to 47217.95)
**2008**	365237557.44 (338591973.07 to 391063349.02)	40529.93 (37727.48 to 43563.43)	160065484.23 (146697949.78 to 173504441.98)	36683.17 (33665.62 to 39903.87)	205172073.21 (191779458.37 to 218481752.99)	44267.89 (41370.77 to 47219.97)
**2009**	365554662.47 (338917912.79 to 391394445.38)	40527.36 (37722.48 to 43561.18)	160188230.90 (146882715.86 to 173553394.47)	36680.41 (33661.33 to 39903.19)	205366431.57 (191915043.10 to 218701726.22)	44267.93 (41369.44 to 47222.08)
**2010**	365751188.67 (339124717.79 to 391617131.43)	40524.29 (37717.75 to 43557.35)	160253684.38 (147041009.41 to 173548519.47)	36676.89 (33655.82 to 39903.40)	205497504.29 (191988482.21 to 218848437.66)	44267.66 (41368.40 to 47223.77)
**2011**	366002843.63 (339302796.74 to 391979039.39)	40536.51 (37718.03 to 43561.95)	160372273.73 (147244157.83 to 173785757.39)	36693.85 (33664.01 to 39939.30)	205630569.90 (192069483.14 to 219042679.18)	44277.94 (41357.08 to 47227.16)
**2012**	366500923.64 (339629128.79 to 392490890.57)	40571.66 (37738.25 to 43589.04)	160664304.14 (147607135.24 to 174218969.12)	36741.23 (33699.92 to 40011.17)	205836619.50 (192107974.07 to 219313156.01)	44304.75 (41361.28 to 47247.41)
**2013**	367181022.70 (340343591.26 to 393152631.13)	40614.38 (37759.45 to 43642.53)	161086362.11 (147996407.68 to 174693488.54)	36799.58 (33745.65 to 40096.57)	206094660.59 (192222620.64 to 219639312.86)	44336.82 (41369.04 to 47271.52)

**2014**	367964853.03 (341176970.18 to 393900414.02)	40650.97 (37769.72 to 43682.45)	161583562.82 (148294530.51 to 175249979.54)	36851.41 (33785.22 to 40172.26)	206381290.20 (192463797.66 to 219988128.22)	44364.63 (41370.36 to 47296.01)
**2015**	368704555.60 (341583167.34 to 394775380.24)	40669.27 (37759.42 to 43707.53)	162062053.68 (148667814.81 to 175748367.23)	36880.73 (33807.10 to 40211.66)	206642501.92 (192594476.86 to 220275689.18)	44379.24 (41357.99 to 47305.17)
**2016**	369576512.63 (341959487.47 to 395969745.80)	40701.00 (37777.81 to 43735.93)	162612734.61 (149102230.69 to 176508773.50)	36924.39 (33841.56 to 40260.01)	206963778.02 (192720028.22 to 220641001.06)	44406.09 (41371.19 to 47387.47)
**2017**	370759059.40 (342510232.15 to 397726853.21)	40764.42 (37797.23 to 43821.77)	163330485.54 (149589415.17 to 177392289.52)	37004.13 (33889.36 to 40350.85)	207428573.86 (193007219.62 to 221187625.68)	44459.68 (41384.99 to 47491.27)
**2018**	371853446.33 (342910680.55 to 399248074.73)	40824.38 (37807.20 to 43932.02)	163993984.39 (149969433.92 to 178113884.38)	37078.95 (33962.64 to 40491.23)	207859461.94 (193270942.48 to 221724702.21)	44510.67 (41407.76 to 47575.53)
**2019**	372479909.52 (342923655.26 to 400136871.68)	40849.67 (37790.15 to 44004.21)	164397247.00 (150184114.38 to 178475962.47)	37112.42 (33967.50 to 40509.96)	208082662.52 (193271917.22 to 221918509.77)	44533.65 (41410.52 to 47624.76)
**2020**	370984933.99 (341242252.89 to 397802710.38)	40695.10 (37626.38 to 43853.81)	163559470.69 (149293711.01 to 177194953.50)	36919.38 (33781.03 to 40328.81)	207425463.31 (192744015.77 to 221130859.77)	44421.50 (41282.94 to 47498.23)
**2021**	370591630.52 (340862080.96 to 397397185.77)	40685.82 (37620.80 to 43852.02)	163430491.08 (149177588.93 to 177041537.85)	36910.27 (33762.87 to 40317.43)	207161139.44 (192553758.31 to 220826723.58)	44416.59 (41290.00 to 47491.58)
**Percentage Change, 1990-2021**	0.07% (0.06% to 0.09%)	0.00% (-0.01% to 0.00%)	0.08% (0.06% to 0.10%)	0.00% (-0.01% to 0.01%)	0.07% (0.05% to 0.09%)	0.00% (-0.01% to 0.01%)
**Statistical Analysis**	Spearman's ρ = 0.995 p-value < 0.001	Spearman's ρ = 0.044 p-value = 0.810	Spearman's ρ = 0.997 p-value < 0.001	Spearman's ρ = 0.193 p-value = 0.289	Spearman's ρ = 0.994 p-value < 0.001	Spearman's ρ = 0.102 p-value = 0.580
			Wilcoxon rank sum test between sex (count): p-value < 0.001<br>Wilcoxon rank sum test between sex (rate): p-value < 0.001			

Table S2b: Counts and age-standardized DALY rates of headache disorders from 1990 to 2021, by sex

	Both		Male		Female	
	Absolute number	Age-standardized rate, per 100 000 people	Absolute number	Age-standardized rate, per 100 000 people	Absolute number	Age-standardized rate, per 100 000 people
**1990**	5891748.16 (1583812.42 to 12128651.80)	693.03 (177.31 to 1449.58)	1974899.70 (515608.89 to 4145375.64)	477.96 (121.47 to 1008.77)	3916848.46 (1057242.13 to 7963031.06)	901.61 (227.71 to 1866.95)
**1991**	5925687.24 (1596400.37 to 12178527.82)	693.07 (176.66 to 1447.90)	1986907.86 (518629.78 to 4162960.00)	477.79 (121.01 to 1007.18)	3938779.38 (1063829.54 to 8007156.55)	902.19 (227.07 to 1867.78)
**1992**	5960165.39 (1607845.20 to 12253171.75)	693.22 (176.19 to 1449.69)	1998848.71 (522590.82 to 4195238.66)	477.72 (120.89 to 1010.83)	3961316.68 (1068232.90 to 8041800.81)	902.79 (226.06 to 1867.08)
**1993**	5992159.43 (1621479.44 to 12292164.07)	693.31 (176.17 to 1446.25)	2009034.34 (531130.72 to 4213618.51)	477.50 (121.28 to 1009.07)	3983125.08 (1072018.17 to 8086445.30)	903.34 (225.87 to 1865.55)
**1994**	6018465.78 (1628450.93 to 12359407.94)	693.36 (176.01 to 1444.98)	2017210.86 (533864.83 to 4223902.93)	477.31 (121.11 to 1007.52)	4001254.92 (1081514.80 to 8125768.51)	903.67 (226.20 to 1863.19)
**1995**	6042211.00 (1634587.19 to 12379288.47)	693.23 (175.43 to 1443.91)	2025027.14 (535079.54 to 4238616.52)	477.19 (121.08 to 1007.67)	4017183.86 (1081922.00 to 8140447.61)	903.52 (225.09 to 1860.46)
**1996**	6076003.68 (1654140.85 to 12453823.77)	694.22 (176.54 to 1443.93)	2034619.25 (541838.20 to 4265302.71)	477.46 (121.50 to 1010.63)	4041384.43 (1094586.64 to 8209522.85)	905.25 (225.92 to 1859.38)
**1997**	6123673.62 (1671163.92 to 12505875.87)	696.73 (177.71 to 1445.84)	2047253.83 (545602.16 to 4290712.62)	478.37 (121.54 to 1012.15)	4076419.79 (1108791.92 to 8277102.70)	909.38 (227.61 to 1862.83)
**1998**	6177850.48 (1697809.65 to 12624787.49)	699.74 (179.37 to 1452.16)	2061756.65 (554866.70 to 4313850.76)	479.50 (121.65 to 1017.76)	4116093.84 (1130981.28 to 8349476.18)	914.31 (230.84 to 1873.09)
**1999**	6226390.23 (1719153.88 to 12713813.79)	702.11 (180.51 to 1454.91)	2074823.32 (560758.82 to 4332274.49)	480.32 (122.38 to 1018.96)	4151566.90 (1143629.42 to 8413696.49)	918.31 (232.24 to 1880.96)
**2000**	6261203.67 (1729572.93 to 12774937.76)	703.02 (181.09 to 1456.20)	2085350.02 (566455.29 to 4351987.03)	480.66 (122.81 to 1019.23)	4175853.65 (1150465.75 to 8455648.77)	919.90 (233.40 to 1884.99)

**2001**	6284579.75 (1740241.17 to 12814829.31)	702.83 (181.45 to 1456.42)	2093651.49 (567170.00 to 4371587.17)	480.63 (122.31 to 1021.02)	4190928.25 (1155992.16 to 8480696.89)	919.69 (233.20 to 1889.47)
**2002**	6305091.51 (1746443.82 to 12848253.02)	702.64 (181.09 to 1456.74)	2100945.06 (571096.09 to 4373462.03)	480.64 (122.68 to 1018.76)	4204146.45 (1164410.37 to 8499310.03)	919.44 (233.10 to 1885.46)
**2003**	6324179.75 (1752398.25 to 12899082.03)	702.61 (181.20 to 1458.37)	2107696.46 (572963.91 to 4395301.15)	480.73 (122.58 to 1021.36)	4216483.30 (1169182.06 to 8528125.39)	919.40 (233.28 to 1888.12)
**2004**	6341910.45 (1767360.44 to 12950736.19)	702.54 (181.13 to 1460.27)	2113643.23 (573967.03 to 4422446.35)	480.70 (122.45 to 1026.00)	4228267.21 (1176568.94 to 8539995.03)	919.43 (232.54 to 1888.35)
**2005**	6360572.23 (1774510.22 to 12984624.75)	702.72 (180.90 to 1457.21)	2120420.01 (577421.24 to 4416301.75)	480.98 (121.67 to 1022.51)	4240152.23 (1185904.62 to 8579530.31)	919.65 (233.03 to 1885.63)
**2006**	6376459.38 (1784429.89 to 13002666.30)	703.07 (180.98 to 1458.83)	2125573.60 (579299.58 to 4424593.36)	481.23 (121.67 to 1023.81)	4250885.78 (1189352.88 to 8588943.87)	920.26 (232.73 to 1890.53)
**2007**	6391586.74 (1792395.22 to 13023643.84)	703.64 (180.92 to 1458.67)	2130993.28 (582078.46 to 4434857.28)	481.74 (121.90 to 1025.79)	4260593.46 (1198509.87 to 8613920.86)	921.04 (232.68 to 1889.57)
**2008**	6406698.20 (1804559.89 to 13030282.17)	704.43 (181.58 to 1462.12)	2136918.47 (586428.11 to 4447449.29)	482.53 (122.23 to 1024.91)	4269779.73 (1205946.71 to 8621535.24)	921.95 (233.18 to 1895.04)
**2009**	6418114.31 (1811896.70 to 13044628.86)	705.08 (181.63 to 1461.21)	2140661.09 (590192.62 to 4436582.98)	483.02 (123.04 to 1026.76)	4277453.23 (1210604.58 to 8632547.36)	922.87 (232.47 to 1892.49)
**2010**	6426613.72 (1820337.58 to 13051561.60)	705.63 (181.15 to 1464.11)	2143718.02 (592006.83 to 4443777.30)	483.47 (122.03 to 1026.94)	4282895.70 (1215158.72 to 8617514.25)	923.62 (232.84 to 1895.30)
**2011**	6433429.16 (1827530.21 to 13068070.24)	706.26 (181.43 to 1465.97)	2146528.02 (595628.50 to 4453196.05)	484.09 (122.54 to 1028.73)	4286901.14 (1221208.72 to 8640291.19)	924.34 (233.23 to 1897.35)
**2012**	6441752.15 (1840796.53 to 13101722.42)	707.19 (181.10 to 1468.83)	2150192.95 (599976.06 to 4443073.30)	484.90 (122.33 to 1031.80)	4291559.20 (1225733.49 to 8642013.24)	925.53 (232.69 to 1898.52)
**2013**	6453658.27 (1847603.93 to 13101318.02)	708.39 (180.45 to 1470.73)	2156790.81 (601302.38 to 4450741.54)	486.20 (122.16 to 1031.76)	4296867.47 (1226660.41 to 8650480.43)	926.83 (232.49 to 1902.69)

**2014**	6465059.94 (1855703.17 to 13135131.18)	709.29 (180.43 to 1476.97)	2163469.34 (605446.53 to 4470627.20)	487.25 (121.73 to 1033.04)	4301590.60 (1229647.74 to 8668704.82)	927.86 (233.22 to 1907.41)
**2015**	6474452.48 (1864039.47 to 13165444.57)	709.77 (180.63 to 1479.09)	2169550.74 (607368.07 to 4482051.16)	487.94 (122.54 to 1034.28)	4304901.74 (1232821.22 to 8663899.76)	928.48 (233.17 to 1906.12)
**2016**	6467040.41 (1858502.71 to 13136643.50)	708.43 (179.21 to 1475.14)	2172105.16 (612167.86 to 4480148.49)	487.80 (122.30 to 1035.01)	4294935.25 (1223355.02 to 8645401.65)	926.38 (231.63 to 1899.48)
**2017**	6445379.44 (1840759.74 to 13108407.19)	705.76 (177.01 to 1470.75)	2172119.97 (610537.53 to 4493855.97)	487.16 (121.79 to 1033.09)	4273259.47 (1208039.95 to 8626149.89)	922.12 (227.46 to 1892.67)
**2018**	6420069.49 (1814144.08 to 13051160.65)	702.78 (175.23 to 1462.87)	2170880.15 (607221.81 to 4487025.72)	486.32 (120.87 to 1032.78)	4249189.34 (1195748.44 to 8575879.90)	917.42 (225.52 to 1881.90)
**2019**	6403434.72 (1800490.17 to 13022538.04)	701.07 (173.80 to 1461.56)	2169402.84 (605330.19 to 4479584.44)	485.67 (120.78 to 1029.55)	4234031.88 (1192380.07 to 8547342.37)	915.00 (224.49 to 1881.06)
**2020**	6365442.66 (1801710.91 to 12953725.47)	697.32 (173.27 to 1455.06)	2160360.81 (608609.46 to 4453954.89)	483.68 (120.34 to 1027.31)	4205081.85 (1195083.96 to 8480623.28)	909.78 (223.35 to 1874.43)
**2021**	6345063.09 (1796988.70 to 12906373.08)	696.22 (173.05 to 1449.93)	2156683.04 (610227.14 to 4441134.25)	483.36 (120.11 to 1021.87)	4188380.05 (1191901.20 to 8451079.17)	908.18 (223.50 to 1866.52)
**Percentage Change, 1990-2021**	0.08% (0.04% to 0.14%)	0.00% (-0.02% to 0.02%)	0.09% (0.06% to 0.17%)	0.01% (-0.01% to 0.03%)	0.07% (0.04% to 0.13%)	0.01% (-0.02% to 0.03%)
**Statistical Analysis**	Spearman's ρ = 0.870 p-value < 0.001	Spearman's ρ = 0.658 p-value < 0.001	Spearman's ρ = 0.974 p-value < 0.001	Spearman's ρ = 0.924 p-value < 0.001	Spearman's ρ = 0.784 p-value < 0.001	Spearman's ρ = 0.625 p-value < 0.001
			Wilcoxon rank sum test between sex (count): p-value < 0.001<br>Wilcoxon rank sum test between sex (rate): p-value < 0.001			

Table S2c: Counts and age-standardized incidence rates of headache disorders from 1990 to 2021, by sex

	Both		Male		Female	
	Absolute number	Age-standardized rate, per 100 000 people	Absolute number	Age-standardized rate, per 100 000 people	Absolute number	Age-standardized rate, per 100 000 people
**1990**	100053721.33 (88528726.15 to 111403577.39)	12143.81 (10689.35 to 13495.53)	46307595.33 (40761679.08 to 51812109.90)	11542.36 (10150.17 to 12846.05)	53746126.00 (47761724.74 to 59619536.98)	12726.54 (11243.61 to 14156.08)
**1991**	100502724.54 (89028054.48 to 111856875.73)	12136.55 (10687.30 to 13492.24)	46530481.98 (41007570.16 to 52040071.71)	11534.30 (10147.94 to 12838.57)	53972242.56 (47971522.27 to 59821390.31)	12720.62 (11239.59 to 14142.34)
**1992**	100925979.81 (89445299.36 to 112303693.77)	12128.58 (10686.26 to 13487.62)	46740345.68 (41236213.25 to 52241292.02)	11526.29 (10146.07 to 12826.69)	54185634.13 (48179249.46 to 60011483.11)	12713.33 (11234.01 to 14127.61)
**1993**	101305305.77 (89810745.86 to 112705133.84)	12120.91 (10686.45 to 13482.64)	46924989.22 (41431832.28 to 52403324.68)	11519.22 (10145.66 to 12819.68)	54380316.55 (48337936.04 to 60185690.04)	12705.63 (11227.48 to 14111.96)
**1994**	101579419.95 (90099713.15 to 112987752.47)	12113.84 (10687.22 to 13479.44)	47052901.41 (41570100.69 to 52494573.38)	11513.16 (10146.30 to 12813.12)	54526518.54 (48423308.07 to 60318187.89)	12697.95 (11221.09 to 14095.63)
**1995**	101801806.06 (90356191.64 to 113239637.72)	12107.78 (10688.86 to 13477.93)	47153474.30 (41687192.98 to 52556642.95)	11508.42 (10148.74 to 12807.58)	54648331.76 (48495246.99 to 60446301.11)	12690.92 (11213.32 to 14081.48)
**1996**	102002068.33 (90568942.26 to 113418868.46)	12101.07 (10674.35 to 13461.93)	47249568.08 (41744285.28 to 52664229.51)	11503.70 (10138.13 to 12779.63)	54752500.25 (48633799.97 to 60549860.16)	12682.79 (11201.74 to 14074.59)
**1997**	102168989.33 (90774111.46 to 113518948.10)	12092.20 (10660.90 to 13438.42)	47338094.14 (41808453.55 to 52818939.57)	11497.61 (10132.09 to 12772.10)	54830895.19 (48807473.44 to 60615317.01)	12671.99 (11185.31 to 14056.17)
**1998**	102329972.63 (90986776.49 to 113667807.45)	12081.71 (10655.26 to 13418.50)	47429008.07 (41893173.01 to 52979857.09)	11490.07 (10134.44 to 12775.73)	54900964.56 (48955968.64 to 60664819.38)	12659.51 (11167.42 to 14035.57)
**1999**	102473286.07 (91064738.12 to 113808281.42)	12071.37 (10652.46 to 13401.12)	47510505.65 (41977568.61 to 53045302.59)	11481.97 (10123.75 to 12779.30)	54962780.42 (49073795.49 to 60696034.35)	12647.90 (11155.75 to 14011.38)
**2000**	102619499.11 (91110609.87 to 113963304.70)	12063.98 (10652.15 to 13387.96)	47585431.32 (42059667.71 to 53156691.10)	11475.42 (10107.37 to 12783.32)	55034067.79 (49186229.68 to 60703955.55)	12640.48 (11149.75 to 13987.72)

**2001**	102790630.06 (91237661.85 to 114189876.34)	12061.18 (10647.04 to 13389.21)	47669016.23 (42224538.94 to 53275393.80)	11472.83 (10110.36 to 12780.44)	55121613.83 (49291305.88 to 60789833.62)	12638.18 (11148.24 to 13993.75)
**2002**	102953826.77 (91408980.34 to 114317879.43)	12062.11 (10645.27 to 13393.66)	47751132.79 (42357009.94 to 53364821.58)	11474.38 (10118.75 to 12781.82)	55202693.98 (49368969.15 to 60899904.45)	12639.08 (11154.34 to 14002.49)
**2003**	103091293.16 (91592139.17 to 114301761.03)	12064.15 (10643.97 to 13399.61)	47820626.40 (42476003.09 to 53450319.01)	11477.06 (10128.73 to 12775.47)	55270666.76 (49427842.92 to 60996304.05)	12640.96 (11166.42 to 14013.12)
**2004**	103214104.96 (91753870.59 to 114485496.92)	12065.38 (10641.32 to 13405.55)	47884429.70 (42574802.77 to 53540792.75)	11478.72 (10125.42 to 12768.57)	55329675.26 (49479747.48 to 61080037.04)	12642.29 (11177.60 to 14020.62)
**2005**	103301386.64 (91884703.05 to 114683258.61)	12064.37 (10639.93 to 13408.60)	47929227.73 (42550313.85 to 53571382.33)	11477.70 (10118.00 to 12758.77)	55372158.91 (49525612.71 to 61141814.69)	12641.84 (11186.46 to 14025.97)
**2006**	103328903.33 (91934280.91 to 114717658.78)	12062.01 (10639.43 to 13405.09)	47944711.68 (42489677.39 to 53593187.95)	11475.27 (10116.03 to 12754.65)	55384191.65 (49553171.69 to 61177639.00)	12640.06 (11186.60 to 14025.05)
**2007**	103327000.05 (91957684.03 to 114643269.22)	12060.23 (10638.86 to 13401.41)	47948091.81 (42446637.79 to 53628183.92)	11473.69 (10114.07 to 12751.54)	55378908.24 (49571689.32 to 61172805.28)	12638.51 (11185.89 to 14023.56)
**2008**	103310902.21 (91895365.93 to 114534815.78)	12058.23 (10637.58 to 13397.85)	47945837.98 (42421678.27 to 53636570.65)	11471.86 (10111.09 to 12747.98)	55365064.22 (49547332.25 to 61131448.51)	12636.74 (11183.94 to 14021.20)
**2009**	103280124.91 (91865139.82 to 114388775.28)	12056.21 (10635.76 to 13394.22)	47935857.37 (42388445.25 to 53629057.69)	11469.92 (10107.98 to 12744.15)	55344267.54 (49504625.71 to 61067534.55)	12635.09 (11181.26 to 14019.20)
**2010**	103233467.54 (91814492.63 to 114219551.11)	12054.36 (10633.89 to 13390.56)	47916454.90 (42358729.67 to 53567546.28)	11467.99 (10105.29 to 12740.28)	55317012.65 (49456864.22 to 61061043.51)	12633.77 (11178.59 to 14018.44)
**2011**	103204794.80 (91793201.09 to 114132857.86)	12054.63 (10633.33 to 13390.70)	47903646.82 (42368109.12 to 53449634.07)	11468.18 (10103.22 to 12734.92)	55301147.97 (49395896.68 to 61057614.21)	12634.62 (11184.44 to 14020.18)
**2012**	103244200.07 (91836536.87 to 114172282.34)	12057.45 (10635.34 to 13394.73)	47926316.79 (42374843.26 to 53373172.79)	11471.07 (10103.35 to 12740.45)	55317883.27 (49384169.62 to 61054824.82)	12637.88 (11189.42 to 14025.08)
**2013**	103338658.31 (91882096.87 to 114280353.39)	12060.55 (10638.34 to 13399.71)	47982698.94 (42407135.84 to 53351325.36)	11474.43 (10104.54 to 12747.59)	55355959.37 (49401087.17 to 61071093.25)	12641.35 (11194.20 to 14029.41)

**2014**	103490388.37 (91939598.79 to 114437789.01)	12062.39 (10641.96 to 13403.30)	48073325.12 (42462727.34 to 53415214.63)	11476.67 (10105.79 to 12753.29)	55417063.25 (49441512.73 to 61137880.10)	12643.65 (11197.75 to 14031.17)
**2015**	103668668.48 (92019409.76 to 114611718.35)	12061.69 (10644.57 to 13402.90)	48183183.24 (42533517.74 to 53489658.25)	11476.63 (10106.70 to 12754.70)	55485485.24 (49485892.02 to 61208373.00)	12643.33 (11199.37 to 14028.65)
**2016**	103907504.58 (92114966.33 to 114899434.58)	12067.52 (10646.24 to 13413.47)	48321290.48 (42627126.69 to 53645978.79)	11482.71 (10100.50 to 12768.94)	55586214.10 (49487839.64 to 61325870.16)	12649.87 (11209.47 to 14047.81)
**2017**	104229326.80 (92331371.96 to 115282365.49)	12083.08 (10654.49 to 13435.91)	48495276.01 (42755540.86 to 53882073.77)	11497.80 (10115.37 to 12804.58)	55734050.79 (49575831.11 to 61495712.54)	12666.75 (11227.77 to 14066.38)
**2018**	104530807.72 (92560412.43 to 115663515.73)	12098.76 (10662.99 to 13459.25)	48655119.84 (42892242.54 to 54142124.33)	11512.73 (10128.05 to 12829.40)	55875687.88 (49664460.57 to 61677845.37)	12683.89 (11239.06 to 14080.06)
**2019**	104703329.13 (92699747.90 to 115909256.17)	12105.68 (10668.93 to 13471.62)	48750757.52 (42984994.93 to 54330369.71)	11519.26 (10131.93 to 12840.56)	55952571.62 (49706986.64 to 61780612.60)	12691.91 (11239.25 to 14066.57)
**2020**	104418693.89 (92480133.84 to 115484807.75)	12073.40 (10637.42 to 13420.18)	48604772.72 (42890791.67 to 54025164.39)	11481.64 (10094.70 to 12779.65)	55813921.17 (49585783.22 to 61508957.79)	12665.75 (11217.23 to 14036.82)
**2021**	104292837.24 (92399738.42 to 115349658.98)	12070.27 (10635.74 to 13416.36)	48555908.52 (42852324.20 to 53951770.13)	11479.08 (10093.46 to 12782.11)	55736928.71 (49546328.77 to 61419006.97)	12662.55 (11215.82 to 14029.50)
**Percentage Change, 1990-2021**	0.04% (0.02% to 0.07%)	-0.01% (-0.01% to 0.00%)	0.05% (0.02% to 0.08%)	-0.01% (-0.01% to 0.00%)	0.04% (0.01% to 0.06%)	-0.01% (-0.01% to 0.00%)
**Statistical Analysis**	Spearman's ρ = 0.967 p-value < 0.001	Spearman's ρ = -0.467 p-value = 0.007	Spearman's ρ = 0.972 p-value < 0.001	Spearman's ρ = -0.390 p-value = 0.027	Spearman's ρ = 0.951 p-value < 0.001	Spearman's ρ = -0.353 p-value = 0.047
			Wilcoxon rank sum test between sex (count): p-value < 0.001 Wilcoxon rank sum test between sex (rate): p-value < 0.001			

Table S2d: Counts and age-standardized YLD rates of headache disorders from 1990 to 2021, by sex

	Both		Male		Female	
	**Absolute number**	**Age-standardized rate, per 100 000 people**	**Absolute number**	**Age-standardized rate, per 100 000 people**	**Absolute number**	**Age-standardized rate, per 100 000 people**
**1990**	5891748.16 (1583812.42 to 12128651.80)	693.03 (177.31 to 1449.58)	1974899.70 (515608.89 to 4145375.64)	477.96 (121.47 to 1008.77)	3916848.46 (1057242.13 to 7963031.06)	901.61 (227.71 to 1866.95)
**1991**	5925687.24 (1596400.37 to 12178527.82)	693.07 (176.66 to 1447.90)	1986907.86 (518629.78 to 4162960.00)	477.79 (121.01 to 1007.18)	3938779.38 (1063829.54 to 8007156.55)	902.19 (227.07 to 1867.78)
**1992**	5960165.39 (1607845.20 to 12253171.75)	693.22 (176.19 to 1449.69)	1998848.71 (522590.82 to 4195238.66)	477.72 (120.89 to 1010.83)	3961316.68 (1068232.90 to 8041800.81)	902.79 (226.06 to 1867.08)
**1993**	5992159.43 (1621479.44 to 12292164.07)	693.31 (176.17 to 1446.25)	2009034.34 (531130.72 to 4213618.51)	477.50 (121.28 to 1009.07)	3983125.08 (1072018.17 to 8086445.30)	903.34 (225.87 to 1865.55)
**1994**	6018465.78 (1628450.93 to 12359407.94)	693.36 (176.01 to 1444.98)	2017210.86 (533864.83 to 4223902.93)	477.31 (121.11 to 1007.52)	4001254.92 (1081514.80 to 8125768.51)	903.67 (226.20 to 1863.19)
**1995**	6042211.00 (1634587.19 to 12379288.47)	693.23 (175.43 to 1443.91)	2025027.14 (535079.54 to 4238616.52)	477.19 (121.08 to 1007.67)	4017183.86 (1081922.00 to 8140447.61)	903.52 (225.09 to 1860.46)
**1996**	6076003.68 (1654140.85 to 12453823.77)	694.22 (176.54 to 1443.93)	2034619.25 (541838.20 to 4265302.71)	477.46 (121.50 to 1010.63)	4041384.43 (1094586.64 to 8209522.85)	905.25 (225.92 to 1859.38)
**1997**	6123673.62 (1671163.92 to 12505875.87)	696.73 (177.71 to 1445.84)	2047253.83 (545602.16 to 4290712.62)	478.37 (121.54 to 1012.15)	4076419.79 (1108791.92 to 8277102.70)	909.38 (227.61 to 1862.83)
**1998**	6177850.48 (1697809.65 to 12624787.49)	699.74 (179.37 to 1452.16)	2061756.65 (554866.70 to 4313850.76)	479.50 (121.65 to 1017.76)	4116093.84 (1130981.28 to 8349476.18)	914.31 (230.84 to 1873.09)
**1999**	6226390.23 (1719153.88 to 12713813.79)	702.11 (180.51 to 1454.91)	2074823.32 (560758.82 to 4332274.49)	480.32 (122.38 to 1018.96)	4151566.90 (1143629.42 to 8413696.49)	918.31 (232.24 to 1880.96)
**2000**	6261203.67 (1729572.93 to 12774937.76)	703.02 (181.09 to 1456.20)	2085350.02 (566455.29 to 4351987.03)	480.66 (122.81 to 1019.23)	4175853.65 (1150465.75 to 8455648.77)	919.90 (233.40 to 1884.99)

**2001**	6284579.75 (1740241.17 to 12814829.31)	702.83 (181.45 to 1456.42)	2093651.49 (567170.00 to 4371587.17)	480.63 (122.31 to 1021.02)	4190928.25 (1155992.16 to 8480696.89)	919.69 (233.20 to 1889.47)
**2002**	6305091.51 (1746443.82 to 12848253.02)	702.64 (181.09 to 1456.74)	2100945.06 (571096.09 to 4373462.03)	480.64 (122.68 to 1018.76)	4204146.45 (1164410.37 to 8499310.03)	919.44 (233.10 to 1885.46)
**2003**	6324179.75 (1752398.25 to 12899082.03)	702.61 (181.20 to 1458.37)	2107696.46 (572963.91 to 4395301.15)	480.73 (122.58 to 1021.36)	4216483.30 (1169182.06 to 8528125.39)	919.40 (233.28 to 1888.12)
**2004**	6341910.45 (1767360.44 to 12950736.19)	702.54 (181.13 to 1460.27)	2113643.23 (573967.03 to 4422446.35)	480.70 (122.45 to 1026.00)	4228267.21 (1176568.94 to 8539995.03)	919.43 (232.54 to 1888.35)
**2005**	6360572.23 (1774510.22 to 12984624.75)	702.72 (180.90 to 1457.21)	2120420.01 (577421.24 to 4416301.75)	480.98 (121.67 to 1022.51)	4240152.23 (1185904.62 to 8579530.31)	919.65 (233.03 to 1885.63)
**2006**	6376459.38 (1784429.89 to 13002666.30)	703.07 (180.98 to 1458.83)	2125573.60 (579299.58 to 4424593.36)	481.23 (121.67 to 1023.81)	4250885.78 (1189352.88 to 8588943.87)	920.26 (232.73 to 1890.53)
**2007**	6391586.74 (1792395.22 to 13023643.84)	703.64 (180.92 to 1458.67)	2130993.28 (582078.46 to 4434857.28)	481.74 (121.90 to 1025.79)	4260593.46 (1198509.87 to 8613920.86)	921.04 (232.68 to 1889.57)
**2008**	6406698.20 (1804559.89 to 13030282.17)	704.43 (181.58 to 1462.12)	2136918.47 (586428.11 to 4447449.29)	482.53 (122.23 to 1024.91)	4269779.73 (1205946.71 to 8621535.24)	921.95 (233.18 to 1895.04)
**2009**	6418114.31 (1811896.70 to 13044628.86)	705.08 (181.63 to 1461.21)	2140661.09 (590192.62 to 4436582.98)	483.02 (123.04 to 1026.76)	4277453.23 (1210604.58 to 8632547.36)	922.87 (232.47 to 1892.49)
**2010**	6426613.72 (1820337.58 to 13051561.60)	705.63 (181.15 to 1464.11)	2143718.02 (592006.83 to 4443777.30)	483.47 (122.03 to 1026.94)	4282895.70 (1215158.72 to 8617514.25)	923.62 (232.84 to 1895.30)
**2011**	6433429.16 (1827530.21 to 13068070.24)	706.26 (181.43 to 1465.97)	2146528.02 (595628.50 to 4453196.05)	484.09 (122.54 to 1028.73)	4286901.14 (1221208.72 to 8640291.19)	924.34 (233.23 to 1897.35)
**2012**	6441752.15 (1840796.53 to 13101722.42)	707.19 (181.10 to 1468.83)	2150192.95 (599976.06 to 4443073.30)	484.90 (122.33 to 1031.80)	4291559.20 (1225733.49 to 8642013.24)	925.53 (232.69 to 1898.52)
**2013**	6453658.27 (1847603.93 to 13101318.02)	708.39 (180.45 to 1470.73)	2156790.81 (601302.38 to 4450741.54)	486.20 (122.16 to 1031.76)	4296867.47 (1226660.41 to 8650480.43)	926.83 (232.49 to 1902.69)

**2014**	6465059.94 (1855703.17 to 13135131.18)	709.29 (180.43 to 1476.97)	2163469.34 (605446.53 to 4470627.20)	487.25 (121.73 to 1033.04)	4301590.60 (1229647.74 to 8668704.82)	927.86 (233.22 to 1907.41)
**2015**	6474452.48 (1864039.47 to 13165444.57)	709.77 (180.63 to 1479.09)	2169550.74 (607368.07 to 4482051.16)	487.94 (122.54 to 1034.28)	4304901.74 (1232821.22 to 8663899.76)	928.48 (233.17 to 1906.12)
**2016**	6467040.41 (1858502.71 to 13136643.50)	708.43 (179.21 to 1475.14)	2172105.16 (612167.86 to 4480148.49)	487.80 (122.30 to 1035.01)	4294935.25 (1223355.02 to 8645401.65)	926.38 (231.63 to 1899.48)
**2017**	6445379.44 (1840759.74 to 13108407.19)	705.76 (177.01 to 1470.75)	2172119.97 (610537.53 to 4493855.97)	487.16 (121.79 to 1033.09)	4273259.47 (1208039.95 to 8626149.89)	922.12 (227.46 to 1892.67)
**2018**	6420069.49 (1814144.08 to 13051160.65)	702.78 (175.23 to 1462.87)	2170880.15 (607221.81 to 4487025.72)	486.32 (120.87 to 1032.78)	4249189.34 (1195748.44 to 8575879.90)	917.42 (225.52 to 1881.90)
**2019**	6403434.72 (1800490.17 to 13022538.04)	701.07 (173.80 to 1461.56)	2169402.84 (605330.19 to 4479584.44)	485.67 (120.78 to 1029.55)	4234031.88 (1192380.07 to 8547342.37)	915.00 (224.49 to 1881.06)
**2020**	6365442.66 (1801710.91 to 12953725.47)	697.32 (173.27 to 1455.06)	2160360.81 (608609.46 to 4453954.89)	483.68 (120.34 to 1027.31)	4205081.85 (1195083.96 to 8480623.28)	909.78 (223.35 to 1874.43)
**2021**	6345063.09 (1796988.70 to 12906373.08)	696.22 (173.05 to 1449.93)	2156683.04 (610227.14 to 4441134.25)	483.36 (120.11 to 1021.87)	4188380.05 (1191901.20 to 8451079.17)	908.18 (223.50 to 1866.52)
**Percentage Change, 1990-2021**	0.08% (0.04% to 0.14%)	0.00% (-0.02% to 0.02%)	0.09% (0.06% to 0.17%)	0.01% (-0.01% to 0.03%)	0.07% (0.04% to 0.13%)	0.01% (-0.02% to 0.03%)
**Statistical Analysis**	Spearman's ρ = 0.870 p-value < 0.001	Spearman's ρ = 0.658 p-value < 0.001	Spearman's ρ = 0.974 p-value < 0.001	Spearman's ρ = 0.924 p-value < 0.001	Spearman's ρ = 0.784 p-value < 0.001	Spearman's ρ = 0.625 p-value < 0.001
			Wilcoxon rank sum test between sex (count): p-value < 0.001<br>Wilcoxon rank sum test between sex (rate): p-value < 0.001			

Table S2e: Counts and age-standardized prevalence rates of migraine from 1990 to 2021, by sex

	Both		Male		Female	
	Absolute number	Age-standardized rate, per 100 000 people	Absolute number	Age-standardized rate, per 100 000 people	Absolute number	Age-standardized rate, per 100 000 people
**1990**	134693058.36 (116329423.36 to 155047785.69)	15984.72 (13736.58 to 18412.19)	44475919.65 (37797958.81 to 51602008.57)	10821.46 (9209.97 to 12554.12)	90217138.71 (78296922.34 to 103850256.71)	21021.72 (18154.62 to 24131.73)
**1991**	135454821.98 (117158297.04 to 155874976.41)	15990.38 (13742.59 to 18405.92)	44723463.71 (38026589.20 to 51819647.04)	10817.61 (9209.16 to 12542.04)	90731358.27 (78831111.06 to 104259524.42)	21043.97 (18169.20 to 24152.10)
**1992**	136229804.86 (118019318.31 to 156723412.16)	15998.12 (13758.18 to 18404.25)	44969275.19 (38263984.93 to 52040365.35)	10815.47 (9207.09 to 12535.96)	91260529.67 (79366253.76 to 104729087.83)	21066.97 (18192.89 to 24168.92)
**1993**	136953444.93 (118824914.56 to 157514925.11)	16003.67 (13774.34 to 18402.27)	45190747.88 (38479801.75 to 52227526.18)	10812.44 (9204.05 to 12524.31)	91762697.05 (79895488.06 to 105188526.18)	21083.74 (18223.40 to 24174.35)
**1994**	137541341.15 (119370472.32 to 158161124.30)	16007.56 (13780.95 to 18398.41)	45364294.51 (38692294.52 to 52358815.71)	10809.96 (9203.70 to 12523.35)	92177046.64 (80385205.16 to 105581916.48)	21094.36 (18242.47 to 24162.57)
**1995**	138074253.23 (119844745.85 to 158749773.36)	16009.18 (13783.80 to 18393.28)	45525453.73 (38888768.11 to 52474116.83)	10808.87 (9207.97 to 12526.70)	92548799.50 (80840511.51 to 105900039.55)	21097.89 (18268.98 to 24141.52)
**1996**	138753485.29 (120682698.42 to 159362391.32)	16026.96 (13814.92 to 18402.17)	45732033.32 (39085395.64 to 52570601.12)	10817.60 (9229.08 to 12523.07)	93021451.97 (81370117.32 to 106344189.99)	21124.58 (18333.27 to 24144.44)
**1997**	139663335.86 (121693720.68 to 160109384.73)	16070.82 (13881.46 to 18436.72)	45999957.35 (39396944.44 to 52814454.40)	10839.70 (9265.44 to 12511.15)	93663378.51 (81989887.63 to 107012606.81)	21190.64 (18448.88 to 24199.03)
**1998**	140676468.07 (122811872.01 to 161052961.21)	16120.70 (13964.51 to 18477.23)	46300411.24 (39765701.01 to 53102959.45)	10864.63 (9306.01 to 12507.98)	94376056.83 (82781744.57 to 107684129.46)	21266.57 (18573.79 to 24263.36)
**1999**	141590922.17 (123862969.57 to 161891767.69)	16160.50 (14031.11 to 18508.52)	46576908.34 (40108490.11 to 53457531.83)	10884.73 (9341.39 to 12510.24)	95014013.83 (83647300.06 to 108305782.56)	21327.92 (18637.32 to 24314.79)
**2000**	142268703.92 (124767707.65 to 162517476.41)	16175.66 (14067.62 to 18502.40)	46792801.21 (40384959.73 to 53724001.35)	10893.22 (9366.61 to 12512.07)	95475902.72 (84325029.45 to 108770568.72)	21351.86 (18657.16 to 24338.70)

**2001**	142724591.54 (125156238.08 to 162972619.12)	16169.63 (14067.06 to 18502.12)	46950939.60 (40539665.72 to 53873181.64)	10891.31 (9368.83 to 12508.22)	95773651.94 (84566639.62 to 109144313.54)	21344.69 (18648.60 to 24320.87)
**2002**	143093687.91 (125461415.16 to 163432992.27)	16161.70 (14065.19 to 18498.27)	47077795.28 (40663472.38 to 53953375.13)	10888.00 (9364.72 to 12512.91)	96015892.63 (84823107.26 to 109455673.89)	21335.16 (18639.49 to 24308.83)
**2003**	143409787.87 (125725712.02 to 163845194.90)	16153.83 (14063.01 to 18491.05)	47182600.59 (40764604.75 to 54038985.90)	10884.17 (9364.90 to 12512.93)	96227187.28 (85052589.64 to 109739567.61)	21325.83 (18636.71 to 24297.88)
**2004**	143711254.73 (125972249.02 to 164188168.15)	16148.26 (14062.23 to 18484.49)	47284214.58 (40861267.63 to 54162629.48)	10881.87 (9367.46 to 12511.89)	96427040.15 (85242797.77 to 110021500.85)	21320.32 (18636.70 to 24291.54)
**2005**	144032298.34 (126315147.65 to 164497273.35)	16149.50 (14067.00 to 18485.62)	47389359.21 (40949372.97 to 54205903.71)	10883.81 (9373.45 to 12516.76)	96642939.13 (85346247.25 to 110331358.73)	21324.38 (18653.44 to 24295.57)
**2006**	144329266.75 (126617770.40 to 164815857.81)	16159.22 (14071.63 to 18498.85)	47489212.72 (41032049.40 to 54295778.47)	10892.36 (9380.61 to 12527.06)	96840054.03 (85528384.97 to 110561362.70)	21338.70 (18663.33 to 24314.76)
**2007**	144603974.27 (126881238.26 to 165106286.06)	16174.90 (14081.36 to 18518.33)	47587807.59 (41115886.64 to 54381327.39)	10906.19 (9391.90 to 12543.73)	97016166.67 (85692224.54 to 110748443.98)	21359.43 (18678.72 to 24340.56)
**2008**	144859611.27 (127119177.57 to 165363524.73)	16192.57 (14092.80 to 18539.73)	47677018.84 (41193867.35 to 54455610.87)	10921.02 (9403.70 to 12561.95)	97182592.43 (85851947.51 to 110926790.08)	21382.40 (18696.35 to 24368.46)
**2009**	145027292.36 (127313796.97 to 165508643.07)	16208.40 (14102.72 to 18558.60)	47733277.83 (41260858.39 to 54491252.37)	10933.51 (9413.09 to 12577.55)	97294014.53 (85954458.15 to 111034548.11)	21403.91 (18714.88 to 24394.26)
**2010**	145099664.90 (127402287.47 to 165523026.24)	16219.65 (14109.15 to 18571.75)	47746227.67 (41293183.25 to 54501078.88)	10940.40 (9417.43 to 12586.66)	97353437.23 (85988724.51 to 111076069.08)	21421.54 (18731.09 to 24415.19)
**2011**	145166843.31 (127481625.63 to 165743694.52)	16236.34 (14114.99 to 18605.63)	47767118.15 (41319224.82 to 54573049.38)	10953.25 (9426.19 to 12615.80)	97399725.16 (85998707.97 to 111253380.81)	21444.00 (18740.08 to 24466.81)
**2012**	145320744.63 (127537203.09 to 166049997.91)	16265.20 (14133.65 to 18656.32)	47848489.90 (41392286.51 to 54729593.50)	10980.10 (9444.76 to 12661.63)	97472254.73 (86024597.99 to 111369167.42)	21477.68 (18778.18 to 24535.95)
**2013**	145532308.77 (127561746.88 to 166232019.90)	16296.67 (14155.39 to 18712.14)	47969617.03 (41494901.94 to 54933187.32)	11011.65 (9475.27 to 12706.52)	97562691.74 (86026344.35 to 111483549.79)	21513.32 (18820.25 to 24611.08)

**2014**	145727494.72 (127686748.10 to 166488787.46)	16321.50 (14171.48 to 18747.16)	48097745.77 (41598950.78 to 55143719.49)	11038.89 (9499.24 to 12754.97)	97629748.95 (86015818.12 to 111579247.61)	21542.31 (18832.46 to 24676.71)
**2015**	145836081.53 (127787951.60 to 166646606.38)	16331.12 (14172.69 to 18762.00)	48193831.09 (41705280.59 to 55244136.24)	11052.71 (9507.80 to 12780.13)	97642250.44 (85938166.55 to 111613667.38)	21556.49 (18828.22 to 24702.70)
**2016**	145692127.25 (127398278.47 to 166589513.22)	16311.20 (14129.47 to 18748.05)	48209751.13 (41689638.26 to 55325439.26)	11045.88 (9495.77 to 12774.44)	97482376.11 (85728485.58 to 111504788.06)	21533.24 (18786.43 to 24705.48)
**2017**	145333825.14 (126762140.41 to 166597760.75)	16267.57 (14064.11 to 18713.89)	48170200.61 (41602811.76 to 55407143.56)	11025.41 (9469.78 to 12762.20)	97163624.54 (85158372.96 to 111331986.08)	21476.50 (18688.87 to 24650.85)
**2018**	144912123.85 (126188267.64 to 166574579.59)	16219.55 (13987.28 to 18676.25)	48105342.20 (41480790.99 to 55536986.39)	11001.05 (9437.68 to 12741.96)	96806781.65 (84613783.41 to 111113224.75)	21414.21 (18579.38 to 24616.96)
**2019**	144559444.37 (125753778.74 to 166535409.43)	16187.88 (13942.34 to 18665.64)	48042019.27 (41372568.19 to 55530835.70)	10983.37 (9413.41 to 12731.24)	96517425.10 (84158698.17 to 111109440.71)	21377.00 (18499.20 to 24585.20)
**2020**	143837136.25 (125122906.66 to 165669589.69)	16128.64 (13894.64 to 18590.34)	47871621.82 (41224119.13 to 55198048.76)	10951.84 (9378.11 to 12683.32)	95965514.43 (83809184.61 to 110397195.97)	21297.28 (18477.94 to 24499.88)
**2021**	143542170.14 (124859309.64 to 165307694.92)	16128.60 (13894.33 to 18592.33)	47804150.91 (41171341.16 to 55104465.37)	10952.93 (9377.99 to 12681.88)	95738019.23 (83608895.84 to 110149171.39)	21302.28 (18481.83 to 24506.50)
**Percentage Change, 1990-2021**	0.07% (0.04% to 0.09%)	0.01% (0.00% to 0.02%)	0.07% (0.04% to 0.11%)	0.01% (0.00% to 0.03%)	0.06% (0.03% to 0.09%)	0.01% (0.00% to 0.03%)
**Statistical Analysis**	Spearman's ρ = 0.850 p-value < 0.001	Spearman's ρ = 0.752 p-value < 0.001	Spearman's ρ = 0.964 p-value < 0.001	Spearman's ρ = 0.911 p-value < 0.001	Spearman's ρ = 0.775 p-value < 0.001	Spearman's ρ = 0.757 p-value < 0.001
				Wilcoxon rank sum test between sex (count): p-value < 0.001<br>Wilcoxon rank sum test between sex (rate): p-value < 0.001		

Table S2f: Counts and age-standardized DALY rates of migraine from 1990 to 2021, by sex

	Both		Male		Female	
	Absolute number	Age-standardized rate, per 100 000 people	Absolute number	Age-standardized rate, per 100 000 people	Absolute number	Age-standardized rate, per 100 000 people
**1990**	5203117.75 (1068484.67 to 11017419.82)	614.32 (118.25 to 1310.40)	1711779.75 (344885.26 to 3748341.36)	415.00 (81.03 to 914.80)	3491338.00 (724875.78 to 7380118.47)	808.18 (153.69 to 1729.52)
**1991**	5233374.45 (1077944.74 to 11082768.84)	614.50 (118.40 to 1308.54)	1722040.31 (347274.24 to 3764152.54)	414.90 (80.92 to 913.44)	3511334.14 (731314.07 to 7431239.74)	808.92 (153.49 to 1730.73)
**1992**	5263981.26 (1088785.02 to 11132458.29)	614.73 (118.60 to 1309.38)	1732196.82 (352037.45 to 3787264.37)	414.87 (81.27 to 914.14)	3531784.44 (736867.02 to 7449312.49)	809.64 (153.55 to 1727.36)
**1993**	5292235.82 (1094033.72 to 11189432.07)	614.89 (118.40 to 1309.05)	1740832.37 (353073.55 to 3811611.12)	414.69 (80.93 to 915.56)	3551403.45 (742893.32 to 7512211.32)	810.24 (153.86 to 1729.77)
**1994**	5315374.87 (1100951.05 to 11239399.52)	614.99 (118.33 to 1309.08)	1747760.44 (354617.16 to 3825588.69)	414.54 (80.73 to 915.64)	3567614.44 (748008.38 to 7531176.77)	810.62 (154.08 to 1726.90)
**1995**	5336306.52 (1107023.82 to 11258291.05)	614.95 (118.31 to 1309.37)	1754371.46 (357089.45 to 3834257.69)	414.47 (80.69 to 913.78)	3581935.06 (753229.17 to 7547686.37)	810.60 (154.25 to 1723.69)
**1996**	5365535.00 (1116517.33 to 11322799.04)	615.84 (118.62 to 1307.67)	1762634.57 (360719.20 to 3851453.97)	414.75 (80.98 to 917.56)	3602900.43 (758193.80 to 7603457.11)	812.11 (154.50 to 1732.33)
**1997**	5406705.39 (1133667.03 to 11411809.43)	618.05 (119.82 to 1311.56)	1773558.66 (363764.64 to 3852893.41)	415.60 (81.15 to 914.59)	3633146.72 (771242.72 to 7634124.76)	815.69 (156.51 to 1742.52)
**1998**	5453114.53 (1148182.48 to 11501960.43)	620.64 (120.59 to 1316.87)	1786030.05 (367675.65 to 3884157.50)	416.63 (81.39 to 917.05)	3667084.48 (780842.86 to 7691781.85)	819.89 (157.62 to 1752.11)
**1999**	5494987.66 (1164931.43 to 11610753.26)	622.72 (121.72 to 1321.54)	1797319.64 (373454.38 to 3906078.72)	417.40 (82.22 to 918.90)	3697668.02 (791459.38 to 7727744.66)	823.34 (159.14 to 1757.73)
**2000**	5524955.74 (1174414.40 to 11675328.08)	623.54 (122.17 to 1322.59)	1806344.76 (377725.34 to 3918319.01)	417.75 (82.31 to 919.04)	3718610.97 (796647.14 to 7779702.70)	824.73 (159.99 to 1764.47)

**2001**	5544573.28 (1180698.77 to 11728066.50)	623.37 (122.05 to 1324.41)	1813190.01 (378493.90 to 3935854.69)	417.72 (81.93 to 918.56)	3731383.27 (802204.88 to 7791352.24)	824.54 (160.15 to 1761.62)
**2002**	5561383.95 (1192464.25 to 11749879.26)	623.17 (122.14 to 1322.27)	1819068.21 (382806.64 to 3936553.58)	417.72 (82.29 to 919.28)	3742315.74 (809501.86 to 7813179.49)	824.25 (159.77 to 1763.01)
**2003**	5577012.31 (1198508.08 to 11795215.96)	623.12 (122.29 to 1322.89)	1824509.47 (383418.02 to 3946781.49)	417.79 (81.95 to 917.03)	3752502.84 (816059.89 to 7845245.08)	824.18 (160.66 to 1765.90)
**2004**	5591156.33 (1208181.31 to 11833329.19)	623.01 (122.20 to 1323.42)	1829166.26 (386324.79 to 3970323.61)	417.74 (81.97 to 922.29)	3761990.07 (822841.56 to 7866300.04)	824.16 (160.47 to 1768.01)
**2005**	5606695.59 (1210095.46 to 11825853.15)	623.22 (122.23 to 1322.20)	1834818.82 (387915.94 to 3971207.74)	418.05 (81.80 to 920.37)	3771876.77 (824454.09 to 7879340.67)	824.40 (160.75 to 1764.01)
**2006**	5619842.43 (1221604.02 to 11880829.49)	623.61 (122.55 to 1326.86)	1839024.40 (393503.79 to 3984221.73)	418.33 (82.47 to 923.05)	3780818.03 (830879.20 to 7874694.27)	825.03 (160.78 to 1767.68)
**2007**	5632229.74 (1225103.69 to 11898608.54)	624.17 (121.77 to 1328.40)	1843342.25 (393320.33 to 3987559.83)	418.81 (82.01 to 921.58)	3788887.49 (832561.42 to 7889809.16)	825.82 (159.71 to 1767.80)
**2008**	5644670.43 (1233868.02 to 11923014.03)	624.96 (122.19 to 1329.61)	1848296.75 (395672.45 to 3991371.99)	419.60 (82.12 to 925.73)	3796373.67 (838521.79 to 7904526.27)	826.70 (160.52 to 1771.70)
**2009**	5653913.65 (1236833.49 to 11928776.04)	625.63 (122.19 to 1329.89)	1851203.56 (398041.12 to 4001917.29)	420.09 (82.28 to 926.08)	3802710.09 (840763.22 to 7920029.49)	827.66 (160.32 to 1772.49)
**2010**	5660086.52 (1243359.80 to 11952827.00)	626.19 (122.11 to 1329.68)	1853370.66 (400963.60 to 3995753.88)	420.52 (82.37 to 924.78)	3806715.86 (842396.20 to 7923004.05)	828.42 (160.13 to 1774.44)
**2011**	5664877.56 (1244740.13 to 11934887.15)	626.82 (122.09 to 1332.68)	1855403.71 (400418.82 to 4006099.22)	421.12 (82.01 to 925.17)	3809473.85 (844321.32 to 7920138.93)	829.15 (160.48 to 1776.46)
**2012**	5671134.02 (1250178.61 to 11932296.73)	627.73 (121.80 to 1336.02)	1858151.50 (400367.22 to 4025156.06)	421.89 (81.72 to 927.84)	3812982.52 (849811.39 to 7918470.77)	830.33 (160.23 to 1777.74)
**2013**	5680870.38 (1248504.92 to 11936599.68)	628.93 (121.01 to 1336.61)	1863674.77 (399686.02 to 4025412.98)	423.13 (81.24 to 931.87)	3817195.62 (849054.65 to 7920472.40)	831.65 (159.15 to 1783.60)

**2014**	5689836.79 (1260264.71 to 11951089.62)	629.80 (121.41 to 1339.86)	1868992.35 (406345.19 to 4029482.04)	424.08 (81.77 to 935.61)	3820844.44 (853919.52 to 7921383.80)	832.71 (159.19 to 1786.48)
**2015**	5697078.78 (1263604.05 to 11942422.27)	630.31 (121.13 to 1342.51)	1873947.53 (405630.33 to 4044393.70)	424.77 (81.48 to 935.51)	3823131.26 (857973.72 to 7924684.57)	833.38 (159.38 to 1786.84)
**2016**	5689607.96 (1260881.92 to 11907561.56)	629.19 (120.51 to 1335.86)	1875512.94 (407233.93 to 4045363.17)	424.61 (81.35 to 937.56)	3814095.02 (853647.99 to 7894115.62)	831.68 (158.25 to 1778.37)
**2017**	5670376.40 (1250075.91 to 11886840.44)	626.92 (118.91 to 1332.80)	1874691.48 (406543.74 to 4048124.77)	423.96 (81.25 to 937.67)	3795684.92 (843745.82 to 7865797.35)	828.19 (155.39 to 1774.68)
**2018**	5647865.76 (1236241.30 to 11877457.79)	624.38 (117.09 to 1328.72)	1872792.92 (404996.54 to 4038396.08)	423.13 (80.49 to 935.49)	3775072.84 (830705.04 to 7849776.55)	824.31 (152.76 to 1767.47)
**2019**	5632053.76 (1231907.06 to 11846822.79)	622.86 (116.12 to 1326.36)	1870739.64 (404956.89 to 4011935.59)	422.49 (80.06 to 936.11)	3761314.12 (826238.06 to 7851427.46)	822.25 (151.29 to 1754.01)
**2020**	5595986.89 (1233370.21 to 11767494.34)	619.40 (116.04 to 1318.92)	1862495.26 (405053.57 to 3981008.41)	420.77 (79.54 to 930.32)	3733491.63 (827993.47 to 7787135.18)	817.33 (151.33 to 1746.36)
**2021**	5577086.08 (1234081.57 to 11719998.01)	618.43 (116.07 to 1315.08)	1859028.36 (409180.70 to 3972660.88)	420.49 (81.01 to 930.36)	3718057.71 (824559.40 to 7770294.82)	815.91 (151.00 to 1739.13)
**Percentage Change, 1990-2021**	0.07% (0.04% to 0.15%)	0.01% (-0.02% to 0.02%)	0.09% (0.05% to 0.18%)	0.01% (-0.01% to 0.03%)	0.06% (0.03% to 0.13%)	0.01% (-0.02% to 0.03%)
**Statistical Analysis**	Spearman's ρ = 0.848 p-value < 0.001	Spearman's ρ = 0.699 p-value < 0.001	Spearman's ρ = 0.970 p-value < 0.001	Spearman's ρ = 0.924 p-value < 0.001	Spearman's ρ = 0.761 p-value < 0.001	Spearman's ρ = 0.661 p-value < 0.001
			Wilcoxon rank sum test between sex (count): p-value < 0.001 Wilcoxon rank sum test between sex (rate): p-value < 0.001			

Table S2g: Counts and age-standardized incidence rates of migraine from 1990 to 2021, by sex

	Both		Male		Female	
	Absolute number	Age-standardized rate, per 100 000 people	Absolute number	Age-standardized rate, per 100 000 people	Absolute number	Age-standardized rate, per 100 000 people
**1990**	9470368.50 (8249961.74 to 10780895.79)	1232.64 (1072.47 to 1401.37)	3319452.75 (2856376.09 to 3825508.22)	864.03 (744.61 to 990.51)	6150915.75 (5407171.65 to 6949414.63)	1607.95 (1401.76 to 1823.58)
**1991**	9491405.84 (8270298.28 to 10814722.55)	1231.08 (1071.23 to 1399.35)	3329194.52 (2865043.37 to 3835585.91)	863.29 (743.87 to 989.21)	6162211.32 (5414231.96 to 6965829.65)	1605.66 (1399.97 to 1820.41)
**1992**	9510308.11 (8287553.28 to 10847963.16)	1229.78 (1070.04 to 1397.56)	3338127.64 (2874645.52 to 3847408.18)	862.71 (743.17 to 989.02)	6172180.47 (5419908.75 to 6985149.00)	1603.70 (1398.94 to 1818.09)
**1993**	9526826.69 (8299318.79 to 10880572.87)	1228.66 (1069.08 to 1396.10)	3345441.73 (2882513.22 to 3853618.00)	862.18 (742.72 to 989.04)	6181384.95 (5424209.93 to 7005136.74)	1601.99 (1398.15 to 1816.27)
**1994**	9533769.71 (8303529.22 to 10896221.26)	1227.87 (1068.47 to 1395.08)	3348472.22 (2886083.08 to 3855056.72)	861.83 (742.43 to 989.25)	6185297.49 (5423550.07 to 7021339.14)	1600.63 (1397.75 to 1814.99)
**1995**	9535441.95 (8303909.68 to 10899870.56)	1227.39 (1068.23 to 1394.73)	3349059.03 (2889066.86 to 3854454.84)	861.66 (742.28 to 989.20)	6186382.91 (5427358.73 to 7033811.22)	1599.70 (1397.80 to 1814.20)
**1996**	9538229.88 (8305844.34 to 10889781.46)	1227.81 (1068.18 to 1395.40)	3350287.75 (2884577.94 to 3852763.92)	862.14 (743.34 to 988.88)	6187942.13 (5427911.51 to 7030738.35)	1600.00 (1397.73 to 1815.46)
**1997**	9543208.70 (8317306.73 to 10884597.12)	1229.52 (1069.44 to 1397.24)	3352648.04 (2883033.58 to 3851773.26)	863.55 (745.23 to 989.03)	6190560.67 (5429413.92 to 7028533.24)	1602.05 (1399.28 to 1817.02)
**1998**	9549013.50 (8330050.74 to 10879656.17)	1231.88 (1071.33 to 1400.95)	3355318.14 (2885070.11 to 3851518.13)	865.38 (747.42 to 989.66)	6193695.36 (5431565.88 to 7023277.60)	1605.06 (1401.87 to 1820.02)
**1999**	9548774.89 (8335370.56 to 10859080.89)	1234.24 (1073.14 to 1403.85)	3355640.20 (2886040.22 to 3848746.72)	867.15 (749.49 to 991.26)	6193134.69 (5432363.71 to 7019561.94)	1608.16 (1406.55 to 1822.99)
**2000**	9538344.46 (8331088.35 to 10846277.92)	1236.04 (1075.27 to 1405.01)	3351851.11 (2883298.29 to 3843362.23)	868.45 (751.14 to 992.78)	6186493.35 (5432783.00 to 7015860.72)	1610.62 (1410.35 to 1824.55)

**2001**	9518771.50 (8317451.89 to 10819552.76)	1237.36 (1076.61 to 1406.18)	3344391.05 (2878412.67 to 3833474.97)	869.29 (751.66 to 993.51)	6174380.45 (5421345.69 to 7002931.64)	1612.59 (1412.04 to 1826.25)
**2002**	9492927.92 (8295832.93 to 10782964.94)	1238.73 (1077.93 to 1407.13)	3334226.12 (2872067.55 to 3822277.37)	870.02 (752.13 to 994.21)	6158701.80 (5407442.01 to 6980135.73)	1614.79 (1414.00 to 1828.24)
**2003**	9463368.22 (8270173.27 to 10741593.97)	1240.17 (1079.21 to 1408.00)	3322348.82 (2863354.13 to 3809880.36)	870.71 (752.62 to 995.11)	6141019.40 (5392035.69 to 6952731.56)	1617.18 (1416.03 to 1830.24)
**2004**	9432549.81 (8237437.39 to 10699481.41)	1241.65 (1080.55 to 1408.76)	3310473.15 (2853899.09 to 3794665.11)	871.42 (753.15 to 996.49)	6122076.66 (5375659.67 to 6926426.83)	1619.65 (1417.95 to 1832.26)
**2005**	9402086.41 (8204840.82 to 10667699.59)	1243.32 (1081.98 to 1409.73)	3299049.69 (2844048.38 to 3778671.23)	872.31 (753.51 to 997.78)	6103036.72 (5359436.82 to 6907214.55)	1622.32 (1419.85 to 1834.07)
**2006**	9370677.74 (8167667.00 to 10635994.73)	1245.18 (1083.57 to 1411.62)	3287909.92 (2834547.70 to 3764165.29)	873.44 (754.43 to 999.09)	6082767.82 (5345309.58 to 6886875.27)	1625.10 (1422.10 to 1836.59)
**2007**	9338479.47 (8133758.31 to 10603638.94)	1247.06 (1085.03 to 1413.66)	3276973.02 (2826438.44 to 3752651.49)	874.63 (755.44 to 1000.48)	6061506.45 (5327605.27 to 6867714.94)	1627.81 (1424.11 to 1839.19)
**2008**	9307879.56 (8107383.43 to 10572453.54)	1248.76 (1086.28 to 1415.38)	3266739.73 (2814969.80 to 3741866.36)	875.68 (756.38 to 1001.69)	6041139.83 (5307014.74 to 6850268.91)	1630.27 (1425.85 to 1841.60)
**2009**	9276848.35 (8079263.77 to 10548315.42)	1250.22 (1087.29 to 1416.78)	3256382.17 (2804782.36 to 3728379.29)	876.49 (757.12 to 1002.60)	6020466.19 (5288360.02 to 6831752.32)	1632.47 (1427.32 to 1843.80)
**2010**	9243982.12 (8054646.10 to 10522024.10)	1251.29 (1087.96 to 1417.83)	3245010.71 (2795792.57 to 3710746.40)	876.89 (757.54 to 1002.99)	5998971.41 (5272396.00 to 6810661.70)	1634.29 (1428.45 to 1845.79)
**2011**	9214182.19 (8029487.78 to 10490221.03)	1252.52 (1089.16 to 1419.47)	3235578.16 (2789126.71 to 3692335.40)	877.62 (758.27 to 1004.29)	5978604.03 (5251985.36 to 6779515.42)	1636.09 (1426.84 to 1847.66)
**2012**	9191990.39 (8010276.16 to 10454242.54)	1254.23 (1090.01 to 1422.84)	3230950.42 (2787062.96 to 3690829.14)	879.14 (759.61 to 1005.72)	5961039.97 (5234049.10 to 6749991.95)	1638.10 (1428.42 to 1850.37)
**2013**	9177562.99 (7996314.65 to 10426753.07)	1255.81 (1090.67 to 1425.88)	3230505.26 (2787390.11 to 3692445.71)	880.82 (760.19 to 1007.80)	5947057.73 (5218637.27 to 6729112.18)	1639.82 (1430.46 to 1853.16)

**2014**	9169803.63 (7986705.72 to 10417820.23)	1256.91 (1091.04 to 1427.83)	3232884.35 (2791784.35 to 3699135.04)	882.15 (759.71 to 1008.75)	5936919.28 (5204602.98 to 6713409.84)	1641.06 (1429.76 to 1855.97)
**2015**	9163344.13 (7989947.07 to 10416503.34)	1257.07 (1090.62 to 1428.81)	3235296.40 (2799047.36 to 3708172.65)	882.59 (759.50 to 1010.00)	5928047.72 (5192997.14 to 6701974.56)	1641.47 (1427.66 to 1857.84)
**2016**	9154177.57 (7984888.96 to 10418588.90)	1256.06 (1090.00 to 1430.57)	3236087.41 (2792519.10 to 3706602.19)	882.04 (759.52 to 1011.62)	5918090.16 (5185753.21 to 6697946.75)	1640.54 (1427.78 to 1858.86)
**2017**	9143578.16 (7973139.77 to 10404473.18)	1254.28 (1088.60 to 1429.22)	3236573.42 (2787881.25 to 3710852.67)	881.04 (758.07 to 1010.86)	5907004.74 (5176150.24 to 6682790.71)	1638.51 (1427.57 to 1858.74)
**2018**	9130557.19 (7956403.73 to 10371478.17)	1252.33 (1087.01 to 1426.10)	3235911.31 (2784218.76 to 3710043.38)	879.92 (756.24 to 1008.52)	5894645.87 (5165586.01 to 6661900.46)	1636.19 (1426.84 to 1855.51)
**2019**	9114415.15 (7926887.89 to 10345946.67)	1250.92 (1085.91 to 1423.63)	3233400.42 (2783019.83 to 3707505.50)	879.10 (753.89 to 1007.84)	5881014.74 (5142109.98 to 6643642.75)	1634.58 (1426.64 to 1854.08)
**2020**	9081416.37 (7902332.48 to 10316991.08)	1248.20 (1084.02 to 1419.52)	3223550.59 (2774758.31 to 3694479.18)	877.22 (752.33 to 1006.32)	5857865.78 (5116795.08 to 6621261.19)	1631.29 (1422.91 to 1851.19)
**2021**	9057610.76 (7881493.51 to 10303044.20)	1248.05 (1084.00 to 1419.21)	3217801.56 (2771062.45 to 3686607.65)	877.41 (752.49 to 1006.54)	5839809.20 (5101893.46 to 6597893.91)	1631.07 (1422.68 to 1851.11)
**Percentage Change, 1990-2021**	-0.04% (-0.07% to -0.02%)	0.01% (0.01% to 0.02%)	-0.03% (-0.06% to -0.01%)	0.02% (0.01% to 0.03%)	-0.05% (-0.08% to -0.03%)	0.01% (0.01% to 0.02%)
**Statistical Analysis**	Spearman's ρ = -0.903 p-value < 0.001	Spearman's ρ = 0.877 p-value < 0.001	Spearman's ρ = -0.865 p-value < 0.001	Spearman's ρ = 0.923 p-value < 0.001	Spearman's ρ = -0.908 p-value < 0.001	Spearman's ρ = 0.889 p-value < 0.001
	Wilcoxon rank sum test between sex (count): p-value < 0.001 Wilcoxon rank sum test between sex (rate): p-value < 0.001					

Table S2h: Counts and age-standardized YLD rates of migraine from 1990 to 2021, by sex

	Both		Male		Female	
	Absolute number	Age-standardized rate, per 100 000 people	Absolute number	Age-standardized rate, per 100 000 people	Absolute number	Age-standardized rate, per 100 000 people
**1990**	5203117.75 (1068484.67 to 11017419.82)	614.32 (118.25 to 1310.40)	1711779.75 (344885.26 to 3748341.36)	415.00 (81.03 to 914.80)	3491338.00 (724875.78 to 7380118.47)	808.18 (153.69 to 1729.52)
**1991**	5233374.45 (1077944.74 to 11082768.84)	614.50 (118.40 to 1308.54)	1722040.31 (347274.24 to 3764152.54)	414.90 (80.92 to 913.44)	3511334.14 (731314.07 to 7431239.74)	808.92 (153.49 to 1730.73)
**1992**	5263981.26 (1088785.02 to 11132458.29)	614.73 (118.60 to 1309.38)	1732196.82 (352037.45 to 3787264.37)	414.87 (81.27 to 914.14)	3531784.44 (736867.02 to 7449312.49)	809.64 (153.55 to 1727.36)
**1993**	5292235.82 (1094033.72 to 11189432.07)	614.89 (118.40 to 1309.05)	1740832.37 (353073.55 to 3811611.12)	414.69 (80.93 to 915.56)	3551403.45 (742893.32 to 7512211.32)	810.24 (153.86 to 1729.77)
**1994**	5315374.87 (1100951.05 to 11239399.52)	614.99 (118.33 to 1309.08)	1747760.44 (354617.16 to 3825588.69)	414.54 (80.73 to 915.64)	3567614.44 (748008.38 to 7531176.77)	810.62 (154.08 to 1726.90)
**1995**	5336306.52 (1107023.82 to 11258291.05)	614.95 (118.31 to 1309.37)	1754371.46 (357089.45 to 3834257.69)	414.47 (80.69 to 913.78)	3581935.06 (753229.17 to 7547686.37)	810.60 (154.25 to 1723.69)
**1996**	5365535.00 (1116517.33 to 11322799.04)	615.84 (118.62 to 1307.67)	1762634.57 (360719.20 to 3851453.97)	414.75 (80.98 to 917.56)	3602900.43 (758193.80 to 7603457.11)	812.11 (154.50 to 1732.33)
**1997**	5406705.39 (1133667.03 to 11411809.43)	618.05 (119.82 to 1311.56)	1773558.66 (363764.64 to 3852893.41)	415.60 (81.15 to 914.59)	3633146.72 (771242.72 to 7634124.76)	815.69 (156.51 to 1742.52)
**1998**	5453114.53 (1148182.48 to 11501960.43)	620.64 (120.59 to 1316.87)	1786030.05 (367675.65 to 3884157.50)	416.63 (81.39 to 917.05)	3667084.48 (780842.86 to 7691781.85)	819.89 (157.62 to 1752.11)
**1999**	5494987.66 (1164931.43 to 11610753.26)	622.72 (121.72 to 1321.54)	1797319.64 (373454.38 to 3906078.72)	417.40 (82.22 to 918.90)	3697668.02 (791459.38 to 7727744.66)	823.34 (159.14 to 1757.73)
**2000**	5524955.74 (1174414.40 to 11675328.08)	623.54 (122.17 to 1322.59)	1806344.76 (377725.34 to 3918319.01)	417.75 (82.31 to 919.04)	3718610.97 (796647.14 to 7779702.70)	824.73 (159.99 to 1764.47)

**2001**	5544573.28 (1180698.77 to 11728066.50)	623.37 (122.05 to 1324.41)	1813190.01 (378493.90 to 3935854.69)	417.72 (81.93 to 918.56)	3731383.27 (802204.88 to 7791352.24)	824.54 (160.15 to 1761.62)
**2002**	5561383.95 (1192464.25 to 11749879.26)	623.17 (122.14 to 1322.27)	1819068.21 (382806.64 to 3936553.58)	417.72 (82.29 to 919.28)	3742315.74 (809501.86 to 7813179.49)	824.25 (159.77 to 1763.01)
**2003**	5577012.31 (1198508.08 to 11795215.96)	623.12 (122.29 to 1322.89)	1824509.47 (383418.02 to 3946781.49)	417.79 (81.95 to 917.03)	3752502.84 (816059.89 to 7845245.08)	824.18 (160.66 to 1765.90)
**2004**	5591156.33 (1208181.31 to 11833329.19)	623.01 (122.20 to 1323.42)	1829166.26 (386324.79 to 3970323.61)	417.74 (81.97 to 922.29)	3761990.07 (822841.56 to 7866300.04)	824.16 (160.47 to 1768.01)
**2005**	5606695.59 (1210095.46 to 11825853.15)	623.22 (122.23 to 1322.20)	1834818.82 (387915.94 to 3971207.74)	418.05 (81.80 to 920.37)	3771876.77 (824454.09 to 7879340.67)	824.40 (160.75 to 1764.01)
**2006**	5619842.43 (1221604.02 to 11880829.49)	623.61 (122.55 to 1326.86)	1839024.40 (393503.79 to 3984221.73)	418.33 (82.47 to 923.05)	3780818.03 (830879.20 to 7874694.27)	825.03 (160.78 to 1767.68)
**2007**	5632229.74 (1225103.69 to 11898608.54)	624.17 (121.77 to 1328.40)	1843342.25 (393320.33 to 3987559.83)	418.81 (82.01 to 921.58)	3788887.49 (832561.42 to 7889809.16)	825.82 (159.71 to 1767.80)
**2008**	5644670.43 (1233868.02 to 11923014.03)	624.96 (122.19 to 1329.61)	1848296.75 (395672.45 to 3991371.99)	419.60 (82.12 to 925.73)	3796373.67 (838521.79 to 7904526.27)	826.70 (160.52 to 1771.70)
**2009**	5653913.65 (1236833.49 to 11928776.04)	625.63 (122.19 to 1329.89)	1851203.56 (398041.12 to 4001917.29)	420.09 (82.28 to 926.08)	3802710.09 (840763.22 to 7920029.49)	827.66 (160.32 to 1772.49)
**2010**	5660086.52 (1243359.80 to 11952827.00)	626.19 (122.11 to 1329.68)	1853370.66 (400963.60 to 3995753.88)	420.52 (82.37 to 924.78)	3806715.86 (842396.20 to 7923004.05)	828.42 (160.13 to 1774.44)
**2011**	5664877.56 (1244740.13 to 11934887.15)	626.82 (122.09 to 1332.68)	1855403.71 (400418.82 to 4006099.22)	421.12 (82.01 to 925.17)	3809473.85 (844321.32 to 7920138.93)	829.15 (160.48 to 1776.46)
**2012**	5671134.02 (1250178.61 to 11932296.73)	627.73 (121.80 to 1336.02)	1858151.50 (400367.22 to 4025156.06)	421.89 (81.72 to 927.84)	3812982.52 (849811.39 to 7918470.77)	830.33 (160.23 to 1777.74)
**2013**	5680870.38 (1248504.92 to 11936599.68)	628.93 (121.01 to 1336.61)	1863674.77 (399686.02 to 4025412.98)	423.13 (81.24 to 931.87)	3817195.62 (849054.65 to 7920472.40)	831.65 (159.15 to 1783.60)

**2014**	5689836.79 (1260264.71 to 11951089.62)	629.80 (121.41 to 1339.86)	1868992.35 (406345.19 to 4029482.04)	424.08 (81.77 to 935.61)	3820844.44 (853919.52 to 7921383.80)	832.71 (159.19 to 1786.48)
**2015**	5697078.78 (1263604.05 to 11942422.27)	630.31 (121.13 to 1342.51)	1873947.53 (405630.33 to 4044393.70)	424.77 (81.48 to 935.51)	3823131.26 (857973.72 to 7924684.57)	833.38 (159.38 to 1786.84)
**2016**	5689607.96 (1260881.92 to 11907561.56)	629.19 (120.51 to 1335.86)	1875512.94 (407233.93 to 4045363.17)	424.61 (81.35 to 937.56)	3814095.02 (853647.99 to 7894115.62)	831.68 (158.25 to 1778.37)
**2017**	5670376.40 (1250075.91 to 11886840.44)	626.92 (118.91 to 1332.80)	1874691.48 (406543.74 to 4048124.77)	423.96 (81.25 to 937.67)	3795684.92 (843745.82 to 7865797.35)	828.19 (155.39 to 1774.68)
**2018**	5647865.76 (1236241.30 to 11877457.79)	624.38 (117.09 to 1328.72)	1872792.92 (404996.54 to 4038396.08)	423.13 (80.49 to 935.49)	3775072.84 (830705.04 to 7849776.55)	824.31 (152.76 to 1767.47)
**2019**	5632053.76 (1231907.06 to 11846822.79)	622.86 (116.12 to 1326.36)	1870739.64 (404956.89 to 4011935.59)	422.49 (80.06 to 936.11)	3761314.12 (826238.06 to 7851427.46)	822.25 (151.29 to 1754.01)
**2020**	5595986.89 (1233370.21 to 11767494.34)	619.40 (116.04 to 1318.92)	1862495.26 (405053.57 to 3981008.41)	420.77 (79.54 to 930.32)	3733491.63 (827993.47 to 7787135.18)	817.33 (151.33 to 1746.36)
**2021**	5577086.08 (1234081.57 to 11719998.01)	618.43 (116.07 to 1315.08)	1859028.36 (409180.70 to 3972660.88)	420.49 (81.01 to 930.36)	3718057.71 (824559.40 to 7770294.82)	815.91 (151.00 to 1739.13)
**Percentage Change, 1990-2021**	0.07% (0.04% to 0.15%)	0.01% (-0.02% to 0.02%)	0.09% (0.05% to 0.18%)	0.01% (-0.01% to 0.03%)	0.06% (0.03% to 0.13%)	0.01% (-0.02% to 0.03%)
**Statistical Analysis**	Spearman's ρ = 0.848 p-value < 0.001	Spearman's ρ = 0.699 p-value < 0.001	Spearman's ρ = 0.970 p-value < 0.001	Spearman's ρ = 0.924 p-value < 0.001	Spearman's ρ = 0.761 p-value < 0.001	Spearman's ρ = 0.661 p-value < 0.001
	Wilcoxon rank sum test between sex (count): p-value < 0.001<br>Wilcoxon rank sum test between sex (rate): p-value < 0.001					

Table S2i: Counts and age-standardized prevalence rates of tension-type headache from 1990 to 2021, by sex

	Both		Male		Female	
	Absolute number	Age-standardized rate, per 100 000 people	Absolute number	Age-standardized rate, per 100 000 people	Absolute number	Age-standardized rate, per 100 000 people
**1990**	263085772.75 (234474303.61 to 296486651.56)	31057.91 (27533.49 to 35153.24)	123598803.54 (109388608.31 to 140161902.53)	30215.98 (26655.86 to 34300.99)	139486969.21 (124412718.79 to 156684931.45)	31814.06 (28237.04 to 35902.34)
**1991**	264166774.59 (235234308.73 to 297433102.56)	31008.54 (27490.83 to 35105.20)	124156939.47 (109885411.20 to 140753677.15)	30163.36 (26587.55 to 34267.13)	140009835.12 (124905828.38 to 157048739.85)	31769.41 (28204.82 to 35860.26)
**1992**	265263023.76 (236105279.29 to 298546305.23)	30961.51 (27435.48 to 35057.36)	124710078.82 (110391244.24 to 141399372.78)	30114.67 (26519.74 to 34224.64)	140552944.94 (125464941.70 to 157529983.66)	31725.62 (28169.02 to 35835.54)
**1993**	266315573.43 (237003222.82 to 299639558.13)	30919.30 (27385.31 to 35012.88)	125222954.32 (110873199.14 to 141960292.45)	30072.14 (26459.35 to 34177.71)	141092619.11 (126041715.55 to 158036728.50)	31685.10 (28133.84 to 35811.65)
**1994**	267175502.85 (237759034.00 to 300514023.99)	30883.36 (27342.44 to 34973.72)	125617856.01 (111359619.08 to 142380605.48)	30036.80 (26408.53 to 34138.75)	141557646.84 (126568237.52 to 158468224.88)	31649.69 (28105.61 to 35791.95)
**1995**	267972086.57 (238325131.04 to 301205431.12)	30853.69 (27305.70 to 34941.96)	125971949.42 (111735989.38 to 142748306.06)	30008.68 (26389.06 to 34107.82)	142000137.15 (126933404.17 to 158816464.28)	31619.63 (28082.24 to 35749.88)
**1996**	268721600.18 (239319557.07 to 301688583.70)	30819.37 (27289.00 to 34863.72)	126326743.82 (112141675.74 to 142937194.64)	29980.34 (26369.50 to 34017.60)	142394856.36 (127265143.81 to 158956388.65)	31581.13 (28111.39 to 35661.79)
**1997**	269348893.14 (240261991.51 to 301807651.46)	30772.04 (27261.76 to 34769.16)	126659583.36 (112398015.94 to 143005821.44)	29945.46 (26382.40 to 33914.12)	142689309.79 (127580226.47 to 159035457.58)	31524.03 (28066.77 to 35545.86)
**1998**	269982409.46 (241217843.18 to 301908227.90)	30718.95 (27239.01 to 34664.86)	127019718.92 (112704912.88 to 143104676.68)	29907.38 (26378.49 to 33806.17)	142962690.55 (127998388.38 to 159150658.32)	31458.91 (27975.00 to 35418.46)
**1999**	270604255.69 (241568962.85 to 301990662.99)	30669.30 (27192.90 to 34567.05)	127371550.13 (113111902.97 to 143206202.51)	29870.69 (26373.32 to 33741.74)	143232705.57 (128429717.49 to 159410666.37)	31399.18 (27958.07 to 35310.92)
**2000**	271294415.00 (242089871.31 to 302240865.32)	30634.94 (27204.82 to 34508.37)	127728752.24 (113508512.05 to 143362280.64)	29843.37 (26365.75 to 33683.79)	143565662.76 (128791356.76 to 159623002.55)	31360.10 (27949.68 to 35221.61)

**2001**	272139352.65 (242912072.98 to 303130827.33)	30620.93 (27188.91 to 34477.25)	128146099.40 (113992889.73 to 143750987.94)	29833.21 (26349.34 to 33673.52)	143993253.25 (129045493.45 to 160180481.14)	31344.09 (27930.57 to 35214.10)
**2002**	273038926.89 (243998976.88 to 304060732.73)	30620.26 (27184.85 to 34455.34)	128596640.33 (114303400.34 to 144196481.63)	29838.40 (26369.95 to 33651.66)	144442286.55 (129583851.68 to 160527105.10)	31339.10 (27915.36 to 35206.71)
**2003**	273887334.11 (244908628.10 to 304948051.20)	30623.70 (27194.58 to 34437.21)	129022531.84 (114571989.34 to 144612304.38)	29848.69 (26419.72 to 33635.03)	144864802.27 (130124956.41 to 161038475.82)	31336.83 (27927.11 to 35205.03)
**2004**	274684600.67 (245790881.14 to 305775035.34)	30624.17 (27209.79 to 34425.70)	129424012.10 (114959894.82 to 144978135.53)	29855.40 (26462.08 to 33616.24)	145260588.58 (130635476.69 to 161504654.47)	31332.19 (27936.62 to 35201.02)
**2005**	275352936.10 (246471917.48 to 306454738.63)	30615.95 (27221.51 to 34419.20)	129751161.63 (115395446.57 to 145264490.66)	29851.85 (26485.31 to 33604.40)	145601774.48 (130922240.04 to 161848714.21)	31320.46 (27941.59 to 35206.17)
**2006**	275812434.60 (246850907.70 to 306932869.40)	30600.99 (27201.86 to 34402.46)	129966196.56 (115712304.26 to 145479451.33)	29840.48 (26468.31 to 33586.08)	145846238.04 (131116860.76 to 162072321.70)	31302.98 (27920.62 to 35197.90)
**2007**	276157358.50 (247207134.07 to 307221717.75)	30586.44 (27181.49 to 34384.97)	130126353.73 (115922881.79 to 145635601.89)	29829.61 (26452.17 to 33568.51)	146031004.77 (131283681.26 to 162204125.45)	31285.64 (27900.50 to 35186.73)
**2008**	276424432.89 (247530898.41 to 307582712.67)	30570.87 (27161.97 to 34366.14)	130247456.90 (116065541.38 to 145715000.20)	29817.34 (26435.13 to 33549.62)	146176975.99 (131427905.76 to 162405972.89)	31267.54 (27879.95 to 35165.88)
**2009**	276599547.70 (247799971.68 to 307905777.97)	30555.88 (27142.84 to 34348.57)	130324738.93 (116056309.92 to 145659345.23)	29805.63 (26418.64 to 33531.81)	146274808.77 (131564920.56 to 162542308.47)	31250.16 (27860.09 to 35146.10)
**2010**	276707856.12 (247875065.89 to 308196729.87)	30543.51 (27126.23 to 34334.96)	130370147.01 (116019776.68 to 145603841.96)	29796.76 (26404.56 to 33520.82)	146337709.10 (131687315.93 to 162671194.59)	31235.29 (27843.32 to 35131.82)
**2011**	276886171.77 (248061548.27 to 308355442.70)	30544.62 (27132.54 to 34330.86)	130472617.72 (116120139.90 to 145632489.12)	29806.74 (26414.58 to 33526.72)	146413554.05 (131793316.18 to 162728836.64)	31229.04 (27824.67 to 35102.39)
**2012**	277277839.73 (248402432.17 to 308599528.74)	30563.50 (27149.84 to 34361.14)	130724041.25 (116307348.15 to 145791936.63)	29841.41 (26442.14 to 33567.87)	146553798.49 (131903237.86 to 162833561.38)	31234.22 (27828.99 to 35088.39)
**2013**	277824172.99 (248888817.65 to 309010005.45)	30589.67 (27171.05 to 34400.28)	131086203.69 (116607455.38 to 146078451.52)	29885.56 (26477.85 to 33629.33)	146737969.30 (131993999.26 to 163017642.07)	31244.83 (27834.07 to 35081.83)

**2014**	278483845.85 (249506734.03 to 309509710.04)	30614.00 (27197.86 to 34425.17)	131516758.51 (116971794.76 to 146435254.21)	29925.57 (26530.26 to 33691.66)	146967087.34 (132106594.67 to 163217961.86)	31256.01 (27826.41 to 35081.23)
**2015**	279141422.04 (250295937.15 to 310212665.28)	30628.56 (27218.00 to 34435.57)	131938062.70 (117341555.67 to 146829171.87)	29949.51 (26563.32 to 33735.85)	147203359.34 (132219193.32 to 163298960.91)	31263.36 (27824.24 to 35071.76)
**2016**	280168459.31 (251095856.30 to 311342732.97)	30684.76 (27231.72 to 34499.43)	132492991.89 (118052030.45 to 147593221.10)	30003.96 (26569.27 to 33786.06)	147675467.42 (132352257.05 to 163899373.61)	31322.62 (27872.53 to 35182.77)
**2017**	281748470.10 (251960626.59 to 313257298.48)	30800.27 (27292.86 to 34676.32)	133272699.42 (118798302.59 to 148748758.44)	30108.80 (26579.55 to 33949.24)	148475770.67 (132848259.92 to 164669724.37)	31449.41 (27969.30 to 35340.37)
**2018**	283272973.98 (253081974.95 to 315077900.13)	30914.83 (27368.49 to 34849.32)	134010742.16 (119282860.07 to 149776897.34)	30210.77 (26648.70 to 34112.82)	149262231.82 (133275048.36 to 165791722.69)	31577.06 (28066.34 to 35546.40)
**2019**	284170816.99 (253848810.92 to 315965010.45)	30971.98 (27399.33 to 34969.13)	134458864.21 (119571391.72 to 150354624.61)	30260.91 (26698.41 to 34215.69)	149711952.78 (133595860.07 to 166566007.70)	31642.23 (28077.75 to 35677.65)
**2020**	282775658.15 (252532797.33 to 314082977.13)	30821.82 (27249.62 to 34815.93)	133558479.70 (118620789.26 to 149223825.76)	30053.88 (26482.69 to 34028.48)	149217178.45 (133213037.54 to 165879863.73)	31548.73 (28002.78 to 35562.82)
**2021**	282494328.80 (252165015.17 to 313416190.31)	30810.85 (27241.14 to 34786.17)	133449338.95 (118544091.44 to 149064034.63)	30043.09 (26465.42 to 33994.50)	149044989.85 (133037481.88 to 165493921.45)	31538.63 (27993.01 to 35543.12)
**Percentage Change, 1990-2021**	0.07% (0.05% to 0.10%)	-0.01% (-0.02% to 0.00%)	0.08% (0.05% to 0.11%)	-0.01% (-0.02% to 0.01%)	0.07% (0.04% to 0.10%)	-0.01% (-0.02% to 0.00%)
**Statistical Analysis**	Spearman's ρ = 0.997 p-value < 0.001	Spearman's ρ = -0.337 p-value = 0.060	Spearman's ρ = 0.997 p-value < 0.001	Spearman's ρ = -0.071 p-value = 0.697	Spearman's ρ = 0.997 p-value < 0.001	Spearman's ρ = -0.495 p-value = 0.004
			Wilcoxon rank sum test between sex (count): p-value < 0.001<br>Wilcoxon rank sum test between sex (rate): p-value < 0.001			

Table S2j: Counts and age-standardized DALY rates of tension-type headache from 1990 to 2021, by sex

	Both		Male		Female	
	Absolute number	Age-standardized rate, per 100 000 people	Absolute number	Age-standardized rate, per 100 000 people	Absolute number	Age-standardized rate, per 100 000 people
**1990**	688630.41 (213826.80 to 2188953.53)	78.70 (23.92 to 257.25)	263119.95 (69992.48 to 963459.65)	62.96 (16.52 to 233.69)	425510.46 (144716.84 to 1237108.07)	93.42 (31.02 to 278.55)
**1991**	692312.78 (214113.13 to 2196810.61)	78.57 (23.63 to 256.60)	264867.55 (70402.80 to 965180.06)	62.89 (16.32 to 233.09)	427445.23 (144955.68 to 1245581.75)	93.26 (30.81 to 278.46)
**1992**	696184.13 (216808.10 to 2209452.57)	78.48 (23.79 to 256.14)	266651.89 (70615.61 to 971971.06)	62.86 (16.25 to 233.36)	429532.25 (145995.85 to 1252088.39)	93.15 (30.89 to 279.10)
**1993**	699923.61 (218192.46 to 2220228.82)	78.43 (23.75 to 256.21)	268201.98 (70878.49 to 974504.39)	62.81 (16.14 to 232.75)	431721.63 (147522.67 to 1257869.57)	93.09 (30.89 to 278.72)
**1994**	703090.90 (219956.16 to 2226145.31)	78.37 (23.79 to 255.85)	269450.42 (71194.18 to 977883.36)	62.76 (16.15 to 232.73)	433640.48 (148785.68 to 1261718.35)	93.05 (30.96 to 277.74)
**1995**	705904.49 (220474.83 to 2233986.82)	78.28 (23.66 to 255.09)	270655.68 (72809.38 to 980308.10)	62.72 (16.22 to 232.26)	435248.81 (148517.83 to 1264333.44)	92.92 (30.66 to 277.34)
**1996**	710468.68 (224021.11 to 2241077.55)	78.38 (23.90 to 255.32)	271984.68 (72673.25 to 982300.89)	62.70 (16.26 to 232.16)	438484.00 (150388.66 to 1273846.50)	93.14 (30.90 to 276.94)
**1997**	716968.23 (226867.12 to 2254886.35)	78.68 (23.98 to 255.45)	273695.17 (74190.95 to 984669.71)	62.76 (16.39 to 231.73)	443273.06 (152099.26 to 1281081.55)	93.69 (31.20 to 277.82)
**1998**	724735.95 (231588.85 to 2260638.08)	79.10 (24.42 to 255.43)	275726.59 (75447.98 to 990923.10)	62.87 (16.53 to 231.74)	449009.36 (156862.75 to 1288540.21)	94.43 (31.91 to 277.42)
**1999**	731402.56 (234771.86 to 2271356.13)	79.39 (24.67 to 255.32)	277503.69 (75219.94 to 995550.52)	62.92 (16.53 to 231.41)	453898.88 (159456.49 to 1296281.36)	94.98 (32.26 to 277.87)
**2000**	736247.94 (237237.31 to 2280348.92)	79.48 (24.78 to 255.77)	279005.26 (75866.37 to 1000230.95)	62.91 (16.49 to 231.91)	457242.68 (161230.09 to 1300424.55)	95.17 (32.37 to 277.60)

**2001**	740006.47 (240564.39 to 2293125.12)	79.46 (24.90 to 255.73)	280461.49 (76850.19 to 1003944.49)	62.91 (16.78 to 232.50)	459544.98 (162338.67 to 1306938.98)	95.15 (32.41 to 277.77)
**2002**	743707.56 (239505.08 to 2304527.57)	79.48 (24.74 to 255.97)	281876.85 (76731.47 to 1010129.60)	62.92 (16.59 to 233.04)	461830.71 (162832.36 to 1311305.58)	95.19 (32.47 to 277.74)
**2003**	747167.44 (241242.06 to 2316737.14)	79.50 (24.68 to 256.16)	283186.98 (76824.33 to 1017527.57)	62.94 (16.53 to 233.82)	463980.45 (164416.85 to 1318171.81)	95.21 (32.42 to 278.22)
**2004**	750754.11 (241988.78 to 2334158.56)	79.53 (24.73 to 256.20)	284476.97 (77350.30 to 1022870.10)	62.96 (16.68 to 234.38)	466277.14 (164409.70 to 1324734.50)	95.28 (32.46 to 279.26)
**2005**	753876.64 (242977.04 to 2341848.88)	79.49 (24.64 to 256.35)	285601.18 (77855.94 to 1022197.55)	62.93 (16.43 to 233.50)	468275.46 (165479.78 to 1330167.40)	95.25 (32.51 to 279.32)
**2006**	756616.96 (243419.31 to 2347414.05)	79.46 (24.62 to 255.88)	286549.20 (78427.95 to 1024276.71)	62.90 (16.38 to 233.34)	470067.75 (164991.36 to 1330085.07)	95.23 (32.48 to 278.73)
**2007**	759357.00 (246106.32 to 2356825.33)	79.46 (24.83 to 256.37)	287651.03 (78662.51 to 1025879.62)	62.93 (16.55 to 233.40)	471705.97 (166811.81 to 1337493.08)	95.22 (32.68 to 279.34)
**2008**	762027.78 (245611.17 to 2359821.97)	79.47 (24.63 to 256.22)	288621.72 (78796.65 to 1028252.97)	62.94 (16.46 to 233.53)	473406.06 (166104.99 to 1338058.78)	95.25 (32.34 to 278.61)
**2009**	764200.66 (248835.34 to 2365459.16)	79.45 (24.82 to 256.29)	289457.53 (80197.34 to 1030837.00)	62.93 (16.57 to 233.75)	474743.13 (169551.30 to 1341480.47)	95.22 (32.91 to 278.97)
**2010**	766527.20 (248631.10 to 2367135.96)	79.44 (24.70 to 256.34)	290347.36 (79872.21 to 1033805.76)	62.95 (16.47 to 234.26)	476179.84 (168628.10 to 1341501.76)	95.20 (32.49 to 278.81)
**2011**	768551.59 (250567.44 to 2372933.50)	79.44 (24.83 to 256.04)	291124.31 (80222.70 to 1032690.12)	62.97 (16.59 to 234.09)	477427.29 (170184.85 to 1343680.84)	95.19 (32.74 to 279.13)
**2012**	770618.13 (250003.15 to 2380904.96)	79.46 (24.78 to 256.30)	292041.45 (81358.75 to 1035114.42)	63.02 (16.71 to 234.09)	478576.68 (168821.16 to 1348428.92)	95.20 (32.55 to 279.39)
**2013**	772787.89 (252205.40 to 2384945.72)	79.47 (24.75 to 256.38)	293116.04 (81908.97 to 1036601.95)	63.07 (16.54 to 234.25)	479671.85 (170300.86 to 1348680.74)	95.18 (32.50 to 279.23)

**2014**	775223.16 (252493.99 to 2393723.80)	79.49 (24.61 to 256.90)	294477.00 (81505.16 to 1040832.20)	63.17 (16.54 to 234.92)	480746.16 (170956.42 to 1355119.99)	95.15 (32.41 to 279.97)
**2015**	777373.70 (253789.00 to 2402372.70)	79.46 (24.53 to 257.29)	295603.21 (82634.64 to 1043990.03)	63.18 (16.60 to 235.28)	481770.48 (171069.82 to 1357653.89)	95.10 (32.17 to 279.83)
**2016**	777432.45 (251774.55 to 2404882.64)	79.24 (24.58 to 257.22)	296592.22 (82488.04 to 1046210.26)	63.18 (16.64 to 235.42)	480840.23 (169286.52 to 1358887.57)	94.71 (32.17 to 280.01)
**2017**	775003.03 (250908.88 to 2417260.25)	78.84 (24.04 to 257.89)	297428.48 (83000.36 to 1049186.88)	63.20 (16.52 to 235.71)	477574.55 (167289.83 to 1361671.83)	93.92 (31.47 to 280.98)
**2018**	772203.73 (246962.98 to 2422266.01)	78.41 (23.67 to 258.09)	298087.23 (81987.28 to 1054783.94)	63.19 (16.25 to 235.77)	474116.51 (163987.13 to 1364937.08)	93.11 (30.82 to 281.25)
**2019**	771380.97 (245182.05 to 2423182.63)	78.21 (23.60 to 258.18)	298663.20 (82172.57 to 1053229.36)	63.18 (16.29 to 235.33)	472717.76 (162342.31 to 1367563.57)	92.75 (30.59 to 281.84)
**2020**	769455.77 (245537.03 to 2409898.81)	77.92 (23.41 to 257.29)	297865.55 (82109.12 to 1047075.05)	62.91 (16.38 to 233.70)	471590.22 (161911.56 to 1363423.68)	92.45 (30.20 to 281.20)
**2021**	767977.02 (245879.07 to 2405182.64)	77.79 (23.49 to 256.54)	297654.68 (83195.64 to 1044194.41)	62.87 (16.40 to 232.91)	470322.34 (162163.34 to 1359954.49)	92.26 (30.36 to 280.15)
**Percentage Change, 1990-2021**	0.12% (0.03% to 0.18%)	-0.01% (-0.06% to 0.02%)	0.13% (0.02% to 0.23%)	0.00% (-0.06% to 0.03%)	0.11% (0.03% to 0.16%)	-0.01% (-0.05% to 0.01%)
**Statistical Analysis**	Spearman's ρ = 0.951 p-value < 0.001	Spearman's ρ = 0.039 p-value = 0.831	Spearman's ρ = 0.997 p-value < 0.001	Spearman's ρ = 0.682 p-value < 0.001	Spearman's ρ = 0.882 p-value < 0.001	Spearman's ρ = 0.065 p-value = 0.726
			Wilcoxon rank sum test between sex (count): p-value < 0.001 Wilcoxon rank sum test between sex (rate): p-value < 0.001			

Table S2k: Counts and age-standardized incidence rates of tension-type headache from 1990 to 2021, by sex

	Both		Male		Female	
	Absolute number	Age-standardized rate, per 100 000 people	Absolute number	Age-standardized rate, per 100 000 people	Absolute number	Age-standardized rate, per 100 000 people
**1990**	90583352.83 (78895825.05 to 101724244.72)	10911.18 (9459.09 to 12276.46)	42988142.58 (37387029.21 to 48468834.20)	10678.34 (9297.48 to 12031.95)	47595210.25 (41570138.12 to 53175114.35)	11118.59 (9626.10 to 12516.34)
**1991**	91011318.70 (79319631.71 to 102179801.33)	10905.47 (9461.17 to 12268.64)	43201287.46 (37601942.39 to 48684753.60)	10671.01 (9294.55 to 12029.03)	47810031.25 (41772957.88 to 53432422.11)	11114.96 (9619.90 to 12507.86)
**1992**	91415671.70 (79704680.07 to 102601884.82)	10898.80 (9462.23 to 12260.06)	43402218.04 (37815463.73 to 48875779.35)	10663.58 (9291.95 to 12024.54)	48013453.66 (41943425.50 to 53660884.13)	11109.62 (9612.26 to 12497.14)
**1993**	91778479.08 (80063469.35 to 102988818.20)	10892.25 (9464.10 to 12251.33)	43579547.48 (38073123.47 to 49057681.98)	10657.04 (9291.14 to 12013.10)	48198931.60 (42094286.38 to 53873041.48)	11103.65 (9603.71 to 12489.19)
**1994**	92045650.25 (80418840.09 to 103288824.71)	10885.97 (9465.64 to 12243.14)	43704429.20 (38283146.28 to 49184904.52)	10651.34 (9295.09 to 12003.11)	48341221.05 (42240737.59 to 54039889.29)	11097.31 (9599.45 to 12475.17)
**1995**	92266364.11 (80742556.77 to 103576434.17)	10880.39 (9467.70 to 12236.47)	43804415.26 (38465075.37 to 49274058.74)	10646.75 (9301.97 to 11995.13)	48461948.85 (42387462.70 to 54206895.14)	11091.23 (9597.46 to 12462.15)
**1996**	92463838.45 (80979597.98 to 103681129.57)	10873.25 (9472.40 to 12221.53)	43899280.33 (38551453.70 to 49268522.39)	10641.56 (9293.77 to 11971.64)	48564558.12 (42501716.90 to 54291158.81)	11082.78 (9600.08 to 12452.61)
**1997**	92625780.63 (81140014.93 to 103775836.45)	10862.69 (9476.27 to 12210.71)	43985446.10 (38620629.97 to 49372904.84)	10634.07 (9295.61 to 11953.53)	48640334.52 (42568128.44 to 54324996.54)	11069.94 (9599.59 to 12448.66)
**1998**	92780959.13 (81271967.61 to 103871095.37)	10849.83 (9471.61 to 12198.94)	44073689.92 (38677478.00 to 49529615.55)	10624.69 (9296.32 to 11932.74)	48707269.20 (42678886.14 to 54343484.19)	11054.44 (9597.40 to 12434.91)
**1999**	92924511.18 (81455528.27 to 103969905.32)	10837.13 (9467.15 to 12182.04)	44154865.45 (38715529.98 to 49673869.46)	10614.83 (9295.10 to 11925.85)	48769645.73 (42829353.38 to 54368285.36)	11039.74 (9599.75 to 12410.52)
**2000**	93081154.65 (81638597.74 to 104097085.33)	10827.94 (9465.64 to 12163.23)	44233580.21 (38740666.56 to 49743418.08)	10606.97 (9283.65 to 11919.97)	48847574.44 (42986215.12 to 54451381.20)	11029.87 (9607.35 to 12391.84)

**2001**	93271858.56 (81840667.12 to 104258518.73)	10823.83 (9459.72 to 12162.00)	44324625.18 (38822700.93 to 49846066.93)	10603.54 (9283.09 to 11921.84)	48947233.38 (43084522.63 to 54533997.70)	11025.59 (9598.94 to 12381.67)
**2002**	93460898.85 (81996611.81 to 104437273.33)	10823.39 (9456.97 to 12159.67)	44416906.67 (38950147.48 to 49930264.35)	10604.36 (9285.81 to 11921.70)	49043992.18 (43158831.95 to 54624075.05)	11024.29 (9593.34 to 12374.46)
**2003**	93627924.94 (82196966.55 to 104609774.82)	10823.98 (9454.66 to 12166.49)	44498277.57 (39068503.32 to 49997885.10)	10606.36 (9288.03 to 11923.21)	49129647.37 (43213911.62 to 54677122.28)	11023.78 (9587.73 to 12382.07)
**2004**	93781555.14 (82377340.13 to 104888397.93)	10823.73 (9451.31 to 12174.06)	44573956.55 (39173085.34 to 50066148.25)	10607.30 (9286.17 to 11933.67)	49207598.60 (43254645.87 to 54798428.61)	11022.65 (9584.96 to 12392.77)
**2005**	93899300.24 (82489907.37 to 105077732.86)	10821.04 (9445.71 to 12173.44)	44630178.05 (39253090.92 to 50126472.79)	10605.39 (9281.32 to 11940.73)	49269122.19 (43324089.27 to 54857508.72)	11019.52 (9586.61 to 12400.87)
**2006**	93958225.59 (82487136.68 to 105143450.19)	10816.83 (9442.98 to 12169.54)	44656801.76 (39258268.88 to 50188980.66)	10601.83 (9278.48 to 11934.09)	49301423.83 (43358124.48 to 54878039.31)	11014.96 (9585.12 to 12394.30)
**2007**	93988520.57 (82531871.16 to 105140766.22)	10813.17 (9441.14 to 12166.35)	44671118.79 (39239898.18 to 50228012.47)	10599.07 (9276.90 to 11929.59)	49317401.79 (43365224.37 to 54876751.29)	11010.70 (9582.90 to 12388.12)
**2008**	94003022.65 (82554707.91 to 105066350.12)	10809.46 (9439.31 to 12163.09)	44679098.25 (39205399.44 to 50220830.55)	10596.18 (9274.57 to 11925.68)	49323924.40 (43351902.01 to 54871972.18)	11006.47 (9579.91 to 12382.15)
**2009**	94003276.56 (82555480.36 to 104954668.24)	10805.99 (9437.66 to 12160.02)	44679475.20 (39142125.62 to 50237842.21)	10593.43 (9271.87 to 11922.31)	49323801.35 (43342607.05 to 54843947.19)	11002.63 (9576.76 to 12377.07)
**2010**	93989485.43 (82536678.69 to 104818749.19)	10803.06 (9435.18 to 12157.00)	44671444.19 (39108805.04 to 50252185.26)	10591.10 (9269.92 to 11919.17)	49318041.24 (43339735.90 to 54798524.48)	10999.48 (9573.89 to 12373.30)
**2011**	93990612.61 (82476605.75 to 104674549.62)	10802.11 (9436.03 to 12155.69)	44668068.67 (39083369.29 to 50231082.91)	10590.56 (9267.39 to 11920.29)	49322543.95 (43355937.34 to 54817689.66)	10998.52 (9579.69 to 12378.62)
**2012**	94052209.68 (82492256.93 to 104683080.61)	10803.23 (9438.23 to 12157.58)	44695366.37 (39092532.13 to 50206957.61)	10591.93 (9265.62 to 11919.29)	49356843.30 (43388560.88 to 54844196.89)	10999.78 (9587.17 to 12386.16)
**2013**	94161095.32 (82527029.23 to 104785391.02)	10804.74 (9439.40 to 12159.79)	44752193.68 (39158720.15 to 50179524.50)	10593.61 (9263.96 to 11917.18)	49408901.64 (43400864.10 to 54881583.92)	11001.54 (9595.43 to 12391.79)

**2014**	94320584.74 (82629193.64 to 105017235.81)	10805.48 (9439.28 to 12159.94)	44840440.77 (39203286.94 to 50231506.33)	10594.52 (9262.13 to 11913.80)	49480143.97 (43467347.67 to 54967877.63)	11002.59 (9604.04 to 12396.38)
**2015**	94505324.35 (82770802.43 to 105308450.97)	10804.62 (9439.01 to 12156.10)	44947886.84 (39291891.31 to 50292621.49)	10594.04 (9260.54 to 11907.84)	49557437.52 (43542345.80 to 54997018.51)	11001.86 (9611.55 to 12398.92)
**2016**	94753327.01 (82945596.40 to 105579087.08)	10811.46 (9444.10 to 12169.61)	45085203.07 (39408848.97 to 50465011.44)	10600.68 (9267.06 to 11919.62)	49668123.94 (43595684.46 to 55168952.74)	11009.33 (9605.07 to 12418.74)
**2017**	95085748.64 (83187493.57 to 105973050.17)	10828.80 (9449.64 to 12186.74)	45258702.59 (39550600.72 to 50671920.63)	10616.76 (9277.10 to 11950.78)	49827046.05 (43700852.26 to 55407492.75)	11028.24 (9607.78 to 12450.44)
**2018**	95400250.54 (83417053.61 to 106341988.79)	10846.42 (9453.82 to 12206.80)	45419208.53 (39684718.23 to 50844865.25)	10632.81 (9275.13 to 11984.01)	49981042.01 (43854238.49 to 55644313.85)	11047.69 (9611.47 to 12442.00)
**2019**	95588913.98 (83563743.78 to 106566529.72)	10854.76 (9449.40 to 12214.81)	45517357.10 (39773077.52 to 50958152.76)	10640.16 (9265.41 to 11991.86)	50071556.88 (43960321.99 to 55804228.69)	11057.33 (9607.55 to 12413.21)
**2020**	95337277.52 (83321477.16 to 106141070.92)	10825.20 (9433.99 to 12184.91)	45381222.13 (39637734.22 to 50762220.89)	10604.42 (9247.73 to 11950.39)	49956055.39 (43841624.55 to 55614186.39)	11034.46 (9597.77 to 12396.39)
**2021**	95235226.48 (83299117.39 to 105938467.33)	10822.21 (9430.78 to 12181.47)	45338106.97 (39635650.34 to 50694953.06)	10601.67 (9245.89 to 11946.76)	49897119.51 (43827321.21 to 55512992.28)	11031.48 (9595.45 to 12389.90)
**Percentage Change, 1990-2021**	0.05% (0.02% to 0.08%)	-0.01% (-0.02% to 0.00%)	0.05% (0.02% to 0.09%)	-0.01% (-0.02% to 0.00%)	0.05% (0.02% to 0.08%)	-0.01% (-0.02% to 0.00%)
**Statistical Analysis**	Spearman's ρ = 0.994 p-value < 0.001	Spearman's ρ = -0.650 p-value < 0.001	Spearman's ρ = 0.992 p-value < 0.001	Spearman's ρ = -0.641 p-value < 0.001	Spearman's ρ = 0.993 p-value < 0.001	Spearman's ρ = -0.612 p-value < 0.001
	Wilcoxon rank sum test between sex (count): p-value < 0.001 Wilcoxon rank sum test between sex (rate): p-value < 0.001					

Table S2I: Counts and age-standardized YLD rates of tension-type headache from 1990 to 2021, by sex

	Both		Male		Female	
	Absolute number	Age-standardized rate, per 100 000 people	Absolute number	Age-standardized rate, per 100 000 people	Absolute number	Age-standardized rate, per 100 000 people
**1990**	688630.41 (213826.80 to 2188953.53)	78.70 (23.92 to 257.25)	263119.95 (69992.48 to 963459.65)	62.96 (16.52 to 233.69)	425510.46 (144716.84 to 1237108.07)	93.42 (31.02 to 278.55)
**1991**	692312.78 (214113.13 to 2196810.61)	78.57 (23.63 to 256.60)	264867.55 (70402.80 to 965180.06)	62.89 (16.32 to 233.09)	427445.23 (144955.68 to 1245581.75)	93.26 (30.81 to 278.46)
**1992**	696184.13 (216808.10 to 2209452.57)	78.48 (23.79 to 256.14)	266651.89 (70615.61 to 971971.06)	62.86 (16.25 to 233.36)	429532.25 (145995.85 to 1252088.39)	93.15 (30.89 to 279.10)
**1993**	699923.61 (218192.46 to 2220228.82)	78.43 (23.75 to 256.21)	268201.98 (70878.49 to 974504.39)	62.81 (16.14 to 232.75)	431721.63 (147522.67 to 1257869.57)	93.09 (30.89 to 278.72)
**1994**	703090.90 (219956.16 to 2226145.31)	78.37 (23.79 to 255.85)	269450.42 (71194.18 to 977883.36)	62.76 (16.15 to 232.73)	433640.48 (148785.68 to 1261718.35)	93.05 (30.96 to 277.74)
**1995**	705904.49 (220474.83 to 2233986.82)	78.28 (23.66 to 255.09)	270655.68 (72809.38 to 980308.10)	62.72 (16.22 to 232.26)	435248.81 (148517.83 to 1264333.44)	92.92 (30.66 to 277.34)
**1996**	710468.68 (224021.11 to 2241077.55)	78.38 (23.90 to 255.32)	271984.68 (72673.25 to 982300.89)	62.70 (16.26 to 232.16)	438484.00 (150388.66 to 1273846.50)	93.14 (30.90 to 276.94)
**1997**	716968.23 (226867.12 to 2254886.35)	78.68 (23.98 to 255.45)	273695.17 (74190.95 to 984669.71)	62.76 (16.39 to 231.73)	443273.06 (152099.26 to 1281081.55)	93.69 (31.20 to 277.82)
**1998**	724735.95 (231588.85 to 2260638.08)	79.10 (24.42 to 255.43)	275726.59 (75447.98 to 990923.10)	62.87 (16.53 to 231.74)	449009.36 (156862.75 to 1288540.21)	94.43 (31.91 to 277.42)
**1999**	731402.56 (234771.86 to 2271356.13)	79.39 (24.67 to 255.32)	277503.69 (75219.94 to 995550.52)	62.92 (16.53 to 231.41)	453898.88 (159456.49 to 1296281.36)	94.98 (32.26 to 277.87)
**2000**	736247.94 (237237.31 to 2280348.92)	79.48 (24.78 to 255.77)	279005.26 (75866.37 to 1000230.95)	62.91 (16.49 to 231.91)	457242.68 (161230.09 to 1300424.55)	95.17 (32.37 to 277.60)

**2001**	740006.47 (240564.39 to 2293125.12)	79.46 (24.90 to 255.73)	280461.49 (76850.19 to 1003944.49)	62.91 (16.78 to 232.50)	459544.98 (162338.67 to 1306938.98)	95.15 (32.41 to 277.77)
**2002**	743707.56 (239505.08 to 2304527.57)	79.48 (24.74 to 255.97)	281876.85 (76731.47 to 1010129.60)	62.92 (16.59 to 233.04)	461830.71 (162832.36 to 1311305.58)	95.19 (32.47 to 277.74)
**2003**	747167.44 (241242.06 to 2316737.14)	79.50 (24.68 to 256.16)	283186.98 (76824.33 to 1017527.57)	62.94 (16.53 to 233.82)	463980.45 (164416.85 to 1318171.81)	95.21 (32.42 to 278.22)
**2004**	750754.11 (241988.78 to 2334158.56)	79.53 (24.73 to 256.20)	284476.97 (77350.30 to 1022870.10)	62.96 (16.68 to 234.38)	466277.14 (164409.70 to 1324734.50)	95.28 (32.46 to 279.26)
**2005**	753876.64 (242977.04 to 2341848.88)	79.49 (24.64 to 256.35)	285601.18 (77855.94 to 1022197.55)	62.93 (16.43 to 233.50)	468275.46 (165479.78 to 1330167.40)	95.25 (32.51 to 279.32)
**2006**	756616.96 (243419.31 to 2347414.05)	79.46 (24.62 to 255.88)	286549.20 (78427.95 to 1024276.71)	62.90 (16.38 to 233.34)	470067.75 (164991.36 to 1330085.07)	95.23 (32.48 to 278.73)
**2007**	759357.00 (246106.32 to 2356825.33)	79.46 (24.83 to 256.37)	287651.03 (78662.51 to 1025879.62)	62.93 (16.55 to 233.40)	471705.97 (166811.81 to 1337493.08)	95.22 (32.68 to 279.34)
**2008**	762027.78 (245611.17 to 2359821.97)	79.47 (24.63 to 256.22)	288621.72 (78796.65 to 1028252.97)	62.94 (16.46 to 233.53)	473406.06 (166104.99 to 1338058.78)	95.25 (32.34 to 278.61)
**2009**	764200.66 (248835.34 to 2365459.16)	79.45 (24.82 to 256.29)	289457.53 (80197.34 to 1030837.00)	62.93 (16.57 to 233.75)	474743.13 (169551.30 to 1341480.47)	95.22 (32.91 to 278.97)
**2010**	766527.20 (248631.10 to 2367135.96)	79.44 (24.70 to 256.34)	290347.36 (79872.21 to 1033805.76)	62.95 (16.47 to 234.26)	476179.84 (168628.10 to 1341501.76)	95.20 (32.49 to 278.81)
**2011**	768551.59 (250567.44 to 2372933.50)	79.44 (24.83 to 256.04)	291124.31 (80222.70 to 1032690.12)	62.97 (16.59 to 234.09)	477427.29 (170184.85 to 1343680.84)	95.19 (32.74 to 279.13)
**2012**	770618.13 (250003.15 to 2380904.96)	79.46 (24.78 to 256.30)	292041.45 (81358.75 to 1035114.42)	63.02 (16.71 to 234.09)	478576.68 (168821.16 to 1348428.92)	95.20 (32.55 to 279.39)
**2013**	772787.89 (252205.40 to 2384945.72)	79.47 (24.75 to 256.38)	293116.04 (81908.97 to 1036601.95)	63.07 (16.54 to 234.25)	479671.85 (170300.86 to 1348680.74)	95.18 (32.50 to 279.23)

**2014**	775223.16 (252493.99 to 2393723.80)	79.49 (24.61 to 256.90)	294477.00 (81505.16 to 1040832.20)	63.17 (16.54 to 234.92)	480746.16 (170956.42 to 1355119.99)	95.15 (32.41 to 279.97)
**2015**	777373.70 (253789.00 to 2402372.70)	79.46 (24.53 to 257.29)	295603.21 (82634.64 to 1043990.03)	63.18 (16.60 to 235.28)	481770.48 (171069.82 to 1357653.89)	95.10 (32.17 to 279.83)
**2016**	777432.45 (251774.55 to 2404882.64)	79.24 (24.58 to 257.22)	296592.22 (82488.04 to 1046210.26)	63.18 (16.64 to 235.42)	480840.23 (169286.52 to 1358887.57)	94.71 (32.17 to 280.01)
**2017**	775003.03 (250908.88 to 2417260.25)	78.84 (24.04 to 257.89)	297428.48 (83000.36 to 1049186.88)	63.20 (16.52 to 235.71)	477574.55 (167289.83 to 1361671.83)	93.92 (31.47 to 280.98)
**2018**	772203.73 (246962.98 to 2422266.01)	78.41 (23.67 to 258.09)	298087.23 (81987.28 to 1054783.94)	63.19 (16.25 to 235.77)	474116.51 (163987.13 to 1364937.08)	93.11 (30.82 to 281.25)
**2019**	771380.97 (245182.05 to 2423182.63)	78.21 (23.60 to 258.18)	298663.20 (82172.57 to 1053229.36)	63.18 (16.29 to 235.33)	472717.76 (162342.31 to 1367563.57)	92.75 (30.59 to 281.84)
**2020**	769455.77 (245537.03 to 2409898.81)	77.92 (23.41 to 257.29)	297865.55 (82109.12 to 1047075.05)	62.91 (16.38 to 233.70)	471590.22 (161911.56 to 1363423.68)	92.45 (30.20 to 281.20)
**2021**	767977.02 (245879.07 to 2405182.64)	77.79 (23.49 to 256.54)	297654.68 (83195.64 to 1044194.41)	62.87 (16.40 to 232.91)	470322.34 (162163.34 to 1359954.49)	92.26 (30.36 to 280.15)
**Percentage Change, 1990-2021**	0.12% (0.03% to 0.18%)	-0.01% (-0.06% to 0.02%)	0.13% (0.02% to 0.23%)	0.00% (-0.06% to 0.03%)	0.11% (0.03% to 0.16%)	-0.01% (-0.05% to 0.01%)
**Statistical Analysis**	688630.41 (213826.80 to 2188953.53)	78.70 (23.92 to 257.25)	263119.95 (69992.48 to 963459.65)	62.96 (16.52 to 233.69)	425510.46 (144716.84 to 1237108.07)	93.42 (31.02 to 278.55)
				Wilcoxon rank sum test between sex (count): p-value < 0.001<br>Wilcoxon rank sum test between sex (rate): p-value < 0.001		

Table S3a: Counts and age-standardized prevalence rates of headache disorders in 2021, and percentage changes from 1990 to 2021, by country and sex

	Male			Female		
	**Absolute number, 2021**	**Age-standardized rate, per 100 000 people, 2021**	**Percentage Change, 1990-2021**	**Absolute number, 2021**	**Age-standardized rate, per 100 000 people, 2021**	**Percentage Change, 1990-2021**
**Europe**	**163430491.08 (149177588.93 to 177041537.85)**	**36910.27 (33762.87 to 40317.43)**	**0.00% (-0.01% to 0.01%)**	**207161139.44 (192553758.31 to 220826723.58)**	**44416.59 (41290.00 to 47491.58)**	**0.00% (-0.01% to 0.01%)**
Albania	501402.49 (449487.87 to 554845.26)	34617.34 (30993.45 to 38410.01)	0.00% (0.00% to 0.00%)	610374.02 (559877.60 to 663521.28)	42424.21 (39052.91 to 46174.27)	0.00% (0.00% to 0.00%)
Andorra	19081.98 (17127.41 to 21053.94)	38569.63 (34565.68 to 42539.73)	0.00% (0.00% to 0.00%)	21829.81 (20091.46 to 23679.83)	46977.08 (43262.21 to 50654.93)	0.00% (0.00% to 0.00%)
Austria	1826515.70 (1651181.65 to 2005643.87)	38175.65 (34228.37 to 42089.80)	0.00% (0.00% to 0.00%)	2190405.88 (2012076.13 to 2358433.81)	45216.16 (41744.17 to 48708.46)	0.00% (0.00% to 0.00%)
Belarus	1663360.14 (1498273.21 to 1850103.06)	35513.66 (31976.20 to 39618.83)	0.00% (0.00% to 0.00%)	2210717.76 (2031047.54 to 2405727.90)	41305.95 (37857.98 to 44685.48)	0.00% (0.00% to 0.00%)
Belgium	2393761.39 (2159698.92 to 2618196.98)	40082.72 (36069.24 to 43878.49)	0.01% (-0.01% to 0.03%)	3086398.22 (2857571.39 to 3324073.86)	50426.47 (46775.23 to 54094.88)	0.03% (0.00% to 0.06%)
Bosnia and Herzegovina	608793.75 (549772.01 to 672481.76)	34617.32 (30995.34 to 38406.18)	0.00% (0.00% to 0.00%)	779167.13 (714886.35 to 846420.68)	42424.19 (39012.53 to 46216.36)	0.00% (0.00% to 0.00%)
Bulgaria	1238561.63 (1112938.52 to 1364156.50)	34617.11 (30964.76 to 38442.47)	0.00% (0.00% to 0.01%)	1601510.42 (1470542.38 to 1735742.20)	42424.27 (38928.57 to 46191.77)	0.00% (0.00% to 0.00%)
Croatia	770254.98 (697199.06 to 850331.16)	34620.19 (31268.04 to 38373.33)	0.00% (-0.04% to 0.04%)	992525.37 (909529.82 to 1075203.35)	42378.06 (39016.10 to 46186.35)	0.00% (-0.03% to 0.03%)
Cyprus	281236.73 (253452.91 to 310199.56)	38568.75 (34566.94 to 42631.73)	0.00% (0.00% to 0.00%)	355365.37 (327813.14 to 385443.56)	46977.50 (43272.16 to 50698.02)	0.00% (0.00% to 0.00%)

Czechia	1949186.60 (1759377.53 to 2148768.60)	34616.84 (31002.41 to 38438.99)	0.00% (0.00% to 0.00%)	2447030.64 (2249219.62 to 2646261.23)	42423.77 (38984.18 to 46228.80)	0.00% (0.00% to 0.00%)
Denmark	1168625.49 (1050119.71 to 1286272.98)	37873.38 (34206.73 to 41849.79)	0.00% (-0.04% to 0.04%)	1465030.21 (1352393.56 to 1581524.72)	47301.01 (43539.41 to 50975.75)	-0.01% (-0.05% to 0.02%)
Estonia	236313.70 (213314.54 to 262019.29)	35513.97 (31991.04 to 39628.43)	0.00% (0.00% to 0.00%)	301984.04 (277836.54 to 328701.00)	41305.92 (37839.78 to 44726.54)	0.00% (0.00% to 0.00%)
Finland	1117495.36 (1008469.04 to 1230121.11)	38569.63 (34618.57 to 42528.01)	0.00% (0.00% to 0.00%)	1370746.87 (1265693.13 to 1483122.42)	46977.26 (43276.06 to 50663.50)	0.00% (0.00% to 0.00%)
France	13013153.05 (11753021.54 to 14279496.65)	38717.59 (34759.39 to 42546.14)	0.00% (-0.02% to 0.01%)	15997001.26 (14791875.17 to 17290828.73)	45215.58 (41773.16 to 48714.61)	0.00% (-0.02% to 0.01%)
Germany	17943044.68 (16313519.19 to 19646163.18)	39409.55 (35774.91 to 43287.37)	0.02% (-0.02% to 0.06%)	22218595.36 (20598356.38 to 23807745.14)	49337.96 (45780.76 to 52868.34)	0.02% (-0.01% to 0.05%)
Greece	2057233.67 (1851218.13 to 2251802.84)	39022.46 (35093.57 to 42805.66)	0.00% (-0.01% to 0.01%)	2621917.39 (2410355.15 to 2833092.52)	47297.06 (43539.46 to 50921.60)	0.00% (-0.02% to 0.02%)
Hungary	1723834.18 (1557320.76 to 1902388.78)	34617.60 (31016.73 to 38370.60)	0.00% (0.00% to 0.00%)	2301671.26 (2116755.20 to 2492119.74)	42424.71 (38968.67 to 46206.69)	0.00% (0.00% to 0.00%)
Iceland	72366.09 (65352.75 to 79778.55)	38570.50 (34552.05 to 42563.72)	0.00% (0.00% to 0.00%)	85122.69 (78667.00 to 91973.58)	46976.96 (43241.12 to 50653.06)	0.00% (0.00% to 0.00%)
Ireland	987102.77 (890397.84 to 1086248.23)	38569.63 (34535.62 to 42621.90)	0.00% (0.00% to 0.00%)	1235626.91 (1140714.63 to 1335358.35)	46976.73 (43290.22 to 50631.56)	0.00% (0.00% to 0.00%)
Israel	1825994.21 (1640563.71 to 2008923.98)	38568.98 (34576.83 to 42552.00)	0.00% (0.00% to 0.00%)	2245258.31 (2071370.57 to 2421003.50)	46977.06 (43255.19 to 50720.37)	0.00% (0.00% to 0.00%)
Italy	12684746.77 (11671185.85 to 13616225.81)	40460.56 (37380.69 to 43660.02)	0.00% (-0.02% to 0.03%)	15917995.62 (14829557.44 to 16960614.46)	48918.21 (45734.52 to 51941.37)	0.00% (-0.02% to 0.02%)
Latvia	327378.13 (295286.50 to 363562.79)	35513.47 (31963.02 to 39565.03)	0.00% (-0.01% to 0.00%)	440387.65 (404357.39 to 479178.21)	41304.96 (37834.01 to 44697.49)	0.00% (0.00% to 0.00%)

Lithuania	491626.98 (440792.17 to 546772.16)	36325.85 (32430.25 to 40557.22)	0.00% (0.00% to 0.00%)	655646.35 (601401.16 to 708936.36)	41960.61 (38390.31 to 45546.74)	0.00% (0.00% to 0.00%)
Luxembourg	137878.41 (124403.70 to 152568.19)	38861.61 (35102.11 to 43119.56)	0.00% (0.00% to 0.00%)	159488.09 (146922.47 to 171598.45)	45901.04 (42400.97 to 49447.45)	0.00% (0.00% to 0.00%)
Malta	90785.88 (81752.95 to 99894.88)	38568.56 (34554.84 to 42618.63)	0.00% (0.00% to 0.00%)	109154.82 (100637.50 to 118151.57)	46977.38 (43263.35 to 50655.22)	0.00% (0.00% to 0.00%)
Monaco	7431.92 (6699.62 to 8199.75)	38569.96 (34597.02 to 42514.03)	0.00% (0.00% to 0.00%)	9502.07 (8710.66 to 10301.00)	46976.99 (43302.52 to 50683.76)	0.00% (0.00% to 0.00%)
Montenegro	113204.20 (102183.49 to 125178.60)	34617.37 (30987.91 to 38411.05)	0.00% (0.00% to 0.00%)	142743.80 (130960.95 to 155230.47)	42424.28 (39065.27 to 46183.03)	0.00% (0.00% to 0.00%)
Netherlands	3691279.64 (3312748.27 to 4056654.86)	40632.84 (36287.43 to 44823.77)	0.01% (0.00% to 0.03%)	4440376.91 (4110411.66 to 4782205.84)	48431.43 (44588.59 to 52223.67)	0.01% (0.00% to 0.03%)
North Macedonia	423325.27 (382113.42 to 468779.35)	34616.94 (31023.47 to 38438.68)	0.00% (0.00% to 0.00%)	503225.93 (462206.84 to 546848.08)	42424.26 (39001.37 to 46198.39)	0.00% (0.00% to 0.00%)
Norway	1184311.05 (1085337.35 to 1278456.38)	40625.92 (37252.31 to 43990.14)	0.03% (0.00% to 0.06%)	1418998.39 (1328160.05 to 1510108.05)	50016.78 (46828.78 to 53151.60)	0.02% (0.00% to 0.04%)
Poland	7347164.07 (6722733.82 to 7991897.54)	36323.24 (33269.48 to 39677.43)	0.00% (0.00% to 0.00%)	9438683.43 (8762127.72 to 10097610.38)	44025.19 (40814.13 to 47195.39)	0.00% (0.00% to 0.00%)
Portugal	2086906.50 (1881885.95 to 2294505.94)	38569.69 (34602.32 to 42563.48)	0.00% (0.00% to 0.00%)	2787884.79 (2565048.86 to 3012725.29)	46976.92 (43276.82 to 50689.56)	0.00% (0.00% to 0.00%)
Republic of Moldova	674794.39 (608949.04 to 751911.66)	35513.05 (31956.95 to 39583.72)	0.00% (0.00% to 0.00%)	858439.75 (786238.79 to 935049.43)	41305.87 (37840.55 to 44803.49)	0.00% (0.00% to 0.00%)
Romania	3441383.85 (3103831.35 to 3793397.54)	34617.21 (31069.85 to 38428.56)	0.00% (0.00% to 0.00%)	4429193.35 (4073265.54 to 4804624.89)	42423.90 (39052.36 to 46181.14)	0.00% (0.00% to 0.00%)
Russian Federation	26763522.83 (24613256.10 to 29078254.35)	36909.65 (33940.01 to 40242.33)	0.00% (-0.02% to 0.04%)	35568872.88 (33102316.55 to 38057029.48)	42803.25 (39809.18 to 45764.61)	0.00% (-0.02% to 0.03%)

San Marino	6493.72 (5864.12 to 7148.39)	38568.97 (34540.35 to 42582.14)	0.00% (0.00% to 0.00%)	8543.86 (7862.48 to 9238.46)	46977.10 (43258.11 to 50679.82)	0.00% (0.00% to 0.00%)
Serbia	1696706.43 (1535944.70 to 1873038.06)	34623.66 (31022.12 to 38386.19)	0.00% (0.00% to 0.00%)	2077120.73 (1903548.17 to 2249837.50)	42427.86 (38917.38 to 46182.23)	0.00% (0.00% to 0.00%)
Slovakia	1000589.49 (901403.81 to 1108203.31)	34617.70 (31033.22 to 38425.45)	0.00% (0.00% to 0.00%)	1278330.02 (1170987.69 to 1385532.19)	42423.93 (38895.18 to 46177.91)	0.00% (0.00% to 0.00%)
Slovenia	384589.07 (346184.94 to 423997.73)	34617.99 (30987.74 to 38476.87)	0.00% (0.00% to 0.00%)	472450.95 (433707.24 to 512041.49)	42424.44 (38959.04 to 46183.44)	0.00% (0.00% to 0.00%)
Spain	9310338.43 (8459072.68 to 10188427.55)	38755.29 (34993.92 to 42572.20)	0.00% (0.00% to 0.01%)	11495489.30 (10594596.29 to 12460455.38)	46379.76 (42731.95 to 50127.76)	0.00% (0.00% to 0.00%)
Sweden	2123476.19 (1953775.57 to 2289717.68)	38799.97 (35745.58 to 42219.24)	0.00% (-0.03% to 0.03%)	2685793.81 (2508733.59 to 2849645.10)	49970.30 (46676.95 to 53170.23)	0.00% (-0.02% to 0.02%)
Switzerland	1703833.80 (1530952.95 to 1878971.92)	35659.63 (32155.86 to 39378.54)	0.03% (-0.01% to 0.09%)	2076028.28 (1907649.39 to 2251827.63)	43777.65 (40286.68 to 47237.42)	0.02% (-0.01% to 0.06%)
Ukraine	8197816.98 (7513185.08 to 8916935.81)	37124.21 (34087.74 to 40483.94)	0.00% (0.00% to 0.00%)	10894423.81 (10096470.92 to 11667213.79)	42894.62 (39735.60 to 45955.41)	0.00% (0.00% to 0.00%)
United Kingdom	13833963.20 (12654905.37 to 14973758.92)	39323.60 (36151.90 to 42638.08)	0.00% (-0.01% to 0.01%)	17417171.16 (16274104.77 to 18549984.14)	47648.03 (44554.03 to 50637.60)	0.00% (-0.01% to 0.01%)

Table S3b: Counts and age-standardized DALY rates of headache disorders in 2021, and percentage changes from 1990 to 2021, by country and sex

	Male			Female		
	**Absolute number, 2021**	**Age-standardized rate, per 100 000 people, 2021**	**Percentage Change, 1990-2021**	**Absolute number, 2021**	**Age-standardized rate, per 100 000 people, 2021**	**Percentage Change, 1990-2021**
**Europe**	**2156683.04 (610227.14 to 4441134.25)**	**483.36 (120.11 to 1021.87)**	**0.01% (-0.01% to 0.03%)**	**4188380.05 (1191901.20 to 8451079.17)**	**908.18 (223.50 to 1866.52)**	**0.01% (-0.02% to 0.03%)**
Albania	6370.36 (2016.95 to 12857.39)	431.91 (124.11 to 886.67)	0.00% (-0.04% to 0.05%)	10953.67 (3062.68 to 23179.72)	750.32 (194.01 to 1613.90)	0.00% (-0.04% to 0.03%)
Andorra	241.91 (50.95 to 519.53)	489.24 (86.07 to 1076.87)	0.00% (-0.05% to 0.05%)	442.21 (102.11 to 915.54)	958.55 (185.68 to 2053.42)	0.00% (-0.04% to 0.03%)
Austria	22315.45 (5453.43 to 47835.01)	469.87 (98.02 to 1033.84)	0.00% (-0.07% to 0.06%)	42848.78 (10379.57 to 87853.27)	910.67 (186.47 to 1925.91)	0.00% (-0.04% to 0.04%)
Belarus	20850.31 (8145.28 to 40680.20)	421.65 (149.25 to 850.44)	0.00% (-0.05% to 0.05%)	44186.63 (16238.71 to 85779.46)	798.55 (262.61 to 1606.64)	0.00% (-0.04% to 0.03%)
Belgium	33742.99 (5848.17 to 72433.05)	575.67 (85.23 to 1269.70)	0.03% (-0.07% to 0.12%)	69266.67 (12490.45 to 146826.94)	1166.28 (185.12 to 2528.78)	0.08% (-0.03% to 0.18%)
Bosnia and Herzegovina	7817.58 (2573.11 to 15554.73)	430.04 (123.89 to 890.30)	0.00% (-0.05% to 0.06%)	13864.93 (4050.40 to 29021.25)	746.95 (193.03 to 1610.11)	0.00% (-0.04% to 0.04%)
Bulgaria	15924.73 (5165.18 to 31875.74)	430.56 (122.90 to 892.84)	0.00% (-0.04% to 0.05%)	28199.19 (8349.48 to 58779.52)	747.64 (192.01 to 1603.14)	0.00% (-0.04% to 0.03%)
Croatia	9866.88 (3231.66 to 19757.21)	430.47 (125.14 to 887.58)	0.00% (-0.06% to 0.07%)	17534.41 (5132.77 to 36425.16)	750.60 (193.44 to 1626.08)	0.00% (-0.05% to 0.06%)
Cyprus	3578.44 (728.20 to 7719.10)	490.73 (88.25 to 1082.27)	0.00% (-0.06% to 0.06%)	7264.48 (1573.34 to 15031.96)	960.26 (183.55 to 2052.65)	0.00% (-0.04% to 0.03%)
Czechia	25017.40 (8263.60 to 49788.50)	430.94 (125.69 to 889.86)	0.00% (-0.04% to 0.05%)	43133.67 (12354.18 to 90639.44)	748.37 (190.88 to 1628.33)	0.00% (-0.04% to 0.04%)

Denmark	13524.04<br>(3306.92 to 28831.59)	445.32<br>(93.96 to 969.36)	0.02%<br>(-0.05% to 0.10%)	25675.53<br>(5828.86 to 53318.55)	863.67<br>(170.89 to 1820.81)	0.02%<br>(-0.05% to 0.10%)
Estonia	2940.07<br>(1157.74 to 5755.00)	422.10<br>(150.58 to 852.54)	0.01%<br>(-0.04% to 0.06%)	5886.78<br>(2202.68 to 11427.82)	800.29<br>(265.57 to 1598.27)	0.00%<br>(-0.03% to 0.03%)
Finland	13769.28<br>(2825.38 to 29427.60)	487.86<br>(86.78 to 1073.33)	0.00%<br>(-0.05% to 0.06%)	26579.26<br>(5975.21 to 54488.41)	959.39<br>(184.19 to 2035.43)	0.00%<br>(-0.04% to 0.04%)
France	171067.16<br>(38864.67 to 362011.25)	516.78<br>(101.13 to 1110.26)	-0.01%<br>(-0.08% to 0.07%)	326156.60<br>(84679.31 to 657207.83)	951.73<br>(216.88 to 1950.88)	-0.01%<br>(-0.07% to 0.06%)
Germany	231513.59<br>(49952.83 to 488124.83)	519.55<br>(96.00 to 1110.27)	0.03%<br>(-0.05% to 0.11%)	455840.62<br>(105101.56 to 925890.88)	1056.88<br>(196.66 to 2225.78)	0.02%<br>(-0.05% to 0.10%)
Greece	26385.85<br>(5314.50 to 57855.17)	511.88<br>(85.80 to 1147.56)	-0.01%<br>(-0.08% to 0.06%)	51872.16<br>(11669.93 to 106816.74)	974.19<br>(182.76 to 2078.22)	0.00%<br>(-0.07% to 0.06%)
Hungary	22259.54<br>(7356.73 to 44486.06)	432.01<br>(127.54 to 905.19)	0.01%<br>(-0.04% to 0.06%)	40668.51<br>(12096.93 to 85032.66)	749.06<br>(195.03 to 1618.28)	0.00%<br>(-0.04% to 0.05%)
Iceland	912.46<br>(176.61 to 1956.59)	490.85<br>(86.42 to 1084.95)	0.00%<br>(-0.05% to 0.05%)	1709.34<br>(367.22 to 3579.01)	962.07<br>(186.91 to 2055.02)	0.00%<br>(-0.04% to 0.04%)
Ireland	12408.68<br>(2454.33 to 27048.04)	487.86<br>(83.73 to 1074.14)	0.00%<br>(-0.06% to 0.05%)	24789.04<br>(5401.03 to 51149.51)	955.72<br>(183.76 to 2041.89)	0.00%<br>(-0.04% to 0.03%)
Israel	23134.32<br>(4213.83 to 51247.55)	491.42<br>(86.90 to 1094.13)	0.00%<br>(-0.05% to 0.05%)	45309.50<br>(8942.59 to 96078.05)	963.01<br>(183.39 to 2071.68)	0.00%<br>(-0.04% to 0.03%)
Italy	163472.55<br>(33294.01 to 350580.00)	532.62<br>(89.26 to 1172.86)	0.01%<br>(-0.04% to 0.06%)	325272.52<br>(69737.49 to 678546.36)	1049.23<br>(181.47 to 2232.96)	0.02%<br>(-0.04% to 0.06%)
Latvia	4088.12<br>(1615.91 to 7883.34)	421.56<br>(149.85 to 844.53)	0.01%<br>(-0.04% to 0.06%)	8624.94<br>(3244.73 to 16692.39)	800.04<br>(266.40 to 1590.50)	0.00%<br>(-0.03% to 0.04%)
Lithuania	5367.77<br>(2047.75 to 10550.65)	377.19<br>(132.14 to 761.61)	0.01%<br>(-0.05% to 0.05%)	11835.40<br>(4134.77 to 23161.69)	752.52<br>(229.49 to 1528.20)	0.00%<br>(-0.04% to 0.03%)

Luxembourg	1773.69 (415.55 to 3736.14)	497.39 (103.85 to 1064.70)	0.01% (-0.04% to 0.06%)	3269.10 (831.51 to 6579.89)	944.96 (215.19 to 1929.69)	0.00% (-0.03% to 0.03%)
Malta	1131.17 (247.14 to 2435.76)	490.02 (88.35 to 1087.21)	0.00% (-0.05% to 0.06%)	2142.87 (482.01 to 4411.82)	960.48 (181.64 to 2045.71)	0.00% (-0.03% to 0.03%)
Monaco	90.96 (20.53 to 191.79)	489.59 (87.80 to 1092.18)	0.00% (-0.05% to 0.05%)	183.19 (43.29 to 374.59)	957.44 (183.64 to 2036.52)	-0.01% (-0.04% to 0.03%)
Montenegro	1452.64 (464.57 to 2961.25)	432.13 (124.96 to 894.11)	0.00% (-0.04% to 0.06%)	2551.12 (722.61 to 5432.53)	747.55 (189.86 to 1619.27)	-0.01% (-0.04% to 0.03%)
Netherlands	41406.73 (8074.70 to 92831.79)	468.09 (78.36 to 1070.25)	0.07% (-0.05% to 0.19%)	81505.26 (16294.50 to 171216.98)	928.70 (157.97 to 1981.10)	0.04% (-0.04% to 0.11%)
North Macedonia	5484.49 (1752.60 to 11053.89)	431.69 (124.56 to 887.98)	0.00% (-0.05% to 0.05%)	9090.58 (2547.79 to 19218.16)	746.84 (191.54 to 1611.94)	0.00% (-0.04% to 0.03%)
Norway	14390.67 (2856.02 to 31524.09)	499.29 (87.89 to 1109.11)	0.09% (0.02% to 0.16%)	26422.71 (5385.36 to 55234.48)	961.46 (170.20 to 2049.84)	0.07% (0.00% to 0.12%)
Poland	95963.54 (33133.53 to 192959.82)	457.39 (137.89 to 955.96)	0.01% (-0.02% to 0.03%)	170082.73 (50749.31 to 348111.69)	785.46 (206.61 to 1680.95)	0.00% (-0.01% to 0.02%)
Portugal	25883.46 (5420.87 to 55524.75)	487.62 (83.55 to 1070.99)	0.01% (-0.05% to 0.06%)	54391.84 (12758.08 to 111585.95)	954.76 (184.12 to 2034.90)	0.00% (-0.03% to 0.03%)
Republic of Moldova	8454.21 (3330.41 to 16556.63)	422.67 (150.16 to 851.31)	0.00% (-0.04% to 0.05%)	17261.92 (6313.62 to 33503.43)	800.19 (266.08 to 1581.48)	0.00% (-0.03% to 0.04%)
Romania	44378.56 (14592.60 to 88512.92)	432.04 (127.38 to 877.21)	0.01% (-0.04% to 0.06%)	78636.56 (23554.33 to 164607.17)	751.34 (194.86 to 1617.96)	0.00% (-0.04% to 0.05%)
Russian Federation	373515.26 (147654.74 to 738518.28)	490.59 (182.01 to 1001.08)	0.01% (-0.01% to 0.02%)	753629.55 (298331.56 to 1446183.65)	875.09 (321.87 to 1741.34)	0.00% (-0.01% to 0.01%)
San Marino	80.08 (16.86 to 171.95)	489.26 (85.66 to 1078.25)	0.00% (-0.05% to 0.05%)	168.48 (38.73 to 346.51)	957.81 (183.29 to 2050.16)	-0.01% (-0.05% to 0.03%)
Serbia	21712.93 (6921.98 to 43923.53)	433.01 (126.43 to 900.79)	0.00% (-0.05% to 0.05%)	36900.12 (10740.76 to 77968.46)	750.37 (192.08 to 1600.07)	0.00% (-0.04% to 0.03%)

Slovakia	12950.31 (4148.10 to 25860.49)	431.78 (125.73 to 882.03)	0.01% (-0.05% to 0.06%)	22895.21 (6671.21 to 48873.55)	750.44 (193.99 to 1622.29)	0.00% (-0.04% to 0.04%)
Slovenia	4962.65 (1656.48 to 9790.50)	431.94 (126.39 to 880.93)	0.01% (-0.04% to 0.05%)	8340.18 (2454.28 to 17535.55)	750.26 (190.22 to 1625.18)	0.00% (-0.04% to 0.04%)
Spain	120492.37 (25368.92 to 260200.62)	507.44 (90.49 to 1122.00)	0.01% (-0.04% to 0.10%)	246689.17 (63992.83 to 497984.29)	1017.76 (216.78 to 2118.58)	0.00% (-0.06% to 0.05%)
Sweden	25969.88 (5265.00 to 54912.98)	483.47 (84.62 to 1037.44)	0.01% (-0.06% to 0.08%)	50475.24 (10255.52 to 106515.41)	981.10 (177.44 to 2084.33)	0.00% (-0.05% to 0.06%)
Switzerland	21074.77 (4923.31 to 44633.94)	445.27 (87.44 to 975.21)	0.02% (-0.05% to 0.10%)	40439.94 (9896.36 to 82678.81)	876.05 (186.46 to 1831.12)	0.01% (-0.05% to 0.07%)
Ukraine	103319.75 (40823.70 to 200958.67)	443.08 (164.50 to 890.88)	0.00% (-0.04% to 0.04%)	216629.76 (81765.09 to 416710.72)	825.76 (272.08 to 1658.70)	0.00% (-0.03% to 0.03%)
United Kingdom	167910.69 (36446.04 to 357634.14)	483.12 (93.82 to 1047.05)	0.01% (-0.04% to 0.05%)	339324.26 (77760.69 to 711127.93)	953.23 (191.24 to 2021.20)	0.00% (-0.04% to 0.04%)

Table S3c: Counts and age-standardized incidence rates of headache disorders in 2021, and percentage changes from 1990 to 2021, by country and sex

	**Male**			**Female**		
	**Absolute number, 2021**	**Age-standardized rate, per 100 000 people, 2021**	**Percentage Change, 1990-2021**	**Absolute number, 2021**	**Age-standardized rate, per 100 000 people, 2021**	**Percentage Change, 1990-2021**
**Europe**	**48555908.52 (42852324.20 to 53951770.13)**	**11479.08 (10093.46 to 12782.11)**	**-0.01% (-0.01% to 0.00%)**	**55736928.71 (49546328.77 to 61419006.97)**	**12662.55 (11215.82 to 14029.50)**	**-0.01% (-0.01% to 0.00%)**
Albania	152513.73 (134073.31 to 170641.68)	10965.85 (9581.84 to 12335.29)	0.00% (-0.01% to 0.01%)	167964.28 (147359.76 to 186928.59)	12372.46 (10856.90 to 13777.05)	0.00% (0.00% to 0.01%)
Andorra	5383.07 (4683.85 to 6078.60)	11673.71 (10192.20 to 13164.88)	0.00% (0.00% to 0.01%)	5632.78 (4974.16 to 6301.47)	13072.40 (11432.96 to 14635.12)	0.00% (0.00% to 0.00%)
Austria	526934.48 (463367.24 to 592004.32)	11620.90 (10086.06 to 13115.56)	0.00% (0.00% to 0.01%)	588472.45 (523471.10 to 652878.82)	12898.94 (11439.36 to 14394.55)	0.00% (0.00% to 0.01%)
Belarus	505414.59 (444106.98 to 566877.66)	11257.71 (9873.99 to 12679.75)	0.00% (-0.01% to 0.01%)	600239.59 (531079.04 to 669303.17)	11983.54 (10515.36 to 13353.38)	0.00% (0.00% to 0.01%)
Belgium	670812.68 (592315.17 to 747766.22)	11787.73 (10317.57 to 13288.52)	0.00% (-0.01% to 0.01%)	762458.96 (681816.60 to 847940.35)	13232.60 (11579.62 to 14824.87)	0.00% (-0.01% to 0.01%)
Bosnia and Herzegovina	182794.68 (160974.84 to 204904.97)	10965.74 (9599.48 to 12340.88)	0.00% (-0.01% to 0.00%)	211889.85 (186487.46 to 233821.41)	12372.32 (10849.70 to 13782.34)	0.00% (0.00% to 0.00%)
Bulgaria	370042.05 (326368.58 to 414540.37)	10965.91 (9604.32 to 12317.95)	0.00% (0.00% to 0.01%)	433662.36 (382168.98 to 481210.63)	12372.95 (10841.85 to 13788.24)	0.00% (-0.01% to 0.01%)
Croatia	230348.41 (203599.11 to 256326.48)	10956.68 (9627.82 to 12282.60)	0.00% (-0.04% to 0.03%)	270914.30 (241926.73 to 300342.70)	12417.31 (10943.39 to 13838.61)	0.00% (-0.04% to 0.04%)
Cyprus	81140.73 (70237.75 to 91742.42)	11673.95 (10184.74 to 13159.17)	0.00% (0.00% to 0.01%)	92750.41 (81613.64 to 103708.75)	13072.45 (11413.63 to 14685.83)	0.00% (0.00% to 0.00%)

Czechia	584603.22 (515653.87 to 654052.20)	10965.92 (9592.39 to 12315.47)	0.00% (-0.01% to 0.01%)	667748.29 (591628.37 to 739917.54)	12372.56 (10860.96 to 13780.47)	0.00% (0.00% to 0.00%)
Denmark	342098.86 (300377.34 to 381161.57)	11600.54 (10075.99 to 13019.76)	0.00% (-0.04% to 0.04%)	382423.34 (334085.02 to 426778.90)	13056.85 (11375.84 to 14648.32)	0.01% (-0.04% to 0.06%)
Estonia	71522.20 (62955.07 to 79955.35)	11258.07 (9865.99 to 12627.03)	0.00% (-0.01% to 0.01%)	82338.80 (73179.45 to 91251.72)	11983.44 (10485.40 to 13359.58)	0.00% (0.00% to 0.00%)
Finland	322391.34 (282430.09 to 359154.91)	11673.79 (10153.30 to 13193.54)	0.00% (-0.01% to 0.00%)	362286.01 (324176.76 to 402672.26)	13072.72 (11431.50 to 14653.47)	0.00% (0.00% to 0.00%)
France	3759212.99 (3331766.41 to 4186405.82)	11649.75 (10213.33 to 13089.52)	0.00% (-0.01% to 0.01%)	4350694.88 (3871100.83 to 4786643.86)	12850.77 (11337.48 to 14420.30)	0.00% (-0.01% to 0.01%)
Germany	5030643.80 (4414066.98 to 5613294.32)	11730.08 (10238.58 to 13253.66)	0.01% (-0.04% to 0.05%)	5522812.86 (4897418.38 to 6243975.41)	13183.15 (11599.98 to 14868.32)	0.00% (-0.04% to 0.05%)
Greece	582220.98 (510845.31 to 651178.04)	11713.04 (10217.85 to 13211.61)	0.00% (-0.01% to 0.01%)	678342.93 (603780.46 to 754441.38)	13089.29 (11441.01 to 14668.72)	0.00% (-0.01% to 0.01%)
Hungary	516779.99 (456529.46 to 579768.66)	10965.76 (9595.83 to 12331.91)	0.00% (-0.01% to 0.01%)	624647.15 (552445.48 to 688736.23)	12372.69 (10861.64 to 13791.97)	0.00% (0.00% to 0.01%)
Iceland	21111.94 (18635.24 to 23716.77)	11673.90 (10187.77 to 13166.98)	0.00% (-0.01% to 0.01%)	22748.26 (20163.37 to 25361.71)	13072.13 (11408.44 to 14673.05)	0.00% (0.00% to 0.01%)
Ireland	288953.71 (255462.33 to 323236.68)	11673.58 (10195.65 to 13151.86)	0.00% (0.00% to 0.00%)	330238.74 (293091.41 to 369148.15)	13072.44 (11430.78 to 14674.90)	0.00% (0.00% to 0.00%)
Israel	551170.96 (482384.96 to 619750.75)	11673.49 (10197.50 to 13163.59)	0.00% (-0.01% to 0.01%)	619757.45 (546269.55 to 690501.83)	13072.37 (11415.22 to 14647.06)	0.00% (0.00% to 0.01%)
Italy	3733829.62 (3300733.25 to 4152523.20)	12692.38 (11132.10 to 14151.36)	0.00% (-0.03% to 0.03%)	4269817.07 (3785044.38 to 4684980.82)	14128.78 (12578.02 to 15576.18)	0.00% (-0.03% to 0.03%)
Latvia	98958.84 (87213.39 to 110774.65)	11258.01 (9868.88 to 12655.13)	0.00% (0.00% to 0.01%)	119299.14 (105920.04 to 132039.28)	11983.45 (10483.20 to 13340.36)	0.00% (0.00% to 0.00%)

Lithuania	146473.66 (127854.94 to 165041.84)	11372.11 (9826.63 to 12858.10)	0.00% (-0.01% to 0.01%)	176279.79 (157063.14 to 195201.22)	12108.20 (10565.99 to 13560.98)	0.00% (-0.01% to 0.00%)
Luxembourg	39257.74 (34277.57 to 44592.52)	11678.91 (10193.95 to 13124.62)	0.00% (-0.01% to 0.01%)	42446.61 (37214.65 to 47289.89)	12951.16 (11373.68 to 14499.40)	0.00% (0.00% to 0.00%)
Malta	25961.95 (22717.75 to 29113.83)	11673.53 (10211.02 to 13168.60)	0.00% (-0.01% to 0.01%)	28508.59 (25513.09 to 31788.76)	13072.21 (11426.80 to 14659.61)	0.00% (0.00% to 0.00%)
Monaco	2113.26 (1864.56 to 2366.36)	11673.80 (10184.91 to 13156.65)	0.00% (-0.01% to 0.01%)	2469.93 (2202.21 to 2750.02)	13072.12 (11411.45 to 14667.32)	0.00% (-0.01% to 0.01%)
Montenegro	34465.00 (30351.24 to 38748.14)	10965.83 (9593.60 to 12316.84)	0.00% (0.00% to 0.01%)	39340.21 (34696.29 to 43602.24)	12372.37 (10855.02 to 13801.69)	0.00% (-0.01% to 0.00%)
Netherlands	1013957.22 (894733.43 to 1140552.94)	11784.90 (10190.55 to 13379.47)	0.00% (0.00% to 0.01%)	1135177.58 (998979.71 to 1272876.80)	13158.74 (11337.87 to 14902.33)	0.00% (0.00% to 0.01%)
North Macedonia	127221.61 (111986.23 to 143555.66)	10965.82 (9593.80 to 12338.92)	0.00% (0.00% to 0.01%)	137476.95 (120957.37 to 153286.47)	12372.45 (10857.78 to 13791.10)	0.00% (0.00% to 0.00%)
Norway	357294.83 (318629.91 to 398868.00)	12797.15 (11400.47 to 14283.16)	0.02% (-0.01% to 0.06%)	391906.34 (347140.35 to 434680.92)	14544.81 (12855.64 to 16188.84)	0.01% (-0.03% to 0.05%)
Poland	2250573.56 (1983619.97 to 2497809.85)	11737.26 (10396.00 to 13118.85)	0.00% (0.00% to 0.00%)	2658108.11 (2347869.56 to 2928576.05)	13259.29 (11648.10 to 14702.16)	0.00% (0.00% to 0.00%)
Portugal	594452.56 (520810.83 to 664156.80)	11673.62 (10216.84 to 13153.19)	0.00% (0.00% to 0.01%)	722571.21 (644900.06 to 804569.14)	13072.59 (11431.34 to 14667.64)	0.00% (0.00% to 0.00%)
Republic of Moldova	203423.65 (178213.48 to 228659.16)	11257.85 (9856.54 to 12693.15)	0.00% (-0.01% to 0.01%)	232080.45 (205529.25 to 260542.10)	11983.25 (10483.06 to 13340.81)	0.00% (-0.01% to 0.01%)
Romania	1036670.46 (916271.61 to 1161052.05)	10965.97 (9586.76 to 12322.54)	0.00% (0.00% to 0.01%)	1210222.68 (1066332.77 to 1335474.67)	12372.52 (10842.49 to 13800.53)	0.00% (-0.01% to 0.00%)
Russian Federation	8245557.17 (7243008.89 to 9178026.93)	11855.44 (10423.24 to 13186.33)	0.00% (-0.04% to 0.04%)	9898485.31 (8763046.93 to 10936173.18)	12710.13 (11290.49 to 14156.67)	0.01% (-0.03% to 0.05%)

San Marino	1863.32 (1642.80 to 2080.83)	11673.79 (10195.94 to 13164.93)	0.00% (-0.01% to 0.01%)	2219.90 (1979.97 to 2469.48)	13072.15 (11405.85 to 14655.12)	0.00% (0.00% to 0.00%)
Serbia	512565.68 (451931.20 to 575188.10)	10965.89 (9587.44 to 12320.69)	0.00% (-0.01% to 0.01%)	568288.11 (502658.70 to 628454.97)	12372.47 (10863.74 to 13793.97)	0.00% (0.00% to 0.01%)
Slovakia	300677.23 (265157.48 to 338509.09)	10965.95 (9583.46 to 12322.18)	0.00% (0.00% to 0.01%)	348486.71 (307319.82 to 386145.38)	12372.47 (10845.02 to 13759.49)	0.00% (0.00% to 0.01%)
Slovenia	115021.51 (101128.38 to 128552.39)	10965.67 (9593.15 to 12327.42)	0.00% (-0.01% to 0.01%)	128639.48 (114006.71 to 141787.58)	12372.83 (10848.17 to 13790.17)	0.00% (-0.01% to 0.01%)
Spain	2648605.88 (2314838.64 to 2969417.46)	11688.64 (10131.10 to 13111.03)	0.00% (-0.01% to 0.01%)	3000700.55 (2675883.87 to 3327979.37)	12898.11 (11434.63 to 14372.02)	0.00% (0.00% to 0.00%)
Sweden	653777.52 (578316.80 to 717960.22)	12444.97 (11004.09 to 13844.36)	0.01% (-0.03% to 0.04%)	730913.49 (653549.70 to 806839.25)	14334.09 (12768.93 to 16000.48)	0.00% (-0.03% to 0.04%)
Switzerland	514306.65 (449773.44 to 574595.17)	11310.97 (9988.18 to 12614.16)	0.01% (-0.03% to 0.06%)	570561.16 (504512.84 to 630655.44)	12711.90 (11158.47 to 14072.35)	0.01% (-0.03% to 0.05%)
Ukraine	2538686.48 (2235101.19 to 2845038.43)	12085.71 (10652.93 to 13488.62)	0.00% (0.00% to 0.01%)	3022001.09 (2674049.88 to 3358320.07)	12813.24 (11305.27 to 14287.16)	0.00% (0.00% to 0.00%)
United Kingdom	4195329.42 (3724031.58 to 4656670.02)	12444.29 (10985.71 to 13892.51)	0.00% (0.00% to 0.00%)	4849796.79 (4318090.05 to 5357625.65)	13947.46 (12381.56 to 15446.27)	0.00% (0.00% to 0.01%)

Table S3d: Counts and age-standardized YLD rates of headache disorders in 2021, and percentage changes from 1990 to 2021, by country and sex

	Male			Female		
	**Absolute number, 2021**	**Age-standardized rate, per 100 000 people, 2021**	**Percentage Change, 1990-2021**	**Absolute number, 2021**	**Age-standardized rate, per 100 000 people, 2021**	**Percentage Change, 1990-2021**
**Europe**	**2156683.04 (610227.14 to 4441134.25)**	**483.36 (120.11 to 1021.87)**	**0.01% (-0.01% to 0.03%)**	**4188380.05 (1191901.20 to 8451079.17)**	**908.18 (223.50 to 1866.52)**	**0.01% (-0.02% to 0.03%)**
Albania	6370.36 (2016.95 to 12857.39)	431.91 (124.11 to 886.67)	0.00% (-0.04% to 0.05%)	10953.67 (3062.68 to 23179.72)	750.32 (194.01 to 1613.90)	0.00% (-0.04% to 0.03%)
Andorra	241.91 (50.95 to 519.53)	489.24 (86.07 to 1076.87)	0.00% (-0.05% to 0.05%)	442.21 (102.11 to 915.54)	958.55 (185.68 to 2053.42)	0.00% (-0.04% to 0.03%)
Austria	22315.45 (5453.43 to 47835.01)	469.87 (98.02 to 1033.84)	0.00% (-0.07% to 0.06%)	42848.78 (10379.57 to 87853.27)	910.67 (186.47 to 1925.91)	0.00% (-0.04% to 0.04%)
Belarus	20850.31 (8145.28 to 40680.20)	421.65 (149.25 to 850.44)	0.00% (-0.05% to 0.05%)	44186.63 (16238.71 to 85779.46)	798.55 (262.61 to 1606.64)	0.00% (-0.04% to 0.03%)
Belgium	33742.99 (5848.17 to 72433.05)	575.67 (85.23 to 1269.70)	0.03% (-0.07% to 0.12%)	69266.67 (12490.45 to 146826.94)	1166.28 (185.12 to 2528.78)	0.08% (-0.03% to 0.18%)
Bosnia and Herzegovina	7817.58 (2573.11 to 15554.73)	430.04 (123.89 to 890.30)	0.00% (-0.05% to 0.06%)	13864.93 (4050.40 to 29021.25)	746.95 (193.03 to 1610.11)	0.00% (-0.04% to 0.04%)
Bulgaria	15924.73 (5165.18 to 31875.74)	430.56 (122.90 to 892.84)	0.00% (-0.04% to 0.05%)	28199.19 (8349.48 to 58779.52)	747.64 (192.01 to 1603.14)	0.00% (-0.04% to 0.03%)
Croatia	9866.88 (3231.66 to 19757.21)	430.47 (125.14 to 887.58)	0.00% (-0.06% to 0.07%)	17534.41 (5132.77 to 36425.16)	750.60 (193.44 to 1626.08)	0.00% (-0.05% to 0.06%)
Cyprus	3578.44 (728.20 to 7719.10)	490.73 (88.25 to 1082.27)	0.00% (-0.06% to 0.06%)	7264.48 (1573.34 to 15031.96)	960.26 (183.55 to 2052.65)	0.00% (-0.04% to 0.03%)
Czechia	25017.40 (8263.60 to 49788.50)	430.94 (125.69 to 889.86)	0.00% (-0.04% to 0.05%)	43133.67 (12354.18 to 90639.44)	748.37 (190.88 to 1628.33)	0.00% (-0.04% to 0.04%)

Denmark	13524.04 (3306.92 to 28831.59)	445.32 (93.96 to 969.36)	0.02% (-0.05% to 0.10%)	25675.53 (5828.86 to 53318.55)	863.67 (170.89 to 1820.81)	0.02% (-0.05% to 0.10%)
Estonia	2940.07 (1157.74 to 5755.00)	422.10 (150.58 to 852.54)	0.01% (-0.04% to 0.06%)	5886.78 (2202.68 to 11427.82)	800.29 (265.57 to 1598.27)	0.00% (-0.03% to 0.03%)
Finland	13769.28 (2825.38 to 29427.60)	487.86 (86.78 to 1073.33)	0.00% (-0.05% to 0.06%)	26579.26 (5975.21 to 54488.41)	959.39 (184.19 to 2035.43)	0.00% (-0.04% to 0.04%)
France	171067.16 (38864.67 to 362011.25)	516.78 (101.13 to 1110.26)	-0.01% (-0.08% to 0.07%)	326156.60 (84679.31 to 657207.83)	951.73 (216.88 to 1950.88)	-0.01% (-0.07% to 0.06%)
Germany	231513.59 (49952.83 to 488124.83)	519.55 (96.00 to 1110.27)	0.03% (-0.05% to 0.11%)	455840.62 (105101.56 to 925890.88)	1056.88 (196.66 to 2225.78)	0.02% (-0.05% to 0.10%)
Greece	26385.85 (5314.50 to 57855.17)	511.88 (85.80 to 1147.56)	-0.01% (-0.08% to 0.06%)	51872.16 (11669.93 to 106816.74)	974.19 (182.76 to 2078.22)	0.00% (-0.07% to 0.06%)
Hungary	22259.54 (7356.73 to 44486.06)	432.01 (127.54 to 905.19)	0.01% (-0.04% to 0.06%)	40668.51 (12096.93 to 85032.66)	749.06 (195.03 to 1618.28)	0.00% (-0.04% to 0.05%)
Iceland	912.46 (176.61 to 1956.59)	490.85 (86.42 to 1084.95)	0.00% (-0.05% to 0.05%)	1709.34 (367.22 to 3579.01)	962.07 (186.91 to 2055.02)	0.00% (-0.04% to 0.04%)
Ireland	12408.68 (2454.33 to 27048.04)	487.86 (83.73 to 1074.14)	0.00% (-0.06% to 0.05%)	24789.04 (5401.03 to 51149.51)	955.72 (183.76 to 2041.89)	0.00% (-0.04% to 0.03%)
Israel	23134.32 (4213.83 to 51247.55)	491.42 (86.90 to 1094.13)	0.00% (-0.05% to 0.05%)	45309.50 (8942.59 to 96078.05)	963.01 (183.39 to 2071.68)	0.00% (-0.04% to 0.03%)
Italy	163472.55 (33294.01 to 350580.00)	532.62 (89.26 to 1172.86)	0.01% (-0.04% to 0.06%)	325272.52 (69737.49 to 678546.36)	1049.23 (181.47 to 2232.96)	0.02% (-0.04% to 0.06%)
Latvia	4088.12 (1615.91 to 7883.34)	421.56 (149.85 to 844.53)	0.01% (-0.04% to 0.06%)	8624.94 (3244.73 to 16692.39)	800.04 (266.40 to 1590.50)	0.00% (-0.03% to 0.04%)
Lithuania	5367.77 (2047.75 to 10550.65)	377.19 (132.14 to 761.61)	0.01% (-0.05% to 0.05%)	11835.40 (4134.77 to 23161.69)	752.52 (229.49 to 1528.20)	0.00% (-0.04% to 0.03%)

Luxembourg	1773.69 (415.55 to 3736.14)	497.39 (103.85 to 1064.70)	0.01% (-0.04% to 0.06%)	3269.10 (831.51 to 6579.89)	944.96 (215.19 to 1929.69)	0.00% (-0.03% to 0.03%)
Malta	1131.17 (247.14 to 2435.76)	490.02 (88.35 to 1087.21)	0.00% (-0.05% to 0.06%)	2142.87 (482.01 to 4411.82)	960.48 (181.64 to 2045.71)	0.00% (-0.03% to 0.03%)
Monaco	90.96 (20.53 to 191.79)	489.59 (87.80 to 1092.18)	0.00% (-0.05% to 0.05%)	183.19 (43.29 to 374.59)	957.44 (183.64 to 2036.52)	-0.01% (-0.04% to 0.03%)
Montenegro	1452.64 (464.57 to 2961.25)	432.13 (124.96 to 894.11)	0.00% (-0.04% to 0.06%)	2551.12 (722.61 to 5432.53)	747.55 (189.86 to 1619.27)	-0.01% (-0.04% to 0.03%)
Netherlands	41406.73 (8074.70 to 92831.79)	468.09 (78.36 to 1070.25)	0.07% (-0.05% to 0.19%)	81505.26 (16294.50 to 171216.98)	928.70 (157.97 to 1981.10)	0.04% (-0.04% to 0.11%)
North Macedonia	5484.49 (1752.60 to 11053.89)	431.69 (124.56 to 887.98)	0.00% (-0.05% to 0.05%)	9090.58 (2547.79 to 19218.16)	746.84 (191.54 to 1611.94)	0.00% (-0.04% to 0.03%)
Norway	14390.67 (2856.02 to 31524.09)	499.29 (87.89 to 1109.11)	0.09% (0.02% to 0.16%)	26422.71 (5385.36 to 55234.48)	961.46 (170.20 to 2049.84)	0.07% (0.00% to 0.12%)
Poland	95963.54 (33133.53 to 192959.82)	457.39 (137.89 to 955.96)	0.01% (-0.02% to 0.03%)	170082.73 (50749.31 to 348111.69)	785.46 (206.61 to 1680.95)	0.00% (-0.01% to 0.02%)
Portugal	25883.46 (5420.87 to 55524.75)	487.62 (83.55 to 1070.99)	0.01% (-0.05% to 0.06%)	54391.84 (12758.08 to 111585.95)	954.76 (184.12 to 2034.90)	0.00% (-0.03% to 0.03%)
Republic of Moldova	8454.21 (3330.41 to 16556.63)	422.67 (150.16 to 851.31)	0.00% (-0.04% to 0.05%)	17261.92 (6313.62 to 33503.43)	800.19 (266.08 to 1581.48)	0.00% (-0.03% to 0.04%)
Romania	44378.56 (14592.60 to 88512.92)	432.04 (127.38 to 877.21)	0.01% (-0.04% to 0.06%)	78636.56 (23554.33 to 164607.17)	751.34 (194.86 to 1617.96)	0.00% (-0.04% to 0.05%)
Russian Federation	373515.26 (147654.74 to 738518.28)	490.59 (182.01 to 1001.08)	0.01% (-0.01% to 0.02%)	753629.55 (298331.56 to 1446183.65)	875.09 (321.87 to 1741.34)	0.00% (-0.01% to 0.01%)
San Marino	80.08 (16.86 to 171.95)	489.26 (85.66 to 1078.25)	0.00% (-0.05% to 0.05%)	168.48 (38.73 to 346.51)	957.81 (183.29 to 2050.16)	-0.01% (-0.05% to 0.03%)
Serbia	21712.93 (6921.98 to 43923.53)	433.01 (126.43 to 900.79)	0.00% (-0.05% to 0.05%)	36900.12 (10740.76 to 77968.46)	750.37 (192.08 to 1600.07)	0.00% (-0.04% to 0.03%)

Slovakia	12950.31 (4148.10 to 25860.49)	431.78 (125.73 to 882.03)	0.01% (-0.05% to 0.06%)	22895.21 (6671.21 to 48873.55)	750.44 (193.99 to 1622.29)	0.00% (-0.04% to 0.04%)
Slovenia	4962.65 (1656.48 to 9790.50)	431.94 (126.39 to 880.93)	0.01% (-0.04% to 0.05%)	8340.18 (2454.28 to 17535.55)	750.26 (190.22 to 1625.18)	0.00% (-0.04% to 0.04%)
Spain	120492.37 (25368.92 to 260200.62)	507.44 (90.49 to 1122.00)	0.01% (-0.04% to 0.10%)	246689.17 (63992.83 to 497984.29)	1017.76 (216.78 to 2118.58)	0.00% (-0.06% to 0.05%)
Sweden	25969.88 (5265.00 to 54912.98)	483.47 (84.62 to 1037.44)	0.01% (-0.06% to 0.08%)	50475.24 (10255.52 to 106515.41)	981.10 (177.44 to 2084.33)	0.00% (-0.05% to 0.06%)
Switzerland	21074.77 (4923.31 to 44633.94)	445.27 (87.44 to 975.21)	0.02% (-0.05% to 0.10%)	40439.94 (9896.36 to 82678.81)	876.05 (186.46 to 1831.12)	0.01% (-0.05% to 0.07%)
Ukraine	103319.75 (40823.70 to 200958.67)	443.08 (164.50 to 890.88)	0.00% (-0.04% to 0.04%)	216629.76 (81765.09 to 416710.72)	825.76 (272.08 to 1658.70)	0.00% (-0.03% to 0.03%)
United Kingdom	167910.69 (36446.04 to 357634.14)	483.12 (93.82 to 1047.05)	0.01% (-0.04% to 0.05%)	339324.26 (77760.69 to 711127.93)	953.23 (191.24 to 2021.20)	0.00% (-0.04% to 0.04%)

Table S3e: Counts and age-standardized prevalence rates of migraine in 2021, and percentage changes from 1990 to 2021, by country and sex

	Male			Female		
	**Absolute number, 2021**	**Age-standardized rate, per 100 000 people, 2021**	**Percentage Change, 1990-2021**	**Absolute number, 2021**	**Age-standardized rate, per 100 000 people, 2021**	**Percentage Change, 1990-2021**
**Europe**	**47804150.91 (41171341.16 to 55104465.37)**	**10952.93 (9377.99 to 12681.88)**	**0.01% (0.00% to 0.03%)**	**95738019.23 (83608895.84 to 110149171.39)**	**21302.28 (18481.83 to 24506.50)**	**0.01% (0.00% to 0.03%)**
Albania	135864.69 (115232.75 to 158518.40)	9382.99 (7841.02 to 11025.35)	0.00% (0.00% to 0.00%)	256548.41 (220171.72 to 297051.82)	17916.74 (15246.53 to 20788.15)	0.00% (0.00% to 0.00%)
Andorra	5681.74 (4771.11 to 6729.07)	11791.08 (9929.64 to 13884.18)	0.00% (0.00% to 0.00%)	10501.06 (9013.06 to 12317.09)	23391.94 (19912.95 to 27680.34)	0.00% (0.00% to 0.00%)
Austria	512129.25 (438573.59 to 597328.23)	11049.39 (9355.28 to 12900.19)	0.00% (-0.01% to 0.00%)	1010553.72 (873611.02 to 1168780.23)	22064.97 (18789.78 to 25685.47)	0.00% (0.00% to 0.00%)
Belarus	408677.83 (350058.66 to 477373.54)	8529.51 (7257.76 to 9988.76)	0.00% (0.00% to 0.00%)	922787.21 (798738.28 to 1057180.97)	17309.81 (14881.07 to 20168.02)	0.00% (0.00% to 0.00%)
Belgium	824233.96 (702107.95 to 989858.66)	14264.66 (12009.21 to 17275.82)	0.04% (-0.03% to 0.13%)	1720374.41 (1481636.54 to 1990760.87)	29301.08 (25241.15 to 34155.34)	0.09% (0.00% to 0.19%)
Bosnia and Herzegovina	166072.15 (141700.29 to 193271.04)	9382.99 (7841.02 to 11025.35)	0.00% (0.00% to 0.00%)	324983.47 (278121.00 to 377983.37)	17916.74 (15246.53 to 20788.15)	0.00% (0.00% to 0.00%)
Bulgaria	337003.34 (289261.73 to 391036.48)	9382.99 (7841.02 to 11025.35)	0.00% (0.00% to 0.00%)	657942.02 (565341.51 to 765669.63)	17916.74 (15246.53 to 20788.15)	0.00% (0.00% to 0.00%)
Croatia	208206.12 (179327.72 to 242414.95)	9332.48 (7969.67 to 10997.50)	-0.01% (-0.06% to 0.05%)	407649.08 (350224.94 to 474154.46)	17894.26 (15212.37 to 20813.77)	0.00% (-0.06% to 0.05%)
Cyprus	84930.17 (71958.20 to 100902.17)	11791.08 (9929.64 to 13884.18)	0.00% (0.00% to 0.00%)	173697.43 (149425.96 to 204561.75)	23391.94 (19912.95 to 27680.34)	0.00% (0.00% to 0.00%)

Czechia	529675.79 (453450.98 to 614207.35)	9382.99 (7841.02 to 11025.35)	0.00% (0.00% to 0.00%)	1010131.35 (866422.01 to 1173184.20)	17916.74 (15246.53 to 20788.15)	0.00% (0.00% to 0.00%)
Denmark	306871.79 (260694.92 to 360457.95)	10342.14 (8688.50 to 12161.75)	0.03% (-0.04% to 0.09%)	599455.47 (518607.47 to 692934.87)	20656.93 (17587.22 to 24148.60)	0.03% (-0.03% to 0.11%)
Estonia	57555.80 (49556.78 to 67184.17)	8529.51 (7257.76 to 9988.76)	0.00% (0.00% to 0.00%)	122907.95 (106428.39 to 140431.01)	17309.81 (14881.07 to 20168.02)	0.00% (0.00% to 0.00%)
Finland	326204.56 (277114.90 to 383170.10)	11791.08 (9929.64 to 13884.18)	0.00% (0.00% to 0.00%)	632833.25 (545081.46 to 739422.59)	23391.94 (19912.95 to 27680.34)	0.00% (0.00% to 0.00%)
France	3965737.24 (3361196.24 to 4628060.63)	12238.19 (10241.73 to 14345.00)	-0.01% (-0.08% to 0.05%)	7542864.44 (6508733.29 to 8750271.28)	22645.95 (19412.66 to 26401.27)	-0.01% (-0.07% to 0.05%)
Germany	5521918.23 (4678965.41 to 6508061.29)	12667.06 (10674.82 to 15123.07)	0.03% (-0.03% to 0.10%)	11017404.14 (9524910.89 to 12856159.75)	26193.30 (22622.02 to 30688.11)	0.03% (-0.03% to 0.11%)
Greece	630737.62 (535523.86 to 743268.75)	12525.37 (10461.78 to 14948.73)	-0.01% (-0.07% to 0.06%)	1237763.01 (1055878.58 to 1436640.58)	23931.26 (20568.89 to 28186.22)	0.00% (-0.05% to 0.06%)
Hungary	470735.96 (401899.83 to 546406.66)	9382.99 (7841.02 to 11025.35)	0.00% (0.00% to 0.00%)	949422.87 (813902.78 to 1104305.83)	17916.74 (15246.53 to 20788.15)	0.00% (0.00% to 0.00%)
Iceland	21635.05 (18399.42 to 25410.78)	11791.08 (9929.64 to 13884.18)	0.00% (0.00% to 0.00%)	40939.09 (35145.88 to 48181.95)	23391.94 (19912.95 to 27680.34)	0.00% (0.00% to 0.00%)
Ireland	294996.81 (249802.71 to 346396.33)	11791.08 (9929.64 to 13884.18)	0.00% (0.00% to 0.00%)	595682.55 (512032.27 to 697561.08)	23391.94 (19912.95 to 27680.34)	0.00% (0.00% to 0.00%)
Israel	552843.86 (466451.14 to 651306.02)	11791.08 (9929.64 to 13884.18)	0.00% (0.00% to 0.00%)	1093227.59 (934107.43 to 1287811.11)	23391.94 (19912.95 to 27680.34)	0.00% (0.00% to 0.00%)
Italy	3865538.29 (3319200.11 to 4525121.96)	12883.94 (10989.60 to 14842.80)	0.01% (-0.03% to 0.06%)	7771391.44 (6802050.95 to 8924649.40)	25735.51 (22511.73 to 29431.95)	0.01% (-0.03% to 0.05%)
Latvia	79895.06 (68987.69 to 93418.51)	8529.51 (7257.76 to 9988.76)	0.00% (0.00% to 0.00%)	179291.31 (155088.63 to 204636.13)	17309.81 (14881.07 to 20168.02)	0.00% (0.00% to 0.00%)

Lithuania	103729.85 (88619.07 to 119495.36)	7535.66 (6370.84 to 8761.53)	0.00% (0.00% to 0.00%)	255113.49 (219660.85 to 295250.03)	16804.43 (14306.02 to 19693.80)	0.00% (0.00% to 0.00%)
Luxembourg	40973.03 (34812.25 to 48016.52)	11729.48 (9973.86 to 13795.19)	0.00% (0.00% to 0.00%)	75318.65 (64920.43 to 88453.51)	22311.90 (19072.94 to 26166.25)	0.00% (0.00% to 0.00%)
Malta	26597.53 (22535.87 to 31313.75)	11791.08 (9929.64 to 13884.18)	0.00% (0.00% to 0.00%)	50841.93 (43654.97 to 59533.91)	23391.94 (19912.95 to 27680.34)	0.00% (0.00% to 0.00%)
Monaco	2119.07 (1789.63 to 2498.22)	11791.08 (9929.64 to 13884.18)	0.00% (0.00% to 0.00%)	4319.15 (3711.88 to 5035.21)	23391.94 (19912.95 to 27680.34)	0.00% (0.00% to 0.00%)
Montenegro	30922.20 (26242.02 to 36053.55)	9382.99 (7841.02 to 11025.35)	0.00% (0.00% to 0.00%)	60022.03 (51408.98 to 69520.81)	17916.74 (15246.53 to 20788.15)	0.00% (0.00% to 0.00%)
Netherlands	973852.01 (833152.88 to 1128630.00)	11233.51 (9459.19 to 13211.79)	0.09% (0.00% to 0.20%)	1971048.50 (1691110.41 to 2279026.97)	22889.17 (19534.78 to 26569.03)	0.05% (-0.01% to 0.12%)
North Macedonia	116625.47 (99090.43 to 135826.13)	9382.99 (7841.02 to 11025.35)	0.00% (0.00% to 0.00%)	214555.40 (183956.13 to 248385.06)	17916.74 (15246.53 to 20788.15)	0.00% (0.00% to 0.00%)
Norway	342606.67 (294632.29 to 397633.12)	12083.92 (10313.97 to 13968.21)	0.09% (0.05% to 0.15%)	637900.28 (556247.63 to 729979.87)	23619.18 (20437.57 to 27075.49)	0.07% (0.03% to 0.12%)
Poland	2032711.38 (1754665.86 to 2337514.62)	9916.82 (8528.22 to 11435.96)	0.00% (0.00% to 0.00%)	3940649.87 (3459035.66 to 4528063.65)	18613.09 (16139.42 to 21202.63)	0.00% (0.00% to 0.00%)
Portugal	610568.70 (516191.71 to 717880.06)	11791.08 (9929.64 to 13884.18)	0.00% (0.00% to 0.00%)	1292515.80 (1114093.09 to 1508696.83)	23391.94 (19912.95 to 27680.34)	0.00% (0.00% to 0.00%)
Republic of Moldova	165878.79 (142079.90 to 194761.16)	8529.51 (7257.76 to 9988.76)	0.00% (0.00% to 0.00%)	361508.37 (312572.09 to 415084.81)	17309.81 (14881.07 to 20168.02)	0.00% (0.00% to 0.00%)
Romania	938218.22 (800956.51 to 1090652.18)	9382.99 (7841.02 to 11025.35)	0.00% (0.00% to 0.00%)	1828542.58 (1568212.61 to 2125384.34)	17916.74 (15246.53 to 20788.15)	0.00% (0.00% to 0.00%)
Russian Federation	7426093.76 (6381957.63 to 8628091.03)	10028.71 (8569.88 to 11598.56)	0.00% (0.00% to 0.00%)	15372973.84 (13408971.01 to 17503422.36)	18486.73 (16076.60 to 21034.84)	0.00% (0.00% to 0.00%)

San Marino	1880.34 (1589.79 to 2208.58)	11791.08 (9929.64 to 13884.18)	0.00% (0.00% to 0.00%)	3997.50 (3441.89 to 4673.15)	23391.94 (19912.95 to 27680.34)	0.00% (0.00% to 0.00%)
Serbia	461169.68 (391122.12 to 537443.07)	9390.11 (7845.03 to 11048.33)	0.00% (0.00% to 0.00%)	863912.78 (745437.08 to 999607.94)	17920.66 (15230.56 to 20840.87)	0.00% (0.00% to 0.00%)
Slovakia	274716.15 (234110.15 to 319309.43)	9382.99 (7841.02 to 11025.35)	0.00% (0.00% to 0.00%)	535560.14 (458700.05 to 621200.53)	17916.74 (15246.53 to 20788.15)	0.00% (0.00% to 0.00%)
Slovenia	104434.88 (89848.32 to 121163.82)	9382.99 (7841.02 to 11025.35)	0.00% (0.00% to 0.00%)	194245.84 (166754.54 to 226182.35)	17916.74 (15246.53 to 20788.15)	0.00% (0.00% to 0.00%)
Spain	2849844.70 (2429896.86 to 3357801.62)	12294.82 (10416.21 to 14559.42)	0.00% (0.00% to 0.01%)	5688032.86 (4877275.32 to 6694068.94)	24288.78 (20947.42 to 28631.13)	0.00% (0.00% to 0.01%)
Sweden	614086.14 (533241.19 to 699782.68)	11627.66 (9967.06 to 13397.83)	0.01% (-0.05% to 0.07%)	1232618.19 (1075133.47 to 1409329.82)	24346.12 (21267.80 to 27911.26)	0.00% (-0.04% to 0.05%)
Switzerland	492200.35 (418023.91 to 574444.73)	10647.37 (8861.49 to 12500.01)	0.01% (-0.03% to 0.08%)	952616.85 (822701.34 to 1099136.22)	21201.67 (18152.09 to 24537.87)	0.01% (-0.04% to 0.06%)
Ukraine	2043637.59 (1763346.05 to 2339641.56)	9012.74 (7707.94 to 10364.12)	0.00% (0.00% to 0.00%)	4550514.83 (3985815.08 to 5176898.33)	17952.84 (15634.09 to 20439.68)	0.00% (0.00% to 0.00%)
United Kingdom	3947591.69 (3415573.64 to 4554397.26)	11571.82 (9875.84 to 13359.52)	0.01% (-0.04% to 0.06%)	8117178.78 (7088297.27 to 9415131.97)	23251.25 (20090.63 to 26808.28)	0.00% (-0.04% to 0.05%)

Table S3f: Counts and age-standardized DALY rates of migraine in 2021, and percentage changes from 1990 to 2021, by country and sex

	Male			Female		
	**Absolute number, 2021**	**Age-standardized rate, per 100 000 people, 2021**	**Percentage Change, 1990-2021**	**Absolute number, 2021**	**Age-standardized rate, per 100 000 people, 2021**	**Percentage Change, 1990-2021**
**Europe**	**1859028.36 (409180.70 to 3972660.88)**	**420.49 (81.01 to 930.36)**	**0.01% (-0.01% to 0.03%)**	**3718057.71 (824559.40 to 7770294.82)**	**815.91 (151.00 to 1739.13)**	**0.01% (-0.02% to 0.03%)**
Albania	5418.86 (1424.13 to 11543.52)	369.75 (87.33 to 805.44)	0.00% (-0.05% to 0.05%)	9653.43 (2133.16 to 21050.44)	666.44 (130.79 to 1501.37)	0.00% (-0.04% to 0.04%)
Andorra	211.91 (35.42 to 473.98)	433.55 (59.17 to 988.63)	0.00% (-0.06% to 0.06%)	399.05 (68.84 to 848.52)	875.21 (126.15 to 1922.32)	0.00% (-0.04% to 0.04%)
Austria	19296.15 (3742.42 to 43852.38)	410.80 (67.64 to 956.56)	0.00% (-0.06% to 0.06%)	38424.10 (7117.37 to 80266.90)	827.22 (125.95 to 1782.41)	0.00% (-0.04% to 0.05%)
Belarus	17202.86 (5514.54 to 36102.47)	350.44 (98.93 to 756.24)	0.00% (-0.05% to 0.05%)	37973.25 (11431.93 to 77840.45)	695.14 (181.04 to 1471.83)	0.00% (-0.04% to 0.03%)
Belgium	30216.94 (4047.17 to 67008.94)	520.07 (59.41 to 1159.94)	0.04% (-0.06% to 0.13%)	63797.92 (8812.30 to 143038.98)	1083.08 (125.37 to 2466.04)	0.08% (-0.04% to 0.20%)
Bosnia and Herzegovina	6629.91 (1764.56 to 14128.60)	368.05 (84.79 to 812.04)	0.00% (-0.05% to 0.06%)	12185.43 (2752.79 to 26670.29)	663.69 (130.29 to 1502.15)	-0.01% (-0.04% to 0.04%)
Bulgaria	13484.33 (3658.19 to 28927.95)	368.37 (85.20 to 810.50)	0.00% (-0.05% to 0.05%)	24732.94 (5650.78 to 53721.10)	664.18 (127.33 to 1500.17)	0.00% (-0.04% to 0.04%)
Croatia	8356.32 (2246.14 to 17455.42)	368.16 (86.56 to 801.60)	0.00% (-0.07% to 0.07%)	15387.87 (3502.10 to 33924.87)	666.82 (130.26 to 1502.15)	0.00% (-0.06% to 0.07%)
Cyprus	3152.56 (471.28 to 7215.94)	435.05 (58.41 to 1002.07)	0.00% (-0.05% to 0.07%)	6579.36 (1078.81 to 14085.57)	876.83 (126.31 to 1918.37)	0.00% (-0.04% to 0.03%)
Czechia	21198.92 (5597.92 to 45330.46)	368.76 (85.22 to 811.75)	0.00% (-0.05% to 0.06%)	37897.68 (8417.90 to 83289.48)	664.87 (127.05 to 1517.91)	0.00% (-0.04% to 0.04%)

Denmark	11658.53 (2170.29 to 26019.65)	388.17 (60.66 to 895.77)	0.03% (-0.05% to 0.11%)	22908.40 (3942.39 to 49148.67)	779.90 (114.24 to 1689.65)	0.03% (-0.05% to 0.10%)
Estonia	2423.28 (771.80 to 5064.63)	350.71 (98.90 to 762.74)	0.00% (-0.04% to 0.06%)	5053.57 (1528.82 to 10382.41)	696.55 (182.37 to 1477.68)	0.00% (-0.03% to 0.04%)
Finland	12060.45 (1911.18 to 26943.61)	432.34 (56.76 to 986.09)	0.00% (-0.05% to 0.06%)	23951.67 (4321.47 to 51422.39)	876.04 (125.05 to 1939.10)	0.00% (-0.04% to 0.04%)
France	149881.54 (26306.55 to 330810.56)	457.45 (69.26 to 1040.00)	-0.01% (-0.09% to 0.07%)	291519.25 (58169.25 to 604091.44)	861.81 (144.52 to 1826.00)	-0.01% (-0.07% to 0.07%)
Germany	203766.88 (34397.63 to 458687.39)	462.90 (63.18 to 1074.57)	0.03% (-0.06% to 0.12%)	411970.87 (70087.16 to 873933.68)	968.50 (138.04 to 2089.72)	0.02% (-0.06% to 0.10%)
Greece	23224.23 (3661.95 to 53112.46)	456.26 (59.23 to 1067.04)	-0.01% (-0.09% to 0.08%)	46761.71 (8138.33 to 100780.90)	890.95 (122.25 to 1978.71)	-0.01% (-0.08% to 0.07%)
Hungary	18888.42 (4962.58 to 40308.14)	369.86 (85.43 to 809.53)	0.01% (-0.05% to 0.07%)	35699.42 (8101.90 to 78199.98)	665.38 (129.81 to 1488.51)	0.00% (-0.04% to 0.05%)
Iceland	803.29 (119.74 to 1830.76)	435.08 (57.39 to 1002.07)	0.00% (-0.05% to 0.06%)	1549.19 (251.80 to 3341.26)	878.48 (127.83 to 1932.61)	0.00% (-0.04% to 0.04%)
Ireland	10914.14 (1661.93 to 24505.01)	432.28 (57.31 to 991.34)	0.00% (-0.06% to 0.05%)	22446.14 (3767.42 to 47864.64)	872.58 (127.06 to 1904.33)	0.00% (-0.04% to 0.03%)
Israel	20461.67 (2908.77 to 47162.36)	435.56 (59.60 to 1007.18)	0.00% (-0.06% to 0.05%)	41228.52 (6263.11 to 90236.41)	879.48 (124.91 to 1935.81)	0.00% (-0.04% to 0.04%)
Italy	143803.30 (22379.71 to 314245.48)	474.46 (58.87 to 1062.93)	0.01% (-0.05% to 0.06%)	294765.35 (47051.64 to 630401.09)	964.57 (119.79 to 2154.27)	0.02% (-0.04% to 0.07%)
Latvia	3367.67 (1105.00 to 6920.88)	350.44 (100.79 to 757.58)	0.01% (-0.04% to 0.07%)	7393.37 (2256.50 to 14891.16)	696.51 (180.66 to 1478.42)	0.00% (-0.04% to 0.04%)
Lithuania	4375.61 (1366.48 to 8965.66)	309.96 (85.57 to 659.07)	0.01% (-0.05% to 0.06%)	10209.14 (2879.02 to 21001.01)	658.58 (155.23 to 1393.95)	0.00% (-0.04% to 0.03%)

Luxembourg	1548.80 (282.15 to 3390.05)	438.03 (69.36 to 1001.47)	0.00% (-0.04% to 0.06%)	2924.12 (577.08 to 6060.70)	853.88 (145.86 to 1832.66)	0.00% (-0.04% to 0.03%)
Malta	990.43 (158.87 to 2207.34)	434.32 (58.91 to 979.82)	0.00% (-0.06% to 0.06%)	1930.77 (349.32 to 4132.86)	877.19 (126.55 to 1930.52)	0.00% (-0.04% to 0.03%)
Monaco	79.21 (13.63 to 177.69)	433.81 (57.70 to 996.00)	0.00% (-0.06% to 0.05%)	164.32 (31.05 to 347.27)	873.91 (128.54 to 1928.36)	-0.01% (-0.04% to 0.04%)
Montenegro	1235.16 (321.33 to 2642.46)	369.75 (87.99 to 812.66)	0.00% (-0.05% to 0.06%)	2248.09 (474.45 to 4979.21)	663.96 (123.82 to 1517.12)	-0.01% (-0.04% to 0.03%)
Netherlands	35975.33 (5355.13 to 81654.54)	411.30 (50.97 to 954.34)	0.08% (-0.04% to 0.20%)	73709.98 (10825.59 to 159757.40)	849.15 (103.99 to 1892.15)	0.04% (-0.04% to 0.11%)
North Macedonia	4663.65 (1221.23 to 10098.26)	369.44 (86.63 to 813.46)	0.00% (-0.05% to 0.05%)	8018.79 (1715.64 to 17798.76)	663.32 (127.85 to 1502.98)	0.00% (-0.05% to 0.03%)
Norway	12610.14 (1890.42 to 28627.45)	441.24 (57.01 to 1020.14)	0.10% (0.02% to 0.17%)	23859.23 (3670.83 to 52177.73)	876.41 (114.29 to 1934.27)	0.07% (0.01% to 0.13%)
Poland	81387.15 (21358.41 to 172032.04)	390.86 (91.99 to 835.61)	0.01% (-0.01% to 0.02%)	149218.49 (34739.85 to 323797.87)	696.09 (140.89 to 1561.34)	0.00% (-0.01% to 0.02%)
Portugal	22647.67 (3742.33 to 50574.80)	432.16 (57.57 to 985.96)	0.00% (-0.05% to 0.06%)	48911.68 (9004.43 to 103950.60)	871.59 (127.90 to 1919.25)	0.00% (-0.04% to 0.03%)
Republic of Moldova	6983.09 (2221.98 to 14735.93)	351.38 (101.96 to 771.73)	0.00% (-0.05% to 0.05%)	14863.49 (4476.62 to 30422.60)	696.66 (184.04 to 1481.75)	0.00% (-0.04% to 0.04%)
Romania	37653.57 (10068.49 to 80040.11)	369.83 (86.14 to 808.07)	0.01% (-0.05% to 0.07%)	69065.12 (15099.10 to 150552.11)	667.42 (125.92 to 1515.63)	0.00% (-0.04% to 0.05%)
Russian Federation	310833.48 (99201.01 to 643002.66)	411.02 (118.16 to 865.34)	0.01% (-0.01% to 0.02%)	644320.11 (209482.12 to 1295534.20)	757.11 (218.95 to 1552.26)	0.00% (-0.01% to 0.01%)
San Marino	70.01 (11.55 to 155.62)	433.59 (56.25 to 988.74)	0.00% (-0.04% to 0.05%)	151.71 (27.38 to 322.70)	874.55 (126.95 to 1898.54)	-0.01% (-0.05% to 0.03%)
Serbia	18439.96 (4754.58 to 39537.33)	370.43 (85.91 to 807.54)	0.00% (-0.06% to 0.05%)	32466.15 (7285.07 to 71033.00)	666.52 (131.18 to 1497.74)	0.00% (-0.05% to 0.04%)

Slovakia	10996.42 (2837.94 to 23480.96)	369.44 (85.86 to 807.94)	0.01% (-0.05% to 0.06%)	20143.50 (4339.25 to 44080.34)	666.60 (125.60 to 1518.48)	0.00% (-0.04% to 0.05%)
Slovenia	4201.33 (1135.79 to 8914.99)	369.63 (85.48 to 815.65)	0.01% (-0.05% to 0.06%)	7315.73 (1623.12 to 15946.09)	666.33 (124.68 to 1513.27)	0.00% (-0.04% to 0.04%)
Spain	106033.00 (16708.56 to 239714.82)	451.70 (58.64 to 1040.45)	0.01% (-0.04% to 0.11%)	221209.61 (44574.92 to 465367.26)	926.36 (152.61 to 2013.43)	0.00% (-0.06% to 0.05%)
Sweden	22826.38 (3477.71 to 49914.22)	428.90 (57.67 to 960.55)	0.01% (-0.06% to 0.08%)	45768.66 (6903.83 to 98656.65)	898.52 (113.88 to 1976.66)	0.00% (-0.06% to 0.07%)
Switzerland	18372.89 (3219.95 to 40997.15)	392.55 (58.18 to 906.93)	0.02% (-0.06% to 0.10%)	36264.43 (7152.93 to 76255.59)	795.72 (127.27 to 1735.98)	0.01% (-0.06% to 0.08%)
Ukraine	85325.67 (27058.09 to 175417.14)	368.27 (106.41 to 780.95)	0.00% (-0.05% to 0.04%)	186205.65 (57036.17 to 371400.12)	718.61 (192.42 to 1502.06)	0.00% (-0.04% to 0.04%)
United Kingdom	146363.05 (25019.41 to 326086.07)	425.00 (62.17 to 970.44)	0.01% (-0.04% to 0.06%)	305684.02 (50958.60 to 653960.03)	867.27 (125.08 to 1890.38)	0.00% (-0.05% to 0.05%)

Table S3g: Counts and age-standardized incidence rates of migraine in 2021, and percentage changes from 1990 to 2021, by country and sex

	Male			Female		
	**Absolute number, 2021**	**Age-standardized rate, per 100 000 people, 2021**	**Percentage Change, 1990-2021**	**Absolute number, 2021**	**Age-standardized rate, per 100 000 people, 2021**	**Percentage Change, 1990-2021**
**Europe**	**3217801.56 (2771062.45 to 3686607.65)**	**877.41 (752.49 to 1006.54)**	**0.02% (0.01% to 0.03%)**	**5839809.20 (5101893.46 to 6597893.91)**	**1631.07 (1422.68 to 1851.11)**	**0.01% (0.01% to 0.02%)**
Albania	9529.55 (8046.95 to 11056.51)	766.27 (642.07 to 891.11)	0.00% (0.00% to 0.00%)	16769.22 (14424.23 to 19500.48)	1424.76 (1218.61 to 1646.79)	0.00% (0.00% to 0.00%)
Andorra	351.22 (296.36 to 412.22)	963.18 (806.41 to 1125.26)	0.00% (0.00% to 0.00%)	603.55 (516.46 to 695.46)	1789.91 (1524.73 to 2056.13)	0.00% (0.00% to 0.00%)
Austria	34273.36 (29015.30 to 40211.01)	919.23 (771.79 to 1074.06)	0.00% (0.00% to 0.00%)	61094.79 (53066.52 to 69874.28)	1731.89 (1494.06 to 1988.04)	0.00% (0.00% to 0.00%)
Belarus	27922.20 (23397.61 to 32568.52)	679.80 (566.79 to 793.99)	0.00% (0.00% to 0.00%)	56307.15 (48166.85 to 64933.33)	1346.39 (1146.54 to 1540.72)	0.00% (0.00% to 0.00%)
Belgium	50913.30 (43333.62 to 58924.63)	1077.29 (907.33 to 1241.02)	0.02% (-0.04% to 0.08%)	88505.24 (76259.64 to 100531.89)	1950.20 (1673.57 to 2231.28)	0.02% (-0.03% to 0.08%)
Bosnia and Herzegovina	11167.39 (9476.28 to 12953.55)	766.27 (642.07 to 891.11)	0.00% (0.00% to 0.00%)	20159.08 (17270.22 to 23300.97)	1424.76 (1218.61 to 1646.79)	0.00% (0.00% to 0.00%)
Bulgaria	22370.23 (18924.18 to 26001.48)	766.27 (642.07 to 891.11)	0.00% (0.00% to 0.00%)	39794.91 (34251.41 to 46322.84)	1424.76 (1218.61 to 1646.79)	0.00% (0.00% to 0.00%)
Croatia	13858.37 (11840.08 to 16162.66)	761.76 (644.17 to 887.86)	-0.01% (-0.05% to 0.03%)	25078.94 (21680.89 to 29080.85)	1427.55 (1204.55 to 1651.13)	0.00% (-0.04% to 0.04%)
Cyprus	5826.81 (4847.71 to 6935.00)	963.18 (806.41 to 1125.26)	0.00% (0.00% to 0.00%)	10503.81 (8938.25 to 12328.68)	1789.91 (1524.73 to 2056.13)	0.00% (0.00% to 0.00%)
Czechia	35702.04 (30110.71 to 41633.79)	766.27 (642.07 to 891.11)	0.00% (0.00% to 0.00%)	63066.42 (54129.42 to 73422.36)	1424.76 (1218.61 to 1646.79)	0.00% (0.00% to 0.00%)

Denmark	21749.39 (18357.47 to 25319.59)	878.53 (732.26 to 1018.79)	0.01% (-0.05% to 0.07%)	39445.74 (34019.14 to 45293.43)	1670.87 (1429.05 to 1924.59)	0.01% (-0.03% to 0.06%)
Estonia	3876.98 (3261.85 to 4519.02)	679.80 (566.79 to 793.99)	0.00% (0.00% to 0.00%)	7484.99 (6446.32 to 8613.80)	1346.39 (1146.54 to 1540.72)	0.00% (0.00% to 0.00%)
Finland	21905.16 (18474.72 to 25625.95)	963.18 (806.41 to 1125.26)	0.00% (0.00% to 0.00%)	38440.14 (32979.70 to 43989.55)	1789.91 (1524.73 to 2056.13)	0.00% (0.00% to 0.00%)
France	270006.21 (230141.66 to 312055.06)	983.53 (832.50 to 1149.39)	0.00% (-0.06% to 0.06%)	476307.99 (410568.60 to 542989.07)	1743.79 (1498.10 to 1998.13)	-0.01% (-0.05% to 0.04%)
Germany	342190.77 (289600.26 to 402936.81)	1003.32 (840.34 to 1172.65)	0.02% (-0.04% to 0.07%)	580663.85 (507240.96 to 657137.89)	1874.22 (1615.32 to 2133.18)	0.01% (-0.03% to 0.06%)
Greece	39530.49 (33692.99 to 46141.51)	1002.42 (841.99 to 1168.92)	-0.01% (-0.06% to 0.05%)	70452.21 (60518.77 to 80838.33)	1806.93 (1543.98 to 2071.82)	0.00% (-0.05% to 0.05%)
Hungary	31501.91 (26580.64 to 36603.24)	766.27 (642.07 to 891.11)	0.00% (0.00% to 0.00%)	57686.40 (49729.88 to 66895.85)	1424.76 (1218.61 to 1646.79)	0.00% (0.00% to 0.00%)
Iceland	1536.48 (1288.40 to 1802.13)	963.18 (806.41 to 1125.26)	0.00% (0.00% to 0.00%)	2672.67 (2283.53 to 3053.55)	1789.91 (1524.73 to 2056.13)	0.00% (0.00% to 0.00%)
Ireland	21329.74 (17986.26 to 24883.76)	963.18 (806.41 to 1125.26)	0.00% (0.00% to 0.00%)	39393.83 (33675.73 to 45267.58)	1789.91 (1524.73 to 2056.13)	0.00% (0.00% to 0.00%)
Israel	44616.75 (37494.37 to 52318.84)	963.18 (806.41 to 1125.26)	0.00% (0.00% to 0.00%)	80705.56 (68859.86 to 92442.59)	1789.91 (1524.73 to 2056.13)	0.00% (0.00% to 0.00%)
Italy	239054.27 (210852.47 to 268253.42)	1037.94 (911.72 to 1176.40)	0.01% (-0.03% to 0.04%)	434191.52 (388852.03 to 485899.50)	1973.05 (1758.59 to 2211.41)	0.01% (-0.02% to 0.04%)
Latvia	5339.34 (4507.72 to 6219.00)	679.80 (566.79 to 793.99)	0.00% (0.00% to 0.00%)	10628.25 (9155.70 to 12228.04)	1346.39 (1146.54 to 1540.72)	0.00% (0.00% to 0.00%)
Lithuania	7190.94 (6145.95 to 8401.47)	625.29 (526.75 to 732.48)	0.00% (0.00% to 0.00%)	15393.41 (13357.87 to 17874.05)	1335.21 (1138.73 to 1541.94)	0.00% (0.00% to 0.00%)

Luxembourg	2727.16 (2311.68 to 3230.54)	954.35 (804.08 to 1118.37)	0.00% (0.00% to 0.00%)	4666.35 (3994.11 to 5376.46)	1734.29 (1477.92 to 1990.38)	0.00% (0.00% to 0.00%)
Malta	1725.02 (1448.44 to 2035.28)	963.18 (806.41 to 1125.26)	0.00% (0.00% to 0.00%)	2955.96 (2532.42 to 3430.82)	1789.91 (1524.73 to 2056.13)	0.00% (0.00% to 0.00%)
Monaco	130.82 (111.56 to 151.46)	963.18 (806.41 to 1125.26)	0.00% (0.00% to 0.00%)	241.89 (209.73 to 275.61)	1789.91 (1524.73 to 2056.13)	0.00% (0.00% to 0.00%)
Montenegro	2195.88 (1855.24 to 2543.84)	766.27 (642.07 to 891.11)	0.00% (0.00% to 0.00%)	3947.93 (3388.64 to 4564.63)	1424.76 (1218.61 to 1646.79)	0.00% (0.00% to 0.00%)
Netherlands	66342.25 (56446.75 to 77243.92)	935.95 (787.99 to 1087.19)	0.06% (0.00% to 0.15%)	121673.43 (105974.23 to 138874.89)	1781.10 (1528.47 to 2043.56)	0.02% (-0.02% to 0.07%)
North Macedonia	7940.32 (6692.40 to 9270.02)	766.27 (642.07 to 891.11)	0.00% (0.00% to 0.00%)	13864.04 (11793.78 to 16247.49)	1424.76 (1218.61 to 1646.79)	0.00% (0.00% to 0.00%)
Norway	23798.83 (21000.32 to 26903.02)	997.03 (874.18 to 1131.91)	0.08% (0.05% to 0.13%)	41941.49 (37239.69 to 46981.80)	1878.96 (1662.44 to 2119.14)	0.06% (0.03% to 0.09%)
Poland	135003.76 (119088.16 to 153264.94)	792.42 (690.29 to 901.48)	0.00% (0.00% to 0.00%)	247963.29 (219456.29 to 282516.87)	1482.61 (1315.33 to 1674.93)	0.00% (0.00% to 0.00%)
Portugal	38984.57 (32954.70 to 45549.93)	963.18 (806.41 to 1125.26)	0.00% (0.00% to 0.00%)	73260.29 (62905.05 to 84271.64)	1789.91 (1524.73 to 2056.13)	0.00% (0.00% to 0.00%)
Republic of Moldova	11059.41 (9234.66 to 12992.51)	679.80 (566.79 to 793.99)	0.00% (0.00% to 0.00%)	21966.78 (18770.04 to 25598.39)	1346.39 (1146.54 to 1540.72)	0.00% (0.00% to 0.00%)
Romania	63934.98 (53901.49 to 74170.34)	766.27 (642.07 to 891.11)	0.00% (0.00% to 0.00%)	114129.27 (98508.37 to 132327.13)	1424.76 (1218.61 to 1646.79)	0.00% (0.00% to 0.00%)
Russian Federation	494399.77 (433115.09 to 558781.97)	767.37 (673.07 to 868.02)	0.00% (0.00% to 0.00%)	939350.95 (834379.17 to 1056351.42)	1411.48 (1243.69 to 1588.16)	0.00% (0.00% to 0.00%)
San Marino	121.95 (103.68 to 141.94)	963.18 (806.41 to 1125.26)	0.00% (0.00% to 0.00%)	230.24 (197.26 to 262.60)	1789.91 (1524.73 to 2056.13)	0.00% (0.00% to 0.00%)
Serbia	31913.71 (26902.78 to 37095.62)	766.27 (642.07 to 891.11)	0.00% (0.00% to 0.00%)	54901.06 (47012.06 to 63770.52)	1424.76 (1218.61 to 1646.79)	0.00% (0.00% to 0.00%)

Slovakia	18693.72 (15719.75 to 21836.37)	766.27 (642.07 to 891.11)	0.00% (0.00% to 0.00%)	33703.04 (28825.39 to 39430.65)	1424.76 (1218.61 to 1646.79)	0.00% (0.00% to 0.00%)
Slovenia	6910.88 (5864.11 to 8045.49)	766.27 (642.07 to 891.11)	0.00% (0.00% to 0.00%)	11933.41 (10280.37 to 13888.61)	1424.76 (1218.61 to 1646.79)	0.00% (0.00% to 0.00%)
Spain	180943.02 (153556.57 to 208875.66)	987.99 (833.43 to 1145.50)	0.00% (0.00% to 0.00%)	322002.41 (277545.88 to 372307.11)	1813.88 (1558.01 to 2105.75)	0.00% (0.00% to 0.00%)
Sweden	42879.21 (37750.50 to 48441.80)	959.44 (841.87 to 1087.20)	0.00% (-0.05% to 0.05%)	78156.30 (69537.84 to 87686.94)	1888.43 (1678.46 to 2120.58)	0.00% (-0.03% to 0.03%)
Switzerland	33537.10 (28449.06 to 39346.43)	897.55 (754.94 to 1050.91)	0.01% (-0.04% to 0.07%)	59488.64 (51303.48 to 68197.42)	1693.43 (1444.77 to 1954.31)	0.00% (-0.04% to 0.05%)
Ukraine	134816.68 (117819.51 to 153078.47)	703.84 (611.75 to 803.21)	0.00% (0.00% to 0.00%)	273313.59 (242377.47 to 306997.49)	1393.99 (1231.44 to 1570.25)	0.00% (0.00% to 0.00%)
United Kingdom	274264.40 (240052.58 to 309615.76)	948.69 (824.97 to 1078.25)	0.01% (-0.03% to 0.05%)	523205.73 (466705.72 to 589485.48)	1830.27 (1616.88 to 2070.98)	0.00% (-0.03% to 0.03%)

Table S3h: Counts and age-standardized YLD rates of migraine in 2021, and percentage changes from 1990 to 2021, by country and sex

	Male			Female		
	**Absolute number, 2021**	**Age-standardized rate, per 100 000 people, 2021**	**Percentage Change, 1990-2021**	**Absolute number, 2021**	**Age-standardized rate, per 100 000 people, 2021**	**Percentage Change, 1990-2021**
**Europe**	**1859028.36 (409180.70 to 3972660.88)**	**420.49 (81.01 to 930.36)**	**0.01% (-0.01% to 0.03%)**	**3718057.71 (824559.40 to 7770294.82)**	**815.91 (151.00 to 1739.13)**	**0.01% (-0.02% to 0.03%)**
Albania	5418.86 (1424.13 to 11543.52)	369.75 (87.33 to 805.44)	0.00% (-0.05% to 0.05%)	9653.43 (2133.16 to 21050.44)	666.44 (130.79 to 1501.37)	0.00% (-0.04% to 0.04%)
Andorra	211.91 (35.42 to 473.98)	433.55 (59.17 to 988.63)	0.00% (-0.06% to 0.06%)	399.05 (68.84 to 848.52)	875.21 (126.15 to 1922.32)	0.00% (-0.04% to 0.04%)
Austria	19296.15 (3742.42 to 43852.38)	410.80 (67.64 to 956.56)	0.00% (-0.06% to 0.06%)	38424.10 (7117.37 to 80266.90)	827.22 (125.95 to 1782.41)	0.00% (-0.04% to 0.05%)
Belarus	17202.86 (5514.54 to 36102.47)	350.44 (98.93 to 756.24)	0.00% (-0.05% to 0.05%)	37973.25 (11431.93 to 77840.45)	695.14 (181.04 to 1471.83)	0.00% (-0.04% to 0.03%)
Belgium	30216.94 (4047.17 to 67008.94)	520.07 (59.41 to 1159.94)	0.04% (-0.06% to 0.13%)	63797.92 (8812.30 to 143038.98)	1083.08 (125.37 to 2466.04)	0.08% (-0.04% to 0.20%)
Bosnia and Herzegovina	6629.91 (1764.56 to 14128.60)	368.05 (84.79 to 812.04)	0.00% (-0.05% to 0.06%)	12185.43 (2752.79 to 26670.29)	663.69 (130.29 to 1502.15)	-0.01% (-0.04% to 0.04%)
Bulgaria	13484.33 (3658.19 to 28927.95)	368.37 (85.20 to 810.50)	0.00% (-0.05% to 0.05%)	24732.94 (5650.78 to 53721.10)	664.18 (127.33 to 1500.17)	0.00% (-0.04% to 0.04%)
Croatia	8356.32 (2246.14 to 17455.42)	368.16 (86.56 to 801.60)	0.00% (-0.07% to 0.07%)	15387.87 (3502.10 to 33924.87)	666.82 (130.26 to 1502.15)	0.00% (-0.06% to 0.07%)
Cyprus	3152.56 (471.28 to 7215.94)	435.05 (58.41 to 1002.07)	0.00% (-0.05% to 0.07%)	6579.36 (1078.81 to 14085.57)	876.83 (126.31 to 1918.37)	0.00% (-0.04% to 0.03%)
Czechia	21198.92 (5597.92 to 45330.46)	368.76 (85.22 to 811.75)	0.00% (-0.05% to 0.06%)	37897.68 (8417.90 to 83289.48)	664.87 (127.05 to 1517.91)	0.00% (-0.04% to 0.04%)

Denmark	11658.53 (2170.29 to 26019.65)	388.17 (60.66 to 895.77)	0.03% (-0.05% to 0.11%)	22908.40 (3942.39 to 49148.67)	779.90 (114.24 to 1689.65)	0.03% (-0.05% to 0.10%)
Estonia	2423.28 (771.80 to 5064.63)	350.71 (98.90 to 762.74)	0.00% (-0.04% to 0.06%)	5053.57 (1528.82 to 10382.41)	696.55 (182.37 to 1477.68)	0.00% (-0.03% to 0.04%)
Finland	12060.45 (1911.18 to 26943.61)	432.34 (56.76 to 986.09)	0.00% (-0.05% to 0.06%)	23951.67 (4321.47 to 51422.39)	876.04 (125.05 to 1939.10)	0.00% (-0.04% to 0.04%)
France	149881.54 (26306.55 to 330810.56)	457.45 (69.26 to 1040.00)	-0.01% (-0.09% to 0.07%)	291519.25 (58169.25 to 604091.44)	861.81 (144.52 to 1826.00)	-0.01% (-0.07% to 0.07%)
Germany	203766.88 (34397.63 to 458687.39)	462.90 (63.18 to 1074.57)	0.03% (-0.06% to 0.12%)	411970.87 (70087.16 to 873933.68)	968.50 (138.04 to 2089.72)	0.02% (-0.06% to 0.10%)
Greece	23224.23 (3661.95 to 53112.46)	456.26 (59.23 to 1067.04)	-0.01% (-0.09% to 0.08%)	46761.71 (8138.33 to 100780.90)	890.95 (122.25 to 1978.71)	-0.01% (-0.08% to 0.07%)
Hungary	18888.42 (4962.58 to 40308.14)	369.86 (85.43 to 809.53)	0.01% (-0.05% to 0.07%)	35699.42 (8101.90 to 78199.98)	665.38 (129.81 to 1488.51)	0.00% (-0.04% to 0.05%)
Iceland	803.29 (119.74 to 1830.76)	435.08 (57.39 to 1002.07)	0.00% (-0.05% to 0.06%)	1549.19 (251.80 to 3341.26)	878.48 (127.83 to 1932.61)	0.00% (-0.04% to 0.04%)
Ireland	10914.14 (1661.93 to 24505.01)	432.28 (57.31 to 991.34)	0.00% (-0.06% to 0.05%)	22446.14 (3767.42 to 47864.64)	872.58 (127.06 to 1904.33)	0.00% (-0.04% to 0.03%)
Israel	20461.67 (2908.77 to 47162.36)	435.56 (59.60 to 1007.18)	0.00% (-0.06% to 0.05%)	41228.52 (6263.11 to 90236.41)	879.48 (124.91 to 1935.81)	0.00% (-0.04% to 0.04%)
Italy	143803.30 (22379.71 to 314245.48)	474.46 (58.87 to 1062.93)	0.01% (-0.05% to 0.06%)	294765.35 (47051.64 to 630401.09)	964.57 (119.79 to 2154.27)	0.02% (-0.04% to 0.07%)
Latvia	3367.67 (1105.00 to 6920.88)	350.44 (100.79 to 757.58)	0.01% (-0.04% to 0.07%)	7393.37 (2256.50 to 14891.16)	696.51 (180.66 to 1478.42)	0.00% (-0.04% to 0.04%)
Lithuania	4375.61 (1366.48 to 8965.66)	309.96 (85.57 to 659.07)	0.01% (-0.05% to 0.06%)	10209.14 (2879.02 to 21001.01)	658.58 (155.23 to 1393.95)	0.00% (-0.04% to 0.03%)

Luxembourg	1548.80 (282.15 to 3390.05)	438.03 (69.36 to 1001.47)	0.00% (-0.04% to 0.06%)	2924.12 (577.08 to 6060.70)	853.88 (145.86 to 1832.66)	0.00% (-0.04% to 0.03%)
Malta	990.43 (158.87 to 2207.34)	434.32 (58.91 to 979.82)	0.00% (-0.06% to 0.06%)	1930.77 (349.32 to 4132.86)	877.19 (126.55 to 1930.52)	0.00% (-0.04% to 0.03%)
Monaco	79.21 (13.63 to 177.69)	433.81 (57.70 to 996.00)	0.00% (-0.06% to 0.05%)	164.32 (31.05 to 347.27)	873.91 (128.54 to 1928.36)	-0.01% (-0.04% to 0.04%)
Montenegro	1235.16 (321.33 to 2642.46)	369.75 (87.99 to 812.66)	0.00% (-0.05% to 0.06%)	2248.09 (474.45 to 4979.21)	663.96 (123.82 to 1517.12)	-0.01% (-0.04% to 0.03%)
Netherlands	35975.33 (5355.13 to 81654.54)	411.30 (50.97 to 954.34)	0.08% (-0.04% to 0.20%)	73709.98 (10825.59 to 159757.40)	849.15 (103.99 to 1892.15)	0.04% (-0.04% to 0.11%)
North Macedonia	4663.65 (1221.23 to 10098.26)	369.44 (86.63 to 813.46)	0.00% (-0.05% to 0.05%)	8018.79 (1715.64 to 17798.76)	663.32 (127.85 to 1502.98)	0.00% (-0.05% to 0.03%)
Norway	12610.14 (1890.42 to 28627.45)	441.24 (57.01 to 1020.14)	0.10% (0.02% to 0.17%)	23859.23 (3670.83 to 52177.73)	876.41 (114.29 to 1934.27)	0.07% (0.01% to 0.13%)
Poland	81387.15 (21358.41 to 172032.04)	390.86 (91.99 to 835.61)	0.01% (-0.01% to 0.02%)	149218.49 (34739.85 to 323797.87)	696.09 (140.89 to 1561.34)	0.00% (-0.01% to 0.02%)
Portugal	22647.67 (3742.33 to 50574.80)	432.16 (57.57 to 985.96)	0.00% (-0.05% to 0.06%)	48911.68 (9004.43 to 103950.60)	871.59 (127.90 to 1919.25)	0.00% (-0.04% to 0.03%)
Republic of Moldova	6983.09 (2221.98 to 14735.93)	351.38 (101.96 to 771.73)	0.00% (-0.05% to 0.05%)	14863.49 (4476.62 to 30422.60)	696.66 (184.04 to 1481.75)	0.00% (-0.04% to 0.04%)
Romania	37653.57 (10068.49 to 80040.11)	369.83 (86.14 to 808.07)	0.01% (-0.05% to 0.07%)	69065.12 (15099.10 to 150552.11)	667.42 (125.92 to 1515.63)	0.00% (-0.04% to 0.05%)
Russian Federation	310833.48 (99201.01 to 643002.66)	411.02 (118.16 to 865.34)	0.01% (-0.01% to 0.02%)	644320.11 (209482.12 to 1295534.20)	757.11 (218.95 to 1552.26)	0.00% (-0.01% to 0.01%)
San Marino	70.01 (11.55 to 155.62)	433.59 (56.25 to 988.74)	0.00% (-0.04% to 0.05%)	151.71 (27.38 to 322.70)	874.55 (126.95 to 1898.54)	-0.01% (-0.05% to 0.03%)
Serbia	18439.96 (4754.58 to 39537.33)	370.43 (85.91 to 807.54)	0.00% (-0.06% to 0.05%)	32466.15 (7285.07 to 71033.00)	666.52 (131.18 to 1497.74)	0.00% (-0.05% to 0.04%)

Slovakia	10996.42 (2837.94 to 23480.96)	369.44 (85.86 to 807.94)	0.01% (-0.05% to 0.06%)	20143.50 (4339.25 to 44080.34)	666.60 (125.60 to 1518.48)	0.00% (-0.04% to 0.05%)
Slovenia	4201.33 (1135.79 to 8914.99)	369.63 (85.48 to 815.65)	0.01% (-0.05% to 0.06%)	7315.73 (1623.12 to 15946.09)	666.33 (124.68 to 1513.27)	0.00% (-0.04% to 0.04%)
Spain	106033.00 (16708.56 to 239714.82)	451.70 (58.64 to 1040.45)	0.01% (-0.04% to 0.11%)	221209.61 (44574.92 to 465367.26)	926.36 (152.61 to 2013.43)	0.00% (-0.06% to 0.05%)
Sweden	22826.38 (3477.71 to 49914.22)	428.90 (57.67 to 960.55)	0.01% (-0.06% to 0.08%)	45768.66 (6903.83 to 98656.65)	898.52 (113.88 to 1976.66)	0.00% (-0.06% to 0.07%)
Switzerland	18372.89 (3219.95 to 40997.15)	392.55 (58.18 to 906.93)	0.02% (-0.06% to 0.10%)	36264.43 (7152.93 to 76255.59)	795.72 (127.27 to 1735.98)	0.01% (-0.06% to 0.08%)
Ukraine	85325.67 (27058.09 to 175417.14)	368.27 (106.41 to 780.95)	0.00% (-0.05% to 0.04%)	186205.65 (57036.17 to 371400.12)	718.61 (192.42 to 1502.06)	0.00% (-0.04% to 0.04%)
United Kingdom	146363.05 (25019.41 to 326086.07)	425.00 (62.17 to 970.44)	0.01% (-0.04% to 0.06%)	305684.02 (50958.60 to 653960.03)	867.27 (125.08 to 1890.38)	0.00% (-0.05% to 0.05%)

Table S3i: Counts and age-standardized prevalence rates of tension-type headache in 2021, and percentage changes from 1990 to 2021, by country and sex

	Male			Female		
	**Absolute number, 2021**	**Age-standardized rate, per 100 000 people, 2021**	**Percentage Change, 1990-2021**	**Absolute number, 2021**	**Age-standardized rate, per 100 000 people, 2021**	**Percentage Change, 1990-2021**
**Europe**	**133449338.95 (118544091.44 to 149064034.63)**	**30043.09 (26465.42 to 33994.50)**	**-0.01% (-0.02% to 0.01%)**	**149044989.85 (133037481.88 to 165493921.45)**	**31538.63 (27993.01 to 35543.12)**	**-0.01% (-0.02% to 0.00%)**
Albania	412649.41 (356270.48 to 474668.53)	28499.84 (24463.48 to 32957.21)	0.00% (-0.01% to 0.01%)	451683.76 (397782.67 to 511215.26)	31393.94 (27424.02 to 35977.41)	0.00% (-0.01% to 0.01%)
Andorra	15635.50 (13466.49 to 17851.07)	31377.90 (26821.63 to 36028.77)	0.00% (-0.01% to 0.01%)	15787.99 (13812.62 to 17887.61)	33395.89 (29218.20 to 38101.13)	0.00% (0.00% to 0.01%)
Austria	1515290.49 (1316783.61 to 1714033.61)	31445.25 (27055.38 to 36011.08)	0.00% (-0.01% to 0.01%)	1588067.33 (1390258.44 to 1790919.35)	32059.98 (28074.90 to 36713.69)	0.00% (-0.01% to 0.01%)
Belarus	1405021.66 (1227687.55 to 1603669.90)	30136.67 (26260.73 to 34594.93)	0.00% (-0.01% to 0.01%)	1627595.80 (1432092.88 to 1857943.68)	30507.31 (26473.60 to 34848.30)	0.00% (0.00% to 0.00%)
Belgium	1889130.32 (1635933.92 to 2144015.17)	31377.98 (26837.07 to 36060.40)	0.00% (-0.01% to 0.01%)	2080486.52 (1828931.28 to 2340554.08)	33395.49 (29244.64 to 38012.29)	0.00% (-0.01% to 0.00%)
Bosnia and Herzegovina	500018.03 (433643.29 to 568577.42)	28499.90 (24473.50 to 32934.79)	0.00% (-0.01% to 0.01%)	577380.56 (509316.23 to 652024.24)	31393.86 (27429.10 to 36025.12)	0.00% (-0.01% to 0.01%)
Bulgaria	1017611.18 (886396.40 to 1154350.03)	28499.62 (24457.39 to 32815.59)	0.00% (-0.01% to 0.01%)	1191971.14 (1048262.85 to 1342117.15)	31394.48 (27426.63 to 36015.14)	0.00% (-0.01% to 0.01%)
Croatia	633768.36 (553739.82 to 718823.98)	28540.20 (24952.55 to 32773.31)	0.00% (-0.05% to 0.06%)	738718.99 (636133.70 to 828291.17)	31347.71 (27394.95 to 35896.52)	0.00% (-0.05% to 0.06%)
Cyprus	230098.40 (198306.36 to 262905.72)	31376.81 (26834.51 to 36002.39)	0.00% (-0.01% to 0.01%)	256313.02 (224624.47 to 290812.31)	33395.99 (29286.36 to 37996.48)	0.00% (-0.01% to 0.01%)

Czechia	1601962.32 (1394874.29 to 1817682.43)	28499.27 (24464.46 to 32918.57)	0.00% (0.00% to 0.01%)	1819236.87 (1605900.00 to 2048240.94)	31393.36 (27430.02 to 36106.70)	0.00% (-0.01% to 0.01%)
Denmark	981541.12 (855617.41 to 1112566.27)	31596.76 (27485.71 to 36270.57)	-0.01% (-0.06% to 0.04%)	1134464.41 (994926.65 to 1280537.53)	35951.39 (31046.59 to 41046.02)	-0.04% (-0.09% to 0.02%)
Estonia	199847.12 (174843.48 to 227599.29)	30137.01 (26224.39 to 34663.36)	0.00% (-0.01% to 0.01%)	224117.46 (197727.39 to 253654.52)	30507.46 (26476.55 to 35027.72)	0.00% (-0.01% to 0.01%)
Finland	917539.42 (795034.21 to 1041823.20)	31377.83 (26852.18 to 36057.52)	0.00% (-0.01% to 0.01%)	1000293.96 (876745.19 to 1123953.35)	33395.96 (29286.77 to 38057.83)	0.00% (-0.01% to 0.01%)
France	10567799.82 (9183718.50 to 11952746.91)	31228.79 (26894.92 to 36100.77)	0.00% (-0.01% to 0.01%)	11417028.21 (10056377.75 to 12839025.18)	31581.39 (27677.44 to 36176.12)	0.00% (-0.01% to 0.01%)
Germany	14608281.76 (12695667.72 to 16515388.55)	31757.22 (27594.68 to 36142.21)	0.02% (-0.03% to 0.08%)	15958010.23 (14125161.84 to 17936558.67)	34502.99 (30463.94 to 39176.13)	0.02% (-0.03% to 0.07%)
Greece	1670647.45 (1448679.38 to 1895277.09)	31377.01 (26776.51 to 36023.14)	0.00% (-0.01% to 0.01%)	1900235.83 (1664266.41 to 2133680.92)	33396.24 (29258.10 to 38087.75)	0.00% (-0.01% to 0.01%)
Hungary	1415675.69 (1229845.63 to 1607518.17)	28500.16 (24454.53 to 32932.08)	0.00% (-0.01% to 0.01%)	1711377.23 (1510142.58 to 1923519.09)	31394.63 (27428.75 to 36022.36)	0.00% (-0.01% to 0.01%)
Iceland	59186.69 (51325.88 to 67401.64)	31378.83 (26895.33 to 36043.25)	0.00% (-0.01% to 0.01%)	61345.71 (54135.50 to 69646.60)	33395.60 (29274.60 to 38083.67)	0.00% (-0.01% to 0.01%)
Ireland	806720.26 (698010.45 to 915157.04)	31377.92 (26857.95 to 36082.83)	0.00% (-0.01% to 0.01%)	890125.05 (782982.95 to 1003836.39)	33395.22 (29223.76 to 38000.69)	0.00% (-0.01% to 0.01%)
Israel	1487411.91 (1276038.07 to 1696942.55)	31377.31 (26855.79 to 35991.18)	0.00% (-0.01% to 0.01%)	1607332.29 (1414233.70 to 1825828.11)	33395.70 (29270.97 to 38102.77)	0.00% (-0.01% to 0.01%)
Italy	10385376.36 (9295299.95 to 11438055.76)	32853.87 (29324.10 to 36867.11)	0.00% (-0.03% to 0.04%)	11454276.99 (10179696.08 to 12632166.36)	34270.64 (30617.95 to 38169.79)	0.00% (-0.03% to 0.03%)
Latvia	276597.19 (241743.29 to 314800.10)	30136.51 (26227.52 to 34609.93)	0.00% (-0.01% to 0.01%)	326394.51 (287234.71 to 368787.26)	30506.02 (26482.99 to 34940.80)	0.00% (-0.01% to 0.01%)

Lithuania	427700.91 (371178.05 to 487467.99)	31721.93 (27438.72 to 36290.84)	0.00% (-0.01% to 0.01%)	497064.88 (438275.21 to 557103.96)	31722.27 (27426.29 to 35956.90)	0.00% (-0.01% to 0.01%)
Luxembourg	113290.77 (98675.81 to 129998.04)	31760.66 (27542.01 to 36706.28)	0.00% (-0.01% to 0.01%)	115680.49 (101576.71 to 130915.67)	32798.22 (28453.87 to 37474.93)	0.00% (-0.01% to 0.01%)
Malta	74563.53 (64516.27 to 84852.61)	31376.76 (26813.62 to 36048.94)	0.00% (-0.01% to 0.01%)	79607.74 (69715.27 to 89758.71)	33395.77 (29243.60 to 38094.23)	0.00% (-0.01% to 0.01%)
Monaco	6122.61 (5323.38 to 6984.93)	31378.38 (26877.91 to 36074.98)	0.00% (-0.01% to 0.01%)	6966.58 (6060.91 to 7837.46)	33395.79 (29261.44 to 38091.41)	0.00% (-0.01% to 0.01%)
Montenegro	92994.83 (80583.45 to 106189.71)	28499.88 (24483.78 to 32933.71)	0.00% (-0.01% to 0.01%)	105596.73 (93210.74 to 119273.26)	31394.19 (27406.99 to 35913.81)	0.00% (-0.01% to 0.01%)
Netherlands	3127198.27 (2710362.29 to 3564271.50)	34154.58 (29195.05 to 39268.16)	0.00% (-0.01% to 0.01%)	3348863.20 (2947171.44 to 3772965.56)	35804.31 (30725.33 to 40725.15)	0.00% (-0.01% to 0.01%)
North Macedonia	347411.84 (300571.15 to 396930.91)	28499.42 (24505.41 to 32922.36)	0.00% (-0.01% to 0.01%)	371056.50 (327106.54 to 421395.41)	31394.06 (27430.50 to 36040.79)	0.00% (-0.01% to 0.01%)
Norway	984250.09 (871706.16 to 1091227.61)	33588.77 (29810.44 to 37568.02)	0.02% (-0.02% to 0.06%)	1078993.34 (969357.80 to 1191574.79)	37461.39 (33539.48 to 41766.70)	0.00% (-0.04% to 0.04%)
Poland	6061931.73 (5376094.11 to 6758679.84)	30066.67 (26780.03 to 33699.29)	0.00% (0.00% to 0.00%)	7078323.15 (6293877.64 to 7811316.86)	32956.50 (29157.74 to 36821.22)	0.00% (0.00% to 0.00%)
Portugal	1713044.66 (1483800.55 to 1945011.38)	31378.24 (26864.36 to 35958.55)	0.00% (-0.01% to 0.01%)	2034545.69 (1778615.51 to 2285255.15)	33395.51 (29283.07 to 37992.90)	0.00% (-0.01% to 0.01%)
Republic of Moldova	570546.02 (495187.68 to 652049.74)	30136.03 (26230.19 to 34622.39)	0.00% (-0.01% to 0.01%)	631577.81 (555209.42 to 721359.86)	30507.40 (26503.46 to 34972.42)	0.00% (-0.01% to 0.01%)
Romania	2825977.39 (2452161.77 to 3209783.43)	28499.70 (24471.58 to 32896.01)	0.00% (-0.01% to 0.00%)	3291220.21 (2903995.74 to 3698590.98)	31393.51 (27400.20 to 36067.57)	0.00% (-0.01% to 0.01%)
Russian Federation	22137249.52 (19651675.13 to 24784626.24)	30677.05 (27299.81 to 34502.64)	0.01% (-0.03% to 0.06%)	26088741.65 (23415523.10 to 28694110.76)	31557.05 (27907.37 to 35435.34)	0.01% (-0.03% to 0.05%)

San Marino	5335.72 (4610.06 to 6072.36)	31377.30 (26855.05 to 36019.36)	0.00% (-0.01% to 0.01%)	6214.66 (5445.75 to 6991.89)	33395.39 (29212.82 to 38045.29)	0.00% (-0.01% to 0.01%)
Serbia	1395497.15 (1211643.98 to 1587185.37)	28501.85 (24469.52 to 32920.80)	0.00% (-0.01% to 0.01%)	1542013.90 (1358637.66 to 1733100.35)	31395.72 (27436.77 to 35957.14)	0.00% (-0.01% to 0.01%)
Slovakia	821423.09 (713004.57 to 933300.53)	28500.29 (24461.91 to 32942.68)	0.00% (-0.01% to 0.01%)	946916.82 (833756.10 to 1066127.38)	31393.70 (27419.97 to 35992.94)	0.00% (-0.01% to 0.01%)
Slovenia	316014.59 (274838.05 to 358922.68)	28500.52 (24484.56 to 32948.23)	0.00% (-0.01% to 0.01%)	351476.55 (310546.99 to 394912.93)	31394.23 (27424.13 to 35955.67)	0.00% (-0.01% to 0.01%)
Spain	7564549.77 (6610293.81 to 8562792.49)	31231.70 (26932.03 to 35856.15)	0.00% (-0.01% to 0.01%)	8075943.93 (7011161.11 to 9185196.05)	31818.27 (27794.41 to 36453.31)	0.00% (-0.01% to 0.01%)
Sweden	1751447.96 (1561216.76 to 1945888.99)	31790.27 (28302.59 to 35807.51)	0.00% (-0.04% to 0.04%)	2020269.73 (1817536.34 to 2219642.71)	36887.92 (33102.18 to 40972.22)	0.00% (-0.03% to 0.04%)
Switzerland	1389472.50 (1202698.63 to 1580124.92)	28846.37 (24857.03 to 33176.44)	0.05% (-0.02% to 0.12%)	1494776.43 (1308685.20 to 1691224.63)	30825.50 (26843.70 to 35204.93)	0.05% (-0.01% to 0.12%)
Ukraine	6947519.75 (6197608.27 to 7767363.42)	31625.32 (28174.40 to 35411.47)	0.00% (-0.01% to 0.01%)	8115481.54 (7188089.23 to 9010667.15)	32082.25 (28414.53 to 36075.87)	0.00% (0.00% to 0.01%)
United Kingdom	11474737.07 (10241611.51 to 12748287.12)	32434.77 (28746.26 to 36320.98)	0.00% (0.00% to 0.00%)	12819430.45 (11517741.51 to 14160915.17)	34492.86 (30830.66 to 38310.59)	0.00% (0.00% to 0.00%)

Table S3j: Counts and age-standardized DALY rates of tension-type headache in 2021, and percentage changes from 1990 to 2021, by country and sex

	Male			Female		
	**Absolute number, 2021**	**Age-standardized rate, per 100 000 people, 2021**	**Percentage Change, 1990-2021**	**Absolute number, 2021**	**Age-standardized rate, per 100 000 people, 2021**	**Percentage Change, 1990-2021**
**Europe**	**297654.68 (83195.64 to 1044194.41)**	**62.87 (16.40 to 232.91)**	**0.00% (-0.06% to 0.03%)**	**470322.34 (162163.34 to 1359954.49)**	**92.26 (30.36 to 280.15)**	**-0.01% (-0.05% to 0.01%)**
Albania	951.50 (265.62 to 3254.72)	62.15 (16.59 to 219.67)	0.01% (-0.13% to 0.15%)	1300.24 (430.81 to 3875.29)	83.88 (26.64 to 264.81)	0.00% (-0.10% to 0.13%)
Andorra	30.00 (6.79 to 118.16)	55.69 (11.20 to 229.80)	0.00% (-0.17% to 0.20%)	43.16 (13.03 to 139.38)	83.33 (23.51 to 288.42)	0.00% (-0.13% to 0.10%)
Austria	3019.30 (723.28 to 12261.99)	59.07 (13.14 to 248.86)	0.00% (-0.17% to 0.21%)	4424.68 (1415.89 to 13946.15)	83.44 (24.87 to 281.00)	0.00% (-0.14% to 0.14%)
Belarus	3647.45 (1068.44 to 11746.48)	71.21 (19.64 to 247.08)	0.01% (-0.12% to 0.16%)	6213.38 (2262.44 to 16489.58)	103.42 (34.82 to 292.34)	0.00% (-0.09% to 0.09%)
Belgium	3526.05 (811.17 to 14261.41)	55.60 (11.68 to 232.63)	0.00% (-0.18% to 0.24%)	5468.74 (1651.21 to 17717.23)	83.20 (24.04 to 283.30)	0.00% (-0.13% to 0.11%)
Bosnia and Herzegovina	1187.67 (350.51 to 3906.06)	62.00 (17.24 to 216.93)	0.01% (-0.15% to 0.14%)	1679.50 (572.15 to 4874.67)	83.26 (26.56 to 261.67)	0.00% (-0.13% to 0.12%)
Bulgaria	2440.40 (695.07 to 8031.90)	62.19 (16.50 to 220.42)	0.01% (-0.13% to 0.17%)	3466.25 (1140.02 to 10233.12)	83.46 (25.66 to 263.51)	0.00% (-0.12% to 0.10%)
Croatia	1510.56 (444.46 to 4960.61)	62.31 (16.79 to 222.14)	0.01% (-0.13% to 0.13%)	2146.54 (722.99 to 6468.99)	83.78 (25.97 to 269.17)	0.00% (-0.12% to 0.11%)
Cyprus	425.88 (93.67 to 1697.77)	55.68 (11.79 to 228.91)	0.01% (-0.22% to 0.18%)	685.12 (203.07 to 2235.29)	83.43 (23.81 to 288.71)	0.00% (-0.13% to 0.11%)
Czechia	3818.49 (1077.78 to 12475.21)	62.18 (16.29 to 223.62)	0.01% (-0.14% to 0.14%)	5235.99 (1765.68 to 15681.31)	83.50 (25.99 to 269.46)	0.00% (-0.12% to 0.13%)
Denmark	1865.51 (427.83 to 7431.85)	57.15 (11.94 to 233.08)	0.00% (-0.18% to 0.22%)	2767.13 (761.22 to 9684.86)	83.77 (22.39 to 300.34)	-0.01% (-0.16% to 0.13%)

Estonia	516.79 (152.26 to 1677.48)	71.39 (19.83 to 246.06)	0.01% (-0.11% to 0.13%)	833.21 (308.63 to 2260.49)	103.74 (35.70 to 296.32)	0.00% (-0.10% to 0.10%)
Finland	1708.83 (389.20 to 6812.26)	55.52 (11.69 to 230.71)	0.01% (-0.19% to 0.20%)	2627.59 (810.06 to 8441.99)	83.35 (24.75 to 280.78)	0.00% (-0.12% to 0.13%)
France	21185.62 (4989.61 to 78003.39)	59.33 (13.59 to 229.31)	0.00% (-0.17% to 0.18%)	34637.35 (11360.81 to 100985.71)	89.92 (27.82 to 272.53)	0.00% (-0.11% to 0.11%)
Germany	27746.71 (6784.05 to 107485.62)	56.65 (12.84 to 230.15)	0.01% (-0.18% to 0.19%)	43869.75 (13647.77 to 131154.59)	88.39 (26.43 to 278.10)	0.00% (-0.14% to 0.15%)
Greece	3161.62 (711.86 to 12335.57)	55.62 (11.25 to 230.15)	0.01% (-0.17% to 0.17%)	5110.44 (1563.99 to 16333.62)	83.24 (23.82 to 282.96)	0.00% (-0.11% to 0.12%)
Hungary	3371.13 (972.96 to 11085.44)	62.16 (16.62 to 222.24)	0.01% (-0.12% to 0.16%)	4969.09 (1701.31 to 14785.27)	83.67 (26.55 to 268.23)	0.01% (-0.12% to 0.13%)
Iceland	109.17 (25.25 to 437.82)	55.77 (11.96 to 230.05)	0.01% (-0.16% to 0.20%)	160.15 (48.42 to 529.26)	83.59 (24.58 to 282.87)	0.00% (-0.11% to 0.13%)
Ireland	1494.54 (324.41 to 5975.98)	55.58 (11.22 to 229.63)	0.00% (-0.17% to 0.20%)	2342.90 (700.55 to 7606.41)	83.14 (24.07 to 283.87)	0.00% (-0.13% to 0.11%)
Israel	2672.65 (567.26 to 10944.28)	55.87 (11.52 to 230.88)	0.01% (-0.17% to 0.22%)	4080.97 (1181.10 to 13658.33)	83.53 (23.77 to 284.49)	0.00% (-0.13% to 0.11%)
Italy	19669.24 (4438.99 to 82617.85)	58.16 (11.88 to 254.33)	0.01% (-0.08% to 0.10%)	30507.18 (9596.48 to 100133.40)	84.66 (24.78 to 294.53)	0.01% (-0.06% to 0.11%)
Latvia	720.45 (219.36 to 2362.17)	71.12 (20.09 to 248.91)	0.01% (-0.13% to 0.19%)	1231.57 (459.70 to 3300.97)	103.52 (36.19 to 292.33)	0.00% (-0.09% to 0.11%)
Lithuania	992.16 (281.68 to 3349.51)	67.24 (17.70 to 245.17)	0.01% (-0.13% to 0.15%)	1626.27 (569.24 to 4710.80)	93.94 (30.61 to 292.46)	0.00% (-0.11% to 0.10%)
Luxembourg	224.89 (54.88 to 850.44)	59.36 (13.70 to 236.48)	0.01% (-0.15% to 0.22%)	344.98 (117.88 to 995.93)	91.09 (29.94 to 280.56)	0.00% (-0.11% to 0.12%)
Malta	140.74 (31.03 to 556.28)	55.71 (11.23 to 230.18)	0.01% (-0.20% to 0.26%)	212.09 (63.99 to 678.53)	83.28 (24.34 to 283.78)	0.00% (-0.13% to 0.14%)
Monaco	11.75 (2.70 to 46.55)	55.78 (11.62 to 232.46)	0.00% (-0.16% to 0.23%)	18.87 (5.65 to 60.28)	83.54 (23.80 to 289.13)	0.00% (-0.11% to 0.14%)

Montenegro	217.48 (62.07 to 731.93)	62.38 (16.84 to 223.73)	0.00% (-0.12% to 0.12%)	303.02 (99.60 to 905.86)	83.59 (25.41 to 267.03)	0.00% (-0.13% to 0.11%)
Netherlands	5431.40 (1104.01 to 24980.94)	56.79 (10.20 to 268.89)	0.00% (-0.20% to 0.28%)	7795.28 (2204.70 to 29384.67)	79.55 (21.07 to 313.51)	0.00% (-0.15% to 0.16%)
North Macedonia	820.83 (237.89 to 2715.02)	62.25 (17.00 to 218.66)	0.00% (-0.14% to 0.19%)	1071.79 (364.76 to 3166.03)	83.52 (27.09 to 263.39)	0.00% (-0.11% to 0.13%)
Norway	1780.53 (394.34 to 7285.99)	58.05 (11.72 to 244.88)	0.04% (-0.08% to 0.17%)	2563.48 (718.21 to 9250.32)	85.05 (22.81 to 319.58)	0.03% (-0.06% to 0.15%)
Poland	14576.39 (4360.78 to 47048.08)	66.53 (18.45 to 230.27)	0.01% (-0.07% to 0.06%)	20864.24 (7203.50 to 62228.41)	89.37 (28.71 to 278.69)	0.01% (-0.07% to 0.05%)
Portugal	3235.78 (764.96 to 12673.79)	55.45 (11.58 to 230.33)	0.01% (-0.17% to 0.24%)	5480.17 (1641.37 to 17313.38)	83.17 (23.55 to 280.89)	0.01% (-0.11% to 0.12%)
Republic of Moldova	1471.12 (446.12 to 4808.76)	71.29 (20.43 to 247.57)	0.01% (-0.11% to 0.16%)	2398.44 (867.00 to 6415.98)	103.52 (36.20 to 288.81)	0.00% (-0.09% to 0.10%)
Romania	6724.99 (1898.65 to 22183.74)	62.21 (16.73 to 219.86)	0.01% (-0.13% to 0.16%)	9571.44 (3172.16 to 28734.90)	83.91 (25.84 to 268.04)	0.01% (-0.12% to 0.11%)
Russian Federation	62681.78 (20013.62 to 195485.51)	79.56 (24.13 to 263.24)	0.01% (-0.04% to 0.05%)	109309.44 (43244.49 to 269262.85)	117.97 (44.56 to 309.96)	0.00% (-0.02% to 0.03%)
San Marino	10.06 (2.32 to 40.04)	55.67 (11.80 to 233.52)	0.00% (-0.17% to 0.21%)	16.77 (5.22 to 53.66)	83.26 (24.57 to 283.78)	0.00% (-0.11% to 0.13%)
Serbia	3272.97 (942.64 to 11022.94)	62.59 (16.98 to 224.04)	0.00% (-0.14% to 0.14%)	4433.96 (1476.63 to 13222.23)	83.86 (26.49 to 266.47)	0.00% (-0.13% to 0.12%)
Slovakia	1953.89 (571.96 to 6494.10)	62.34 (17.13 to 222.66)	0.01% (-0.12% to 0.15%)	2751.71 (890.42 to 7970.61)	83.84 (25.31 to 265.84)	0.00% (-0.11% to 0.13%)
Slovenia	761.32 (226.19 to 2477.55)	62.30 (16.68 to 222.21)	0.01% (-0.14% to 0.17%)	1024.45 (353.07 to 2981.43)	83.93 (26.60 to 265.35)	0.01% (-0.10% to 0.13%)
Spain	14459.37 (3245.04 to 58174.80)	55.74 (11.32 to 233.67)	0.03% (-0.13% to 0.32%)	25479.55 (8416.87 to 75936.44)	91.40 (28.26 to 290.92)	0.00% (-0.13% to 0.16%)
Sweden	3143.50 (715.28 to 12568.87)	54.57 (11.70 to 220.82)	0.01% (-0.13% to 0.20%)	4706.58 (1361.10 to 15748.67)	82.58 (22.70 to 291.31)	0.00% (-0.14% to 0.13%)

Switzerland	2701.88 (657.54 to 10400.04)	52.72 (11.77 to 216.49)	0.04% (-0.18% to 0.24%)	4175.51 (1319.01 to 12666.79)	80.33 (24.46 to 262.00)	0.02% (-0.10% to 0.15%)
Ukraine	17994.08 (5546.67 to 57463.60)	74.81 (21.63 to 252.24)	0.00% (-0.10% to 0.13%)	30424.11 (11308.13 to 79935.04)	107.15 (37.44 to 302.59)	0.00% (-0.09% to 0.09%)
United Kingdom	21547.64 (4986.40 to 85157.88)	58.11 (12.48 to 238.83)	0.00% (-0.07% to 0.04%)	33640.25 (10314.71 to 109589.26)	85.96 (25.56 to 289.18)	0.00% (-0.06% to 0.03%)

Table S3k: Counts and age-standardized incidence rates of tension-type headache in 2021, and percentage changes from 1990 to 2021, by country and sex

	Male			Female		
	**Absolute number, 2021**	**Age-standardized rate, per 100 000 people, 2021**	**Percentage Change, 1990-2021**	**Absolute number, 2021**	**Age-standardized rate, per 100 000 people, 2021**	**Percentage Change, 1990-2021**
**Europe**	**45338106.97 (39635650.34 to 50694953.06)**	**10601.67 (9245.89 to 11946.76)**	**-0.01% (-0.02% to 0.00%)**	**49897119.51 (43827321.21 to 55512992.28)**	**11031.48 (9595.45 to 12389.90)**	**-0.01% (-0.02% to 0.00%)**
Albania	142984.17 (123982.03 to 160625.69)	10199.58 (8819.55 to 11548.48)	0.00% (-0.01% to 0.01%)	151195.06 (131315.64 to 168892.89)	10947.71 (9420.24 to 12318.32)	0.00% (-0.01% to 0.01%)
Andorra	5031.85 (4322.54 to 5712.20)	10710.53 (9219.66 to 12222.42)	0.00% (-0.01% to 0.01%)	5029.23 (4382.38 to 5696.17)	11282.50 (9699.53 to 12804.00)	0.00% (-0.01% to 0.01%)
Austria	492661.12 (428246.02 to 555571.18)	10701.66 (9168.44 to 12203.46)	0.00% (-0.01% to 0.01%)	527377.67 (461287.00 to 591982.22)	11167.06 (9715.02 to 12719.18)	0.00% (-0.01% to 0.01%)
Belarus	477492.39 (414746.21 to 537809.22)	10577.91 (9195.44 to 11976.66)	0.00% (-0.01% to 0.01%)	543932.44 (474787.77 to 610678.46)	10637.15 (9206.62 to 12023.60)	0.00% (-0.01% to 0.01%)
Belgium	619899.38 (541898.72 to 697645.86)	10710.45 (9235.74 to 12247.11)	0.00% (-0.01% to 0.01%)	673953.72 (595972.61 to 757827.35)	11282.41 (9728.34 to 12810.06)	0.00% (-0.01% to 0.01%)
Bosnia and Herzegovina	171627.29 (150033.37 to 193700.02)	10199.47 (8827.45 to 11573.91)	0.00% (-0.01% to 0.00%)	191730.77 (166996.09 to 213348.08)	10947.56 (9420.78 to 12327.86)	0.00% (-0.01% to 0.01%)
Bulgaria	347671.82 (303495.42 to 391329.77)	10199.64 (8849.39 to 11552.89)	0.00% (-0.01% to 0.01%)	393867.45 (343412.59 to 438989.49)	10948.19 (9432.93 to 12343.79)	0.00% (-0.01% to 0.01%)
Croatia	216490.04 (190353.80 to 242105.61)	10194.93 (8880.69 to 11517.25)	0.00% (-0.04% to 0.04%)	245835.36 (217468.31 to 275398.69)	10989.76 (9557.24 to 12378.04)	0.00% (-0.04% to 0.05%)
Cyprus	75313.91 (64639.01 to 85677.67)	10710.77 (9218.76 to 12252.55)	0.00% (-0.01% to 0.01%)	82246.61 (71924.68 to 93085.21)	11282.54 (9701.00 to 12825.03)	0.00% (-0.01% to 0.00%)

Czechia	548901.18 (480783.13 to 617377.86)	10199.65 (8823.66 to 11547.30)	0.00% (-0.01% to 0.01%)	604681.87 (528127.57 to 673913.93)	10947.80 (9421.13 to 12329.17)	0.00% (-0.01% to 0.01%)
Denmark	320349.47 (278312.22 to 359422.33)	10722.01 (9222.10 to 12087.45)	0.00% (-0.04% to 0.04%)	342977.60 (294815.84 to 386451.32)	11385.98 (9760.85 to 12971.36)	0.01% (-0.05% to 0.07%)
Estonia	67645.23 (59017.73 to 76056.50)	10578.27 (9197.20 to 11952.86)	0.00% (-0.01% to 0.01%)	74853.81 (65820.80 to 83366.04)	10637.05 (9195.76 to 11991.45)	0.00% (-0.01% to 0.01%)
Finland	300486.18 (262504.87 to 336968.39)	10710.61 (9203.40 to 12251.34)	0.00% (-0.01% to 0.01%)	323845.87 (287134.23 to 363395.97)	11282.82 (9710.17 to 12793.68)	0.00% (0.00% to 0.01%)
France	3489206.79 (3068925.23 to 3921140.27)	10666.22 (9223.19 to 12104.16)	0.00% (-0.01% to 0.01%)	3874386.89 (3410055.70 to 4312012.07)	11106.98 (9617.33 to 12662.34)	0.00% (-0.01% to 0.01%)
Germany	4688453.04 (4064388.63 to 5266123.59)	10726.77 (9231.72 to 12304.93)	0.00% (-0.04% to 0.06%)	4942149.01 (4304413.48 to 5632421.88)	11308.92 (9795.98 to 12872.16)	0.00% (-0.04% to 0.05%)
Greece	542690.49 (473338.34 to 608495.00)	10710.62 (9215.60 to 12276.10)	0.00% (-0.01% to 0.01%)	607890.72 (535574.53 to 682305.19)	11282.36 (9700.95 to 12815.70)	0.00% (-0.01% to 0.01%)
Hungary	485278.08 (425741.67 to 546745.35)	10199.49 (8827.84 to 11526.12)	0.00% (-0.01% to 0.01%)	566960.75 (495626.41 to 631358.71)	10947.93 (9418.02 to 12343.93)	0.00% (-0.01% to 0.01%)
Iceland	19575.45 (17012.01 to 22168.14)	10710.72 (9210.83 to 12242.53)	0.00% (-0.01% to 0.01%)	20075.59 (17516.86 to 22544.56)	11282.22 (9687.57 to 12828.53)	0.00% (-0.01% to 0.01%)
Ireland	267623.98 (233630.06 to 302429.45)	10710.40 (9211.70 to 12242.78)	0.00% (-0.01% to 0.01%)	290844.92 (254310.32 to 326581.86)	11282.53 (9703.81 to 12786.30)	0.00% (-0.01% to 0.01%)
Israel	506554.20 (437600.77 to 575503.25)	10710.31 (9211.60 to 12244.34)	0.00% (-0.01% to 0.01%)	539051.89 (469243.55 to 607898.46)	11282.46 (9683.78 to 12789.29)	0.00% (-0.01% to 0.01%)
Italy	3494775.35 (3054206.65 to 3908595.80)	11654.45 (10132.15 to 13129.68)	0.00% (-0.03% to 0.04%)	3835625.55 (3382231.37 to 4252572.08)	12155.73 (10603.87 to 13601.81)	0.00% (-0.04% to 0.03%)
Latvia	93619.49 (81854.66 to 105337.19)	10578.21 (9191.54 to 11980.76)	0.00% (0.00% to 0.01%)	108670.89 (95347.10 to 121397.07)	10637.06 (9189.64 to 12010.49)	0.00% (0.00% to 0.01%)

Lithuania	139282.73 (120903.56 to 157776.93)	10746.82 (9221.04 to 12239.33)	0.00% (-0.01% to 0.01%)	160886.38 (141043.52 to 179323.62)	10772.99 (9320.13 to 12213.78)	0.00% (-0.01% to 0.00%)
Luxembourg	36530.58 (31673.75 to 41561.53)	10724.56 (9216.64 to 12169.56)	0.00% (-0.01% to 0.01%)	37780.26 (32632.24 to 42732.93)	11216.87 (9639.29 to 12751.12)	0.00% (0.00% to 0.01%)
Malta	24236.92 (21024.25 to 27250.00)	10710.35 (9219.83 to 12268.75)	0.00% (-0.01% to 0.01%)	25552.63 (22651.48 to 28676.02)	11282.31 (9752.47 to 12795.30)	0.00% (-0.01% to 0.01%)
Monaco	1982.44 (1729.38 to 2226.47)	10710.62 (9213.19 to 12256.90)	0.00% (-0.01% to 0.01%)	2228.04 (1967.41 to 2500.39)	11282.21 (9686.72 to 12831.67)	0.00% (-0.01% to 0.01%)
Montenegro	32269.12 (28101.64 to 36507.97)	10199.56 (8824.49 to 11561.35)	0.00% (0.00% to 0.01%)	35392.29 (30845.06 to 39461.33)	10947.62 (9414.66 to 12340.79)	0.00% (-0.01% to 0.00%)
Netherlands	947614.97 (827369.89 to 1073587.83)	10848.95 (9200.27 to 12455.53)	0.00% (-0.01% to 0.01%)	1013504.15 (882328.61 to 1144953.57)	11377.64 (9608.58 to 13036.64)	0.00% (-0.01% to 0.01%)
North Macedonia	119281.29 (104031.76 to 135169.91)	10199.55 (8826.74 to 11564.02)	0.00% (-0.01% to 0.01%)	123612.91 (107452.71 to 138603.67)	10947.69 (9417.75 to 12335.71)	0.00% (-0.01% to 0.00%)
Norway	333496.01 (294549.99 to 373986.97)	11800.12 (10401.90 to 13303.11)	0.02% (-0.02% to 0.06%)	349964.85 (302979.70 to 391877.01)	12665.85 (10929.87 to 14278.29)	0.00% (-0.05% to 0.05%)
Poland	2115569.80 (1847967.19 to 2365704.97)	10944.84 (9590.60 to 12281.96)	0.00% (0.00% to 0.00%)	2410144.82 (2110311.61 to 2679346.59)	11776.68 (10203.65 to 13237.29)	0.00% (0.00% to 0.00%)
Portugal	555467.99 (484385.39 to 622742.52)	10710.44 (9235.28 to 12255.19)	0.00% (-0.01% to 0.01%)	649310.92 (572363.75 to 729097.68)	11282.68 (9729.74 to 12788.23)	0.00% (-0.01% to 0.01%)
Republic of Moldova	192364.24 (166796.87 to 217302.39)	10578.05 (9180.46 to 11983.80)	0.00% (-0.01% to 0.01%)	210113.67 (183386.49 to 237153.92)	10636.86 (9184.34 to 12012.49)	0.00% (-0.01% to 0.01%)
Romania	972735.49 (854831.74 to 1094231.44)	10199.69 (8825.76 to 11530.53)	0.00% (-0.01% to 0.01%)	1096093.41 (956668.83 to 1221453.98)	10947.76 (9417.27 to 12329.62)	0.00% (-0.01% to 0.01%)
Russian Federation	7751157.40 (6747137.64 to 8665152.75)	11088.07 (9651.69 to 12388.18)	0.00% (-0.04% to 0.04%)	8959134.36 (7809549.89 to 10010286.93)	11298.64 (9892.07 to 12710.82)	0.01% (-0.04% to 0.05%)

San Marino	1741.37 (1529.45 to 1956.72)	10710.61 (9215.38 to 12258.43)	0.00% (-0.01% to 0.01%)	1989.66 (1750.97 to 2243.39)	11282.24 (9684.36 to 12816.99)	0.00% (-0.01% to 0.01%)
Serbia	480651.97 (419249.16 to 541727.22)	10199.62 (8831.72 to 11537.48)	0.00% (-0.01% to 0.01%)	513387.05 (449628.06 to 572468.45)	10947.72 (9428.38 to 12336.31)	0.00% (0.00% to 0.01%)
Slovakia	281983.50 (246468.03 to 318808.51)	10199.68 (8822.14 to 11535.30)	0.00% (-0.01% to 0.01%)	314783.67 (275244.28 to 351321.57)	10947.71 (9427.35 to 12326.79)	0.00% (-0.01% to 0.01%)
Slovenia	108110.63 (94598.77 to 121645.29)	10199.40 (8823.15 to 11555.80)	0.00% (-0.01% to 0.01%)	116706.07 (102333.77 to 129662.20)	10948.08 (9413.71 to 12330.20)	0.00% (-0.01% to 0.01%)
Spain	2467662.85 (2134475.83 to 2776687.41)	10700.65 (9151.58 to 12132.70)	0.00% (-0.01% to 0.01%)	2678698.15 (2348469.35 to 3008586.85)	11084.23 (9650.25 to 12493.53)	0.00% (0.00% to 0.01%)
Sweden	610898.31 (537204.60 to 676187.62)	11485.53 (10042.81 to 12847.14)	0.01% (-0.03% to 0.04%)	652757.18 (571880.76 to 727505.08)	12445.65 (10861.59 to 14016.10)	0.00% (-0.04% to 0.04%)
Switzerland	480769.55 (418315.16 to 537980.21)	10413.42 (9030.64 to 11720.72)	0.01% (-0.03% to 0.07%)	511072.53 (448930.46 to 568056.97)	11018.46 (9457.41 to 12374.00)	0.01% (-0.04% to 0.06%)
Ukraine	2403869.80 (2101236.38 to 2710731.50)	11381.86 (9943.97 to 12793.28)	0.00% (-0.01% to 0.01%)	2748687.50 (2415132.52 to 3078681.35)	11419.25 (9924.74 to 12878.41)	0.00% (-0.01% to 0.01%)
United Kingdom	3921065.02 (3449765.71 to 4386838.85)	11495.59 (10022.64 to 12930.13)	0.00% (0.00% to 0.00%)	4326591.06 (3793026.15 to 4838917.05)	12117.18 (10549.45 to 13627.58)	0.00% (0.00% to 0.00%)

Table S3I: Counts and age-standardized YLD rates of tension-type headache in 2021, and percentage changes from 1990 to 2021, by country and sex

	Male			Female		
	**Absolute number, 2021**	**Age-standardized rate, per 100 000 people, 2021**	**Percentage Change, 1990-2021**	**Absolute number, 2021**	**Age-standardized rate, per 100 000 people, 2021**	**Percentage Change, 1990-2021**
**Europe**	**297654.68 (83195.64 to 1044194.41)**	**62.87 (16.40 to 232.91)**	**0.00% (-0.06% to 0.03%)**	**470322.34 (162163.34 to 1359954.49)**	**92.26 (30.36 to 280.15)**	**-0.01% (-0.05% to 0.01%)**
Albania	951.50 (265.62 to 3254.72)	62.15 (16.59 to 219.67)	0.01% (-0.13% to 0.15%)	1300.24 (430.81 to 3875.29)	83.88 (26.64 to 264.81)	0.00% (-0.10% to 0.13%)
Andorra	30.00 (6.79 to 118.16)	55.69 (11.20 to 229.80)	0.00% (-0.17% to 0.20%)	43.16 (13.03 to 139.38)	83.33 (23.51 to 288.42)	0.00% (-0.13% to 0.10%)
Austria	3019.30 (723.28 to 12261.99)	59.07 (13.14 to 248.86)	0.00% (-0.17% to 0.21%)	4424.68 (1415.89 to 13946.15)	83.44 (24.87 to 281.00)	0.00% (-0.14% to 0.14%)
Belarus	3647.45 (1068.44 to 11746.48)	71.21 (19.64 to 247.08)	0.01% (-0.12% to 0.16%)	6213.38 (2262.44 to 16489.58)	103.42 (34.82 to 292.34)	0.00% (-0.09% to 0.09%)
Belgium	3526.05 (811.17 to 14261.41)	55.60 (11.68 to 232.63)	0.00% (-0.18% to 0.24%)	5468.74 (1651.21 to 17717.23)	83.20 (24.04 to 283.30)	0.00% (-0.13% to 0.11%)
Bosnia and Herzegovina	1187.67 (350.51 to 3906.06)	62.00 (17.24 to 216.93)	0.01% (-0.15% to 0.14%)	1679.50 (572.15 to 4874.67)	83.26 (26.56 to 261.67)	0.00% (-0.13% to 0.12%)
Bulgaria	2440.40 (695.07 to 8031.90)	62.19 (16.50 to 220.42)	0.01% (-0.13% to 0.17%)	3466.25 (1140.02 to 10233.12)	83.46 (25.66 to 263.51)	0.00% (-0.12% to 0.10%)
Croatia	1510.56 (444.46 to 4960.61)	62.31 (16.79 to 222.14)	0.01% (-0.13% to 0.13%)	2146.54 (722.99 to 6468.99)	83.78 (25.97 to 269.17)	0.00% (-0.12% to 0.11%)
Cyprus	425.88 (93.67 to 1697.77)	55.68 (11.79 to 228.91)	0.01% (-0.22% to 0.18%)	685.12 (203.07 to 2235.29)	83.43 (23.81 to 288.71)	0.00% (-0.13% to 0.11%)
Czechia	3818.49 (1077.78 to 12475.21)	62.18 (16.29 to 223.62)	0.01% (-0.14% to 0.14%)	5235.99 (1765.68 to 15681.31)	83.50 (25.99 to 269.46)	0.00% (-0.12% to 0.13%)
Denmark	1865.51 (427.83 to 7431.85)	57.15 (11.94 to 233.08)	0.00% (-0.18% to 0.22%)	2767.13 (761.22 to 9684.86)	83.77 (22.39 to 300.34)	-0.01% (-0.16% to 0.13%)

Estonia	516.79 (152.26 to 1677.48)	71.39 (19.83 to 246.06)	0.01% (-0.11% to 0.13%)	833.21 (308.63 to 2260.49)	103.74 (35.70 to 296.32)	0.00% (-0.10% to 0.10%)
Finland	1708.83 (389.20 to 6812.26)	55.52 (11.69 to 230.71)	0.01% (-0.19% to 0.20%)	2627.59 (810.06 to 8441.99)	83.35 (24.75 to 280.78)	0.00% (-0.12% to 0.13%)
France	21185.62 (4989.61 to 78003.39)	59.33 (13.59 to 229.31)	0.00% (-0.17% to 0.18%)	34637.35 (11360.81 to 100985.71)	89.92 (27.82 to 272.53)	0.00% (-0.11% to 0.11%)
Germany	27746.71 (6784.05 to 107485.62)	56.65 (12.84 to 230.15)	0.01% (-0.18% to 0.19%)	43869.75 (13647.77 to 131154.59)	88.39 (26.43 to 278.10)	0.00% (-0.14% to 0.15%)
Greece	3161.62 (711.86 to 12335.57)	55.62 (11.25 to 230.15)	0.01% (-0.17% to 0.17%)	5110.44 (1563.99 to 16333.62)	83.24 (23.82 to 282.96)	0.00% (-0.11% to 0.12%)
Hungary	3371.13 (972.96 to 11085.44)	62.16 (16.62 to 222.24)	0.01% (-0.12% to 0.16%)	4969.09 (1701.31 to 14785.27)	83.67 (26.55 to 268.23)	0.01% (-0.12% to 0.13%)
Iceland	109.17 (25.25 to 437.82)	55.77 (11.96 to 230.05)	0.01% (-0.16% to 0.20%)	160.15 (48.42 to 529.26)	83.59 (24.58 to 282.87)	0.00% (-0.11% to 0.13%)
Ireland	1494.54 (324.41 to 5975.98)	55.58 (11.22 to 229.63)	0.00% (-0.17% to 0.20%)	2342.90 (700.55 to 7606.41)	83.14 (24.07 to 283.87)	0.00% (-0.13% to 0.11%)
Israel	2672.65 (567.26 to 10944.28)	55.87 (11.52 to 230.88)	0.01% (-0.17% to 0.22%)	4080.97 (1181.10 to 13658.33)	83.53 (23.77 to 284.49)	0.00% (-0.13% to 0.11%)
Italy	19669.24 (4438.99 to 82617.85)	58.16 (11.88 to 254.33)	0.01% (-0.08% to 0.10%)	30507.18 (9596.48 to 100133.40)	84.66 (24.78 to 294.53)	0.01% (-0.06% to 0.11%)
Latvia	720.45 (219.36 to 2362.17)	71.12 (20.09 to 248.91)	0.01% (-0.13% to 0.19%)	1231.57 (459.70 to 3300.97)	103.52 (36.19 to 292.33)	0.00% (-0.09% to 0.11%)
Lithuania	992.16 (281.68 to 3349.51)	67.24 (17.70 to 245.17)	0.01% (-0.13% to 0.15%)	1626.27 (569.24 to 4710.80)	93.94 (30.61 to 292.46)	0.00% (-0.11% to 0.10%)
Luxembourg	224.89 (54.88 to 850.44)	59.36 (13.70 to 236.48)	0.01% (-0.15% to 0.22%)	344.98 (117.88 to 995.93)	91.09 (29.94 to 280.56)	0.00% (-0.11% to 0.12%)
Malta	140.74 (31.03 to 556.28)	55.71 (11.23 to 230.18)	0.01% (-0.20% to 0.26%)	212.09 (63.99 to 678.53)	83.28 (24.34 to 283.78)	0.00% (-0.13% to 0.14%)
Monaco	11.75 (2.70 to 46.55)	55.78 (11.62 to 232.46)	0.00% (-0.16% to 0.23%)	18.87 (5.65 to 60.28)	83.54 (23.80 to 289.13)	0.00% (-0.11% to 0.14%)

Montenegro	217.48<br>(62.07 to 731.93)	62.38<br>(16.84 to 223.73)	0.00%<br>(-0.12% to 0.12%)	303.02<br>(99.60 to 905.86)	83.59<br>(25.41 to 267.03)	0.00%<br>(-0.13% to 0.11%)
Netherlands	5431.40<br>(1104.01 to 24980.94)	56.79<br>(10.20 to 268.89)	0.00%<br>(-0.20% to 0.28%)	7795.28<br>(2204.70 to 29384.67)	79.55<br>(21.07 to 313.51)	0.00%<br>(-0.15% to 0.16%)
North Macedonia	820.83<br>(237.89 to 2715.02)	62.25<br>(17.00 to 218.66)	0.00%<br>(-0.14% to 0.19%)	1071.79<br>(364.76 to 3166.03)	83.52<br>(27.09 to 263.39)	0.00%<br>(-0.11% to 0.13%)
Norway	1780.53<br>(394.34 to 7285.99)	58.05<br>(11.72 to 244.88)	0.04%<br>(-0.08% to 0.17%)	2563.48<br>(718.21 to 9250.32)	85.05<br>(22.81 to 319.58)	0.03%<br>(-0.06% to 0.15%)
Poland	14576.39<br>(4360.78 to 47048.08)	66.53<br>(18.45 to 230.27)	0.01%<br>(-0.07% to 0.06%)	20864.24<br>(7203.50 to 62228.41)	89.37<br>(28.71 to 278.69)	0.01%<br>(-0.07% to 0.05%)
Portugal	3235.78<br>(764.96 to 12673.79)	55.45<br>(11.58 to 230.33)	0.01%<br>(-0.17% to 0.24%)	5480.17<br>(1641.37 to 17313.38)	83.17<br>(23.55 to 280.89)	0.01%<br>(-0.11% to 0.12%)
Republic of Moldova	1471.12<br>(446.12 to 4808.76)	71.29<br>(20.43 to 247.57)	0.01%<br>(-0.11% to 0.16%)	2398.44<br>(867.00 to 6415.98)	103.52<br>(36.20 to 288.81)	0.00%<br>(-0.09% to 0.10%)
Romania	6724.99<br>(1898.65 to 22183.74)	62.21<br>(16.73 to 219.86)	0.01%<br>(-0.13% to 0.16%)	9571.44<br>(3172.16 to 28734.90)	83.91<br>(25.84 to 268.04)	0.01%<br>(-0.12% to 0.11%)
Russian Federation	62681.78<br>(20013.62 to 195485.51)	79.56<br>(24.13 to 263.24)	0.01%<br>(-0.04% to 0.05%)	109309.44<br>(43244.49 to 269262.85)	117.97<br>(44.56 to 309.96)	0.00%<br>(-0.02% to 0.03%)
San Marino	10.06<br>(2.32 to 40.04)	55.67<br>(11.80 to 233.52)	0.00%<br>(-0.17% to 0.21%)	16.77<br>(5.22 to 53.66)	83.26<br>(24.57 to 283.78)	0.00%<br>(-0.11% to 0.13%)
Serbia	3272.97<br>(942.64 to 11022.94)	62.59<br>(16.98 to 224.04)	0.00%<br>(-0.14% to 0.14%)	4433.96<br>(1476.63 to 13222.23)	83.86<br>(26.49 to 266.47)	0.00%<br>(-0.13% to 0.12%)
Slovakia	1953.89<br>(571.96 to 6494.10)	62.34<br>(17.13 to 222.66)	0.01%<br>(-0.12% to 0.15%)	2751.71<br>(890.42 to 7970.61)	83.84<br>(25.31 to 265.84)	0.00%<br>(-0.11% to 0.13%)
Slovenia	761.32<br>(226.19 to 2477.55)	62.30<br>(16.68 to 222.21)	0.01%<br>(-0.14% to 0.17%)	1024.45<br>(353.07 to 2981.43)	83.93<br>(26.60 to 265.35)	0.01%<br>(-0.10% to 0.13%)
Spain	14459.37<br>(3245.04 to 58174.80)	55.74<br>(11.32 to 233.67)	0.03%<br>(-0.13% to 0.32%)	25479.55<br>(8416.87 to 75936.44)	91.40<br>(28.26 to 290.92)	0.00%<br>(-0.13% to 0.16%)
Sweden	3143.50<br>(715.28 to 12568.87)	54.57<br>(11.70 to 220.82)	0.01%<br>(-0.13% to 0.20%)	4706.58<br>(1361.10 to 15748.67)	82.58<br>(22.70 to 291.31)	0.00%<br>(-0.14% to 0.13%)

Switzerland	2701.88 (657.54 to 10400.04)	52.72 (11.77 to 216.49)	0.04% (-0.18% to 0.24%)	4175.51 (1319.01 to 12666.79)	80.33 (24.46 to 262.00)	0.02% (-0.10% to 0.15%)
Ukraine	17994.08 (5546.67 to 57463.60)	74.81 (21.63 to 252.24)	0.00% (-0.10% to 0.13%)	30424.11 (11308.13 to 79935.04)	107.15 (37.44 to 302.59)	0.00% (-0.09% to 0.09%)
United Kingdom	21547.64 (4986.40 to 85157.88)	58.11 (12.48 to 238.83)	0.00% (-0.07% to 0.04%)	33640.25 (10314.71 to 109589.26)	85.96 (25.56 to 289.18)	0.00% (-0.06% to 0.03%)

Table S4a: Prevalence, DALY, and incidence rates of headache disorders, by age and sex, 2021

	Prevalence (95% UI)		DALYs (95% UI)		Incidence (95% UI)	
	**Male**	**Female**	**Male**	**Female**	**Male**	**Female**
**<5 years**	0.00 (0.00 to 0.00)	0.00 (0.00 to 0.00)	0.00 (0.00 to 0.00)	0.00 (0.00 to 0.00)	0.00 (0.00 to 0.00)	0.00 (0.00 to 0.00)
**5 to 9**	9802.81 (6692.45 to 13677.53)	10824.92 (7801.45 to 14792.33)	77.85 (3.38 to 206.01)	126.36 (4.63 to 338.70)	8221.42 (5605.40 to 11681.07)	8864.78 (6323.49 to 12293.59)
**10 to 14**	35256.48 (27597.62 to 43881.32)	40328.09 (32775.20 to 48757.59)	385.35 (29.23 to 931.90)	645.05 (44.18 to 1533.57)	14258.84 (9714.75 to 19391.43)	16246.01 (11348.35 to 21487.23)
**15 to 19**	44556.55 (33350.55 to 55319.53)	53728.30 (43522.61 to 63507.79)	570.20 (73.65 to 1326.28)	1038.76 (134.19 to 2428.99)	14054.76 (9391.89 to 19553.59)	16214.27 (11275.47 to 22303.84)
**20 to 24**	47264.64 (38228.44 to 59280.75)	58375.66 (49595.42 to 68615.85)	631.57 (93.81 to 1485.29)	1235.90 (183.77 to 2756.13)	14151.96 (9308.19 to 19878.71)	16147.62 (10900.58 to 22420.34)
**25 to 29**	49931.13 (40130.43 to 61435.05)	61097.68 (52409.10 to 71711.60)	650.82 (104.87 to 1469.46)	1285.75 (201.46 to 2811.88)	15740.07 (10869.76 to 21970.40)	17111.96 (11988.28 to 23647.65)
**30 to 34**	53236.14 (42821.73 to 64986.16)	63195.68 (53273.98 to 73671.73)	683.74 (140.06 to 1448.93)	1330.24 (272.19 to 2820.83)	15553.81 (10478.14 to 20816.38)	16753.68 (11447.68 to 22301.34)
**35 to 39**	51900.89 (40828.01 to 63932.16)	62379.25 (52197.56 to 72899.58)	725.15 (198.59 to 1522.27)	1400.24 (368.41 to 2831.92)	14431.77 (9611.12 to 19995.70)	15789.69 (10737.96 to 21563.21)
**40 to 44**	49976.25 (40258.58 to 62484.68)	60950.89 (51783.06 to 71723.55)	756.33 (217.47 to 1568.07)	1453.89 (375.62 to 3049.19)	13561.92 (8666.92 to 18760.33)	14721.82 (9737.18 to 20299.83)
**45 to 49**	46895.56 (37883.28 to 57300.25)	57748.53 (48969.82 to 67086.18)	720.43 (217.08 to 1431.48)	1375.74 (392.54 to 2723.43)	12168.94 (8015.05 to 17095.11)	13200.74 (8701.37 to 18452.01)
**50 to 54**	44792.60 (34704.41 to 55987.44)	55161.44 (45599.17 to 65469.47)	662.67 (213.63 to 1334.26)	1262.35 (394.21 to 2396.64)	12372.20 (8220.18 to 17083.38)	13313.65 (8956.29 to 18291.07)
**55 to 59**	43723.95 (34069.98 to 55949.24)	52493.79 (43167.20 to 63367.68)	599.61 (210.39 to 1170.25)	1121.97 (394.05 to 2222.90)	11114.89 (7475.54 to 15863.36)	11676.06 (7865.77 to 16570.99)
**60 to 64**	38780.91 (29596.68 to 50287.51)	46464.55 (37485.92 to 57350.96)	547.91 (219.26 to 1060.84)	992.68 (388.29 to 1834.51)	9923.00 (6359.80 to 14794.44)	10555.51 (6833.53 to 15816.57)
**65 to 69**	36965.17 (28437.80 to 48320.43)	44432.72 (35724.06 to 55122.19)	455.86 (184.29 to 871.28)	798.59 (343.33 to 1477.47)	10689.92 (6796.24 to 15831.28)	12025.80 (7761.83 to 17639.90)
**70 to 74**	35089.17 (26728.14 to 47075.83)	42456.79 (33760.87 to 54378.74)	351.66 (128.46 to 676.10)	599.75 (226.90 to 1165.81)	9655.86 (6368.91 to 13449.12)	10904.96 (7353.18 to 15142.57)
**75 to 79**	31190.79 (21287.67 to 41773.39)	37209.36 (27001.64 to 47992.44)	279.40 (93.87 to 583.20)	466.76 (160.76 to 949.32)	8754.71 (5705.34 to 12385.81)	9680.47 (6383.33 to 13580.45)
**80 to 84**	30426.14 (22052.46 to 41003.56)	35822.57 (26725.25 to 47074.45)	249.69 (86.59 to 520.95)	405.50 (150.11 to 802.89)	8876.85 (5775.38 to 12372.53)	9935.48 (6511.53 to 13802.37)

**85 to 89**	28119.17 (19890.95 to 38862.67)	33132.89 (24093.29 to 44122.85)	222.35 (84.78 to 447.50)	353.58 (139.45 to 672.63)	7624.73 (4700.84 to 11231.10)	8512.98 (5233.40 to 12410.20)
**90 to 94**	26894.21 (18680.84 to 37191.10)	30409.14 (21980.51 to 41282.16)	182.15 (65.03 to 368.57)	275.24 (106.68 to 541.84)	9174.92 (5864.11 to 13125.17)	9599.19 (6107.45 to 13665.45)
**95 plus**	35037.81 (23581.28 to 48066.61)	37098.14 (25690.30 to 50202.58)	131.94 (30.43 to 319.55)	190.29 (46.45 to 430.92)	12757.19 (6913.68 to 19295.43)	12896.11 (7019.66 to 19665.43)

Table S4b: Prevalence, DALY, and incidence rates of migraine, by age and sex, 2021

	Prevalence (95% UI)		DALYs (95% UI)		Incidence (95% UI)	
	Male	Female	Male	Female	Male	Female
**<5 years**	0.00 (0.00 to 0.00)	0.00 (0.00 to 0.00)	0.00 (0.00 to 0.00)	0.00 (0.00 to 0.00)	0.00 (0.00 to 0.00)	0.00 (0.00 to 0.00)
**5 to 9**	2023.59 (1312.76 to 2878.76)	3474.16 (2217.68 to 4962.30)	68.47 (1.84 to 191.13)	116.89 (2.79 to 330.27)	1281.14 (830.45 to 1854.65)	2204.21 (1404.86 to 3161.03)
**10 to 14**	9588.32 (6913.81 to 12992.65)	16670.26 (12204.92 to 22294.28)	348.61 (17.81 to 861.12)	603.38 (28.44 to 1494.34)	1878.29 (1393.05 to 2464.90)	3397.65 (2438.45 to 4508.84)
**15 to 19**	13717.34 (10197.61 to 17742.68)	25781.16 (19115.20 to 32505.81)	511.81 (50.95 to 1211.79)	960.30 (90.71 to 2337.87)	1231.57 (708.69 to 1926.79)	2637.59 (1528.10 to 4021.67)
**20 to 24**	15209.05 (11442.65 to 19301.51)	30550.33 (23482.56 to 38361.66)	563.75 (63.91 to 1400.19)	1137.58 (127.65 to 2650.13)	1121.27 (670.18 to 1688.80)	2326.28 (1405.80 to 3443.17)
**25 to 29**	15772.07 (12315.82 to 19996.51)	32049.72 (25824.89 to 39672.18)	578.69 (64.78 to 1372.57)	1180.70 (130.80 to 2723.06)	1082.10 (649.40 to 1639.87)	2007.21 (1199.09 to 2964.41)
**30 to 34**	15984.59 (12443.60 to 20424.14)	32087.85 (25594.51 to 40249.92)	598.75 (89.66 to 1356.15)	1205.17 (172.35 to 2684.24)	1018.58 (612.26 to 1608.17)	1839.07 (1139.47 to 2811.06)
**35 to 39**	16263.43 (12945.70 to 20171.29)	32500.29 (26297.41 to 40154.19)	629.54 (130.19 to 1379.91)	1255.18 (247.61 to 2636.88)	1072.41 (665.43 to 1689.05)	2045.11 (1291.85 to 3116.69)
**40 to 44**	16520.23 (13214.92 to 20887.62)	33112.77 (26713.93 to 40992.93)	655.49 (147.55 to 1428.52)	1299.40 (273.21 to 2820.26)	903.93 (556.13 to 1334.65)	1721.12 (1076.58 to 2505.17)
**45 to 49**	15331.97 (12162.09 to 19456.45)	30547.90 (24395.34 to 38192.04)	619.02 (154.90 to 1287.06)	1218.29 (281.00 to 2502.28)	641.78 (368.15 to 962.00)	1139.18 (657.47 to 1739.49)
**50 to 54**	13932.95 (11318.62 to 17760.67)	27488.74 (22324.42 to 34529.29)	564.45 (146.64 to 1190.22)	1107.09 (278.45 to 2258.06)	553.58 (317.37 to 857.60)	968.00 (554.17 to 1480.94)
**55 to 59**	12306.09 (9879.45 to 15698.40)	23971.23 (19249.33 to 30340.67)	502.50 (146.81 to 1058.65)	971.98 (271.04 to 2034.17)	415.18 (241.14 to 638.07)	708.91 (411.57 to 1104.32)
**60 to 64**	10685.77 (8573.32 to 13512.29)	20224.71 (16319.97 to 25471.26)	451.79 (149.71 to 904.10)	845.76 (273.64 to 1666.74)	282.06 (162.09 to 440.49)	419.59 (246.23 to 662.78)
**65 to 69**	8823.64 (6937.95 to 11205.14)	16106.73 (12647.39 to 20337.04)	370.07 (120.63 to 756.32)	670.48 (213.00 to 1333.81)	223.90 (123.82 to 327.55)	276.99 (151.20 to 414.15)
**70 to 74**	7144.90 (5591.27 to 9177.06)	12552.72 (9793.83 to 16116.40)	283.41 (90.94 to 589.32)	499.99 (159.07 to 1024.12)	216.60 (129.57 to 330.64)	262.03 (158.52 to 405.30)
**75 to 79**	5939.79 (4617.89 to 7591.86)	10133.43 (7834.45 to 13179.29)	224.84 (65.02 to 479.09)	388.77 (114.22 to 805.60)	217.36 (136.83 to 332.54)	278.73 (175.25 to 426.70)
**80 to 84**	5354.78 (4196.23 to 7000.20)	8818.29 (6865.57 to 11577.09)	199.58 (61.31 to 427.29)	334.21 (107.52 to 696.46)	212.43 (128.55 to 326.22)	284.91 (177.95 to 438.02)
**85 to 89**	4782.43 (3675.36 to 6106.09)	7698.76 (5871.24 to 9983.55)	176.66 (61.86 to 360.39)	289.38 (102.80 to 567.16)	167.22 (103.35 to 249.82)	217.56 (132.18 to 330.05)

**90 to 94**	4027.63 (3132.98 to 5177.56)	6259.67 (4831.51 to 8138.41)	143.51 (41.83 to 289.41)	224.21 (68.41 to 458.27)	102.15 (68.30 to 146.20)	121.16 (80.21 to 174.00)
**95 plus**	2989.26 (2191.19 to 4025.82)	4624.74 (3331.58 to 6350.98)	95.14 (18.83 to 221.86)	147.39 (30.67 to 330.93)	25.29 (16.95 to 36.29)	30.35 (20.04 to 43.72)

Table S4c: Prevalence, DALY, and incidence rates of tension-type headache, by age and sex, 2021

	Prevalence (95% UI)		DALYs (95% UI)		Incidence (95% UI)	
	**Male**	**Female**	**Male**	**Female**	**Male**	**Female**
**<5 years**	0.00 (0.00 to 0.00)	0.00 (0.00 to 0.00)	0.00 (0.00 to 0.00)	0.00 (0.00 to 0.00)	0.00 (0.00 to 0.00)	0.00 (0.00 to 0.00)
**5 to 9**	7939.32 (4749.88 to 11883.15)	7611.82 (4482.00 to 11351.93)	9.38 (0.42 to 55.37)	9.47 (0.56 to 56.17)	6940.28 (4318.93 to 10532.19)	6660.58 (4085.70 to 10018.39)
**10 to 14**	28374.82 (20502.47 to 37501.09)	28354.11 (20285.35 to 36914.35)	36.74 (3.51 to 202.75)	41.67 (5.40 to 208.53)	12380.55 (7923.46 to 17451.75)	12848.36 (8115.56 to 18038.76)
**15 to 19**	35723.71 (22721.77 to 48235.43)	37718.03 (24474.62 to 50369.93)	58.39 (9.14 to 273.69)	78.47 (17.29 to 312.96)	12823.20 (8149.97 to 18083.97)	13576.68 (8644.84 to 19272.00)
**20 to 24**	37827.14 (27506.78 to 51827.20)	40262.30 (29212.61 to 54650.69)	67.83 (12.86 to 303.05)	98.32 (25.42 to 341.53)	13030.69 (8088.37 to 18658.88)	13821.33 (8589.67 to 20033.30)
**25 to 29**	40585.42 (28826.45 to 54301.22)	42919.24 (30881.66 to 57359.58)	72.13 (13.22 to 322.34)	105.05 (25.81 to 393.35)	14657.97 (9707.18 to 20552.07)	15104.76 (9963.85 to 21192.74)
**30 to 34**	44399.86 (31938.63 to 57593.80)	45944.20 (33256.26 to 59291.35)	84.99 (17.45 to 336.26)	125.07 (35.74 to 398.47)	14535.24 (9550.70 to 19873.55)	14914.60 (9806.34 to 20331.01)
**35 to 39**	42649.58 (28859.39 to 56684.26)	44480.88 (31101.18 to 58457.42)	95.62 (21.43 to 349.55)	145.06 (41.87 to 427.57)	13359.36 (8463.05 to 18894.87)	13744.58 (8603.94 to 19625.20)
**40 to 44**	40179.75 (29077.46 to 54856.75)	41906.83 (30511.73 to 56738.89)	100.84 (28.27 to 336.19)	154.50 (52.92 to 408.25)	12657.99 (7671.02 to 17879.88)	13000.69 (8101.57 to 18499.33)
**45 to 49**	37364.21 (26256.10 to 49145.06)	39395.08 (28243.94 to 51910.58)	101.42 (27.12 to 306.95)	157.45 (50.58 to 394.68)	11527.16 (7476.32 to 16461.54)	12061.56 (7828.14 to 17177.06)
**50 to 54**	35930.52 (24942.56 to 48003.21)	38366.15 (26833.58 to 50812.84)	98.21 (26.55 to 299.80)	155.26 (49.32 to 383.08)	11818.62 (7556.66 to 16635.51)	12345.65 (7917.67 to 17246.94)
**55 to 59**	35869.42 (25549.77 to 49222.51)	37631.01 (26931.88 to 50932.02)	97.11 (25.61 to 304.33)	149.99 (46.19 to 378.63)	10699.71 (7003.94 to 15481.16)	10967.15 (7130.76 to 15769.93)
**60 to 64**	31464.10 (21312.23 to 44659.27)	32926.04 (22563.69 to 46626.56)	96.12 (27.54 to 295.39)	146.92 (49.32 to 364.48)	9640.94 (6074.46 to 14540.56)	10135.92 (6339.76 to 15364.77)
**65 to 69**	30875.75 (21672.83 to 43737.33)	33779.84 (24237.83 to 46657.16)	85.79 (24.36 to 275.02)	128.11 (42.96 to 338.17)	10466.01 (6571.84 to 15589.27)	11748.81 (7421.19 to 17320.16)
**70 to 74**	30105.05 (21098.06 to 42705.91)	34202.19 (24585.61 to 47543.62)	68.25 (19.01 to 247.73)	99.76 (31.64 to 306.52)	9439.27 (6157.73 to 13201.58)	10642.92 (7089.32 to 14876.16)
**75 to 79**	26851.86 (16611.97 to 37640.74)	30138.28 (19158.83 to 41581.00)	54.55 (13.76 to 203.96)	77.99 (23.23 to 252.34)	8537.35 (5510.52 to 12152.55)	9401.74 (6079.83 to 13306.97)
**80 to 84**	26495.16 (17732.17 to 37966.23)	29624.62 (19961.35 to 41686.73)	50.11 (11.71 to 191.80)	71.30 (20.59 to 240.33)	8664.42 (5596.16 to 12062.50)	9650.57 (6239.09 to 13481.25)

**85 to 89**	24513.98 (15776.28 to 35785.45)	27560.21 (18086.33 to 39177.17)	45.69 (9.59 to 177.75)	64.19 (17.07 to 210.15)	7457.51 (4522.15 to 11043.17)	8295.42 (5021.21 to 12199.25)
**90 to 94**	23832.15 (15412.75 to 34280.10)	25763.84 (16840.71 to 36924.81)	38.64 (8.16 to 150.78)	51.03 (13.23 to 176.53)	9072.77 (5759.81 to 13021.22)	9478.03 (5961.77 to 13543.95)
**95 plus**	33037.47 (21222.06 to 46428.71)	34045.53 (22050.72 to 48131.45)	36.80 (3.43 to 185.61)	42.90 (5.09 to 198.48)	12731.90 (6885.51 to 19273.80)	12865.76 (6985.87 to 19632.24)

Table S5a: Prevalence, DALY, incidence, and YLD rates of headache disorders for both sexes, by age, 2021

	Prevalence (95% UI)	DALYs (95% UI)	Incidence (95% UI)	YLDs (95% UI)
**<5 years**	0.00<br>(0.00 to 0.00)	0.00<br>(0.00 to 0.00)	0.00<br>(0.00 to 0.00)	0.00<br>(0.00 to 0.00)
**5 to 9**	10299.81<br>(7276.70 to 14222.85)	101.43<br>(4.07 to 269.06)	8534.25<br>(5958.83 to 11919.82)	101.43<br>(4.07 to 269.06)
**10 to 14**	37721.11<br>(30163.92 to 45907.65)	511.55<br>(36.09 to 1235.14)	15224.54<br>(10508.11 to 20436.54)	511.55<br>(36.09 to 1235.14)
**15 to 19**	49006.83<br>(38657.12 to 59353.26)	797.56<br>(102.13 to 1841.17)	15102.59<br>(10395.02 to 20918.31)	797.56<br>(102.13 to 1841.17)
**20 to 24**	52659.68<br>(43791.25 to 63509.04)	925.01<br>(137.46 to 2087.72)	15120.96<br>(10083.47 to 21228.25)	925.01<br>(137.46 to 2087.72)
**25 to 29**	55403.49<br>(46297.47 to 66497.29)	961.98<br>(149.36 to 2144.34)	16412.39<br>(11392.69 to 22792.85)	961.98<br>(149.36 to 2144.34)
**30 to 34**	58159.15<br>(48361.23 to 69430.09)	1003.30<br>(205.46 to 2115.97)	16146.91<br>(10946.99 to 21595.29)	1003.30<br>(205.46 to 2115.97)
**35 to 39**	57124.70<br>(46620.70 to 68023.89)	1061.71<br>(287.20 to 2158.98)	15108.74<br>(10175.62 to 20810.45)	1061.71<br>(287.20 to 2158.98)
**40 to 44**	55484.67<br>(46007.03 to 67188.83)	1106.45<br>(296.23 to 2312.11)	14144.10<br>(9294.53 to 19544.38)	1106.45<br>(296.23 to 2312.11)
**45 to 49**	52380.80<br>(43761.96 to 62587.50)	1051.63<br>(302.08 to 2101.16)	12690.42<br>(8339.31 to 17674.75)	1051.63<br>(302.08 to 2101.16)
**50 to 54**	50064.23<br>(40166.18 to 60898.73)	967.55<br>(304.46 to 1880.11)	12850.84<br>(8596.30 to 17718.59)	967.55<br>(304.46 to 1880.11)
**55 to 59**	48263.78<br>(38687.98 to 59879.86)	870.02<br>(310.29 to 1694.91)	11405.39<br>(7650.63 to 16196.15)	870.02<br>(310.29 to 1694.91)
**60 to 64**	42865.10<br>(33924.66 to 53888.37)	784.33<br>(305.94 to 1441.63)	10259.21<br>(6572.30 to 15301.54)	784.33<br>(305.94 to 1441.63)
**65 to 69**	41059.56<br>(32565.37 to 52172.47)	643.77<br>(277.83 to 1194.03)	11422.37<br>(7274.27 to 16791.67)	643.77<br>(277.83 to 1194.03)
**70 to 74**	39243.94<br>(30780.76 to 51236.88)	491.56<br>(185.55 to 950.04)	10360.26<br>(6906.33 to 14393.76)	491.56<br>(185.55 to 950.04)
**75 to 79**	34679.12<br>(24523.19 to 45341.70)	387.99<br>(132.51 to 783.12)	9291.27<br>(6085.58 to 13085.67)	387.99<br>(132.51 to 783.12)
**80 to 84**	33782.21<br>(25135.69 to 44815.91)	346.59<br>(125.63 to 686.15)	9535.22<br>(6209.02 to 13262.82)	346.59<br>(125.63 to 686.15)
**85 to 89**	31385.21<br>(22642.69 to 42338.10)	307.83<br>(120.40 to 591.30)	8203.35<br>(5060.90 to 12017.93)	307.83<br>(120.40 to 591.30)
**90 to 94**	29359.43<br>(21084.09 to 40037.27)	247.44<br>(95.63 to 488.55)	9472.48<br>(6059.32 to 13553.49)	247.44<br>(95.63 to 488.55)
**95 plus**	36604.35<br>(25303.66 to 49755.85)	176.31<br>(42.77 to 408.46)	12862.81<br>(6979.37 to 19587.83)	176.31<br>(42.77 to 408.46)

Table S5b: Prevalence, DALY, incidence, and YLD rates of migraine for both sexes, by age, 2021

	Prevalence (95% UI)	DALYs (95% UI)	Incidence (95% UI)	YLDs (95% UI)
**<5 years**	0.00 (0.00 to 0.00)	0.00 (0.00 to 0.00)	0.00 (0.00 to 0.00)	0.00 (0.00 to 0.00)
**5 to 9**	2728.93 (1763.04 to 3855.41)	92.01 (2.26 to 260.02)	1729.98 (1107.72 to 2481.44)	92.01 (2.26 to 260.02)
**10 to 14**	13029.90 (9536.89 to 17443.16)	472.42 (23.40 to 1172.38)	2616.65 (1904.94 to 3418.63)	472.42 (23.40 to 1172.38)
**15 to 19**	19570.89 (14500.84 to 24887.41)	729.42 (71.30 to 1741.01)	1913.79 (1113.39 to 2928.92)	729.42 (71.30 to 1741.01)
**20 to 24**	22658.13 (17300.32 to 28727.17)	842.38 (97.00 to 2002.91)	1706.37 (1028.41 to 2523.98)	842.38 (97.00 to 2002.91)
**25 to 29**	23749.21 (19014.37 to 29644.62)	873.71 (96.40 to 2032.06)	1535.46 (923.60 to 2301.97)	873.71 (96.40 to 2032.06)
**30 to 34**	23944.44 (18968.14 to 30227.80)	898.50 (130.93 to 1985.61)	1424.15 (872.92 to 2199.24)	898.50 (130.93 to 1985.61)
**35 to 39**	24358.05 (19581.11 to 30093.92)	941.44 (187.95 to 2014.21)	1557.33 (963.09 to 2396.05)	941.44 (187.95 to 2014.21)
**40 to 44**	24848.40 (20063.88 to 30993.52)	978.68 (213.63 to 2128.45)	1314.10 (832.13 to 1930.85)	978.68 (213.63 to 2128.45)
**45 to 49**	23022.31 (18370.83 to 28957.84)	921.90 (216.19 to 1898.42)	893.17 (516.69 to 1354.50)	921.90 (216.19 to 1898.42)
**50 to 54**	20824.86 (16923.04 to 26353.69)	840.34 (214.29 to 1735.76)	764.27 (436.73 to 1172.13)	840.34 (214.29 to 1735.76)
**55 to 59**	18344.72 (14728.72 to 23232.12)	745.53 (208.99 to 1531.24)	567.24 (329.40 to 881.29)	745.53 (208.99 to 1531.24)
**60 to 64**	15756.14 (12711.01 to 19840.82)	661.20 (218.05 to 1312.46)	355.16 (206.35 to 556.33)	661.20 (218.05 to 1312.46)
**65 to 69**	12816.89 (10050.00 to 16221.27)	534.78 (172.17 to 1093.64)	253.01 (139.32 to 376.58)	534.78 (172.17 to 1093.64)
**70 to 74**	10194.49 (7989.24 to 13114.07)	405.54 (129.23 to 829.89)	242.22 (145.94 to 372.22)	405.54 (129.23 to 829.89)
**75 to 79**	8370.40 (6469.31 to 10832.46)	319.85 (93.66 to 666.41)	252.93 (159.55 to 387.53)	319.85 (93.66 to 666.41)
**80 to 84**	7508.76 (5884.05 to 9797.84)	283.30 (91.22 to 591.11)	257.50 (159.27 to 395.50)	283.30 (91.22 to 591.11)
**85 to 89**	6682.18 (5108.49 to 8600.61)	250.09 (88.35 to 495.51)	200.01 (122.24 to 301.46)	250.09 (88.35 to 495.51)
**90 to 94**	5593.08 (4336.62 to 7249.46)	200.11 (60.72 to 410.30)	115.48 (76.90 to 165.65)	200.11 (60.72 to 410.30)
**95 plus**	4232.77 (3058.66 to 5784.91)	134.87 (27.92 to 305.27)	29.14 (19.33 to 41.95)	134.87 (27.92 to 305.27)

Table S5c: Prevalence, DALY, incidence, and YLD rates of tension-type headache for both sexes, by age, 2021

	Prevalence (95% UI)	DALYs (95% UI)	Incidence (95% UI)	YLDs (95% UI)
**<5 years**	0.00<br>(0.00 to 0.00)	0.00<br>(0.00 to 0.00)	0.00<br>(0.00 to 0.00)	0.00<br>(0.00 to 0.00)
**5 to 9**	7780.07<br>(4650.73 to 11692.02)	9.42<br>(0.49 to 55.44)	6804.27<br>(4200.24 to 10360.93)	9.42<br>(0.49 to 55.44)
**10 to 14**	28364.75<br>(20380.24 to 37382.03)	39.13<br>(4.60 to 206.69)	12607.89<br>(8036.40 to 17722.33)	39.13<br>(4.60 to 206.69)
**15 to 19**	36691.38<br>(23506.26 to 49165.32)	68.13<br>(13.06 to 293.09)	13188.80<br>(8389.63 to 18696.38)	68.13<br>(13.06 to 293.09)
**20 to 24**	39009.55<br>(28278.09 to 53087.25)	82.63<br>(19.04 to 323.17)	13414.59<br>(8357.99 to 19264.19)	82.63<br>(19.04 to 323.17)
**25 to 29**	41729.15<br>(29971.92 to 55585.09)	88.27<br>(19.43 to 358.69)	14876.93<br>(9794.29 to 20826.21)	88.27<br>(19.43 to 358.69)
**30 to 34**	45163.23<br>(32592.08 to 58559.68)	104.80<br>(26.01 to 362.91)	14722.76<br>(9691.89 to 20100.99)	104.80<br>(26.01 to 362.91)
**35 to 39**	43562.55<br>(29993.50 to 57648.57)	120.27<br>(31.54 to 381.54)	13551.41<br>(8554.68 to 19211.14)	120.27<br>(31.54 to 381.54)
**40 to 44**	41046.61<br>(29847.30 to 55835.92)	127.77<br>(41.48 to 374.75)	12830.00<br>(7901.80 to 18260.63)	127.77<br>(41.48 to 374.75)
**45 to 49**	38390.64<br>(27294.50 to 50597.98)	129.74<br>(39.01 to 356.03)	11797.25<br>(7636.07 to 16737.84)	129.74<br>(39.01 to 356.03)
**50 to 54**	37168.82<br>(25848.64 to 49443.73)	127.21<br>(37.96 to 341.85)	12086.57<br>(7761.57 to 16914.47)	127.21<br>(37.96 to 341.85)
**55 to 59**	36781.33<br>(26274.97 to 49850.02)	124.49<br>(35.95 to 343.61)	10838.15<br>(7075.41 to 15644.18)	124.49<br>(35.95 to 343.61)
**60 to 64**	32241.18<br>(22131.72 to 45649.02)	123.12<br>(39.35 to 328.33)	9904.04<br>(6225.50 to 14919.77)	123.12<br>(39.35 to 328.33)
**65 to 69**	32468.04<br>(23069.77 to 45337.71)	108.99<br>(34.95 to 310.86)	11169.36<br>(7053.42 to 16490.12)	108.99<br>(34.95 to 310.86)
**70 to 74**	32415.52<br>(23058.52 to 45539.25)	86.02<br>(26.38 to 282.56)	10118.04<br>(6631.78 to 14136.75)	86.02<br>(26.38 to 282.56)
**75 to 79**	28756.65<br>(17987.60 to 39810.92)	68.14<br>(19.34 to 230.74)	9038.35<br>(5834.63 to 12796.69)	68.14<br>(19.34 to 230.74)
**80 to 84**	28441.39<br>(19181.22 to 40169.28)	63.29<br>(17.06 to 221.51)	9277.71<br>(5984.05 to 12963.86)	63.29<br>(17.06 to 221.51)
**85 to 89**	26498.36<br>(17371.25 to 37898.79)	57.74<br>(14.61 to 199.95)	8003.34<br>(4839.39 to 11798.63)	57.74<br>(14.61 to 199.95)
**90 to 94**	25186.96<br>(16417.75 to 35984.38)	47.33<br>(11.71 to 167.25)	9357.00<br>(5916.65 to 13445.97)	47.33<br>(11.71 to 167.25)
**95 plus**	33803.93<br>(21823.63 to 47781.27)	41.44<br>(4.72 to 192.04)	12833.68<br>(6946.96 to 19556.29)	41.44<br>(4.72 to 192.04)

Table S6a: Socio-demographic index (SDI) with prevalence, DALY, and incidence counts and age-standardized rates of headache disorders in 2021, and percentage changes from 1990 to 2021, by country

	Prevalence (95% UI)			DALYs (95% UI)			Incidence (95% UI)			
	Absolute number, 2021	Age-standardized rate, per 100 000 people, 2021	Percentage Change, 1990-2021	Absolute number, 2021	Age-standardized rate, per 100 000 people, 2021	Percentage Change, 1990-2021	Absolute number, 2021	Age-standardized rate, per 100 000 people, 2021	Percentage Change, 1990-2021	SDI
Albania	1111776.51 (1007725.71 to 1213009.19)	38476.42 (35215.99 to 42165.44)	0.00% (0.00% to 0.00%)	17324.03 (5088.78 to 35849.56)	589.37 (160.90 to 1235.42)	0.01% (-0.02% to 0.04%)	320478.01 (280353.07 to 358471.05)	11652.16 (10199.48 to 12977.90)	0.00% (0.00% to 0.00%)	0.707
Andorra	40911.79 (37313.09 to 44497.92)	42629.38 (39124.84 to 46322.45)	0.00% (0.00% to 0.01%)	684.12 (153.33 to 1435.71)	715.80 (134.58 to 1535.08)	0.01% (-0.02% to 0.04%)	11015.85 (9641.41 to 12349.81)	12350.39 (10776.00 to 13772.84)	0.00% (0.00% to 0.01%)	0.869
Austria	4016921.58 (3666215.10 to 4354768.63)	41651.10 (38182.12 to 45318.70)	0.00% (0.00% to 0.00%)	65164.23 (15869.01 to 134359.10)	686.95 (142.79 to 1453.30)	-0.01% (-0.04% to 0.03%)	1115406.93 (991102.41 to 1241004.89)	12249.61 (10746.42 to 13722.59)	0.00% (-0.01% to 0.00%)	0.854
Belarus	3874077.90 (3541232.58 to 4237754.83)	38495.04 (34961.22 to 42186.13)	0.00% (0.00% to 0.00%)	65036.94 (24155.20 to 126096.41)	614.94 (207.62 to 1230.78)	-0.01% (-0.03% to 0.02%)	1105654.19 (973547.29 to 1236978.18)	11622.85 (10191.79 to 13031.11)	0.00% (0.00% to 0.00%)	0.784
Belgium	5480159.61 (5049086.86 to 5925564.99)	45248.17 (41636.71 to 48749.14)	0.02% (0.00% to 0.04%)	103009.65 (18387.96 to 212966.24)	869.78 (135.03 to 1852.24)	0.06% (-0.03% to 0.14%)	1433271.64 (1282396.32 to 1589930.19)	12505.75 (10938.66 to 14009.21)	0.00% (0.00% to 0.01%)	0.854
Bosnia and Herzegovina	1387960.88 (1258671.53 to 1515572.43)	38524.65 (35242.06 to 42228.28)	0.00% (0.00% to 0.00%)	21682.51 (6568.01 to 44421.38)	587.96 (158.83 to 1242.84)	0.00% (-0.04% to 0.03%)	394684.53 (347513.14 to 437221.32)	11664.79 (10215.59 to 12982.50)	0.00% (0.00% to 0.00%)	0.723
Bulgaria	2840072.06 (2577614.10 to 3101871.06)	38499.72 (35228.89 to 42208.79)	0.00% (0.00% to 0.00%)	44123.92 (13609.75 to 89700.92)	587.23 (158.48 to 1230.04)	-0.01% (-0.04% to 0.02%)	803704.42 (706997.19 to 889925.34)	11662.08 (10212.16 to 12990.41)	0.00% (0.00% to 0.00%)	0.768
Croatia	1762780.35 (1609806.27 to 1915623.37)	38493.85 (35225.66 to 42260.45)	0.00% (-0.03% to 0.02%)	27401.29 (8495.51 to 55807.65)	589.57 (159.87 to 1241.63)	0.00% (-0.05% to 0.04%)	501262.71 (445516.66 to 556989.26)	11681.90 (10317.21 to 13028.42)	0.00% (-0.03% to 0.03%)	0.798
Cyprus	636602.10 (581234.28 to 692744.27)	42809.49 (39325.69 to 46508.94)	0.00% (0.00% to 0.00%)	10842.92 (2321.93 to 22608.43)	727.41 (137.72 to 1541.74)	0.00% (-0.03% to 0.04%)	173891.14 (151934.94 to 195682.45)	12370.83 (10786.01 to 13829.15)	0.00% (0.00% to 0.00%)	0.836

Czechia	4396217.24 (3991280.92 to 4789038.99)	38480.43 (35213.99 to 42132.30)	0.00% (-0.01% to 0.00%)	68151.08 (20846.91 to 140407.18)	587.19 (160.02 to 1241.70)	-0.01% (-0.03% to 0.02%)	1252351.51 (1106773.62 to 1383941.42)	11660.29 (10196.90 to 12991.89)	0.00% (0.00% to 0.00%)	0.828
Denmark	2633655.70 (2400503.68 to 2855589.05)	42540.25 (38862.44 to 46345.61)	-0.01% (-0.04% to 0.02%)	39199.57 (9007.06 to 82289.48)	651.55 (132.65 to 1386.15)	0.02% (-0.04% to 0.08%)	724522.20 (638718.69 to 807712.18)	12319.07 (10742.49 to 13807.05)	0.00% (-0.03% to 0.04%)	0.896
Estonia	538297.74 (492720.75 to 587196.07)	38419.23 (34900.22 to 42118.05)	0.00% (-0.01% to 0.00%)	8826.85 (3326.14 to 17185.89)	610.87 (207.24 to 1226.13)	-0.01% (-0.04% to 0.02%)	153861.00 (136813.21 to 171409.24)	11617.62 (10176.07 to 13006.44)	0.00% (0.00% to 0.00%)	0.845
Finland	2488242.24 (2271971.76 to 2702230.63)	42710.96 (39220.53 to 46431.90)	0.00% (0.00% to 0.00%)	40348.54 (8961.08 to 83700.94)	719.17 (135.45 to 1529.10)	-0.01% (-0.04% to 0.02%)	684677.35 (610842.21 to 759611.08)	12362.70 (10771.76 to 13786.81)	0.00% (0.00% to 0.00%)	0.860
France	29010154.31 (26651455.51 to 31426239.00)	41999.81 (38565.09 to 45577.12)	0.00% (-0.01% to 0.01%)	497223.76 (125805.05 to 1010566.32)	736.23 (162.86 to 1534.45)	-0.01% (-0.06% to 0.05%)	8109907.87 (7207921.81 to 8963033.64)	12253.78 (10759.92 to 13769.39)	0.00% (-0.01% to 0.00%)	0.838
Germany	40161640.04 (37151603.80 to 43400814.82)	44235.70 (40699.99 to 47770.99)	0.02% (-0.01% to 0.04%)	687354.21 (153335.90 to 1417108.01)	779.61 (144.64 to 1653.36)	0.01% (-0.05% to 0.07%)	10553456.66 (9352219.81 to 11817058.96)	12434.30 (10911.24 to 13926.21)	0.00% (-0.03% to 0.03%)	0.903
Greece	4679151.06 (4290076.59 to 5069667.12)	43194.93 (39684.70 to 46928.01)	0.00% (-0.01% to 0.01%)	78258.00 (16938.00 to 162791.45)	744.45 (133.84 to 1599.87)	-0.01% (-0.05% to 0.04%)	1260563.91 (1118939.75 to 1400818.38)	12402.96 (10852.34 to 13852.73)	0.00% (-0.01% to 0.00%)	0.792
Hungary	4025505.44 (3658080.84 to 4386843.28)	38557.99 (35300.01 to 42278.68)	0.00% (0.00% to 0.00%)	62928.05 (19452.36 to 128889.81)	591.05 (160.17 to 1242.28)	0.00% (-0.03% to 0.04%)	1141427.14 (1010855.04 to 1262524.79)	11669.91 (10227.19 to 12994.97)	0.00% (0.00% to 0.00%)	0.791
Iceland	157488.77 (143959.11 to 171266.58)	42675.73 (39162.20 to 46416.32)	0.00% (0.00% to 0.00%)	2621.80 (550.21 to 5498.98)	720.38 (136.27 to 1543.73)	0.00% (-0.03% to 0.03%)	43860.19 (38771.25 to 49020.54)	12357.34 (10770.86 to 13805.78)	0.00% (0.00% to 0.00%)	0.876
Ireland	2222729.68 (2034750.37 to 2419564.76)	42809.50 (39341.31 to 46529.01)	0.00% (0.00% to 0.00%)	37197.72 (7844.75 to 77324.11)	723.70 (135.77 to 1547.14)	0.00% (-0.03% to 0.03%)	619192.46 (549749.60 to 688617.94)	12374.49 (10797.48 to 13842.21)	0.00% (0.00% to 0.00%)	0.874
Israel	4071252.52 (3737278.24 to 4419946.04)	42776.43 (39289.20 to 46515.46)	0.00% (0.00% to 0.00%)	68443.81 (13115.27 to 145947.55)	726.71 (134.37 to 1561.47)	-0.01% (-0.04% to 0.02%)	1170928.40 (1026555.13 to 1306687.84)	12370.15 (10792.25 to 13796.17)	0.00% (0.00% to 0.00%)	0.809

Italy	28602742.38 (26621545.37 to 30483272.94)	44664.39 (41565.61 to 47645.63)	0.00% (-0.01% to 0.01%)	488745.07 (101535.37 to 1015636.58)	788.66 (136.79 to 1690.55)	0.01% (-0.03% to 0.04%)	8003646.68 (7125062.44 to 8832112.56)	13401.93 (11857.95 to 14855.31)	0.00% (-0.02% to 0.02%)	0.806
Latvia	767765.78 (702010.93 to 835785.64)	38469.64 (34919.44 to 42160.71)	0.00% (0.00% to 0.00%)	12713.06 (4842.33 to 24503.72)	613.97 (208.75 to 1223.66)	-0.01% (-0.04% to 0.02%)	218257.98 (193757.82 to 243170.42)	11621.25 (10181.76 to 12999.80)	0.00% (0.00% to 0.00%)	0.831
Lithuania	1147273.33 (1040503.57 to 1249506.09)	39204.94 (35687.04 to 43235.42)	0.00% (0.00% to 0.00%)	17203.17 (6174.47 to 33996.92)	568.25 (181.60 to 1159.12)	-0.01% (-0.04% to 0.02%)	322753.46 (285018.22 to 359165.60)	11740.46 (10177.45 to 13176.82)	0.00% (0.00% to 0.00%)	0.856
Luxembourg	297366.50 (271670.20 to 323621.43)	42321.23 (38657.06 to 46167.66)	0.00% (0.00% to 0.00%)	5042.79 (1259.66 to 10353.77)	716.84 (158.71 to 1504.34)	0.00% (-0.03% to 0.03%)	81704.35 (71729.03 to 91839.71)	12303.05 (10729.98 to 13812.22)	0.00% (0.00% to 0.00%)	0.884
Malta	199940.71 (182443.28 to 217443.59)	42687.11 (39180.77 to 46392.29)	0.00% (-0.01% to 0.00%)	3274.04 (728.92 to 6824.83)	719.60 (137.05 to 1536.08)	-0.01% (-0.04% to 0.02%)	54470.53 (48314.32 to 60396.72)	12357.67 (10783.33 to 13780.26)	0.00% (0.00% to 0.00%)	0.802
Monaco	16933.99 (15417.91 to 18425.67)	42818.98 (39327.13 to 46456.39)	0.00% (0.00% to 0.00%)	274.15 (63.57 to 570.20)	725.81 (137.03 to 1558.23)	-0.01% (-0.03% to 0.02%)	4583.19 (4063.75 to 5111.50)	12376.21 (10776.57 to 13822.46)	0.00% (0.00% to 0.00%)	0.908
Montenegro	255948.00 (233410.83 to 279151.74)	38520.16 (35235.84 to 42273.68)	0.00% (0.00% to 0.00%)	4003.76 (1178.76 to 8345.83)	589.31 (157.90 to 1246.55)	-0.01% (-0.03% to 0.03%)	73805.22 (64938.86 to 81882.58)	11661.89 (10200.64 to 12979.07)	0.00% (0.00% to 0.00%)	0.796
Netherlands	8131656.55 (7435484.94 to 8826297.48)	44512.20 (40494.96 to 48384.33)	0.01% (0.00% to 0.03%)	122912.00 (24445.30 to 261170.84)	696.70 (117.88 to 1511.79)	0.05% (-0.03% to 0.12%)	2149134.80 (1901819.17 to 2406246.44)	12465.75 (10787.29 to 14085.75)	0.00% (0.00% to 0.01%)	0.888
North Macedonia	926551.19 (840702.74 to 1011371.31)	38432.02 (35156.80 to 42138.56)	0.00% (0.00% to 0.00%)	14575.07 (4314.51 to 30049.00)	585.04 (157.02 to 1233.25)	-0.01% (-0.04% to 0.02%)	264698.56 (232576.54 to 295362.27)	11651.74 (10205.74 to 12978.36)	0.00% (0.00% to 0.00%)	0.751
Norway	2603309.44 (2417933.43 to 2778893.13)	45218.08 (42206.51 to 48434.97)	0.02% (0.00% to 0.04%)	40813.38 (8271.52 to 86951.30)	724.37 (128.04 to 1565.65)	0.07% (0.01% to 0.11%)	749201.18 (669057.21 to 834462.06)	13651.16 (12119.39 to 15249.30)	0.02% (-0.01% to 0.04%)	0.916
Poland	16785847.49 (15527329.82 to 18025893.44)	40194.33 (37217.06 to 43327.84)	0.00% (0.00% to 0.00%)	266046.27 (84278.07 to 546344.85)	621.37 (173.13 to 1320.28)	0.00% (-0.02% to 0.02%)	4908681.67 (4358461.87 to 5410021.27)	12497.09 (11012.62 to 13861.48)	0.00% (0.00% to 0.00%)	0.812
Portugal	4874791.29 (4460348.37 to 5288045.49)	42868.14 (39366.80 to 46559.82)	0.00% (0.00% to 0.00%)	80275.30 (18257.65 to 167123.25)	725.81 (135.60 to 1551.77)	0.00% (-0.03% to 0.03%)	1317023.77 (1171295.10	12381.99 (10816.95 to 13812.65)	0.00% (0.00% to 0.00%)	0.744

							to 1468448.09)			
Republic of Moldova	1533234.14 (1398128.93 to 1676699.52)	38461.40 (34929.08 to 42130.70)	0.00% (-0.01% to 0.00%)	25716.13 (9663.90 to 49963.76)	614.27 (210.48 to 1224.94)	-0.01% (-0.04% to 0.02%)	435504.09 (383513.81 to 489406.25)	11619.88 (10183.64 to 12996.22)	0.00% (0.00% to 0.00%)	0.732
Romania	7870577.20 (7138500.54 to 8577746.52)	38510.22 (35238.67 to 42208.33)	0.00% (0.00% to 0.00%)	123015.12 (37926.43 to 253248.30)	590.34 (159.82 to 1237.77)	0.00% (-0.03% to 0.03%)	2246893.14 (1985294.18 to 2478666.99)	11663.61 (10215.33 to 12986.62)	0.00% (0.00% to 0.00%)	0.768
Russian Federation	62332395.71 (57769445.66 to 67054482.23)	39965.73 (37115.45 to 43140.11)	0.00% (-0.01% to 0.02%)	1127144.82 (444266.70 to 2172711.79)	689.20 (252.96 to 1365.28)	0.00% (-0.01% to 0.01%)	18144042.49 (16020909.20 to 20054671.54)	12288.94 (10856.13 to 13655.23)	0.00% (-0.02% to 0.03%)	0.809
San Marino	15037.58 (13739.87 to 16332.56)	42886.77 (39405.77 to 46603.34)	0.00% (0.00% to 0.00%)	248.56 (56.23 to 523.85)	730.07 (136.36 to 1559.46)	0.00% (-0.03% to 0.03%)	4083.22 (3631.86 to 4560.59)	12380.14 (10805.04 to 13823.03)	0.00% (0.00% to 0.00%)	0.888
Serbia	3773827.15 (3427357.56 to 4116973.03)	38444.45 (35153.88 to 42117.43)	0.00% (-0.01% to 0.00%)	58613.04 (17828.78 to 120078.88)	587.77 (159.66 to 1226.17)	-0.01% (-0.04% to 0.02%)	1080853.79 (951961.43 to 1196947.71)	11646.64 (10204.49 to 12978.10)	0.00% (-0.01% to 0.00%)	0.792
Slovakia	2278919.51 (2064371.22 to 2487379.12)	38519.78 (35252.66 to 42253.64)	0.00% (-0.01% to 0.00%)	35845.52 (10936.92 to 74181.14)	590.07 (159.68 to 1245.05)	0.00% (-0.04% to 0.03%)	649163.93 (570678.42 to 720699.03)	11665.16 (10222.89 to 12980.30)	0.00% (0.00% to 0.00%)	0.811
Slovenia	857040.02 (778360.36 to 935158.22)	38434.96 (35169.02 to 42139.83)	0.00% (-0.01% to 0.00%)	13302.83 (4157.55 to 27205.83)	586.76 (158.64 to 1234.60)	-0.01% (-0.04% to 0.02%)	243660.99 (215054.78 to 269619.72)	11652.82 (10201.69 to 12990.49)	0.00% (-0.01% to 0.00%)	0.842
Spain	20805827.73 (19057607.99 to 22647098.48)	42562.89 (38998.75 to 46280.46)	0.00% (0.00% to 0.00%)	367181.53 (88646.61 to 743247.46)	761.92 (154.41 to 1604.50)	0.00% (-0.04% to 0.05%)	5649306.43 (5007437.48 to 6285855.36)	12290.65 (10804.21 to 13682.43)	0.00% (0.00% to 0.00%)	0.769
Sweden	4809269.99 (4460518.39 to 5114657.51)	44268.06 (41066.79 to 47368.75)	0.00% (-0.02% to 0.02%)	76445.12 (15602.64 to 163791.11)	726.13 (130.67 to 1552.67)	0.00% (-0.05% to 0.04%)	1384691.00 (1239973.97 to 1522185.67)	13366.95 (11894.52 to 14829.75)	0.00% (-0.02% to 0.03%)	0.887
Switzerland	3779862.07 (3455348.41 to 4118181.32)	39650.85 (36201.32 to 43093.39)	0.03% (-0.01% to 0.06%)	61514.71 (14884.79 to 126998.11)	656.54 (136.41 to 1398.70)	0.01% (-0.04% to 0.06%)	1084867.81 (956762.98 to 1201250.14)	11998.61 (10607.68 to 13311.18)	0.01% (-0.02% to 0.05%)	0.933
Ukraine	19092240.80 (17688362.53	40101.64 (37085.00 to 43123.07)	0.00% (0.00% to 0.00%)	319949.51 (121242.85	639.78 (220.30 to 1285.48)	-0.01% (-0.03% to 0.02%)	5560687.57 (4937700.70	12451.11 (11002.97 to 13877.76)	0.00% (0.00% to 0.00%)	0.761

	to 20602403.26)			to 619277.46)			to 6181265.61)			
United Kingdom	31251134.36 (29010848.37 to 33456243.13)	43534.09 (40481.89 to 46591.20)	0.00% (-0.01% to 0.01%)	507234.95 (114326.49 to 1066623.73)	720.61 (143.14 to 1541.48)	0.00% (-0.03% to 0.04%)	9045126.21 (8046636.68 to 10035284.86)	13199.69 (11724.90 to 14665.17)	0.00% (0.00% to 0.00%)	0.859
**Statistical Test for SDI**	Spearman's ρ = -0.131 p-value = 0.390	Spearman's ρ = 0.510 p-value < 0.001		Spearman's ρ = -0.140 p-value = 0.359	Spearman's ρ = 0.389 p-value = 0.008		Spearman's ρ = -0131. p-value = 0.392	Spearman's ρ = 0.455 p-value = 0.002		

Table S6b: Socio-demographic index (SDI) with prevalence, DALY, and incidence counts and age-standardized rates of migraine in 2021, and percentage changes from 1990 to 2021, by country

	Prevalence (95% UI)			DALYs (95% UI)			Incidence (95% UI)			
	Absolute number, 2021	Age-standardized rate, per 100 000 people, 2021	Percentage Change, 1990-2021	Absolute number, 2021	Age-standardized rate, per 100 000 people, 2021	Percentage Change, 1990-2021	Absolute number, 2021	Age-standardized rate, per 100 000 people, 2021	Percentage Change, 1990-2021	SDI
Albania	392413.10 (337140.67 to 452887.21)	13598.05 (11517.73 to 15739.28)	0.01% (0.00% to 0.01%)	15072.29 (3582.10 to 32681.51)	516.38 (111.31 to 1154.53)	0.01% (-0.02% to 0.04%)	26298.77 (22549.23 to 30543.77)	1084.70 (921.54 to 1253.10)	0.00% (0.00% to 0.00%)	0.707
Andorra	16182.80 (13861.95 to 18948.69)	17391.72 (14783.07 to 20499.22)	0.01% (0.01% to 0.01%)	610.96 (105.11 to 1323.79)	646.76 (91.47 to 1428.97)	0.01% (-0.02% to 0.05%)	954.77 (814.50 to 1109.18)	1364.67 (1158.41 to 1572.01)	0.01% (0.01% to 0.01%)	0.869
Austria	1522682.97 (1315123.42 to 1761091.77)	16466.12 (14043.90 to 19214.85)	-0.01% (-0.01% to 0.00%)	57720.25 (10769.14 to 124519.76)	615.73 (95.30 to 1358.32)	-0.01% (-0.04% to 0.03%)	95368.15 (81876.92 to 109644.93)	1314.38 (1125.54 to 1514.20)	0.00% (0.00% to 0.00%)	0.854
Belarus	1331465.04 (1154466.27 to 1524260.49)	13015.65 (11182.02 to 15082.62)	-0.01% (-0.01% to -0.01%)	55176.12 (17071.72 to 112397.35)	526.93 (143.58 to 1111.25)	-0.01% (-0.04% to 0.02%)	84229.35 (71470.98 to 97496.84)	1009.66 (855.42 to 1163.87)	0.00% (-0.01% to 0.00%)	0.784
Belgium	2544608.37 (2192801.83 to 2985664.82)	21751.47 (18730.05 to 25705.50)	0.08% (0.01% to 0.16%)	94014.86 (12767.28 to 205439.93)	800.36 (91.99 to 1772.02)	0.07% (-0.04% to 0.15%)	139418.54 (119851.61 to 158091.00)	1506.95 (1288.44 to 1714.30)	0.02% (-0.02% to 0.07%)	0.854
Bosnia and Herzegovina	491055.62 (425225.04 to 566028.59)	13632.57 (11539.12 to 15778.58)	0.00% (0.00% to 0.00%)	18815.34 (4515.30 to 40504.83)	515.27 (107.38 to 1145.56)	0.00% (-0.04% to 0.03%)	31326.47 (26867.56 to 36256.93)	1089.27 (925.71 to 1259.42)	0.00% (0.00% to 0.00%)	0.723
Bulgaria	994945.37 (861408.71 to 1144457.20)	13595.28 (11506.72 to 15736.87)	-0.01% (-0.01% to 0.00%)	38217.27 (9300.70 to 82359.53)	514.42 (106.41 to 1139.61)	-0.01% (-0.04% to 0.03%)	62165.15 (53191.04 to 72187.69)	1086.68 (923.30 to 1256.69)	-0.01% (-0.01% to 0.00%)	0.768
Croatia	615855.20 (536629.95 to 712210.72)	13584.21 (11643.92 to 15873.25)	-0.01% (-0.05% to 0.03%)	23744.19 (5717.80 to 50875.52)	516.50 (107.38 to 1142.33)	-0.01% (-0.05% to 0.04%)	38937.31 (33671.78 to 45359.32)	1087.90 (919.35 to 1261.92)	0.00% (-0.03% to 0.03%)	0.798
Cyprus	258627.60 (222725.23 to 302839.91)	17622.86 (14998.63 to 20771.18)	0.01% (0.00% to 0.01%)	9731.92 (1569.49 to 21129.20)	657.50 (94.31 to 1443.10)	0.00% (-0.03% to 0.04%)	16330.62 (13796.29 to 19224.21)	1370.05 (1163.83 to 1578.47)	0.00% (0.00% to 0.00%)	0.836

Czechia	1539807.14 (1332072.98 to 1769906.12)	13581.76 (11491.97 to 15721.88)	-0.01% (-0.01% to -0.01%)	59096.60 (14003.58 to 127917.20)	514.44 (105.14 to 1155.09)	-0.01% (-0.04% to 0.03%)	98768.46 (84436.15 to 114611.47)	1087.51 (924.05 to 1257.72)	0.00% (0.00% to 0.00%)	0.828
Denmark	906327.27 (783204.77 to 1046930.30)	15422.39 (13002.12 to 17919.21)	0.03% (-0.02% to 0.08%)	34566.93 (6198.30 to 72993.07)	581.19 (88.91 to 1273.05)	0.03% (-0.03% to 0.09%)	61195.13 (52310.56 to 70307.66)	1266.41 (1076.02 to 1458.59)	0.01% (-0.03% to 0.06%)	0.896
Estonia	180463.75 (156480.80 to 206209.93)	12900.48 (11078.06 to 14961.95)	-0.01% (-0.02% to -0.01%)	7476.86 (2289.15 to 15185.74)	523.11 (142.17 to 1110.52)	-0.01% (-0.04% to 0.02%)	11361.97 (9694.91 to 13121.80)	1004.87 (851.11 to 1158.00)	-0.01% (-0.01% to -0.01%)	0.845
Finland	959037.81 (826906.73 to 1120219.04)	17477.69 (14864.62 to 20595.43)	-0.01% (-0.01% to 0.00%)	36012.12 (6289.88 to 77781.08)	649.91 (91.00 to 1426.19)	-0.01% (-0.04% to 0.02%)	60345.30 (51402.29 to 69798.89)	1366.62 (1160.44 to 1574.69)	0.00% (0.00% to 0.00%)	0.860
France	11508601.68 (9881348.61 to 13376867.86)	17478.90 (14980.50 to 20463.93)	-0.01% (-0.05% to 0.04%)	441400.79 (84487.74 to 934151.12)	661.31 (107.65 to 1429.12)	-0.01% (-0.06% to 0.05%)	746314.19 (639834.58 to 855267.22)	1360.59 (1153.37 to 1566.45)	-0.01% (-0.04% to 0.03%)	0.838
Germany	16539322.38 (14277979.53 to 19290791.57)	19203.72 (16546.88 to 22548.90)	0.02% (-0.02% to 0.08%)	615737.75 (104953.06 to 1316251.10)	707.40 (101.69 to 1567.80)	0.02% (-0.05% to 0.08%)	922854.61 (800530.58 to 1048308.86)	1421.37 (1215.13 to 1626.46)	0.01% (-0.03% to 0.05%)	0.903
Greece	1868500.63 (1599645.43 to 2180107.87)	18254.82 (15571.69 to 21589.54)	0.00% (-0.04% to 0.05%)	69985.94 (11835.20 to 151360.14)	674.80 (91.73 to 1475.63)	-0.01% (-0.06% to 0.04%)	109982.70 (94130.93 to 125923.97)	1401.01 (1190.61 to 1612.85)	0.00% (-0.04% to 0.03%)	0.792
Hungary	1420158.83 (1231619.28 to 1633084.72)	13659.57 (11564.90 to 15808.12)	-0.01% (-0.01% to 0.00%)	54587.84 (13037.44 to 117434.66)	517.97 (106.21 to 1147.24)	0.00% (-0.03% to 0.04%)	89188.31 (76539.25 to 103431.50)	1090.02 (926.44 to 1260.30)	0.00% (0.00% to 0.00%)	0.791
Iceland	62574.14 (53834.22 to 72796.51)	17439.61 (14827.64 to 20549.55)	0.00% (-0.01% to 0.00%)	2352.48 (381.60 to 5109.59)	651.01 (92.73 to 1435.23)	0.00% (-0.03% to 0.03%)	4209.15 (3578.03 to 4867.64)	1366.05 (1159.56 to 1575.06)	0.00% (0.00% to 0.00%)	0.876
Ireland	890679.36 (765137.90 to 1040070.43)	17632.30 (14997.65 to 20793.88)	0.00% (0.00% to 0.00%)	33360.28 (5525.86 to 72824.76)	654.12 (94.34 to 1444.88)	0.00% (-0.03% to 0.03%)	60723.56 (51513.06 to 70019.49)	1374.16 (1167.95 to 1582.77)	0.00% (0.00% to 0.00%)	0.874
Israel	1646071.46 (1408912.11 to 1929109.68)	17572.11 (14949.33 to 20712.90)	-0.01% (-0.01% to -0.01%)	61690.19 (9154.26 to 135395.15)	656.91 (92.78 to 1443.76)	-0.01% (-0.04% to 0.02%)	125322.31 (106284.50 to 144414.74)	1370.09 (1163.93 to 1578.32)	0.00% (0.00% to 0.00%)	0.809

Italy	11636929.73 (10174340.39 to 13422449.68)	19244.27 (16729.30 to 22008.96)	0.01% (-0.02% to 0.04%)	438568.65 (68925.67 to 944341.92)	717.20 (89.24 to 1568.08)	0.01% (-0.03% to 0.05%)	673245.79 (599378.97 to 754102.43)	1494.64 (1329.20 to 1675.31)	0.00% (-0.02% to 0.03%)	0.806
Latvia	259186.37 (225126.13 to 296312.02)	12978.48 (11148.32 to 15044.14)	-0.01% (-0.01% to -0.01%)	10761.04 (3321.95 to 21824.39)	526.11 (142.47 to 1108.17)	-0.01% (-0.04% to 0.02%)	15967.60 (13654.18 to 18399.10)	1008.08 (854.07 to 1161.98)	-0.01% (-0.01% to -0.01%)	0.831
Lithuania	358843.34 (310510.04 to 412286.05)	12244.96 (10458.50 to 14260.23)	-0.01% (-0.01% to -0.01%)	14584.74 (4246.62 to 29905.12)	487.22 (121.55 to 1024.43)	-0.01% (-0.04% to 0.02%)	22584.35 (19514.17 to 26192.30)	976.09 (830.75 to 1129.22)	-0.01% (-0.01% to -0.01%)	0.856
Luxembourg	116291.68 (99994.13 to 136017.10)	16915.46 (14437.25 to 19829.86)	0.00% (0.00% to 0.00%)	4472.92 (872.26 to 9474.61)	641.87 (107.72 to 1397.18)	0.00% (-0.03% to 0.03%)	7393.51 (6283.54 to 8584.21)	1333.63 (1135.03 to 1536.23)	0.00% (0.00% to 0.00%)	0.884
Malta	77439.46 (66742.88 to 90119.67)	17448.60 (14837.97 to 20561.45)	-0.01% (-0.01% to -0.01%)	2921.20 (517.52 to 6307.73)	650.36 (92.31 to 1421.91)	-0.01% (-0.04% to 0.02%)	4680.98 (3976.12 to 5478.41)	1363.74 (1158.17 to 1572.08)	0.00% (-0.01% to 0.00%)	0.802
Monaco	6438.22 (5553.35 to 7523.67)	17639.08 (15006.93 to 20802.69)	0.00% (0.00% to 0.00%)	243.53 (45.70 to 521.42)	655.88 (96.05 to 1443.87)	-0.01% (-0.04% to 0.02%)	372.71 (320.19 to 427.62)	1375.74 (1168.80 to 1584.54)	0.00% (0.00% to 0.01%)	0.908
Montenegro	90944.24 (78570.22 to 104953.56)	13632.03 (11541.95 to 15776.95)	0.00% (0.00% to 0.00%)	3483.26 (786.48 to 7549.62)	516.27 (105.41 to 1149.24)	-0.01% (-0.04% to 0.03%)	6143.81 (5248.62 to 7111.43)	1087.93 (924.59 to 1257.63)	0.00% (0.00% to 0.00%)	0.796
Netherlands	2944900.50 (2542076.55 to 3405005.83)	17015.11 (14543.35 to 19738.31)	0.06% (0.01% to 0.13%)	109685.32 (16353.25 to 242318.90)	628.54 (78.22 to 1410.88)	0.06% (-0.02% to 0.12%)	188015.68 (162112.92 to 215993.19)	1351.72 (1157.08 to 1557.38)	0.04% (0.00% to 0.08%)	0.888
North Macedonia	331180.87 (284824.85 to 383702.41)	13534.57 (11451.17 to 15668.78)	-0.01% (-0.01% to -0.01%)	12682.44 (2942.47 to 27528.89)	512.39 (106.60 to 1141.21)	-0.01% (-0.04% to 0.02%)	21804.36 (18578.37 to 25492.37)	1084.44 (921.19 to 1254.10)	-0.01% (-0.01% to 0.00%)	0.751
Norway	980506.95 (852993.92 to 1124376.32)	17700.09 (15229.47 to 20234.71)	0.08% (0.04% to 0.12%)	36469.37 (5565.68 to 79611.21)	653.12 (84.81 to 1449.64)	0.08% (0.02% to 0.12%)	65740.32 (58406.10 to 73787.60)	1425.49 (1259.66 to 1603.91)	0.07% (0.04% to 0.09%)	0.916
Poland	5973361.25 (5222975.69 to 6772298.13)	14258.61 (12376.50 to 16315.58)	0.00% (-0.01% to 0.00%)	230605.64 (56407.91 to 492545.37)	543.31 (117.09 to 1189.22)	0.00% (-0.01% to 0.01%)	382967.05 (339974.28 to 433272.49)	1131.81 (999.78 to 1277.76)	0.00% (0.00% to 0.00%)	0.812
Portugal	1903084.50 (1643638.13	17692.88 (15059.18 to 20857.53)	0.00% (0.00% to 0.00%)	71559.35 (12909.69 to 153420.92)	656.01 (93.82 to 1444.85)	0.00% (-0.03% to 0.03%)	112244.87 (95860.44 to 129639.81)	1375.15 (1168.59 to 1583.65)	0.00% (0.00% to 0.00%)	0.744

	to 2225481.89)									
Republic of Moldova	527387.15 (457303.00 to 607970.50)	12971.64 (11140.99 to 15039.45)	-0.01% (-0.01% to -0.01%)	21846.58 (6672.59 to 44583.80)	526.37 (144.95 to 1120.24)	-0.01% (-0.04% to 0.02%)	33026.19 (28000.85 to 38566.09)	1008.23 (854.09 to 1162.23)	-0.01% (-0.01% to -0.01%)	0.732
Romania	2766760.79 (2398063.67 to 3185736.85)	13609.33 (11518.75 to 15752.39)	-0.01% (-0.01% to 0.00%)	106718.69 (25310.75 to 228723.74)	517.26 (105.21 to 1151.94)	0.00% (-0.03% to 0.03%)	178064.25 (153122.22 to 206093.00)	1087.77 (924.29 to 1257.83)	0.00% (0.00% to 0.00%)	0.768
Russian Federation	22799067.61 (19758993.61 to 25864413.57)	14379.09 (12456.59 to 16421.26)	0.00% (0.00% to 0.00%)	955153.59 (306140.85 to 1915841.84)	589.48 (172.15 to 1220.02)	0.00% (-0.01% to 0.00%)	1433750.72 (1272611.88 to 1615349.92)	1088.30 (959.74 to 1222.56)	0.00% (0.00% to 0.00%)	0.809
San Marino	5877.84 (5077.87 to 6881.18)	17738.70 (15098.00 to 20908.45)	0.01% (0.01% to 0.01%)	221.72 (39.07 to 478.47)	660.00 (94.00 to 1449.18)	0.00% (-0.03% to 0.03%)	352.19 (299.97 to 404.35)	1374.79 (1169.05 to 1583.39)	0.00% (0.00% to 0.00%)	0.888
Serbia	1325082.46 (1147908.98 to 1527772.03)	13545.29 (11487.32 to 15681.86)	-0.01% (-0.01% to -0.01%)	50906.11 (12097.85 to 109628.21)	514.68 (107.95 to 1136.79)	-0.01% (-0.04% to 0.02%)	86814.76 (74217.59 to 100956.48)	1080.23 (917.37 to 1248.49)	-0.01% (-0.01% to -0.01%)	0.792
Slovakia	810276.29 (699862.57 to 935455.64)	13618.05 (11526.24 to 15761.99)	-0.01% (-0.01% to -0.01%)	31139.92 (7227.28 to 67224.49)	516.94 (105.43 to 1156.64)	0.00% (-0.04% to 0.03%)	52396.76 (44649.51 to 61229.36)	1088.42 (924.96 to 1258.68)	0.00% (-0.01% to 0.00%)	0.811
Slovenia	298680.72 (258727.88 to 343964.28)	13531.47 (11447.95 to 15665.17)	-0.01% (-0.01% to -0.01%)	11517.06 (2765.60 to 24685.68)	513.87 (104.38 to 1148.52)	-0.01% (-0.04% to 0.03%)	18844.30 (16115.96 to 21877.66)	1084.46 (921.24 to 1254.18)	-0.01% (-0.01% to 0.00%)	0.842
Spain	8537877.56 (7350314.09 to 10025982.00)	18266.01 (15746.95 to 21477.00)	0.00% (-0.01% to 0.00%)	327242.61 (62354.28 to 698496.34)	688.24 (105.50 to 1469.69)	0.00% (-0.04% to 0.05%)	502945.43 (431506.28 to 580753.48)	1393.83 (1188.05 to 1607.19)	0.00% (0.00% to 0.00%)	0.769
Sweden	1846704.34 (1613904.80 to 2112037.97)	17826.53 (15449.25 to 20442.69)	0.00% (-0.03% to 0.03%)	68595.04 (10307.23 to 147741.68)	657.82 (84.47 to 1464.24)	0.00% (-0.05% to 0.05%)	121035.51 (107677.79 to 135701.62)	1409.70 (1249.25 to 1585.26)	0.00% (-0.03% to 0.02%)	0.887
Switzerland	1444817.20 (1250524.02 to 1670106.75)	15818.27 (13505.05 to 18301.38)	0.01% (-0.03% to 0.05%)	54637.32 (10398.48 to 117888.09)	590.17 (93.02 to 1295.68)	0.01% (-0.05% to 0.06%)	93025.73 (79679.06 to 107400.93)	1284.81 (1090.41 to 1475.61)	0.00% (-0.03% to 0.04%)	0.933

Ukraine	6594152.42 (5746869.78 to 7566954.18)	13592.26 (11831.93 to 15502.08)	-0.01% (-0.01% to -0.01%)	271531.32 (83932.12 to 543384.43)	548.06 (152.25 to 1136.00)	-0.01% (-0.04% to 0.02%)	408130.27 (360557.29 to 458775.83)	1046.04 (921.40 to 1181.66)	-0.01% (-0.01% to -0.01%)	0.761
United Kingdom	12064770.46 (10517925.66 to 13963773.48)	17467.16 (15069.84 to 20164.22)	0.01% (-0.02% to 0.04%)	452047.07 (77045.83 to 968291.88)	648.34 (93.61 to 1413.04)	0.01% (-0.03% to 0.04%)	797470.13 (708496.71 to 898063.33)	1387.52 (1222.47 to 1566.53)	0.01% (-0.02% to 0.03%)	0.859
**Statistical Test for SDI**	Spearman's ρ = -0.127 p-value = 0.405	Spearman's ρ = 0.391 p-value = 0.008		Spearman's ρ = -0.131 p-value = 0.391	Spearman's ρ = 0.392 p-value = 0.008		Spearman's ρ = -0.110 p-value = 0.470	Spearman's ρ = 0.438 p-value = 0.003		

Table S6c: Socio-demographic index (SDI) with prevalence, DALY, and incidence counts and age-standardized rates of tension-type headache in 2021, and percentage changes from 1990 to 2021, by country

	Prevalence (95% UI)			DALYs (95% UI)			Incidence (95% UI)			
	Absolute number, 2021	Age-standardized rate, per 100 000 people, 2021	Percentage Change, 1990-2021	Absolute number, 2021	Age-standardized rate, per 100 000 people, 2021	Percentage Change, 1990-2021	Absolute number, 2021	Age-standardized rate, per 100 000 people, 2021	Percentage Change, 1990-2021	SDI
Albania	864333.17 (756269.08 to 980673.58)	29929.00 (25981.73 to 34255.02)	0.00% (0.00% to 0.00%)	2251.74 (715.54 to 7082.91)	72.99 (21.94 to 242.13)	0.01% (-0.08% to 0.10%)	294179.24 (254647.38 to 331984.33)	10567.46 (9117.97 to 11922.52)	0.00% (0.00% to 0.00%)	0.707
Andorra	31423.49 (27452.80 to 35599.97)	32352.91 (28179.03 to 36861.21)	0.00% (0.00% to 0.01%)	73.15 (19.82 to 260.48)	69.04 (17.06 to 256.96)	0.01% (-0.09% to 0.11%)	10061.08 (8712.60 to 11398.53)	10985.72 (9464.72 to 12425.65)	0.00% (0.00% to 0.00%)	0.869
Austria	3103357.82 (2715444.43 to 3487542.35)	31759.67 (27635.41 to 36479.78)	0.00% (0.00% to 0.00%)	7443.98 (2141.41 to 26586.70)	71.22 (19.01 to 267.60)	0.00% (-0.12% to 0.10%)	1020038.78 (895524.56 to 1147065.04)	10935.23 (9446.25 to 12425.36)	0.00% (-0.01% to 0.00%)	0.854
Belarus	3032617.46 (2670381.18 to 3446845.49)	30340.65 (26426.41 to 34728.35)	0.00% (0.00% to 0.00%)	9860.82 (3328.76 to 28254.23)	88.01 (27.48 to 269.39)	0.00% (-0.09% to 0.09%)	1021424.83 (892205.65 to 1153585.45)	10613.19 (9181.14 to 11995.05)	0.00% (0.00% to 0.00%)	0.784
Belgium	3969616.84 (3489453.36 to 4488578.92)	32396.92 (28239.73 to 36903.59)	0.00% (-0.01% to 0.00%)	8994.79 (2463.24 to 32639.70)	69.42 (17.77 to 261.14)	0.00% (-0.11% to 0.10%)	1293853.10 (1138357.60 to 1449035.28)	10998.81 (9469.81 to 12445.79)	0.00% (0.00% to 0.00%)	0.854
Bosnia and Herzegovina	1077398.60 (944877.29 to 1215194.37)	29958.94 (26050.58 to 34343.35)	0.00% (-0.01% to 0.00%)	2867.17 (931.51 to 8679.88)	72.69 (21.99 to 238.70)	0.00% (-0.11% to 0.09%)	363358.06 (316378.13 to 404837.98)	10575.51 (9138.10 to 11929.93)	0.00% (0.00% to 0.00%)	0.723
Bulgaria	2209582.32 (1929372.70 to 2478332.40)	29955.10 (26079.24 to 34340.31)	0.00% (0.00% to 0.00%)	5906.65 (1871.44 to 17850.31)	72.81 (21.03 to 240.78)	0.00% (-0.10% to 0.08%)	741539.27 (647710.63 to 828288.01)	10575.40 (9134.59 to 11930.69)	0.00% (0.00% to 0.00%)	0.768
Croatia	1372487.35 (1199050.64 to 1544565.33)	29953.92 (26171.06 to 34212.29)	0.00% (-0.04% to 0.04%)	3657.10 (1163.29 to 11350.30)	73.08 (21.40 to 245.11)	0.00% (-0.10% to 0.08%)	462325.40 (407661.12 to 516379.53)	10594.00 (9213.94 to 11944.99)	0.00% (-0.03% to 0.03%)	0.798
Cyprus	486411.42 (421730.38 to 553681.48)	32411.95 (28254.01 to 37014.80)	0.00% (0.00% to 0.01%)	1111.01 (302.23 to 3982.28)	69.91 (18.09 to 259.22)	0.01% (-0.12% to 0.10%)	157560.52 (136812.54 to 178603.21)	11000.77 (9453.23 to 12466.52)	0.00% (0.00% to 0.00%)	0.836

Czechia	3421199.19 (2998773.73 to 3833393.42)	29944.15 (26025.75 to 34247.32)	0.00% (-0.01% to 0.00%)	9054.47 (2849.09 to 27930.03)	72.75 (21.07 to 244.46)	0.00% (-0.09% to 0.08%)	1153583.05 (1010386.84 to 1286485.72)	10572.78 (9122.21 to 11924.83)	0.00% (0.00% to 0.00%)	0.828
Denmark	2116005.52 (1858506.23 to 2388872.96)	33764.78 (29477.82 to 38545.83)	-0.03% (-0.07% to 0.02%)	4632.65 (1204.37 to 17116.71)	70.36 (16.92 to 266.48)	-0.01% (-0.13% to 0.13%)	663327.07 (575287.31 to 741568.53)	11052.66 (9475.52 to 12514.31)	0.00% (-0.03% to 0.04%)	0.896
Estonia	423964.58 (373686.42 to 481706.88)	30335.97 (26372.25 to 34759.24)	0.00% (0.00% to 0.00%)	1350.00 (466.04 to 3954.34)	87.75 (28.49 to 271.39)	0.00% (-0.08% to 0.08%)	142499.03 (126073.35 to 159809.83)	10612.76 (9186.97 to 11995.59)	0.00% (0.00% to 0.00%)	0.845
Finland	1917833.38 (1686801.86 to 2164471.63)	32383.20 (28196.63 to 36941.96)	0.00% (-0.01% to 0.00%)	4336.41 (1198.56 to 15480.41)	69.26 (18.66 to 256.04)	0.00% (-0.10% to 0.10%)	624332.05 (551698.78 to 699593.56)	10996.08 (9447.74 to 12448.71)	0.00% (-0.01% to 0.00%)	0.860
France	21984828.03 (19299481.34 to 24782861.99)	31418.48 (27367.37 to 36027.39)	0.00% (0.00% to 0.00%)	55822.97 (16584.78 to 177931.90)	74.92 (20.53 to 253.03)	0.00% (-0.10% to 0.09%)	7363593.68 (6490652.24 to 8178996.93)	10893.20 (9425.25 to 12363.67)	0.00% (0.00% to 0.00%)	0.838
Germany	30566291.99 (26922507.68 to 34354087.07)	33107.73 (28985.82 to 37680.86)	0.02% (-0.02% to 0.06%)	71616.46 (20869.31 to 238118.17)	72.21 (19.84 to 254.64)	0.00% (-0.14% to 0.10%)	9630602.05 (8384014.31 to 10837886.53)	11012.92 (9501.26 to 12540.99)	0.00% (-0.03% to 0.03%)	0.903
Greece	3570883.28 (3140584.30 to 4030948.66)	32408.70 (28249.91 to 36985.43)	0.00% (0.00% to 0.01%)	8272.06 (2284.34 to 29200.64)	69.65 (17.78 to 256.03)	0.00% (-0.12% to 0.12%)	1150581.21 (1011300.32 to 1290099.70)	11001.95 (9465.27 to 12446.06)	0.00% (0.00% to 0.00%)	0.792
Hungary	3127052.91 (2742043.15 to 3503148.54)	29976.16 (26046.38 to 34344.52)	0.00% (0.00% to 0.00%)	8340.21 (2696.69 to 25492.29)	73.07 (22.02 to 241.63)	0.00% (-0.09% to 0.09%)	1052238.83 (920670.88 to 1169347.07)	10579.89 (9135.77 to 11922.77)	0.00% (0.00% to 0.00%)	0.791
Iceland	120532.39 (105593.34 to 136816.06)	32368.67 (28203.02 to 36964.11)	0.00% (0.00% to 0.00%)	269.32 (73.19 to 973.80)	69.37 (18.13 to 256.86)	0.00% (-0.11% to 0.11%)	39651.04 (34649.68 to 44726.43)	10991.29 (9457.04 to 12473.55)	0.00% (0.00% to 0.00%)	0.876
Ireland	1696845.31 (1488430.77 to 1916001.36)	32406.02 (28242.61 to 36967.09)	0.00% (0.00% to 0.00%)	3837.44 (1033.02 to 13761.09)	69.58 (17.98 to 257.28)	0.00% (-0.11% to 0.12%)	558468.89 (487529.21 to 627633.09)	11000.32 (9468.17 to 12461.84)	0.00% (0.00% to 0.00%)	0.874
Israel	3094744.19 (2703221.12 to 3511141.26)	32400.54 (28232.98 to 37007.16)	0.00% (0.00% to 0.00%)	6753.62 (1761.98 to 24927.52)	69.80 (17.93 to 260.03)	0.00% (-0.09% to 0.08%)	1045606.10 (906143.83 to 1180491.08)	11000.06 (9471.90 to 12441.52)	0.00% (0.00% to 0.00%)	0.809

Italy	21839653.34 (19560998.04 to 23983501.55)	33572.31 (29964.27 to 37532.66)	0.00% (-0.02% to 0.02%)	50176.42 (14083.58 to 183683.64)	71.46 (18.10 to 278.39)	0.01% (-0.06% to 0.08%)	7330400.90 (6462462.45 to 8145999.80)	11907.30 (10379.71 to 13336.30)	0.00% (-0.03% to 0.03%)	0.806
Latvia	602991.70 (532225.52 to 685044.74)	30338.15 (26401.78 to 34724.16)	0.00% (0.00% to 0.00%)	1952.02 (673.71 to 5673.06)	87.86 (28.02 to 271.83)	0.00% (-0.09% to 0.08%)	202290.39 (178485.34 to 226744.01)	10613.17 (9181.93 to 11978.28)	0.00% (0.00% to 0.00%)	0.831
Lithuania	924765.79 (816741.20 to 1037971.84)	31729.84 (27557.16 to 36200.23)	0.00% (0.00% to 0.00%)	2618.43 (870.43 to 8137.19)	81.03 (24.53 to 266.39)	0.00% (-0.10% to 0.09%)	300169.11 (262686.32 to 335948.28)	10764.37 (9260.52 to 12195.19)	0.00% (0.00% to 0.00%)	0.856
Luxembourg	228971.26 (201257.60 to 258806.73)	32279.96 (28111.40 to 36828.77)	0.00% (0.00% to 0.00%)	569.87 (173.89 to 1852.64)	74.98 (21.88 to 262.84)	0.00% (-0.11% to 0.08%)	74310.84 (64648.15 to 84091.99)	10969.42 (9428.47 to 12453.80)	0.00% (0.00% to 0.00%)	0.884
Malta	154171.27 (135412.36 to 173757.30)	32376.71 (28207.45 to 36976.61)	0.00% (-0.01% to 0.00%)	352.83 (96.90 to 1261.74)	69.25 (17.73 to 258.90)	-0.01% (-0.14% to 0.10%)	49789.55 (43846.76 to 55672.87)	10993.94 (9458.99 to 12439.10)	0.00% (0.00% to 0.00%)	0.802
Monaco	13089.19 (11419.18 to 14774.06)	32411.21 (28250.66 to 36983.37)	0.00% (0.00% to 0.00%)	30.62 (8.57 to 108.75)	69.94 (17.66 to 261.90)	0.00% (-0.10% to 0.14%)	4210.49 (3702.57 to 4729.86)	11000.46 (9455.21 to 12458.07)	0.00% (0.00% to 0.00%)	0.908
Montenegro	198591.57 (174528.53 to 224394.71)	29955.13 (26063.60 to 34324.21)	0.00% (0.00% to 0.00%)	520.50 (164.52 to 1621.39)	73.04 (21.40 to 242.42)	0.00% (-0.09% to 0.09%)	67661.41 (58761.09 to 75703.66)	10573.96 (9116.30 to 11920.21)	0.00% (0.00% to 0.00%)	0.796
Netherlands	6476061.47 (5683170.58 to 7328480.75)	34984.91 (30123.30 to 39942.65)	0.00% (0.00% to 0.00%)	13226.68 (3311.93 to 54846.54)	68.16 (15.73 to 292.36)	0.00% (-0.14% to 0.16%)	1961119.12 (1710397.30 to 2217020.52)	11114.04 (9423.86 to 12741.29)	0.00% (0.00% to 0.00%)	0.888
North Macedonia	718468.34 (631223.15 to 814777.23)	29923.19 (25998.04 to 34281.34)	0.00% (0.00% to 0.00%)	1892.63 (606.06 to 5825.83)	72.65 (21.86 to 241.46)	0.00% (-0.10% to 0.10%)	242894.20 (211010.66 to 272508.80)	10567.30 (9129.20 to 11916.74)	0.00% (0.00% to 0.00%)	0.751
Norway	2063243.43 (1850471.07 to 2279055.63)	35492.44 (31718.03 to 39481.91)	0.01% (-0.02% to 0.04%)	4344.01 (1107.11 to 16723.17)	71.25 (16.88 to 282.32)	0.03% (-0.06% to 0.13%)	683460.86 (601089.99 to 764089.28)	12225.67 (10673.08 to 13778.10)	0.01% (-0.02% to 0.04%)	0.916
Poland	13140254.87 (11726101.46 to 14550075.33)	31533.24 (28135.30 to 35109.35)	0.00% (0.00% to 0.00%)	35440.63 (11577.11 to 107825.89)	78.06 (23.62 to 254.86)	0.01% (-0.07% to 0.05%)	4525714.61 (3987722.83 to 5023474.89)	11365.29 (9873.17 to 12708.66)	0.00% (0.00% to 0.00%)	0.812
Portugal	3747590.35 (3292299.65 to 4229981.77)	32429.23 (28267.44 to 37011.93)	0.00% (0.00% to 0.01%)	8715.95 (2435.10 to 30614.89)	69.80 (17.90 to 255.82)	0.01% (-0.12% to 0.12%)	1204778.90 (1058832.92	11006.84 (9472.07 to 12441.46)	0.00% (0.00% to 0.00%)	0.744

							to 1352803.91)			
Republic of Moldova	1202123.83 (1055850.88 to 1368268.37)	30334.65 (26376.91 to 34792.29)	0.00% (0.00% to 0.00%)	3869.56 (1331.83 to 11073.73)	87.90 (28.60 to 267.41)	0.00% (-0.08% to 0.06%)	402477.91 (350885.36 to 455567.93)	10611.66 (9177.32 to 11960.03)	0.00% (0.00% to 0.00%)	0.732
Romania	6117197.60 (5362639.88 to 6865043.34)	29957.47 (26033.17 to 34318.15)	0.00% (0.00% to 0.00%)	16296.43 (5103.52 to 49964.00)	73.08 (21.09 to 241.38)	0.00% (-0.10% to 0.09%)	2068828.89 (1804544.84 to 2300926.25)	10575.83 (9131.70 to 11915.94)	0.00% (0.00% to 0.00%)	0.768
Russian Federation	48225991.18 (43156672.65 to 53273636.20)	31147.27 (27732.82 to 35055.65)	0.01% (-0.02% to 0.03%)	171991.22 (63374.06 to 460776.63)	99.72 (34.79 to 288.43)	0.00% (-0.03% to 0.03%)	16710291.76 (14611029.07 to 18612991.82)	11200.64 (9779.76 to 12544.09)	0.00% (-0.02% to 0.03%)	0.809
San Marino	11550.38 (10155.90 to 13020.42)	32430.84 (28280.21 to 36962.56)	0.00% (0.00% to 0.01%)	26.84 (7.54 to 95.17)	70.07 (18.33 to 260.01)	0.01% (-0.09% to 0.13%)	3731.03 (3295.12 to 4197.34)	11005.36 (9469.08 to 12460.00)	0.00% (0.00% to 0.00%)	0.888
Serbia	2937511.06 (2582145.71 to 3314003.05)	29928.17 (26039.72 to 34277.48)	0.00% (-0.01% to 0.00%)	7706.93 (2462.84 to 23767.31)	73.08 (21.71 to 242.74)	0.00% (-0.10% to 0.08%)	994039.03 (868734.74 to 1107999.13)	10566.41 (9126.68 to 11909.54)	0.00% (-0.01% to 0.00%)	0.792
Slovakia	1768339.92 (1551603.50 to 1994579.56)	29962.05 (26021.42 to 34279.58)	0.00% (-0.01% to 0.00%)	4705.60 (1515.18 to 14451.14)	73.13 (21.84 to 243.89)	0.00% (-0.09% to 0.10%)	596767.17 (521035.46 to 665684.66)	10576.74 (9135.52 to 11920.58)	0.00% (0.00% to 0.00%)	0.811
Slovenia	667491.14 (581256.30 to 748537.64)	29927.39 (26032.45 to 34300.82)	0.00% (-0.01% to 0.00%)	1785.77 (582.53 to 5402.57)	72.89 (21.48 to 241.72)	0.00% (-0.10% to 0.10%)	224816.70 (196727.06 to 250133.70)	10568.36 (9125.70 to 11919.72)	0.00% (-0.01% to 0.00%)	0.842
Spain	15640493.70 (13676285.21 to 17704584.91)	31535.63 (27408.60 to 36010.06)	0.00% (0.00% to 0.01%)	39938.92 (11640.26 to 133191.27)	73.69 (20.11 to 267.06)	0.01% (-0.10% to 0.14%)	5146361.00 (4510583.95 to 5786216.14)	10896.83 (9420.95 to 12313.98)	0.00% (0.00% to 0.00%)	0.769
Sweden	3771717.69 (3381333.58 to 4156615.55)	34297.27 (30701.03 to 38326.02)	0.00% (-0.02% to 0.03%)	7850.08 (2084.21 to 28634.82)	68.31 (17.15 to 259.89)	0.00% (-0.09% to 0.09%)	1263655.49 (1114822.40 to 1402628.89)	11957.24 (10503.70 to 13393.96)	0.00% (-0.03% to 0.03%)	0.887
Switzerland	2884248.94 (2516060.47 to 3265068.87)	29829.20 (25864.17 to 34171.76)	0.05% (0.00% to 0.11%)	6877.39 (1979.57 to 23037.84)	66.36 (18.13 to 241.18)	0.03% (-0.10% to 0.12%)	991842.08 (864090.08 to 1108088.22)	10713.80 (9241.01 to 12024.54)	0.01% (-0.02% to 0.05%)	0.933
Ukraine	15063001.30 (13449448.58 to 16721250.84)	31872.74 (28432.01 to 35612.60)	0.00% (0.00% to 0.00%)	48418.19 (16949.09 to 137600.83)	91.72 (29.95 to 277.45)	0.00% (-0.07% to 0.07%)	5152557.31 (4530439.38 to 5776198.43)	11405.07 (9946.67 to 12812.49)	0.00% (0.00% to 0.00%)	0.761

United Kingdom	24294167.52 (21795300.59 to 26912634.21)	33487.02 (29942.12 to 37299.08)	0.00% (0.00% to 0.00%)	55187.88 (15436.92 to 197681.55)	72.27 (19.01 to 266.15)	0.00% (-0.06% to 0.03%)	8247656.08 (7234881.30 to 9220731.37)	11812.17 (10329.04 to 13276.29)	0.00% (0.00% to 0.00%)	0.859
**Statistical Test for SDI**	Spearman's ρ = -0.131 p-value = 0.392	Spearman's ρ = 0.522 p-value < 0.001		Spearman's ρ = -0.170 p-value = 0.265	Spearman's ρ = -0.466 p-value = 0.001.		Spearman's ρ = -0.138 p-value = 0.367	Spearman's ρ = 0.474 p-value < 0.001		

Supplementary Figures

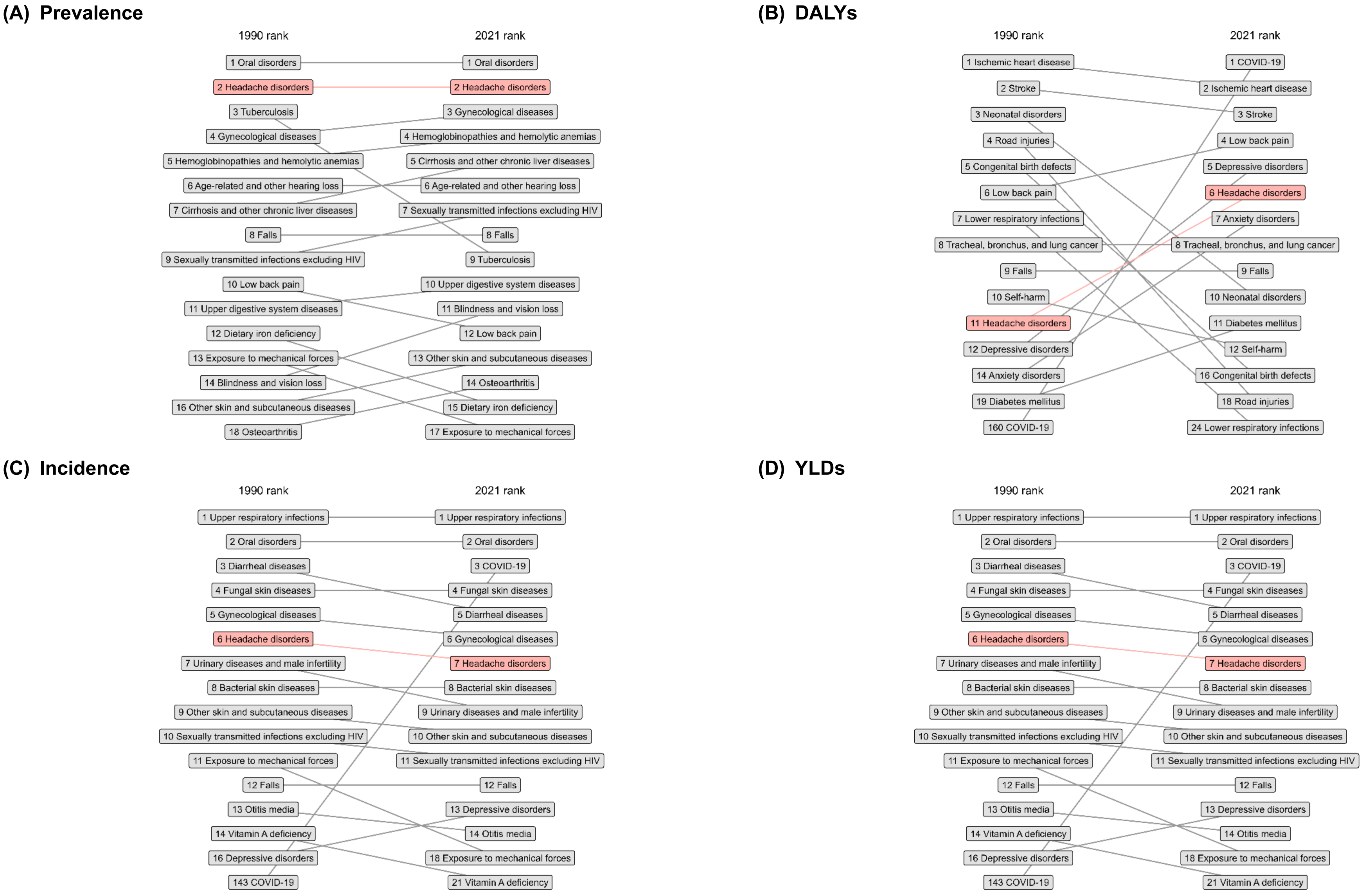

Figure S1a: Changes in top ranks of age-standardized (A) prevalence, (B) DALY, (C) incidence, and (D) YLD rates for cause level 3 conditions from 1990 to 2021

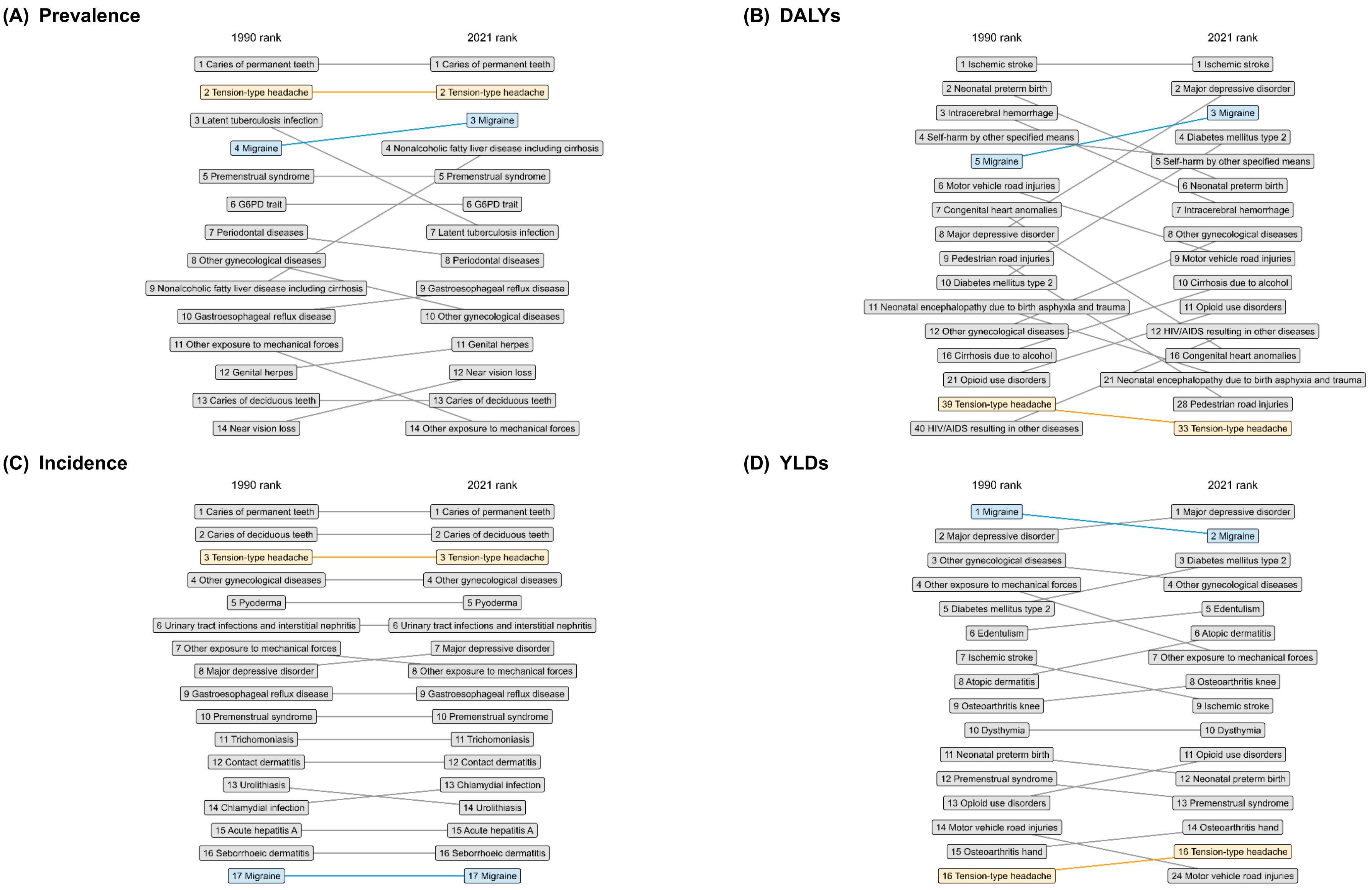

Figure S1b: Changes in top ranks of age-standardized (A) prevalence, (B) DALY, (C) incidence, and (D) YLD rates for cause level 4 conditions from 1990 to 2021

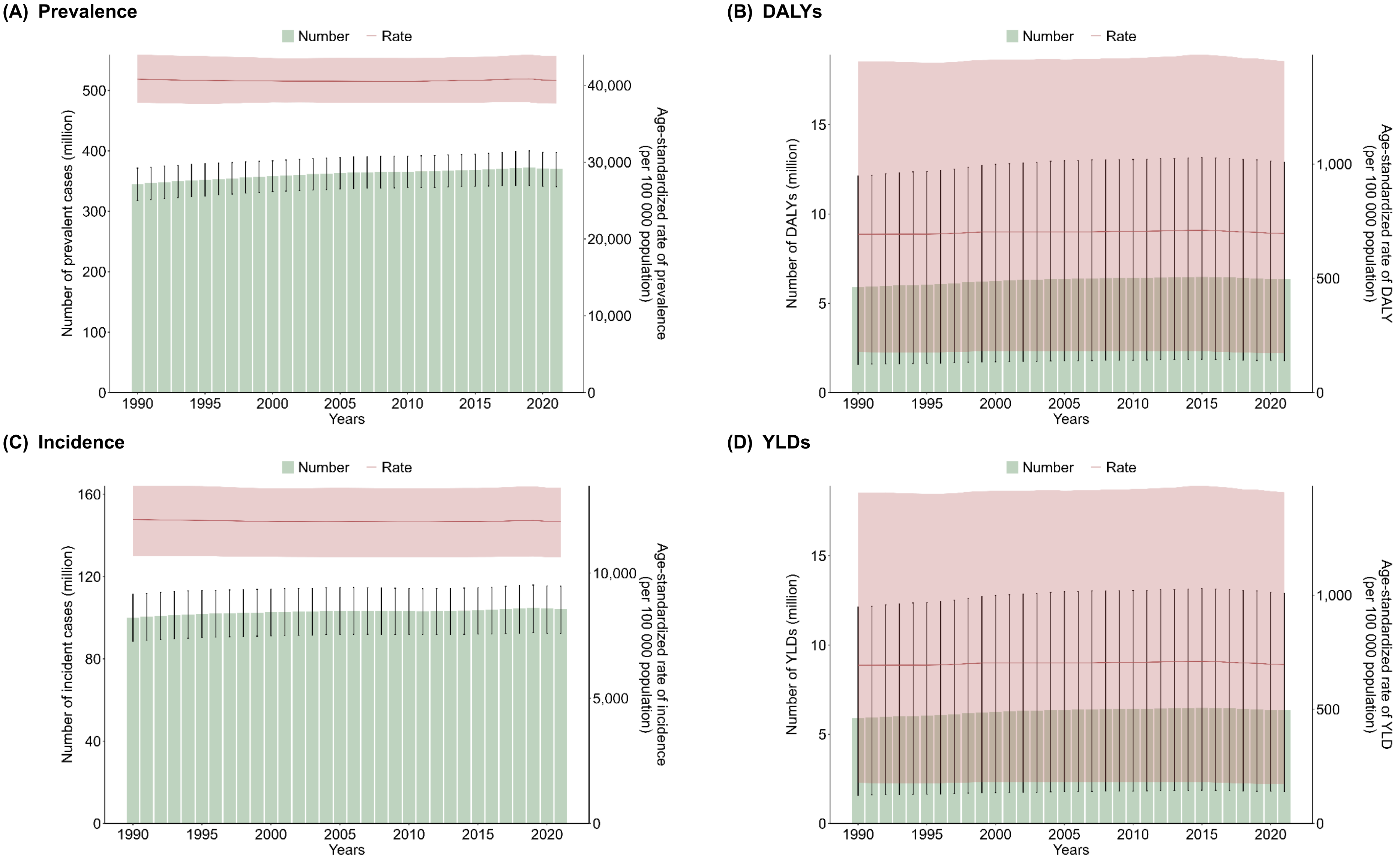

Figure S2a: Age-standardized (A) prevalence, (B) DALY, (C) incidence, and (D) YLD rates and counts of headache disorders by year, 1990-2021

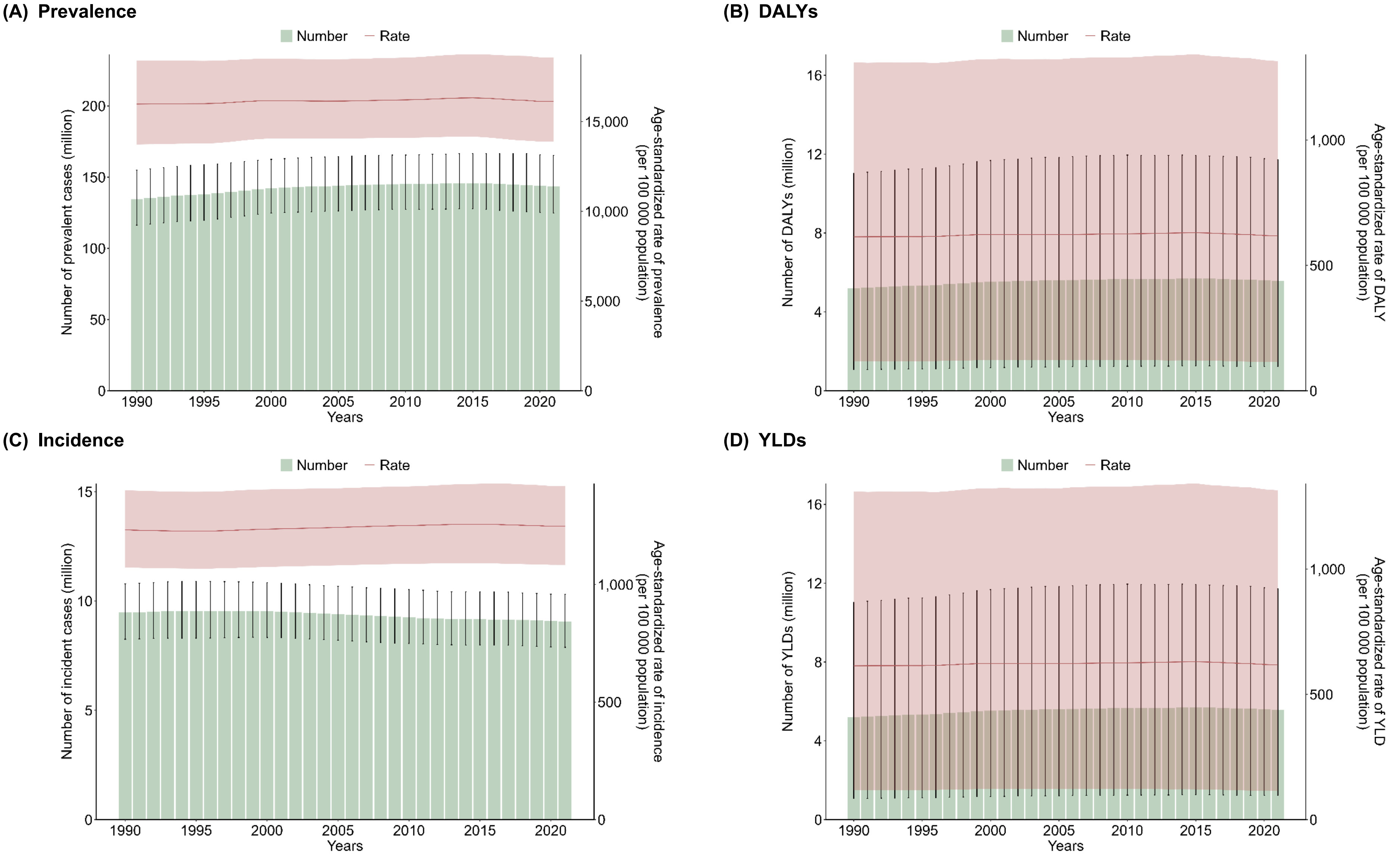

Figure S2b: Age-standardized (A) prevalence, (B) DALY, (C) incidence, and (D) YLD rates and counts of migraine by year, 1990-2021

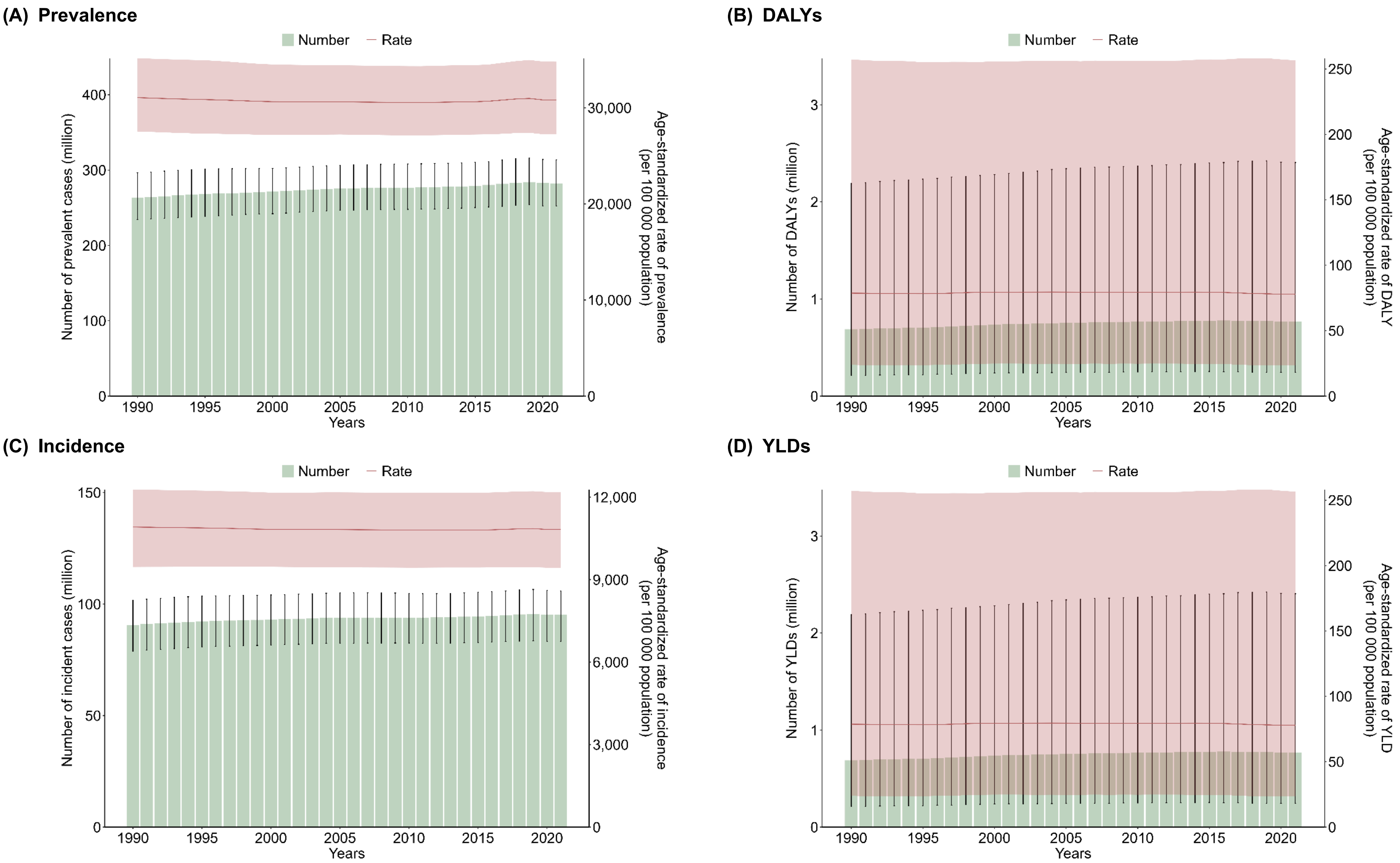

Figure S2c: Age-standardized (A) prevalence, (B) DALY, (C) incidence, and (D) YLD rates and counts of tension-type headache by year, 1990-2021

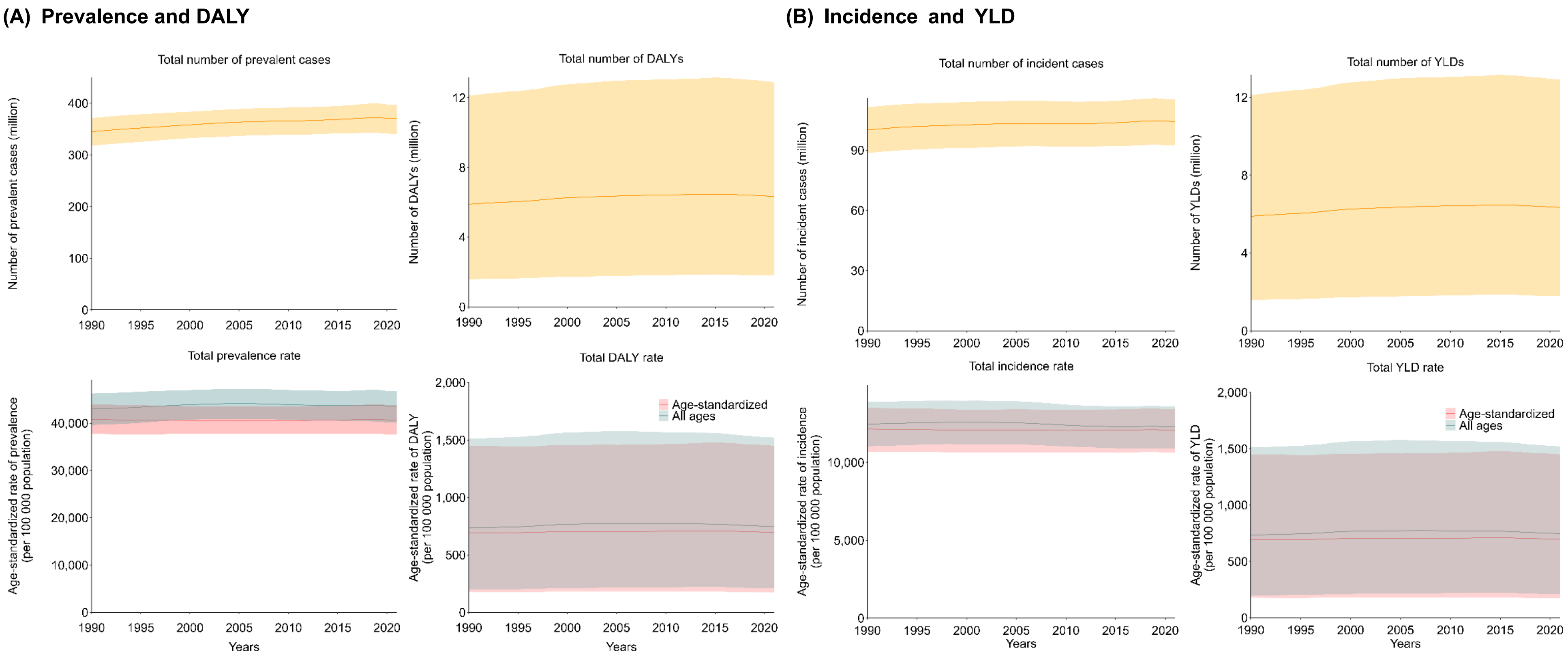

Figure S3a: Age-standardized (A) prevalence and DALY, and (B) incidence and YLD rates and counts of headache disorders by year, 1990-2021

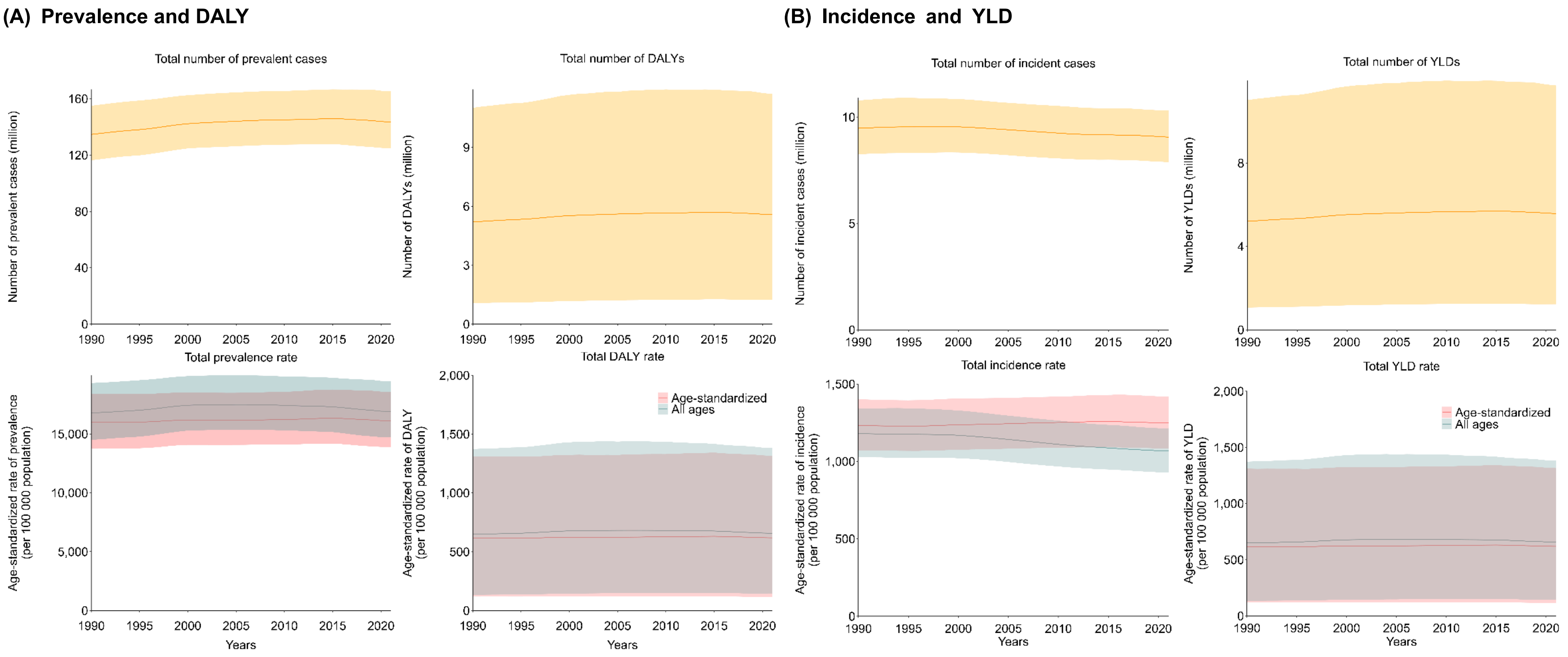

Figure S3b: Age-standardized (A) prevalence and DALY, and (B) incidence and YLD rates and counts of migraine by year, 1990-2021

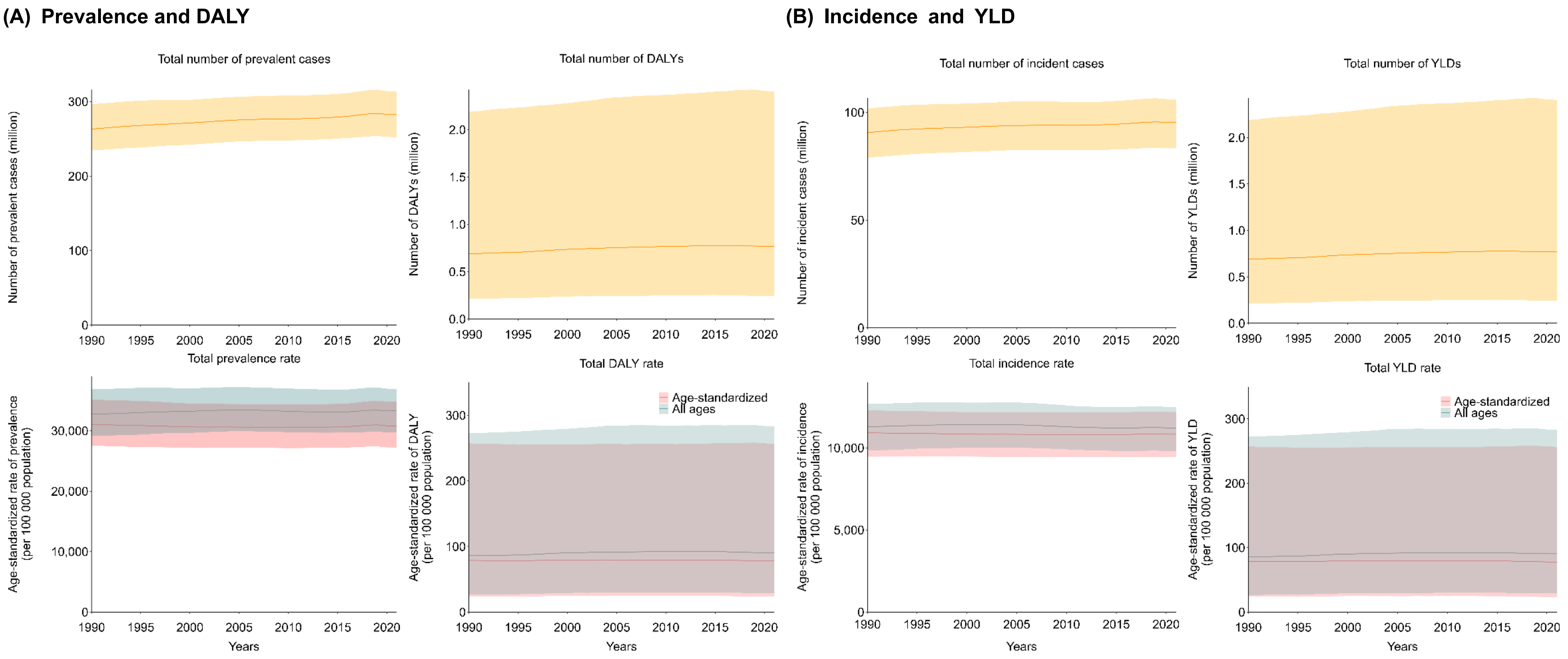

Figure S3c: Age-standardized (A) prevalence and DALY, and (B) incidence and YLD rates and counts of tension-type headache by year, 1990-2021

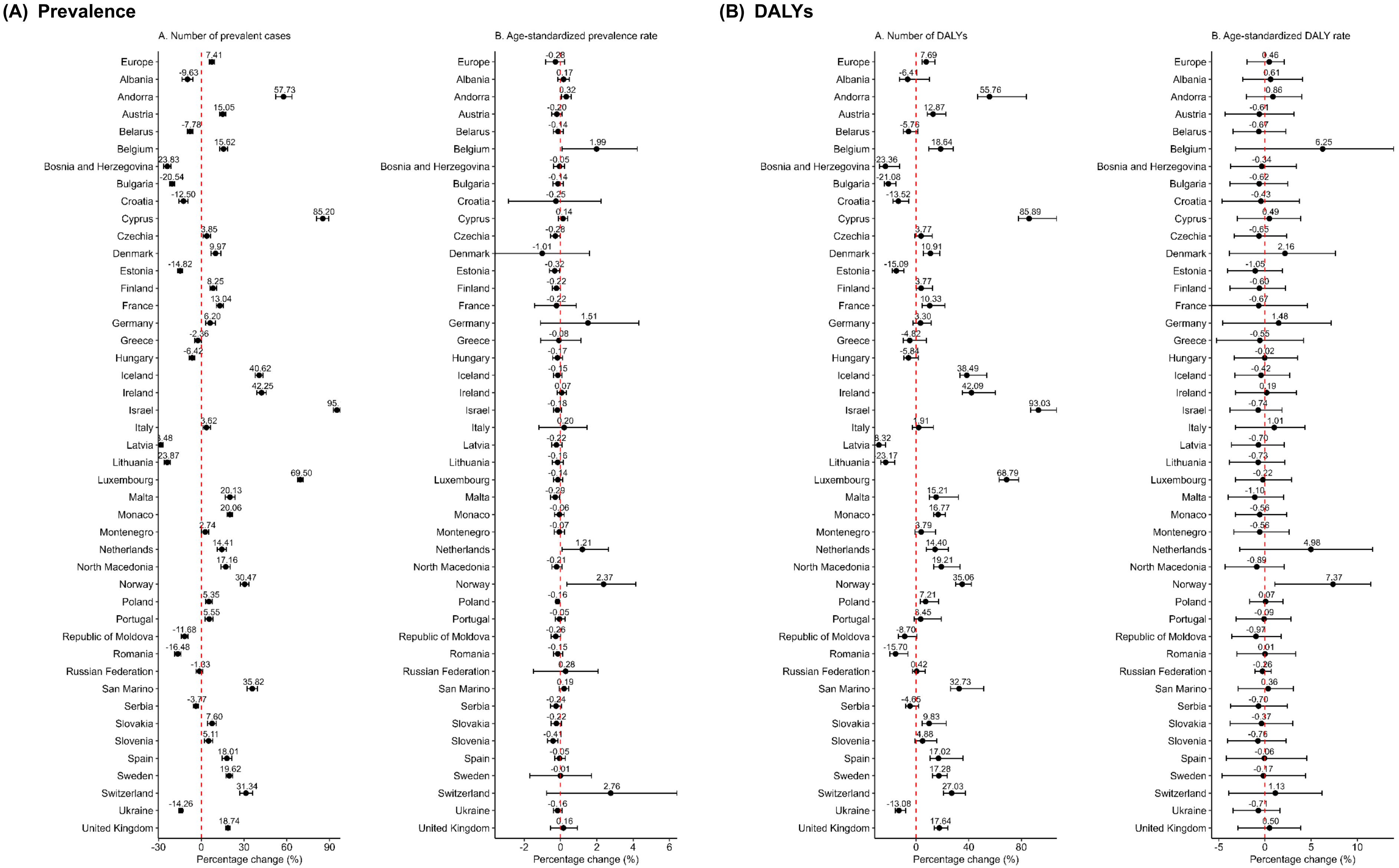

(A) Prevalence
A. Number of prevalent cases
Europe 7.41
Albania -9.63
Andorra 57.73
Austria 15.05
Belarus -7.78
Belgium 15.62
Bosnia and Herzegovina 23.83
Bulgaria -20.54
Croatia -12.50
Cyprus 85.20
Czechia 3.85
Denmark 9.97
Estonia -14.82
Finland 8.25
France 13.04
Germany 6.20
Greece -2.36
Hungary -6.42
Iceland 40.62
Ireland 42.25
Israel 95.
Italy 3.62
Latvia 3.48
Lithuania 23.87
Luxembourg 69.50
Malta 20.13
Monaco 20.06
Montenegro 2.74
Netherlands 14.41
North Macedonia 17.16
Norway 30.47
Poland 5.35
Portugal 5.55
Republic of Moldova -11.68
Romania -16.48
Russian Federation -1.83
San Marino 35.82
Serbia -3.77
Slovakia 7.60
Slovenia 5.11
Spain 18.01
Sweden 19.62
Switzerland 31.34
Ukraine -14.26
United Kingdom 18.74
-30 0 30 60 90
Percentage change (%)
B. Age-standardized prevalence rate
Europe -0.28
Albania 0.17
Andorra 0.32
Austria -0.20
Belarus -0.14
Belgium 1.99
Bosnia and Herzegovina -0.05
Bulgaria -0.14
Croatia -0.25
Cyprus 0.14
Czechia -0.28
Denmark -1.01
Estonia -0.32
Finland -0.22
France -0.22
Germany 1.51
Greece -0.08
Hungary -0.17
Iceland -0.15
Ireland 0.07
Israel -0.18
Italy 0.20
Latvia -0.22
Lithuania -0.16
Luxembourg -0.14
Malta -0.29
Monaco -0.06
Montenegro -0.07
Netherlands 1.21
North Macedonia -0.21
Norway 2.37
Poland -0.16
Portugal -0.05
Republic of Moldova -0.26
Romania -0.15
Russian Federation 0.28
San Marino 0.19
Serbia -0.24
Slovakia -0.22
Slovenia -0.41
Spain -0.05
Sweden -0.01
Switzerland 2.76
Ukraine -0.16
United Kingdom 0.16
-2 0 2 4 6
Percentage change (%)
(B) DALYs
A. Number of DALYs
Europe 7.69
Albania -6.41
Andorra 55.76
Austria 12.87
Belarus -5.76
Belgium 18.64
Bosnia and Herzegovina 23.36
Bulgaria -21.08
Croatia -13.52
Cyprus 85.89
Czechia 3.77
Denmark 10.91
Estonia -15.09
Finland 3.77
France 10.33
Germany 3.30
Greece -4.82
Hungary -5.84
Iceland 38.49
Ireland 42.09
Israel 93.03
Italy 1.91
Latvia 8.32
Lithuania 23.17
Luxembourg 68.79
Malta 15.21
Monaco 16.77
Montenegro 3.79
Netherlands 14.40
North Macedonia 19.21
Norway 35.06
Poland 7.21
Portugal 8.45
Republic of Moldova -8.70
Romania -15.70
Russian Federation 0.42
San Marino 32.73
Serbia -4.65
Slovakia 9.83
Slovenia 4.88
Spain 17.02
Sweden 17.28
Switzerland 27.03
Ukraine -13.08
United Kingdom 17.64
0 40 80
Percentage change (%)
B. Age-standardized DALY rate
Europe 0.46
Albania 0.61
Andorra 0.86
Austria -0.61
Belarus -0.67
Belgium 6.25
Bosnia and Herzegovina -0.34
Bulgaria -0.62
Croatia -0.43
Cyprus 0.49
Czechia -0.65
Denmark 2.16
Estonia -1.05
Finland -0.60
France -0.67
Germany 1.48
Greece -0.55
Hungary -0.02
Iceland -0.42
Ireland 0.19
Israel -0.74
Italy 1.01
Latvia -0.70
Lithuania -0.73
Luxembourg -0.22
Malta -1.10
Monaco -0.56
Montenegro -0.56
Netherlands 4.98
North Macedonia -0.89
Norway 7.37
Poland 0.07
Portugal -0.09
Republic of Moldova -0.97
Romania 0.01
Russian Federation -0.26
San Marino 0.36
Serbia -0.70
Slovakia -0.37
Slovenia -0.76
Spain -0.06
Sweden -0.17
Switzerland 1.13
Ukraine -0.71
United Kingdom 0.50
-5 0 5 10
Percentage change (%)

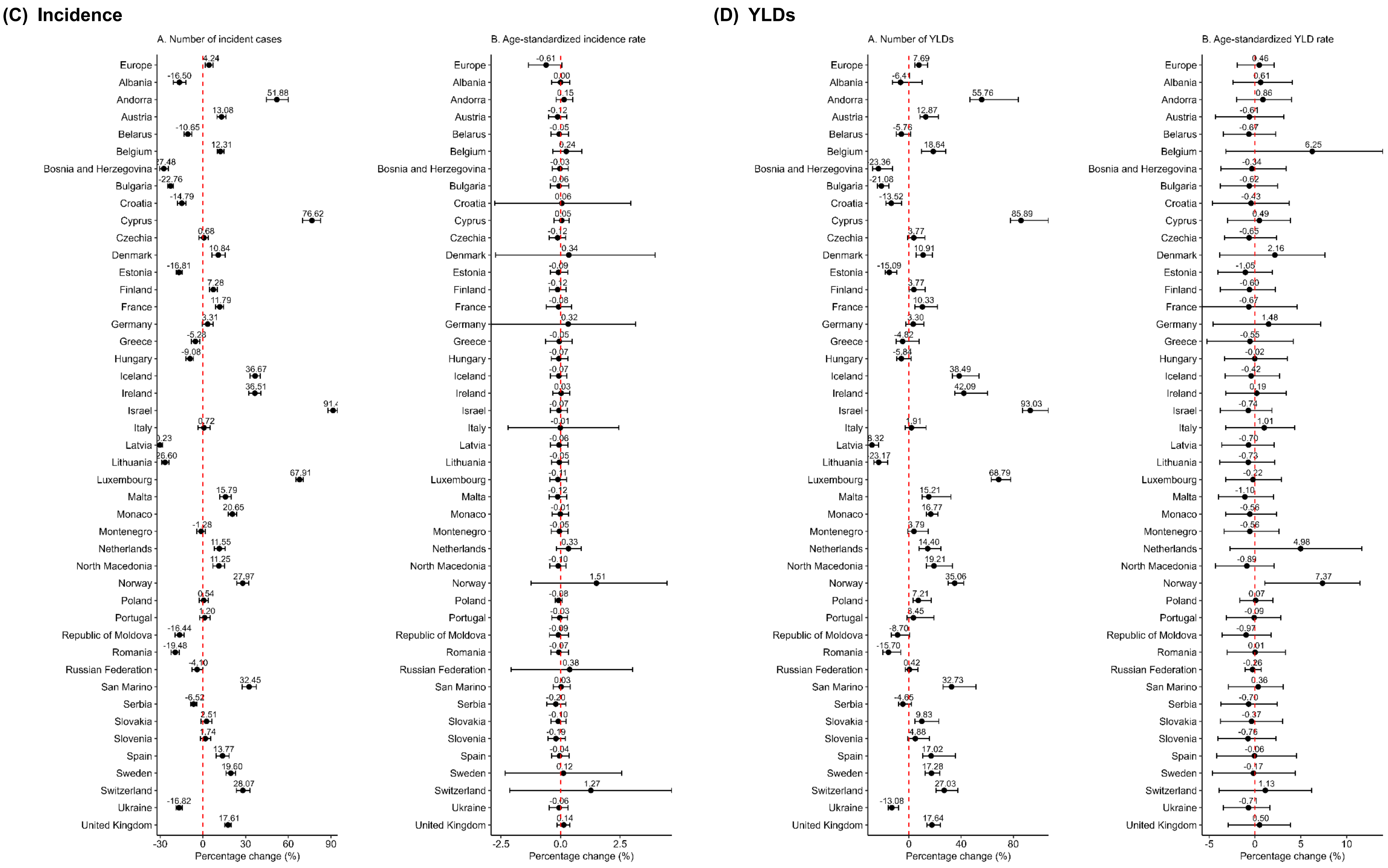

Figure S4a: Region-wise percentage changes in (A) prevalence, (B) DALY, (C) incidence, and (D) YLD in headache disorders burden, 1990–2021

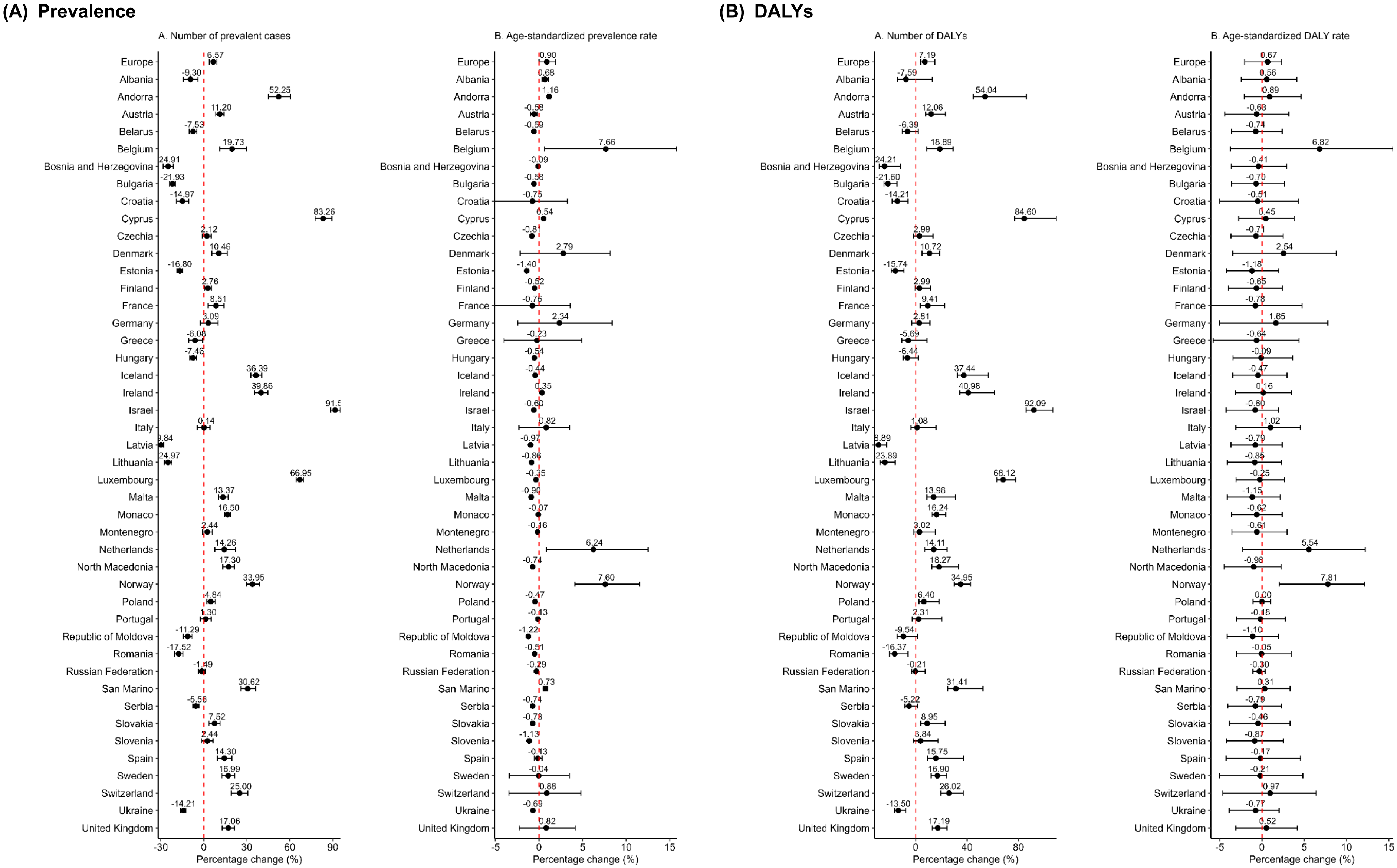
(A) Prevalence
A. Number of prevalent cases
B. Age-standardized prevalence rate
(B) DALYs
A. Number of DALYs
B. Age-standardized DALY rate
Europe
Albania
Andorra
Austria
Belarus
Belgium
Bosnia and Herzegovina
Bulgaria
Croatia
Cyprus
Czechia
Denmark
Estonia
Finland
France
Germany
Greece
Hungary
Iceland
Ireland
Israel
Italy
Latvia
Lithuania
Luxembourg
Malta
Monaco
Montenegro
Netherlands
North Macedonia
Norway
Poland
Portugal
Republic of Moldova
Romania
Russian Federation
San Marino
Serbia
Slovakia
Slovenia
Spain
Sweden
Switzerland
Ukraine
United Kingdom
Percentage change (%)

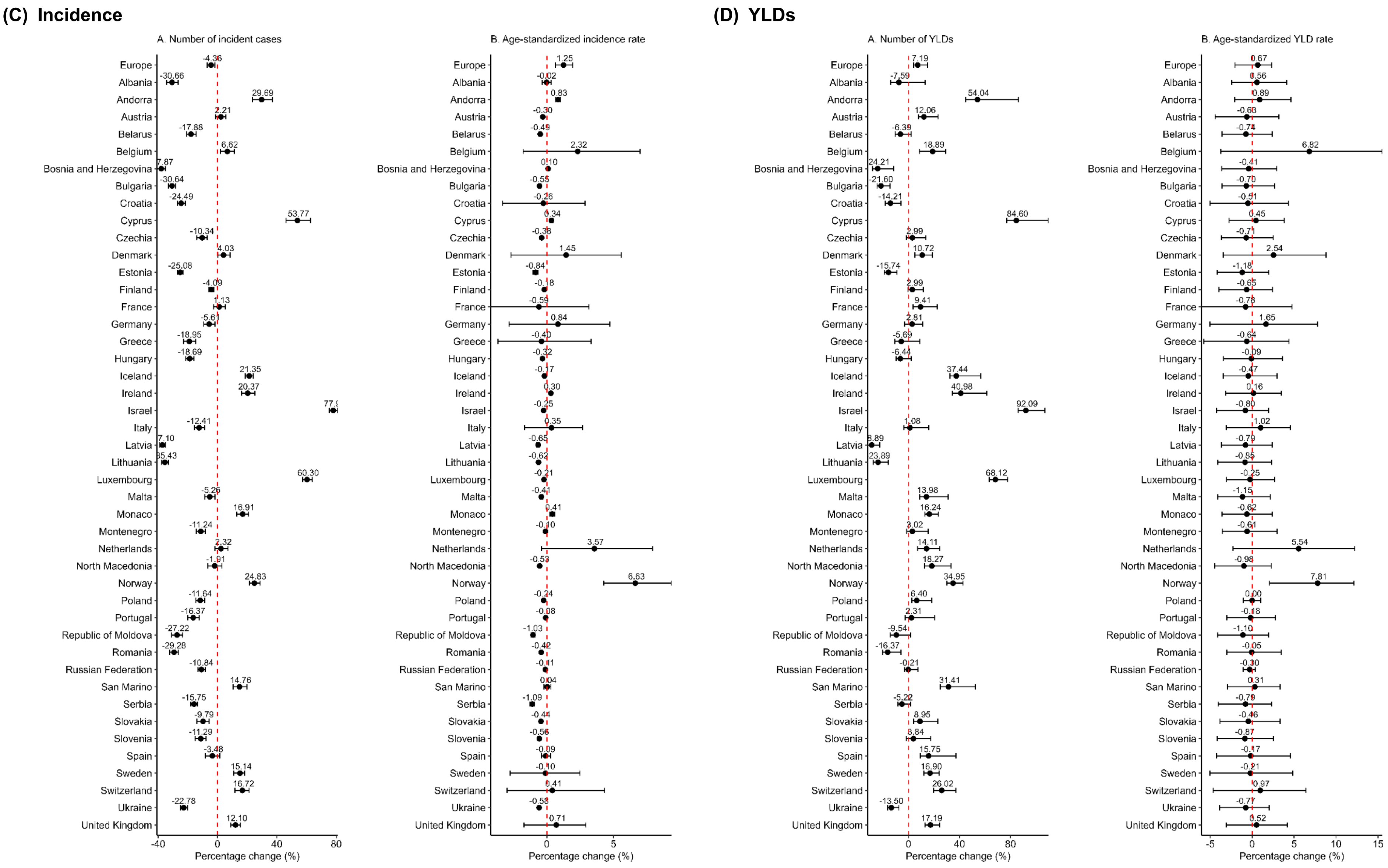

Figure S4b: Region-wise percentage changes in (A) prevalence, (B) DALY, (C) incidence, and (D) YLD in migraine burden, 1990–2021

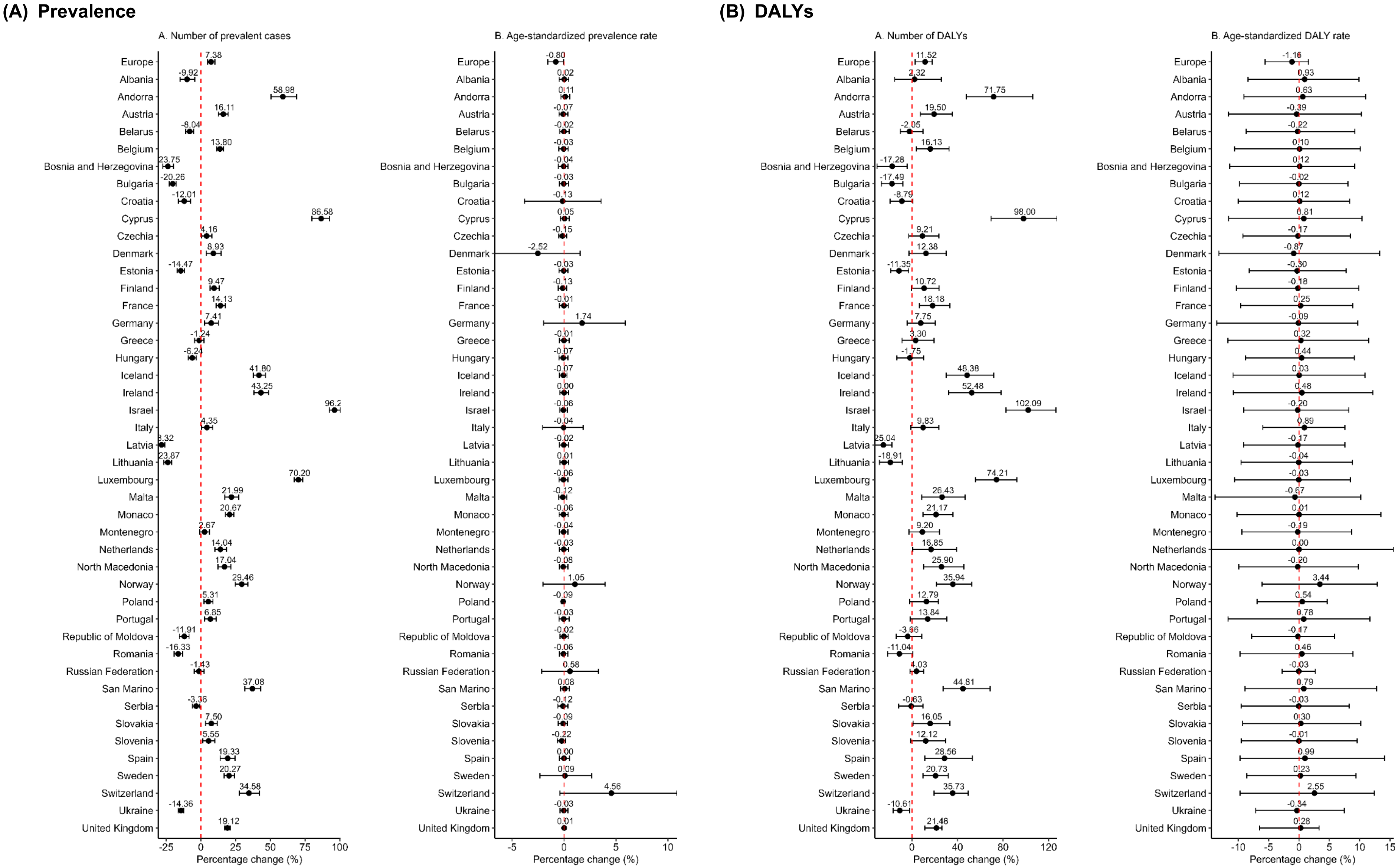
(A) Prevalence
A. Number of prevalent cases
B. Age-standardized prevalence rate
(B) DALYs
A. Number of DALYs
B. Age-standardized DALY rate
Europe
Albania
Andorra
Austria
Belarus
Belgium
Bosnia and Herzegovina
Bulgaria
Croatia
Cyprus
Czechia
Denmark
Estonia
Finland
France
Germany
Greece
Hungary
Iceland
Ireland
Israel
Italy
Latvia
Lithuania
Luxembourg
Malta
Monaco
Montenegro
Netherlands
North Macedonia
Norway
Poland
Portugal
Republic of Moldova
Romania
Russian Federation
San Marino
Serbia
Slovakia
Slovenia
Spain
Sweden
Switzerland
Ukraine
United Kingdom
Percentage change (%)

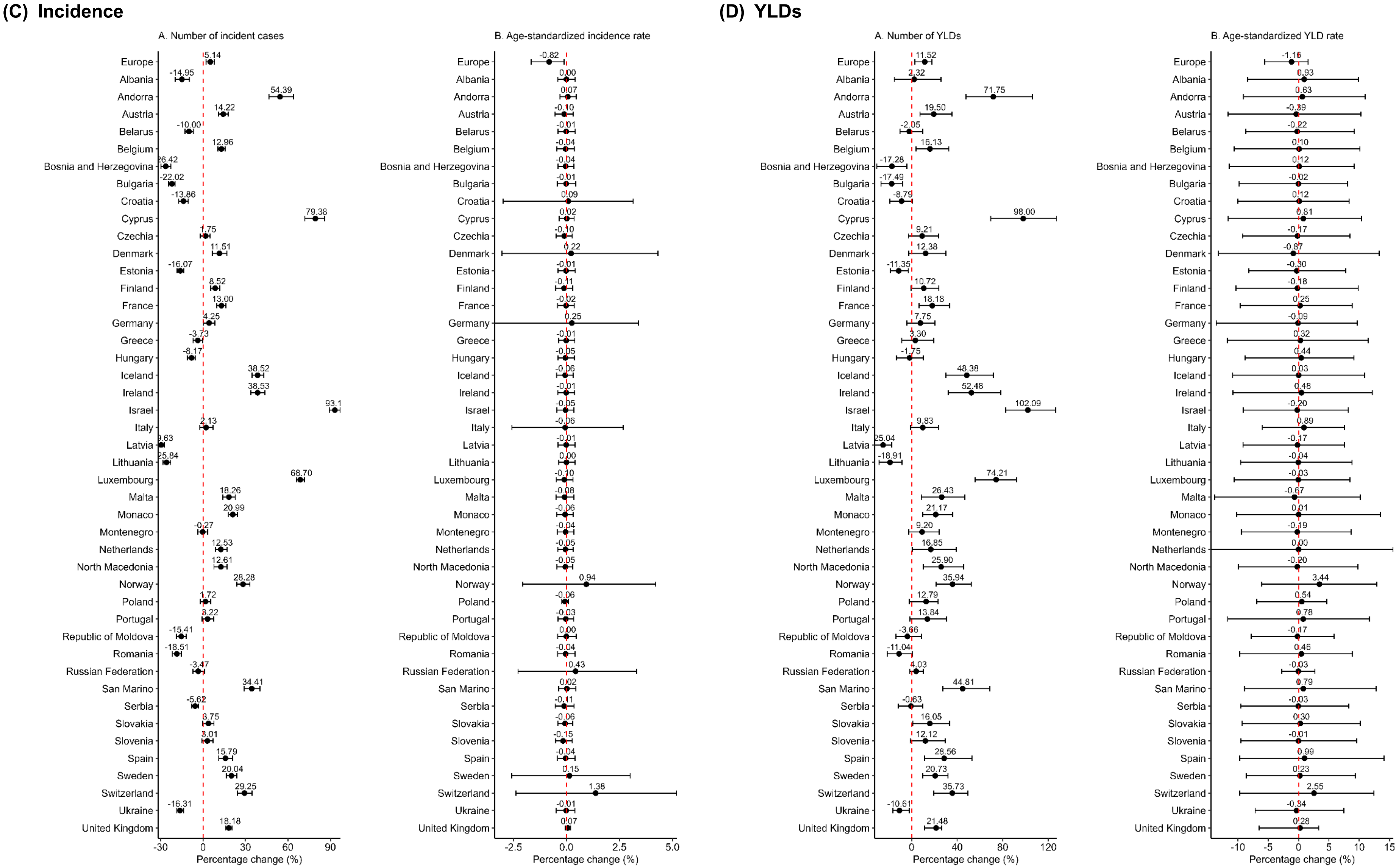

Figure S4c: Region-wise percentage changes in (A) prevalence, (B) DALY, (C) incidence, and (D) YLD in tension-type headache burden, 1990–2021

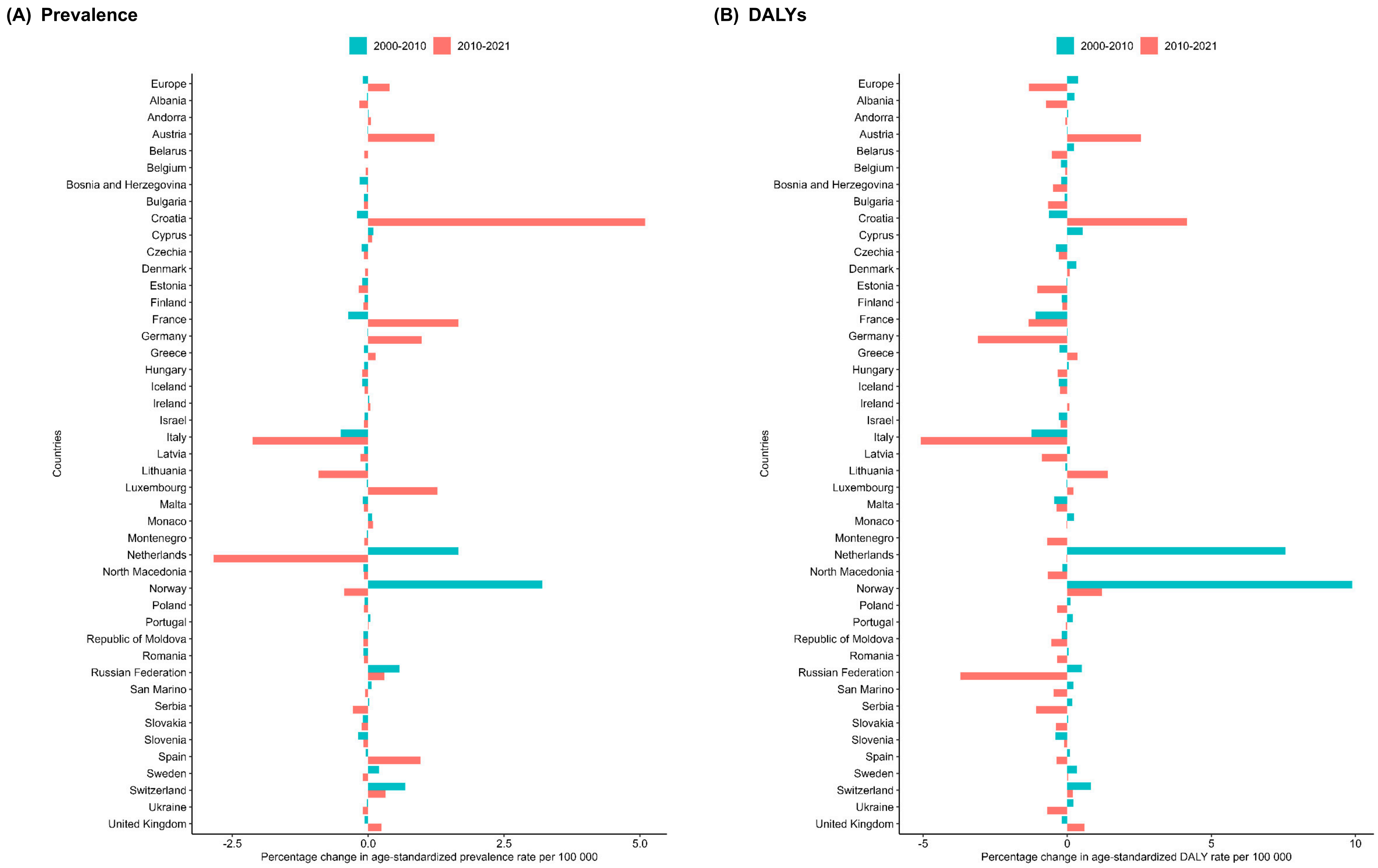
(A) Prevalence
2000-2010
2010-2021
Europe
Albania
Andorra
Austria
Belarus
Belgium
Bosnia and Herzegovina
Bulgaria
Croatia
Cyprus
Czechia
Denmark
Estonia
Finland
France
Germany
Greece
Hungary
Iceland
Ireland
Israel
Italy
Latvia
Lithuania
Luxembourg
Malta
Monaco
Montenegro
Netherlands
North Macedonia
Norway
Poland
Portugal
Republic of Moldova
Romania
Russian Federation
San Marino
Serbia
Slovakia
Slovenia
Spain
Sweden
Switzerland
Ukraine
United Kingdom
Countries
-2.5
0.0
2.5
5.0
Percentage change in age-standardized prevalence rate per 100 000
(B) DALYs
2000-2010
2010-2021
Countries
-5
0
5
10
Percentage change in age-standardized DALY rate per 100 000

(C) Incidence

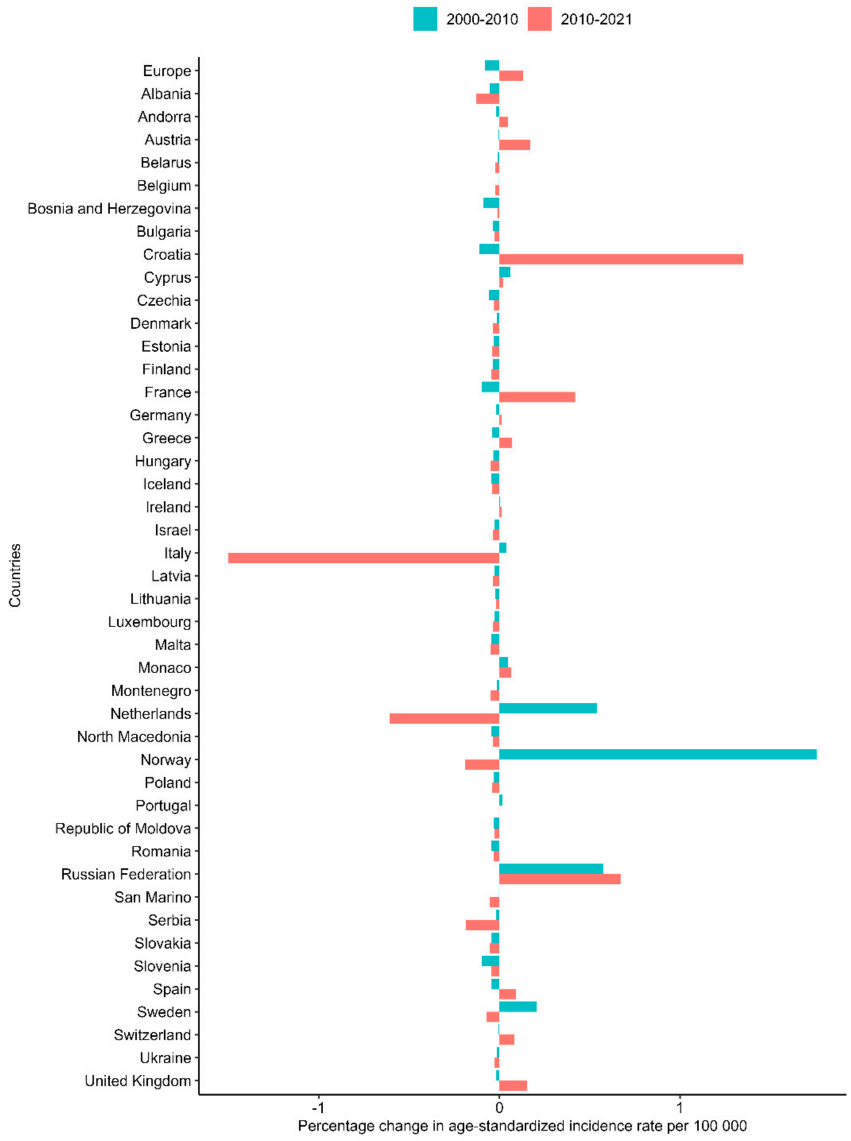

(D) YLDs

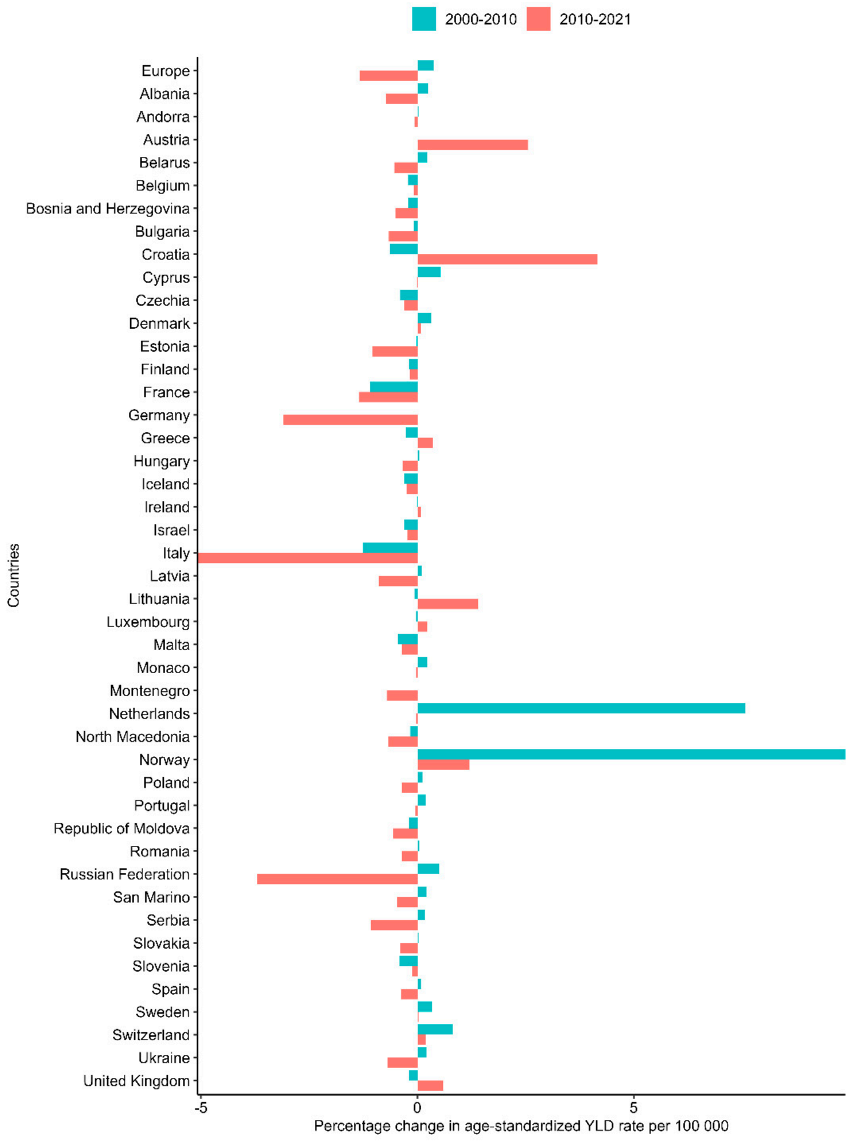

Figure S5a: Changes in age-standardized (A) prevalence, (B) DALY, (C) incidence, and (D) YLD rate for headache disorders, by country, 2000-2010 and 2010-2021

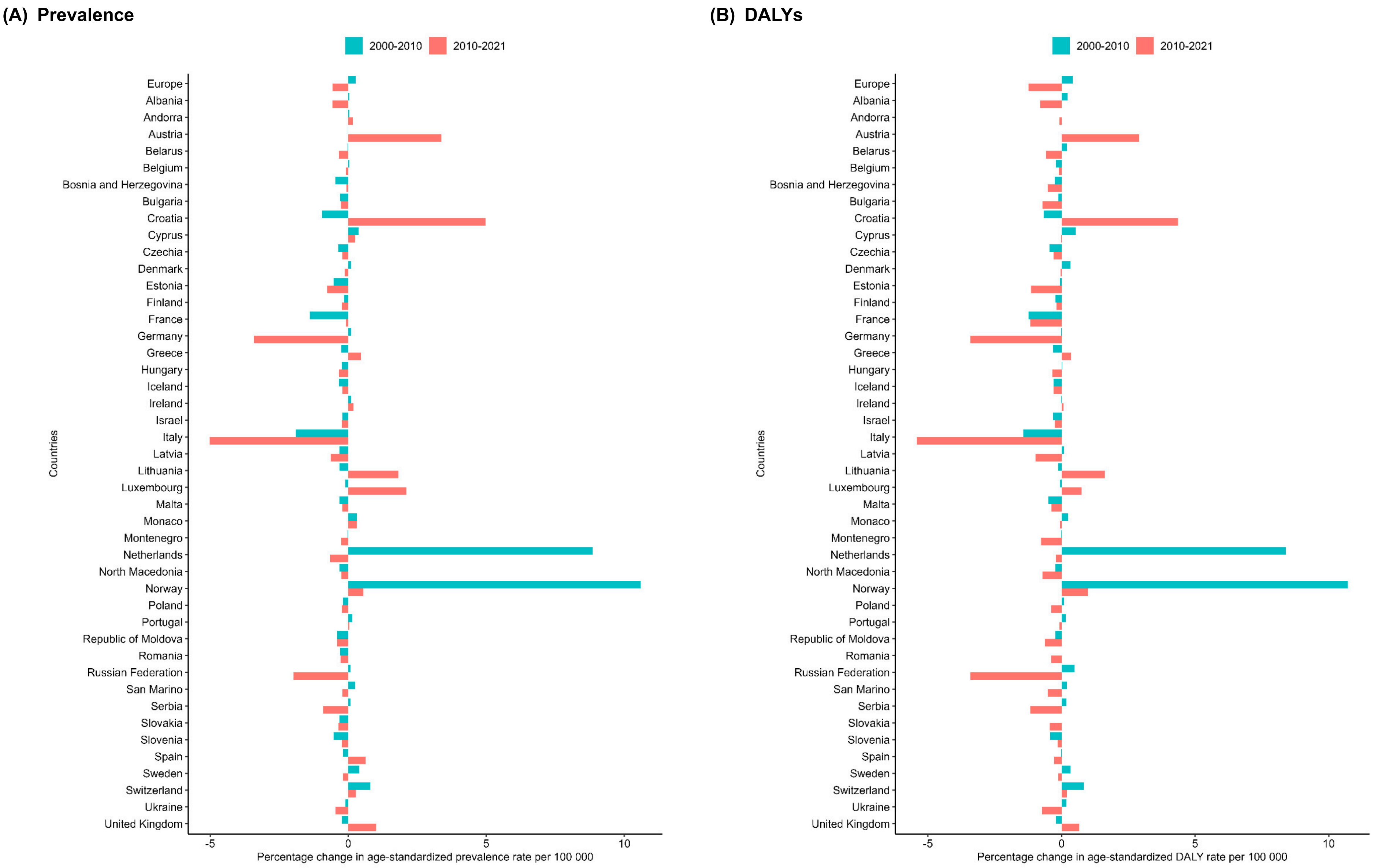
(A) Prevalence
2000-2010
2010-2021
Countries
Europe
Albania
Andorra
Austria
Belarus
Belgium
Bosnia and Herzegovina
Bulgaria
Croatia
Cyprus
Czechia
Denmark
Estonia
Finland
France
Germany
Greece
Hungary
Iceland
Ireland
Israel
Italy
Latvia
Lithuania
Luxembourg
Malta
Monaco
Montenegro
Netherlands
North Macedonia
Norway
Poland
Portugal
Republic of Moldova
Romania
Russian Federation
San Marino
Serbia
Slovakia
Slovenia
Spain
Sweden
Switzerland
Ukraine
United Kingdom
-5
0
5
10
Percentage change in age-standardized prevalence rate per 100 000
(B) DALYs
2000-2010
2010-2021
Countries
-5
0
5
10
Percentage change in age-standardized DALY rate per 100 000

(C) Incidence

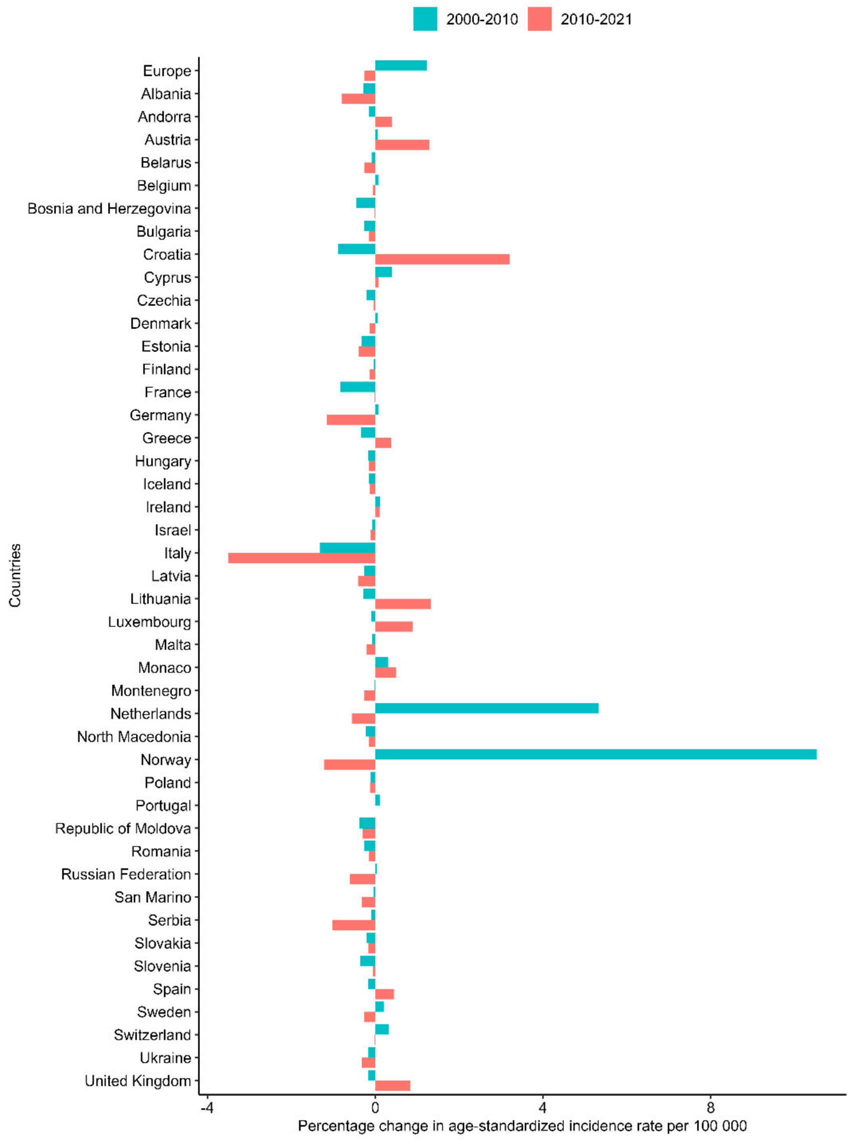

(D) YLDs

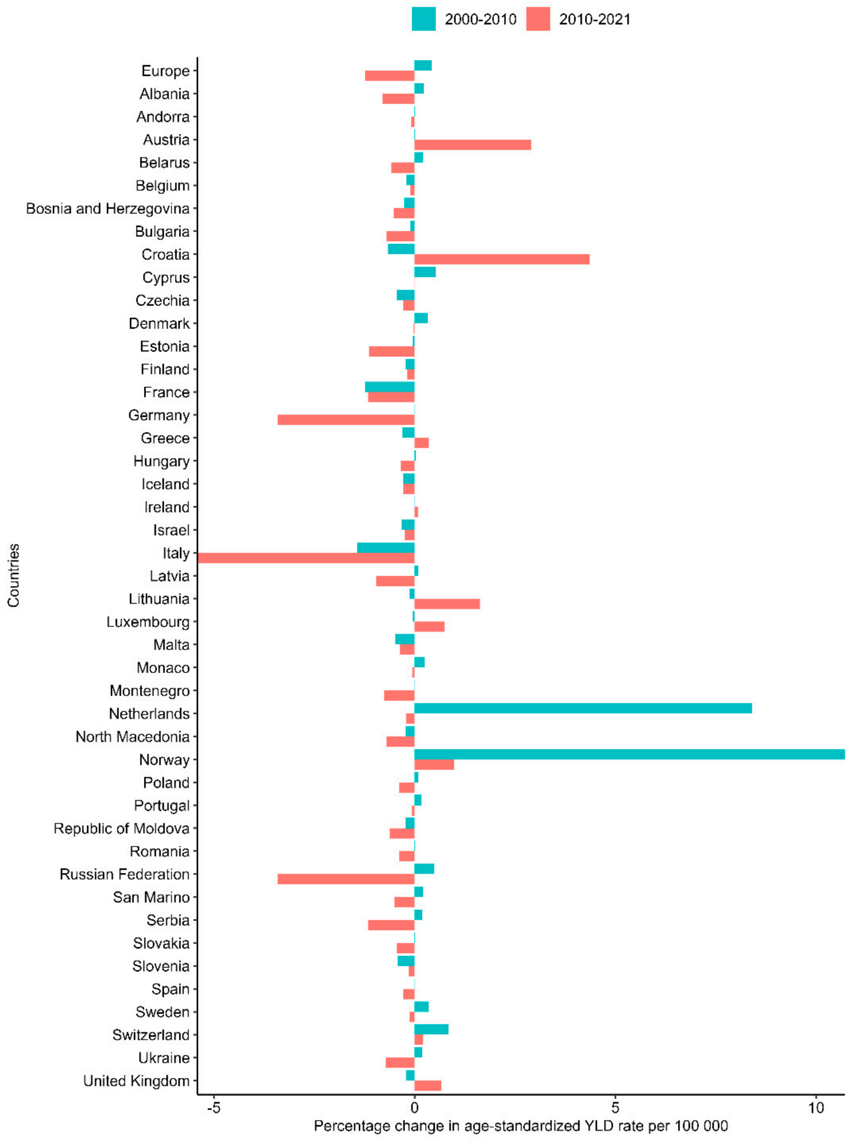

Figure S5b: Changes in age-standardized (A) prevalence, (B) DALY, (C) incidence, and (D) YLD rate for migraine, by country, 2000-2010 and 2010-2021

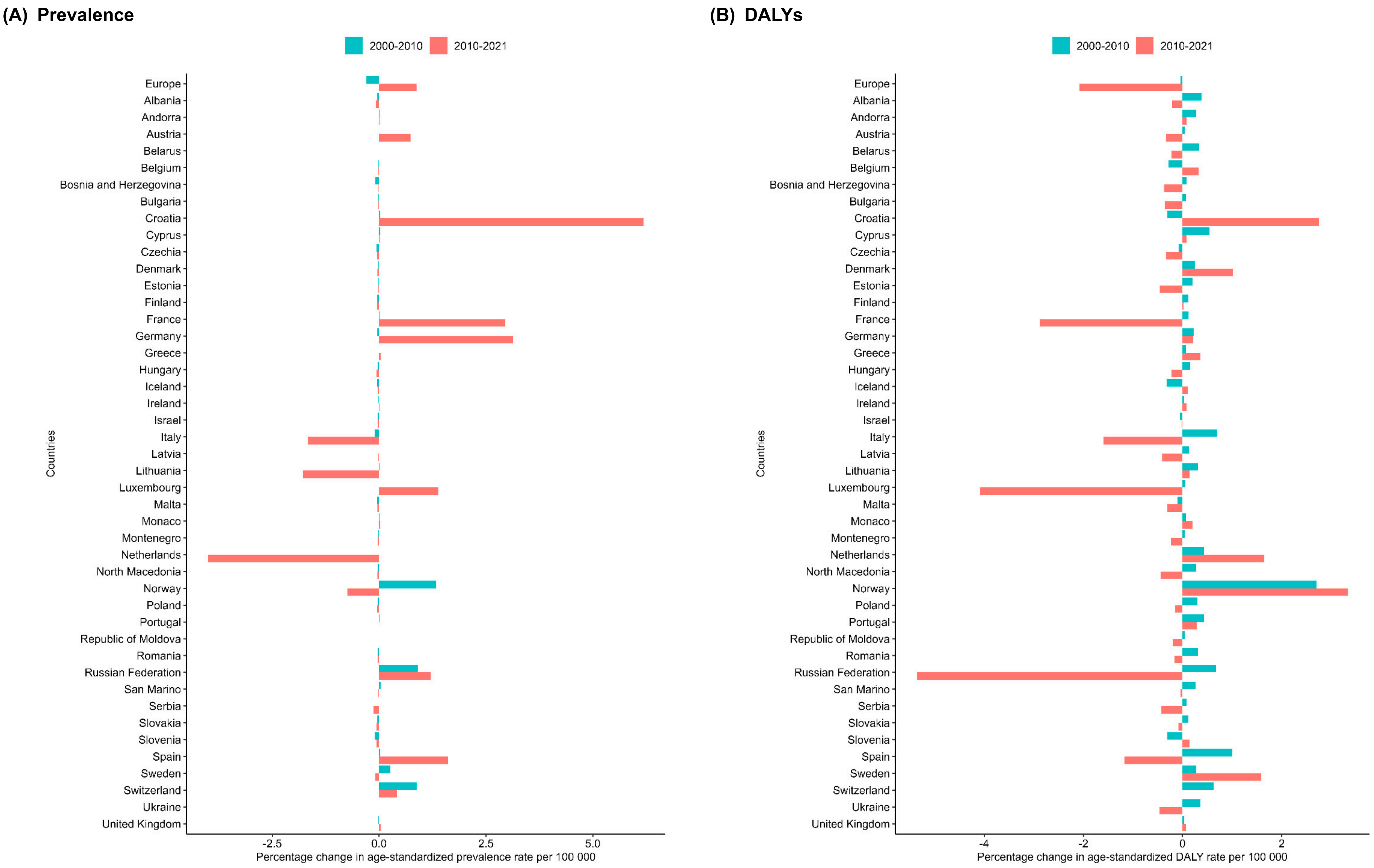
(A) Prevalence
2000-2010
2010-2021
Countries
Europe
Albania
Andorra
Austria
Belarus
Belgium
Bosnia and Herzegovina
Bulgaria
Croatia
Cyprus
Czechia
Denmark
Estonia
Finland
France
Germany
Greece
Hungary
Iceland
Ireland
Israel
Italy
Latvia
Lithuania
Luxembourg
Malta
Monaco
Montenegro
Netherlands
North Macedonia
Norway
Poland
Portugal
Republic of Moldova
Romania
Russian Federation
San Marino
Serbia
Slovakia
Slovenia
Spain
Sweden
Switzerland
Ukraine
United Kingdom
-2.5
0.0
2.5
5.0
Percentage change in age-standardized prevalence rate per 100 000
(B) DALYs
2000-2010
2010-2021
Countries
-4
-2
0
2
Percentage change in age-standardized DALY rate per 100 000

(C) Incidence

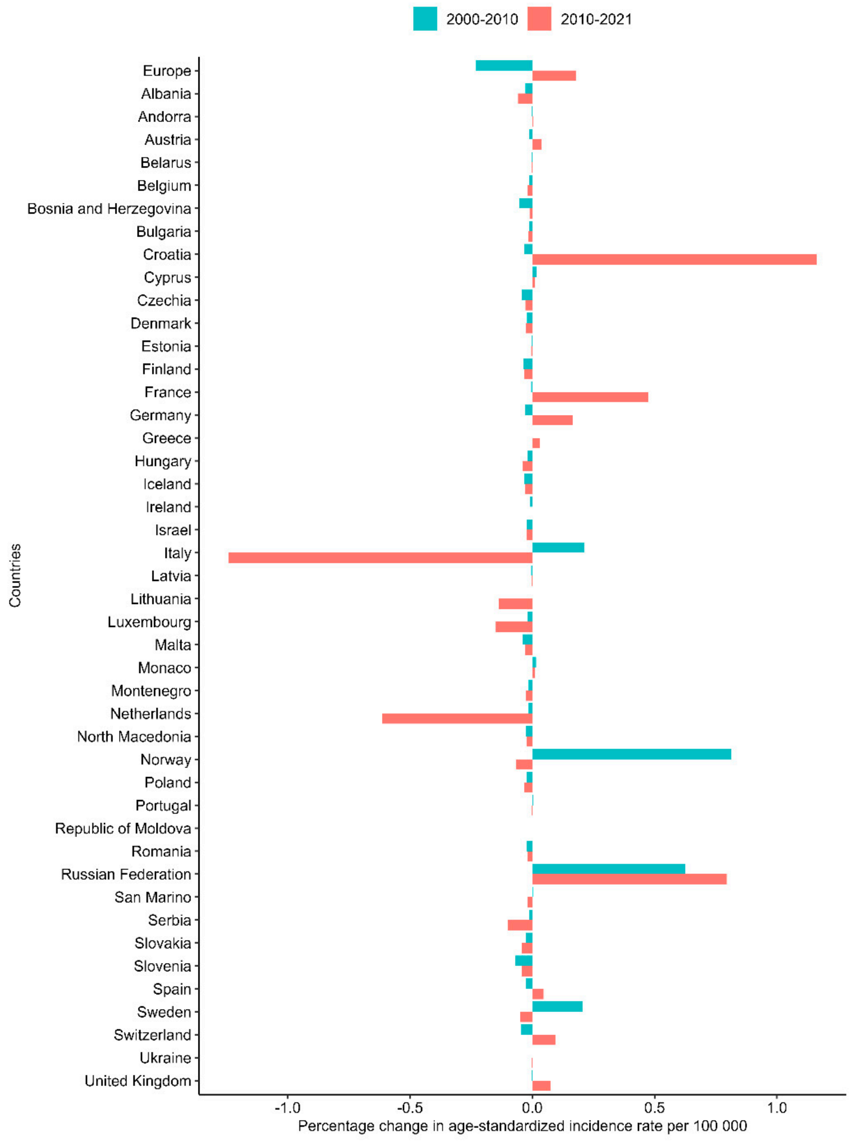

(D) YLDs

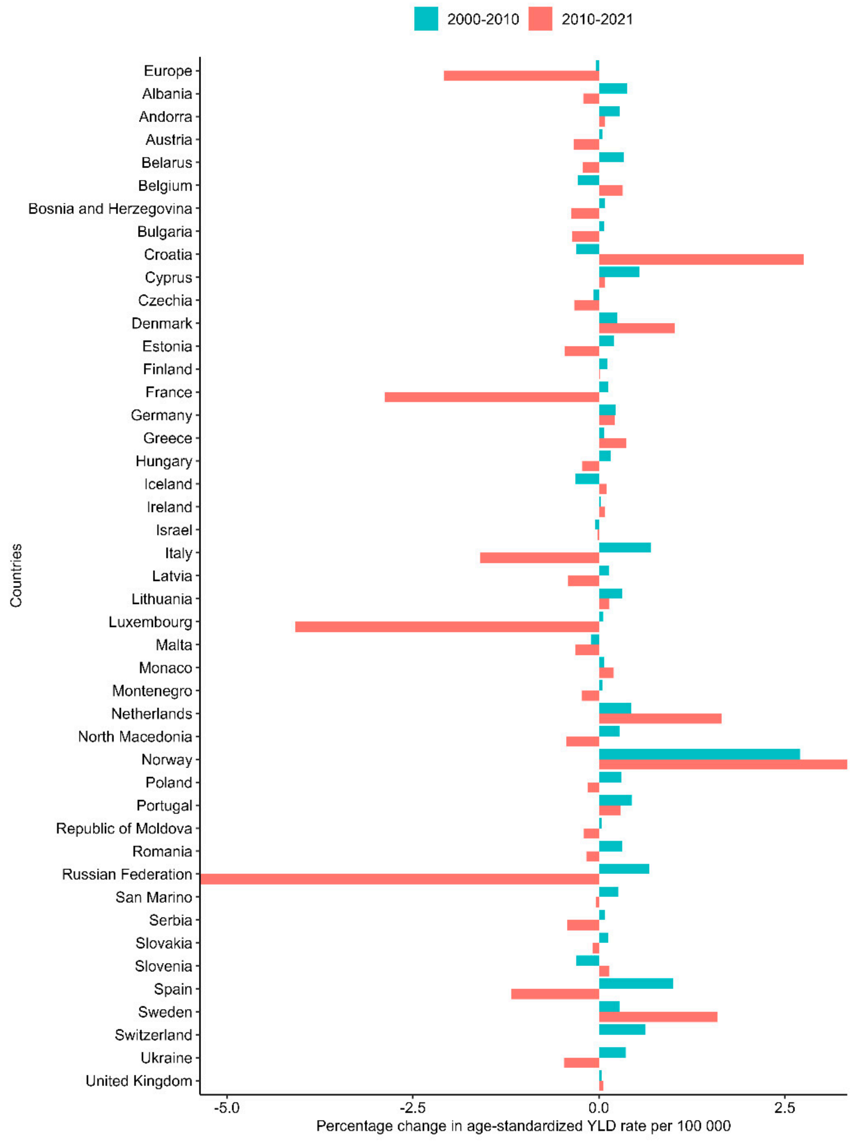

Figure S5c: Changes in age-standardized (A) prevalence, (B) DALY, (C) incidence, and (D) YLD rate for tension-type headache, by country, 2000-2010 and 2010-2021

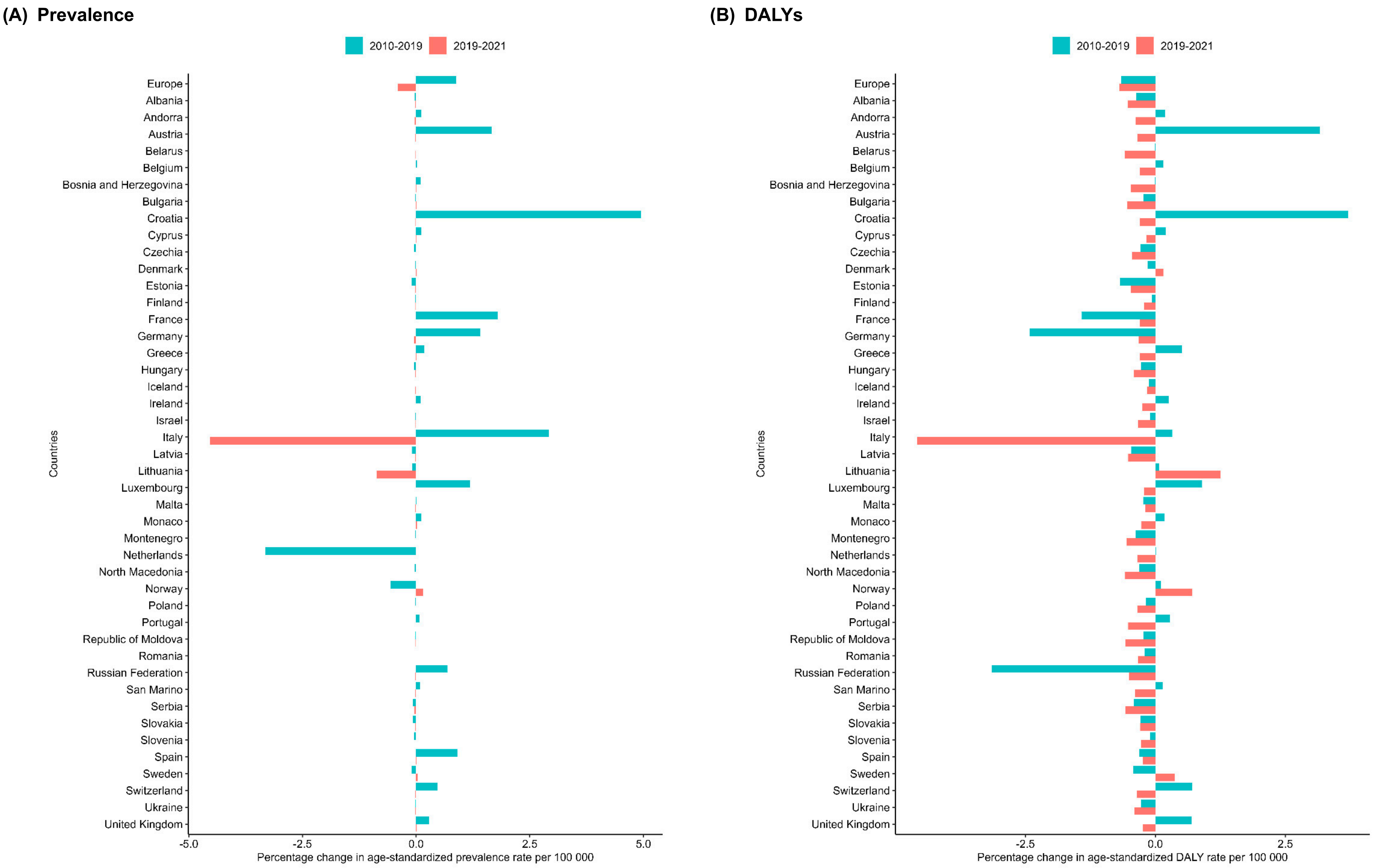

(A) Prevalence
2010-2019
2019-2021
Countries
Europe
Albania
Andorra
Austria
Belarus
Belgium
Bosnia and Herzegovina
Bulgaria
Croatia
Cyprus
Czechia
Denmark
Estonia
Finland
France
Germany
Greece
Hungary
Iceland
Ireland
Israel
Italy
Latvia
Lithuania
Luxembourg
Malta
Monaco
Montenegro
Netherlands
North Macedonia
Norway
Poland
Portugal
Republic of Moldova
Romania
Russian Federation
San Marino
Serbia
Slovakia
Slovenia
Spain
Sweden
Switzerland
Ukraine
United Kingdom
-5.0
-2.5
0.0
2.5
5.0
Percentage change in age-standardized prevalence rate per 100 000
(B) DALYs
2010-2019
2019-2021
Countries
Europe
Albania
Andorra
Austria
Belarus
Belgium
Bosnia and Herzegovina
Bulgaria
Croatia
Cyprus
Czechia
Denmark
Estonia
Finland
France
Germany
Greece
Hungary
Iceland
Ireland
Israel
Italy
Latvia
Lithuania
Luxembourg
Malta
Monaco
Montenegro
Netherlands
North Macedonia
Norway
Poland
Portugal
Republic of Moldova
Romania
Russian Federation
San Marino
Serbia
Slovakia
Slovenia
Spain
Sweden
Switzerland
Ukraine
United Kingdom
-2.5
0.0
2.5
Percentage change in age-standardized DALY rate per 100 000

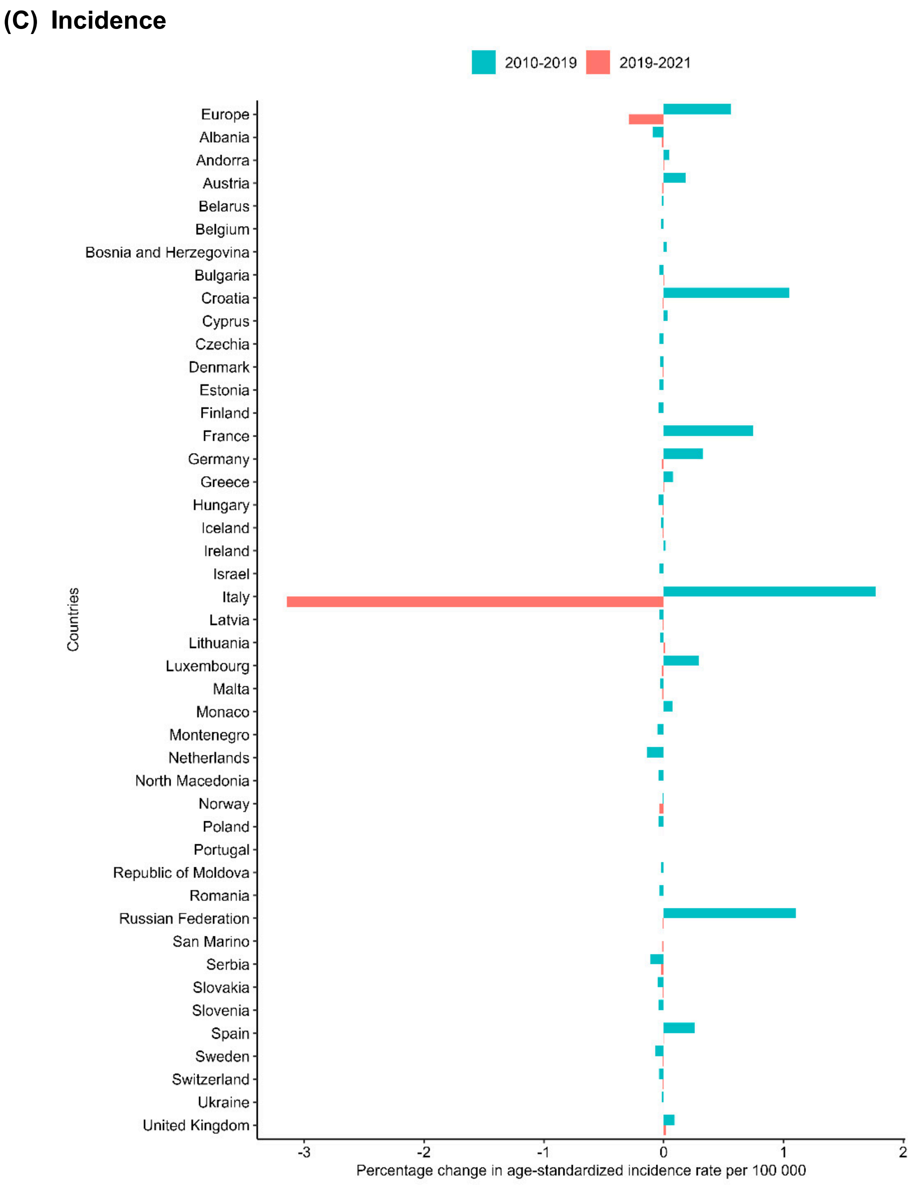

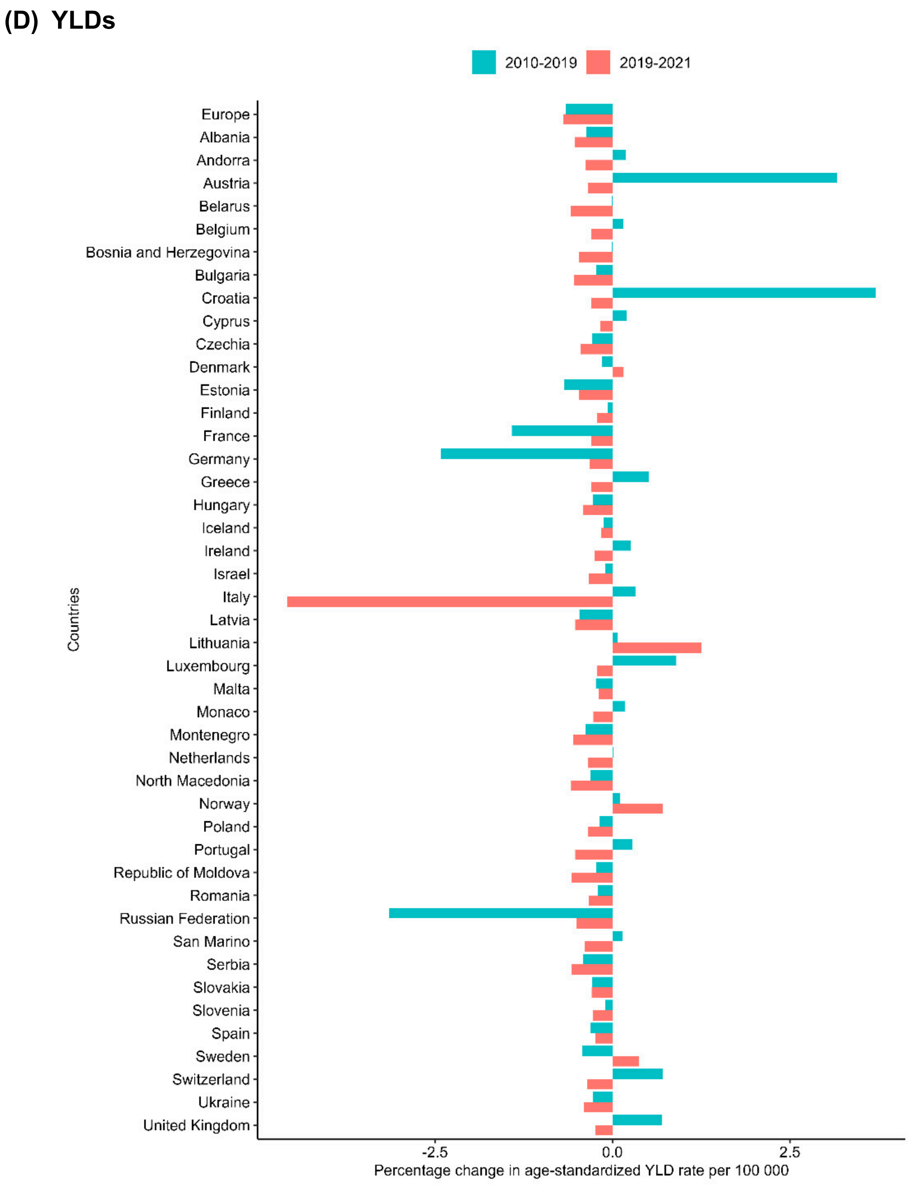

Figure S6a: Changes in age-standardized (A) prevalence, (B) DALY, (C) incidence, and (D) YLD rate for headache disorders, by country, 2010-2019 and 2019-2021

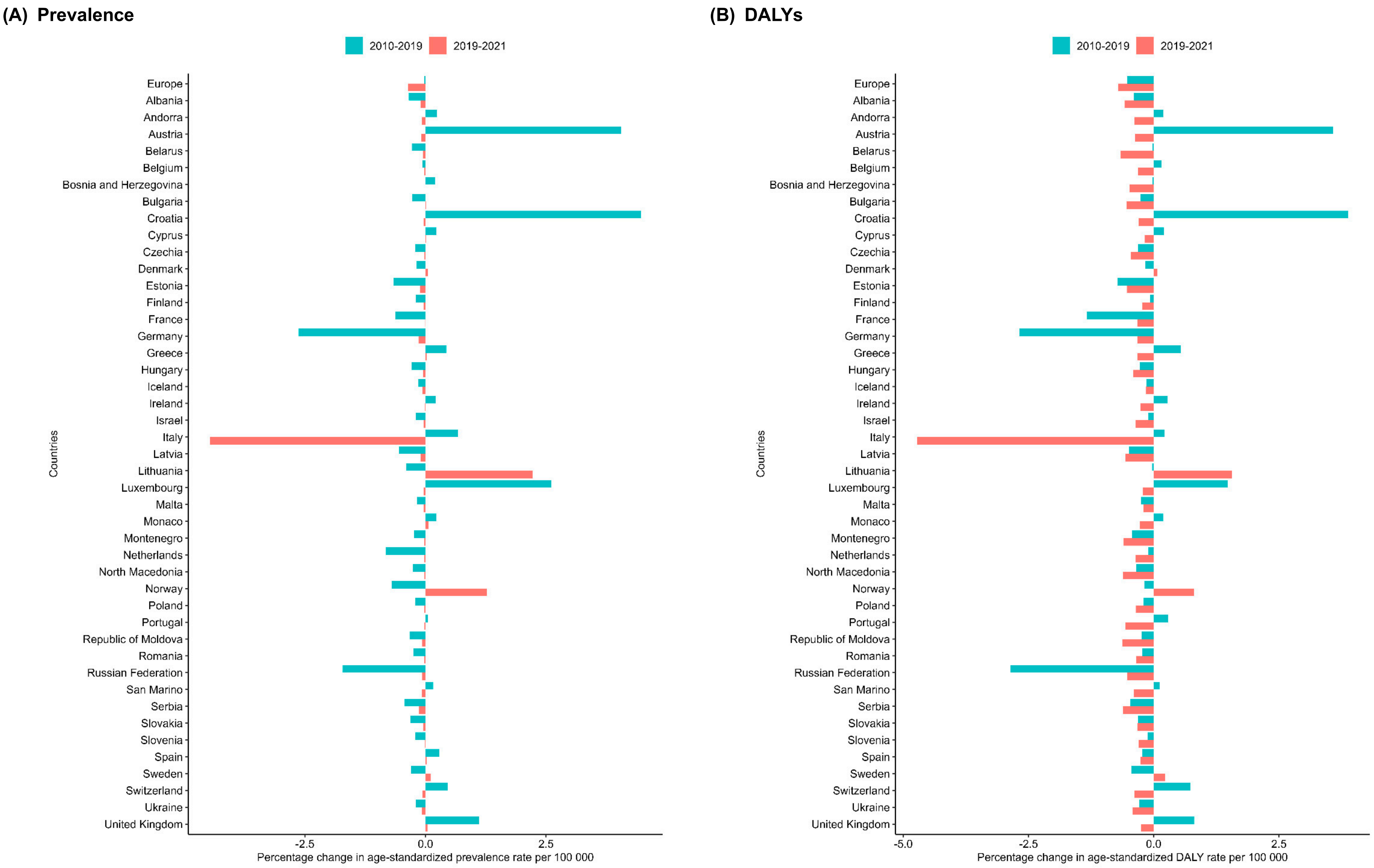
(A) Prevalence
2010-2019
2019-2021
Europe
Albania
Andorra
Austria
Belarus
Belgium
Bosnia and Herzegovina
Bulgaria
Croatia
Cyprus
Czechia
Denmark
Estonia
Finland
France
Germany
Greece
Hungary
Iceland
Ireland
Israel
Italy
Latvia
Lithuania
Luxembourg
Malta
Monaco
Montenegro
Netherlands
North Macedonia
Norway
Poland
Portugal
Republic of Moldova
Romania
Russian Federation
San Marino
Serbia
Slovakia
Slovenia
Spain
Sweden
Switzerland
Ukraine
United Kingdom
Countries
-2.5
0.0
2.5
Percentage change in age-standardized prevalence rate per 100 000
(B) DALYs
2010-2019
2019-2021
Countries
-5.0
-2.5
0.0
2.5
Percentage change in age-standardized DALY rate per 100 000

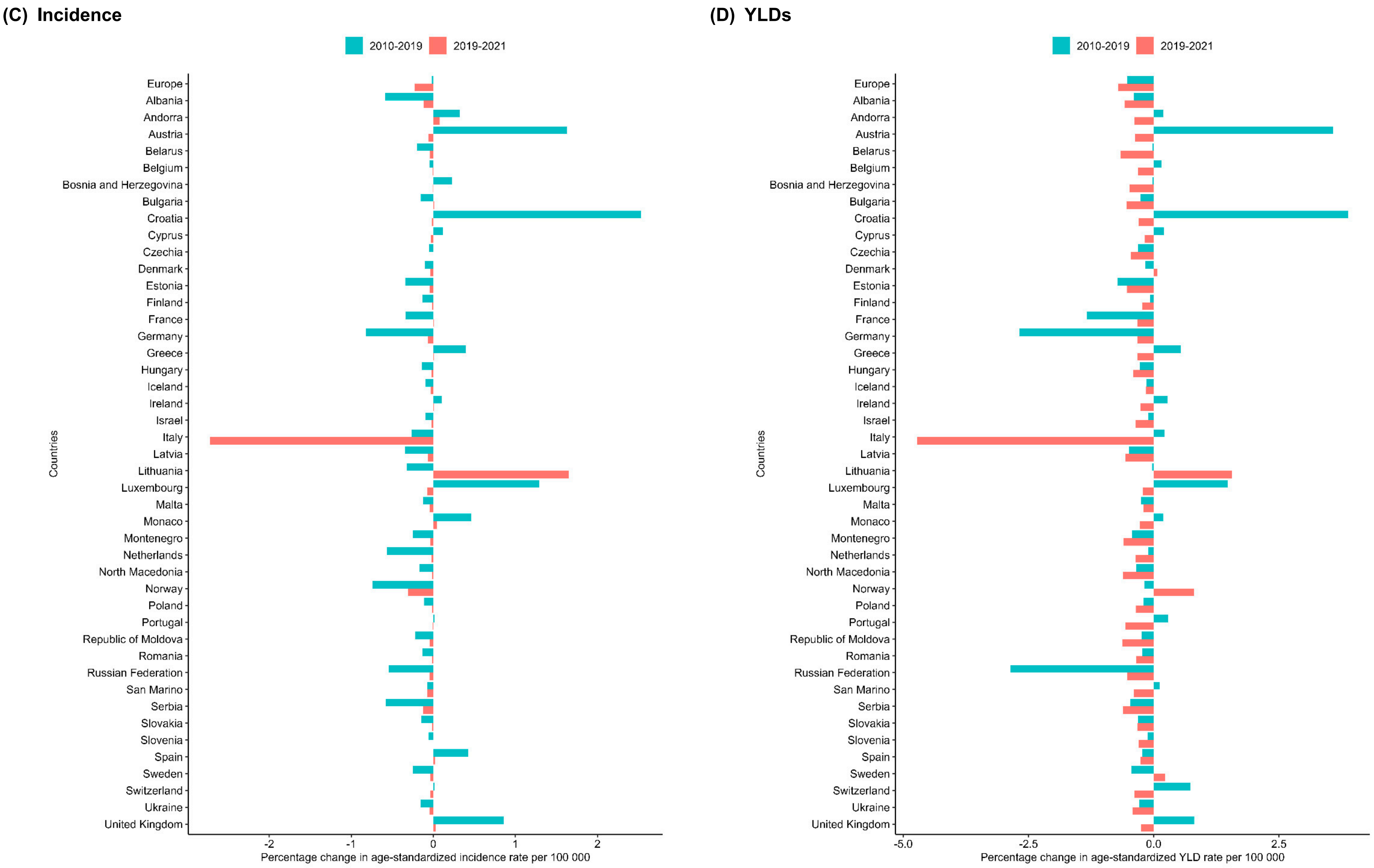

Figure S6b: Changes in age-standardized (A) prevalence, (B) DALY, (C) incidence, and (D) YLD rate for migraine, by country, 2010-2019 and 2019-2021

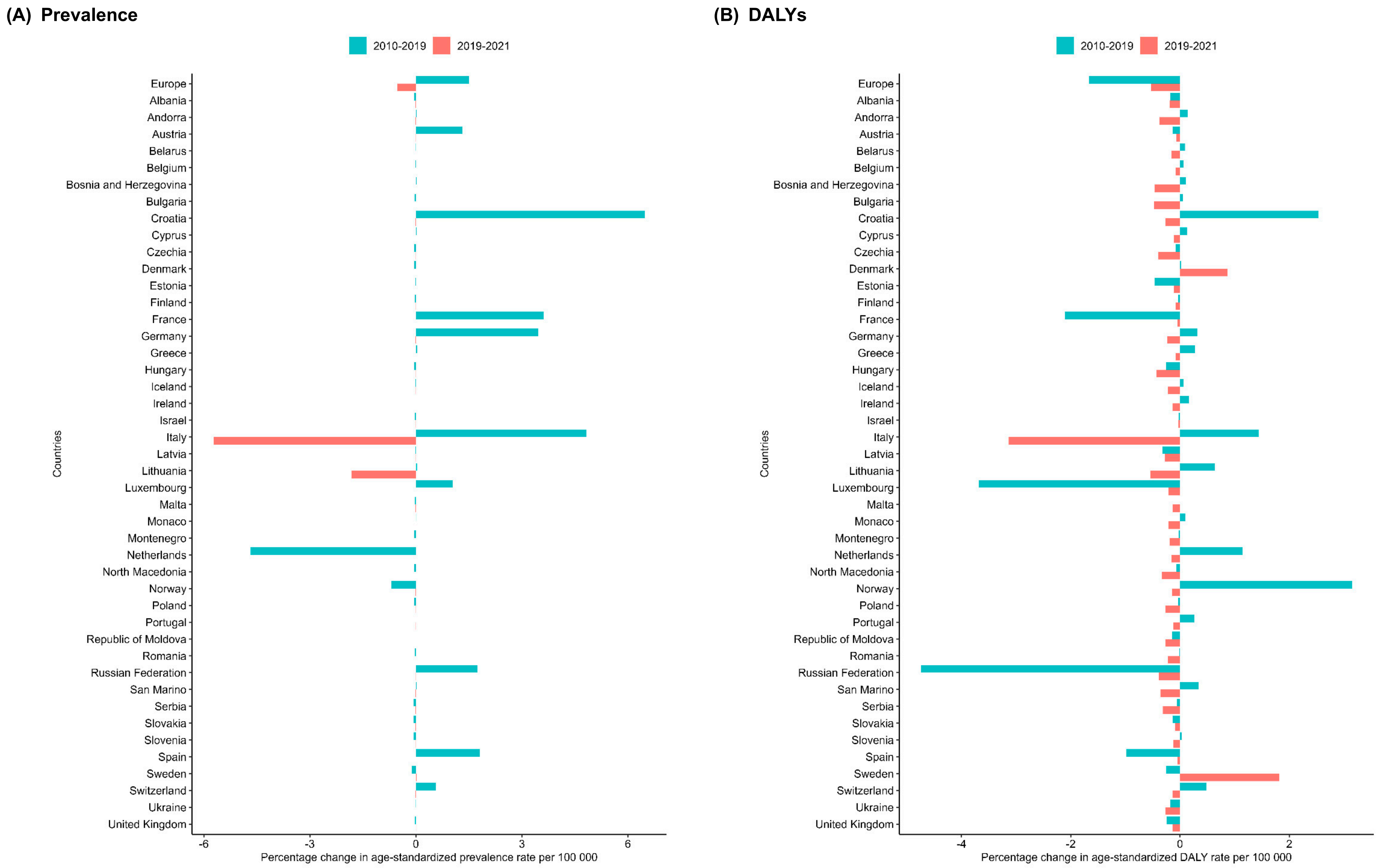

(A) Prevalence
2010-2019
2019-2021
Europe
Albania
Andorra
Austria
Belarus
Belgium
Bosnia and Herzegovina
Bulgaria
Croatia
Cyprus
Czechia
Denmark
Estonia
Finland
France
Germany
Greece
Hungary
Iceland
Ireland
Israel
Italy
Latvia
Lithuania
Luxembourg
Malta
Monaco
Montenegro
Netherlands
North Macedonia
Norway
Poland
Portugal
Republic of Moldova
Romania
Russian Federation
San Marino
Serbia
Slovakia
Slovenia
Spain
Sweden
Switzerland
Ukraine
United Kingdom
Countries
-6
-3
0
3
6
Percentage change in age-standardized prevalence rate per 100 000
(B) DALYs
2010-2019
2019-2021
Countries
-4
-2
0
2
Percentage change in age-standardized DALY rate per 100 000

(C) Incidence

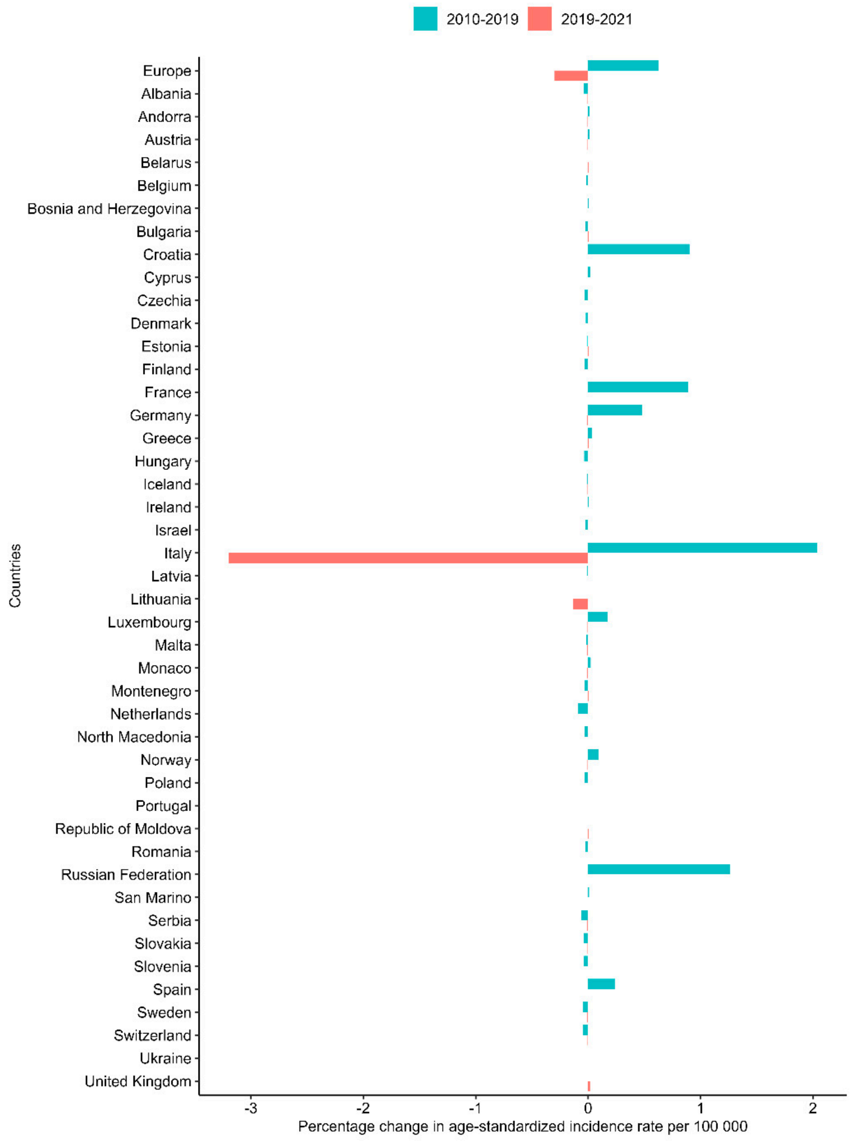

(D) YLDs

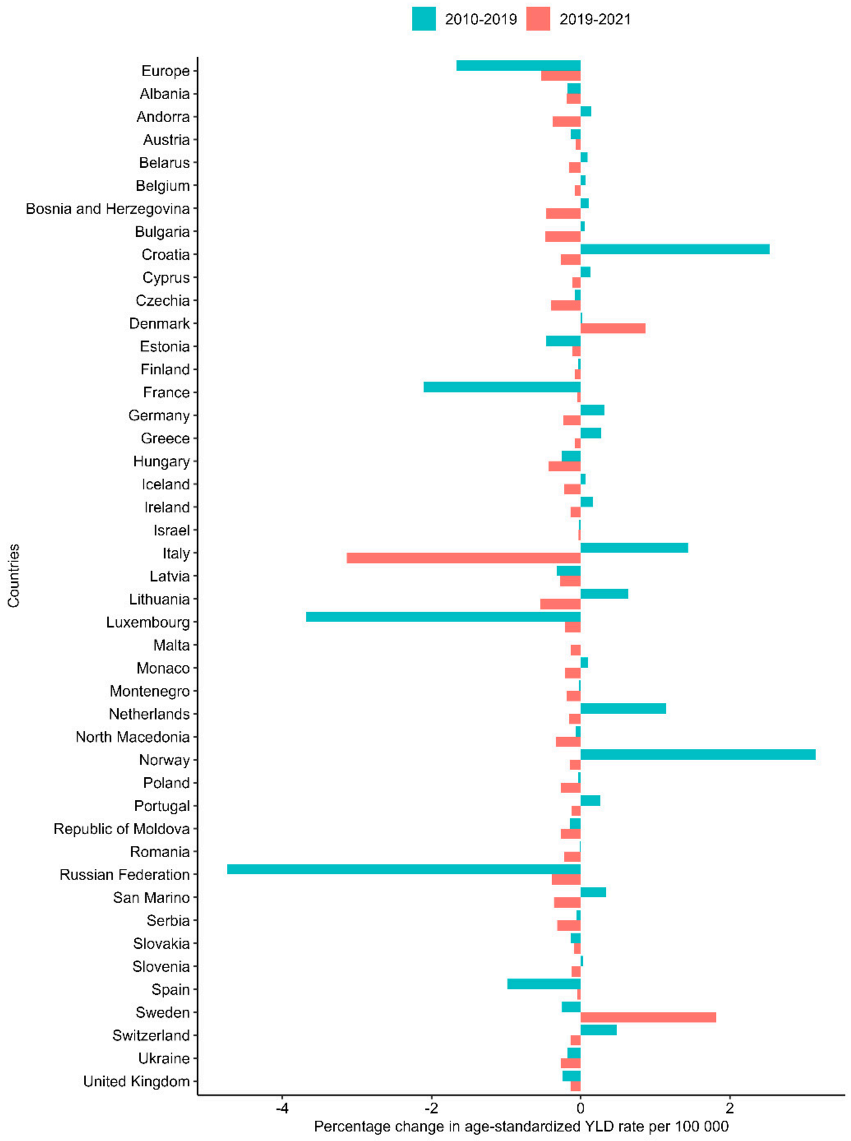

Figure S6c: Changes in age-standardized (A) prevalence, (B) DALY, (C) incidence, and (D) YLD rate for tension-type headache, by country, 2010-2019 and 2019-2021

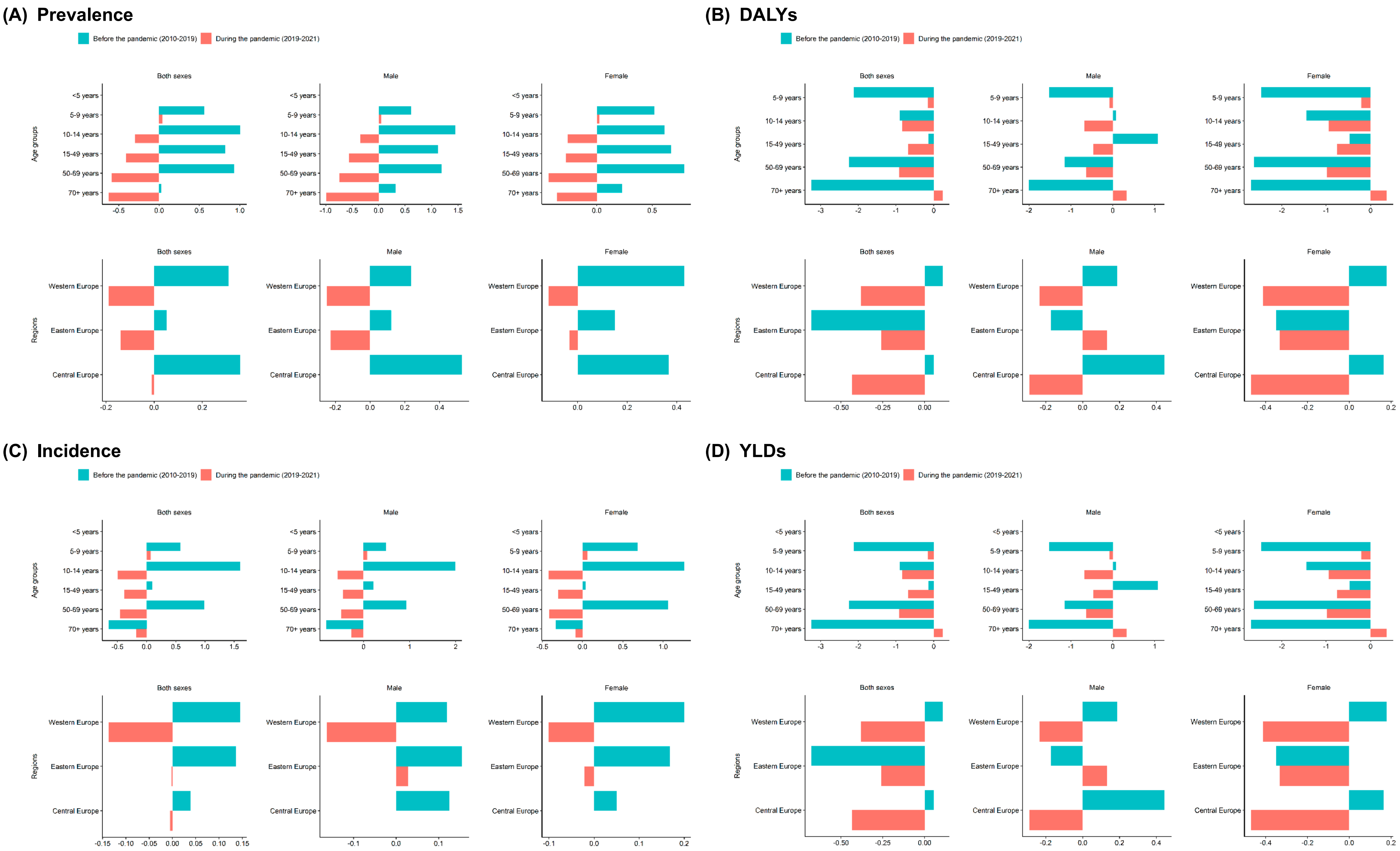

Figure S7a: Annual changes in (A) prevalence, (B) DALY, (C) incidence, and (D) YLD rates of headache disorders comparing pre-pandemic (2010-2019) to pandemic periods (2019-2021), stratified by age group and by region

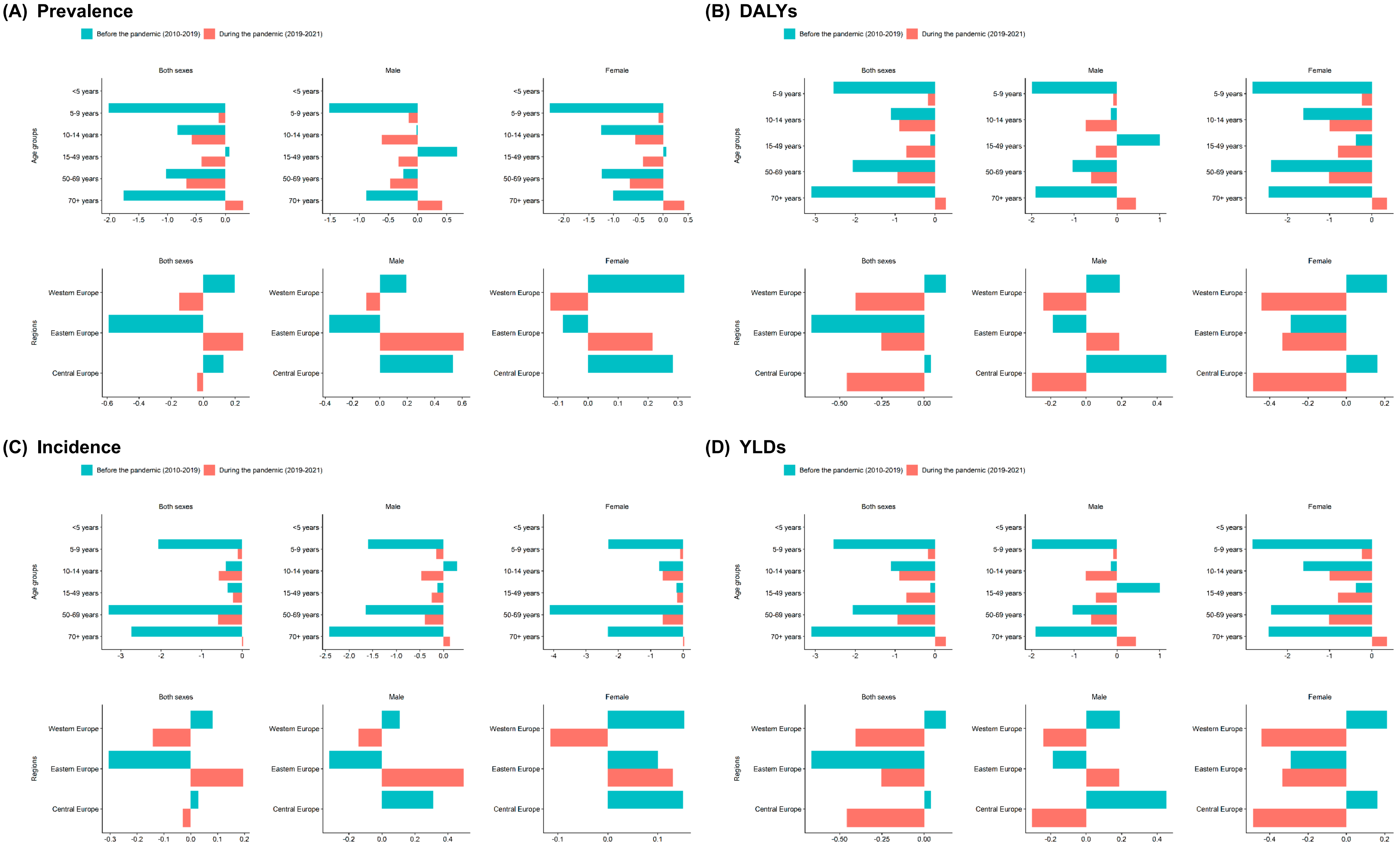

Figure S7b: Annual changes in (A) prevalence, (B) DALY, (C) incidence, and (D) YLD rates of migraine comparing pre-pandemic (2010-2019) to pandemic periods (2019-2021), stratified by age group and by region

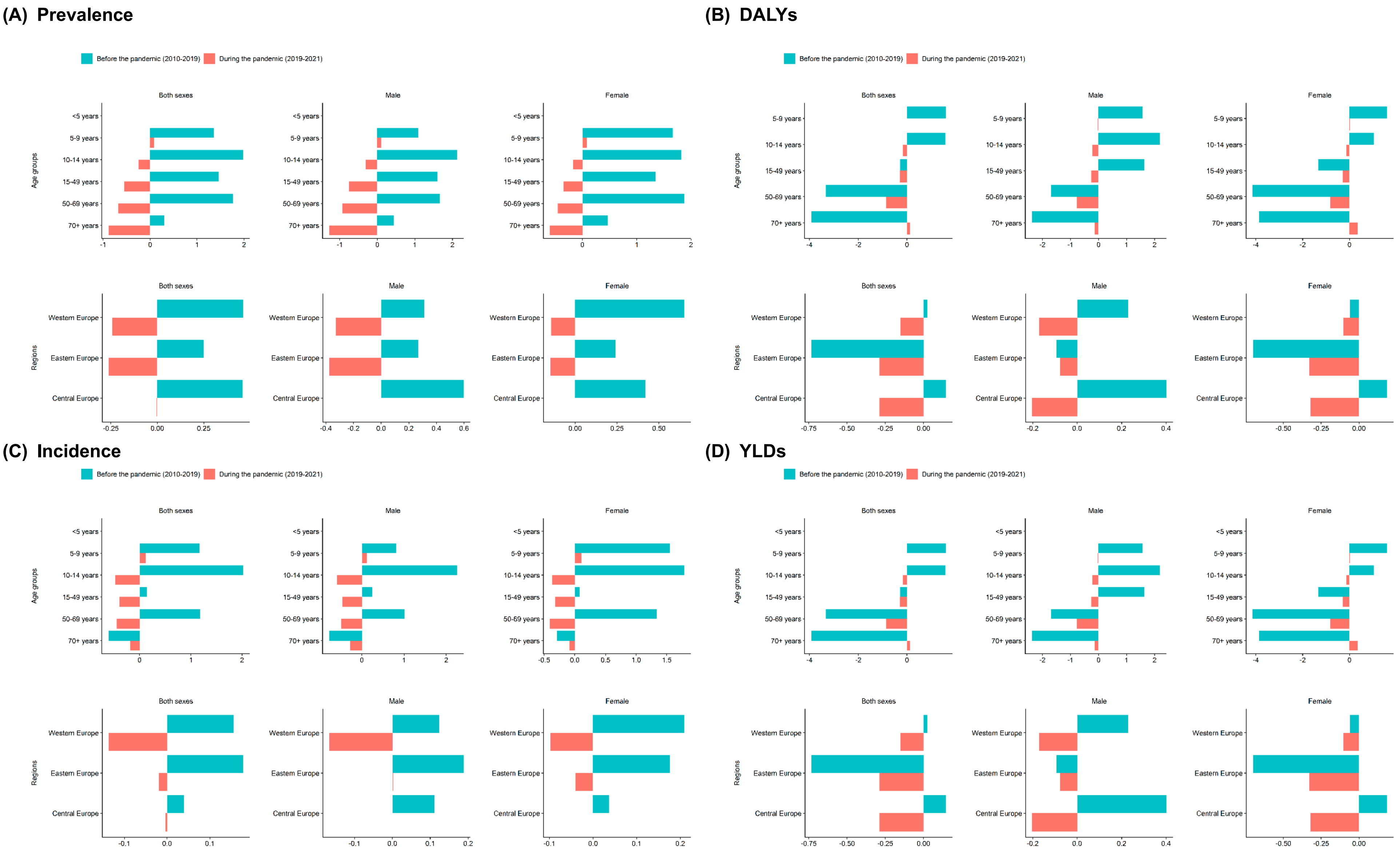

Figure S7c: Annual changes in (A) prevalence, (B) DALY, (C) incidence, and (D) YLD rates of tension-type headache comparing pre-pandemic (2010-2019) to pandemic periods (2019-2021), stratified by age group and by region

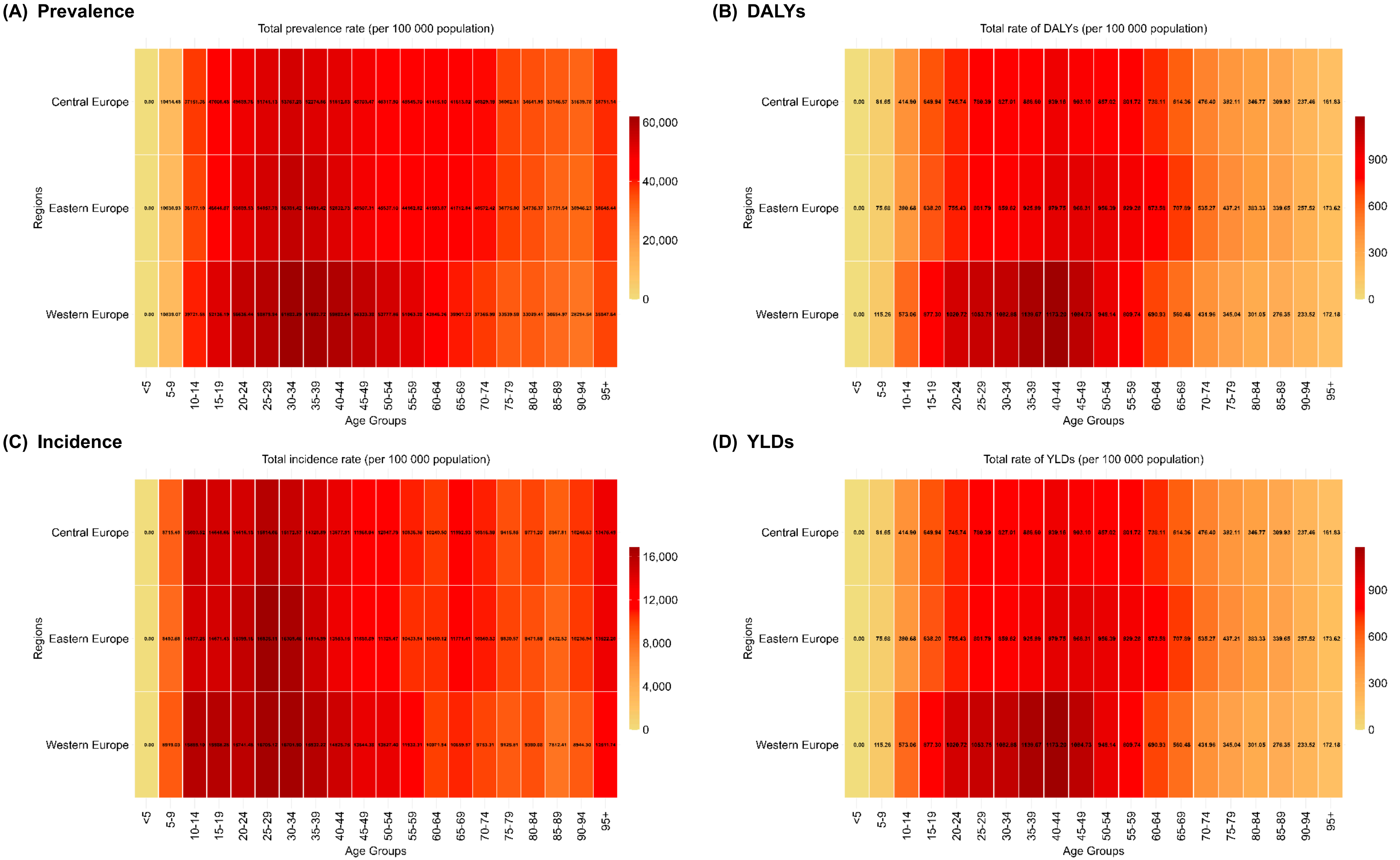

Figure S8a: Total (A) prevalence, (B) DALY, (C) incidence, and (D) YLD rates of headache disorders for both sexes, by age group and region, 2021

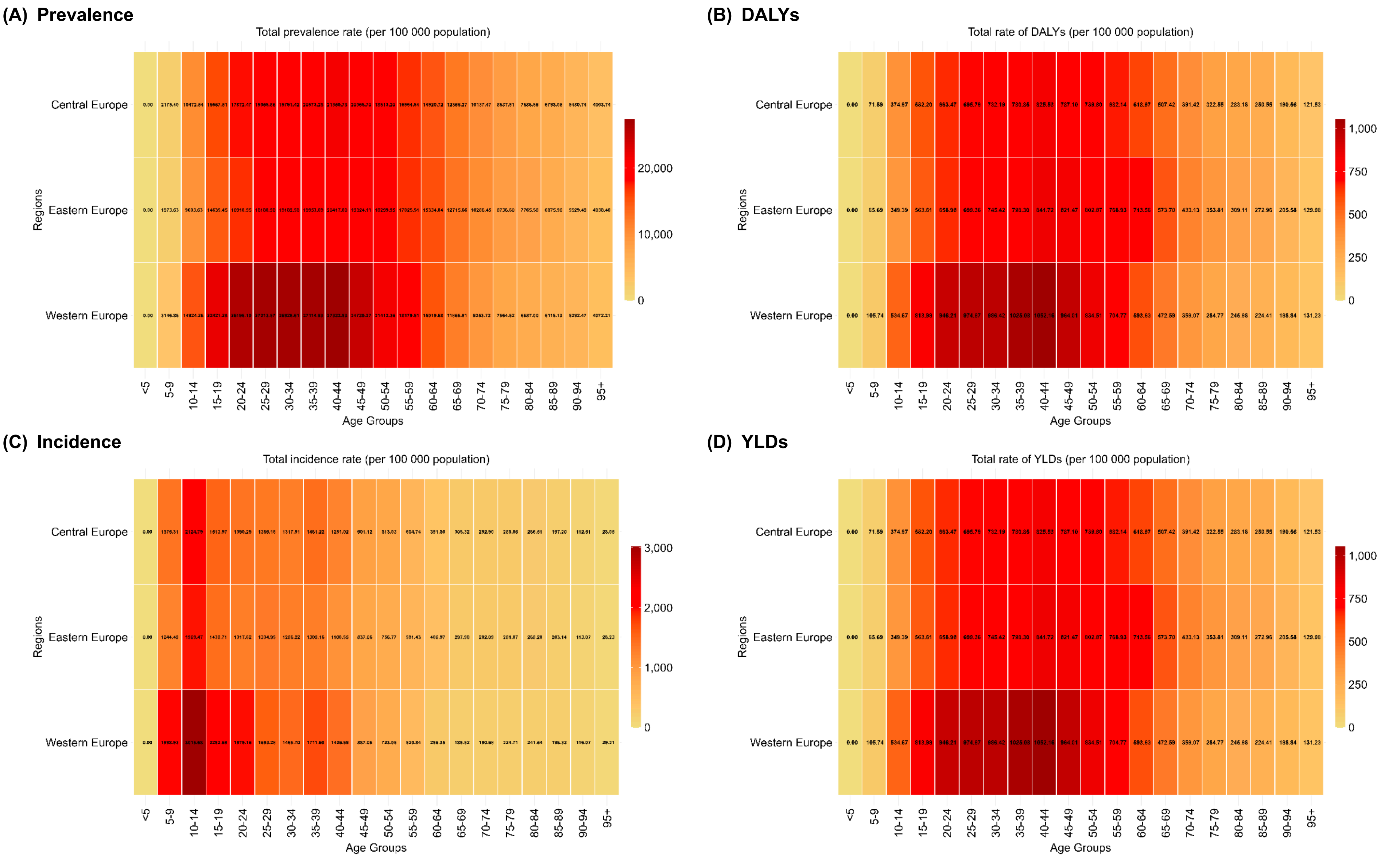

Figure S8b: Total (A) prevalence, (B) DALY, (C) incidence, and (D) YLD rates of migraine for both sexes, by age group and region, 2021

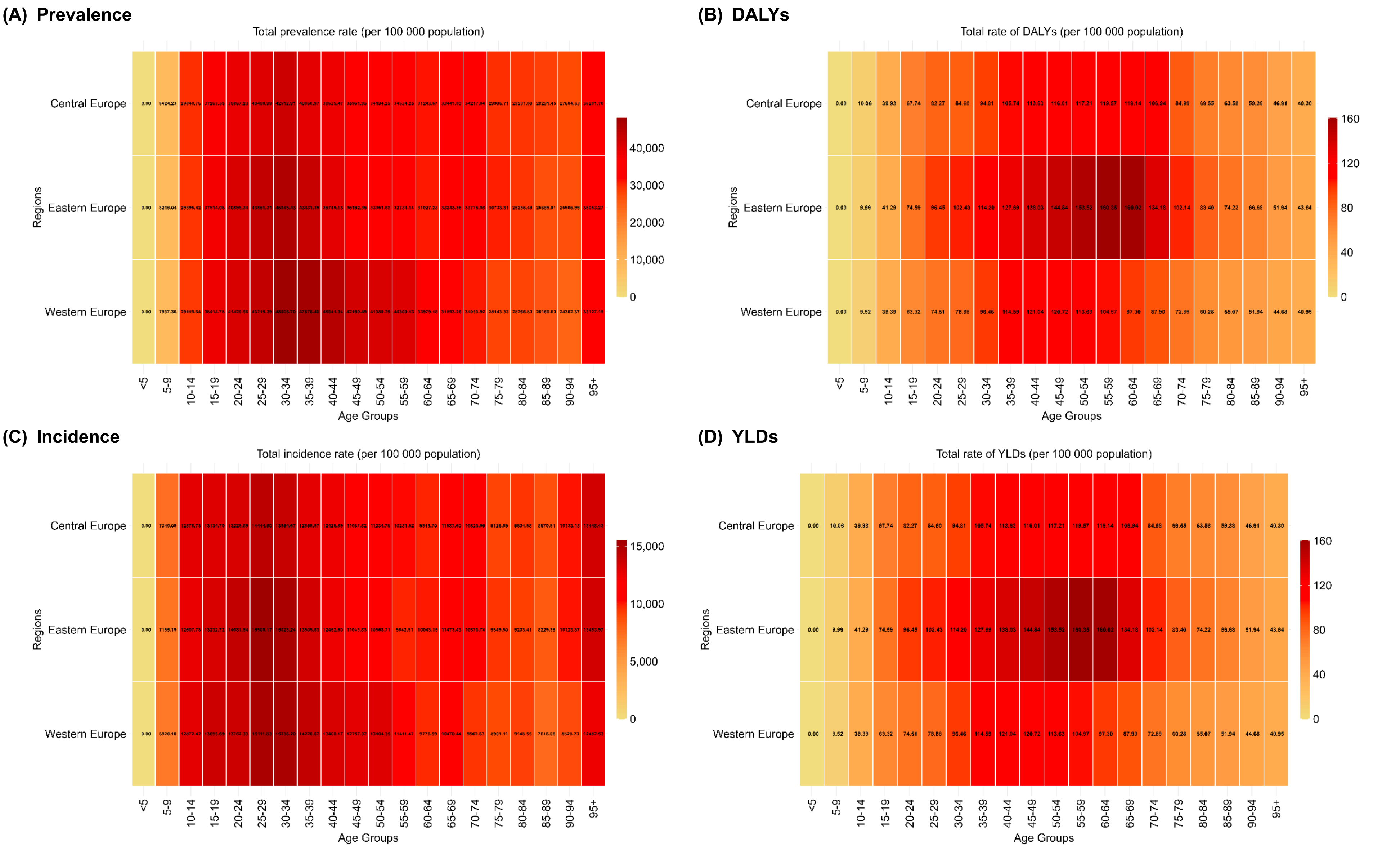

Figure S8c: Total (A) prevalence, (B) DALY, (C) incidence, and (D) YLD rates of tension-type headache for both sexes, by age group and region, 2021

(A) Prevalence **(B) DALYs**

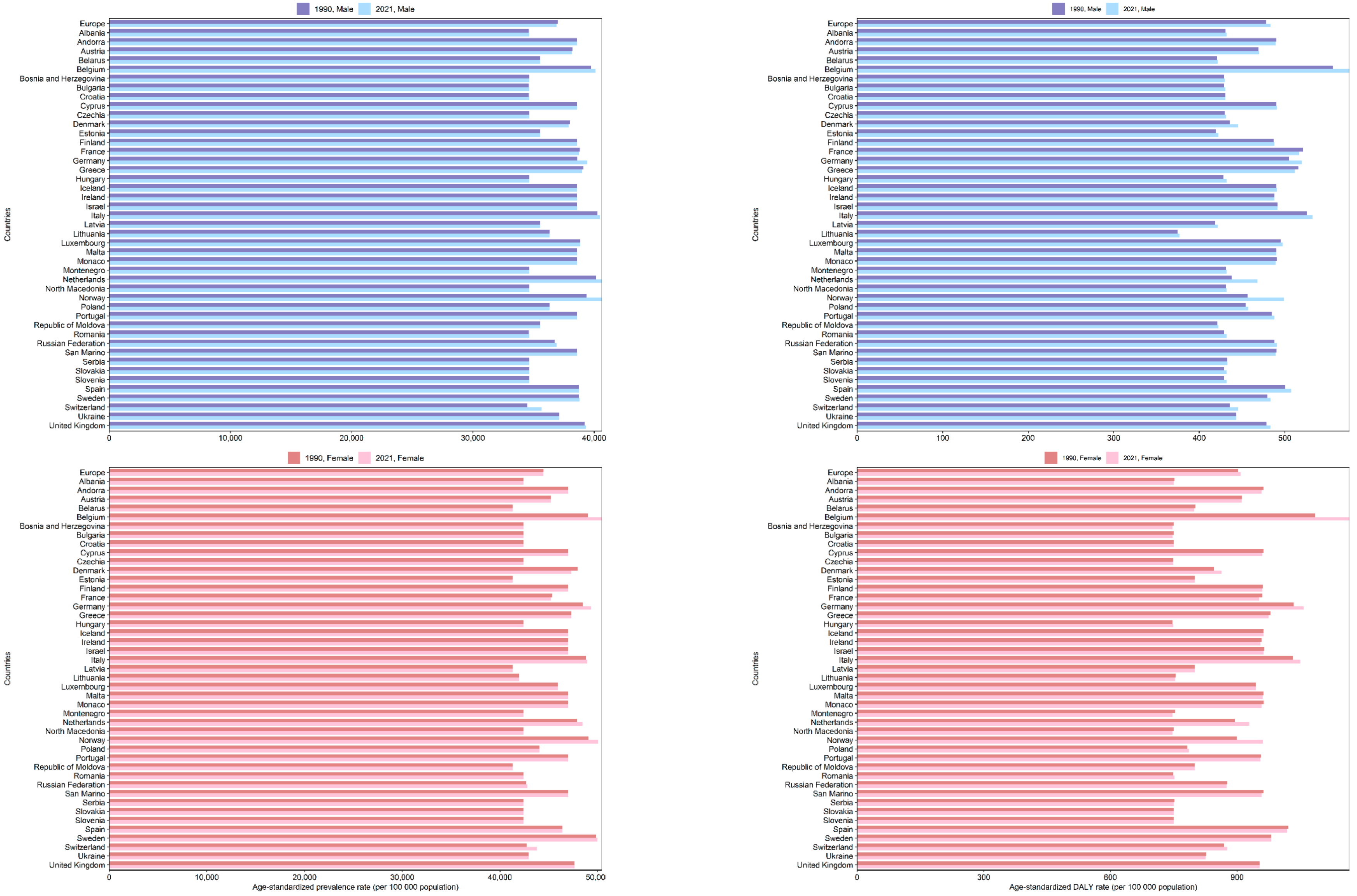

(C) Incidence

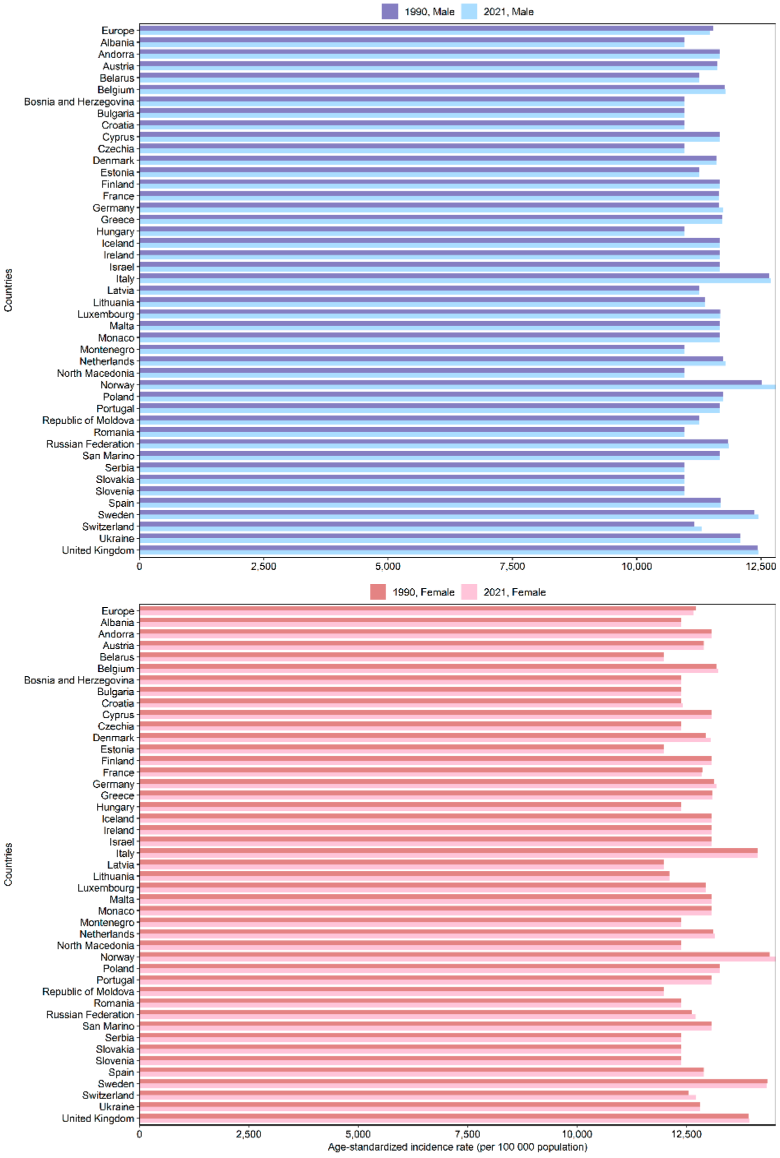

(D) YLDs

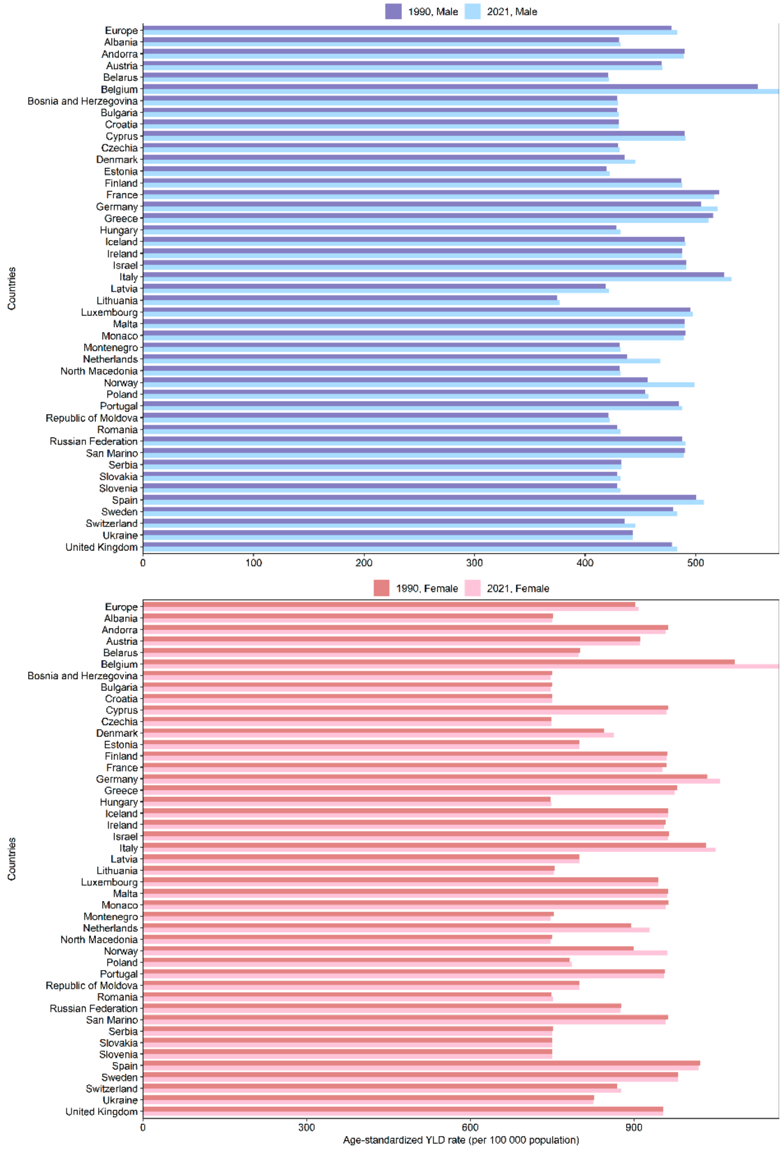

Figure S9a: Age-standardized rates of (A) prevalence, (B) DALY, (C) incidence, and (D) YLD of headache disorders, by country and sex, 1990–2021

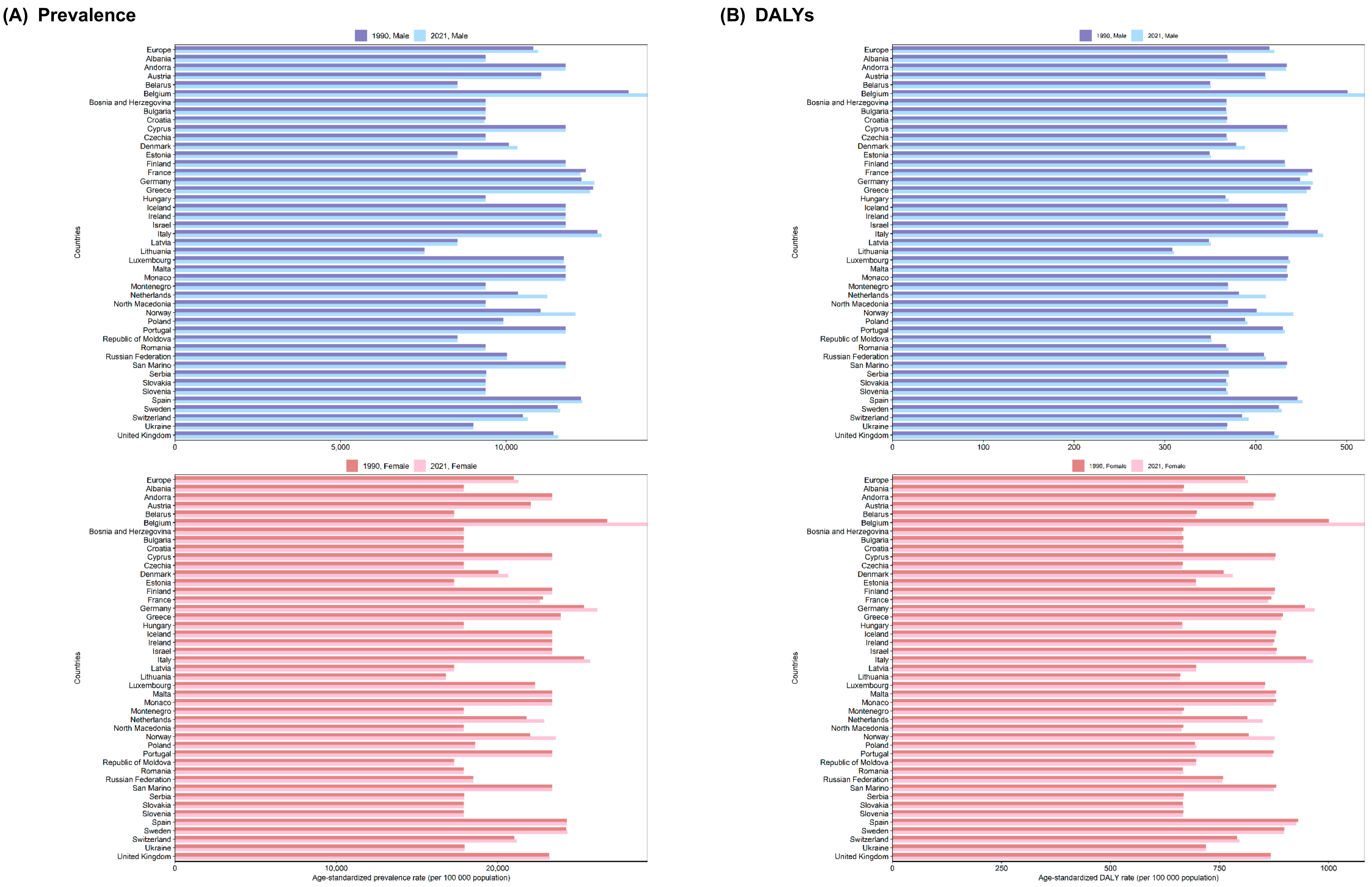

(A) Prevalence
(B) DALYs
1990, Male
2021, Male
1990, Female
2021, Female
Countries
Europe
Albania
Andorra
Austria
Belarus
Belgium
Bosnia and Herzegovina
Bulgaria
Croatia
Cyprus
Czechia
Denmark
Estonia
Finland
France
Germany
Greece
Hungary
Iceland
Ireland
Israel
Italy
Latvia
Lithuania
Luxembourg
Malta
Monaco
Montenegro
Netherlands
North Macedonia
Norway
Poland
Portugal
Republic of Moldova
Romania
Russian Federation
San Marino
Serbia
Slovakia
Slovenia
Spain
Sweden
Switzerland
Ukraine
United Kingdom
0
5,000
10,000
10,000
20,000
100
200
300
400
500
250
500
750
1000
Age-standardized prevalence rate (per 100 000 population)
Age-standardized DALY rate (per 100 000 population)

(C) Incidence

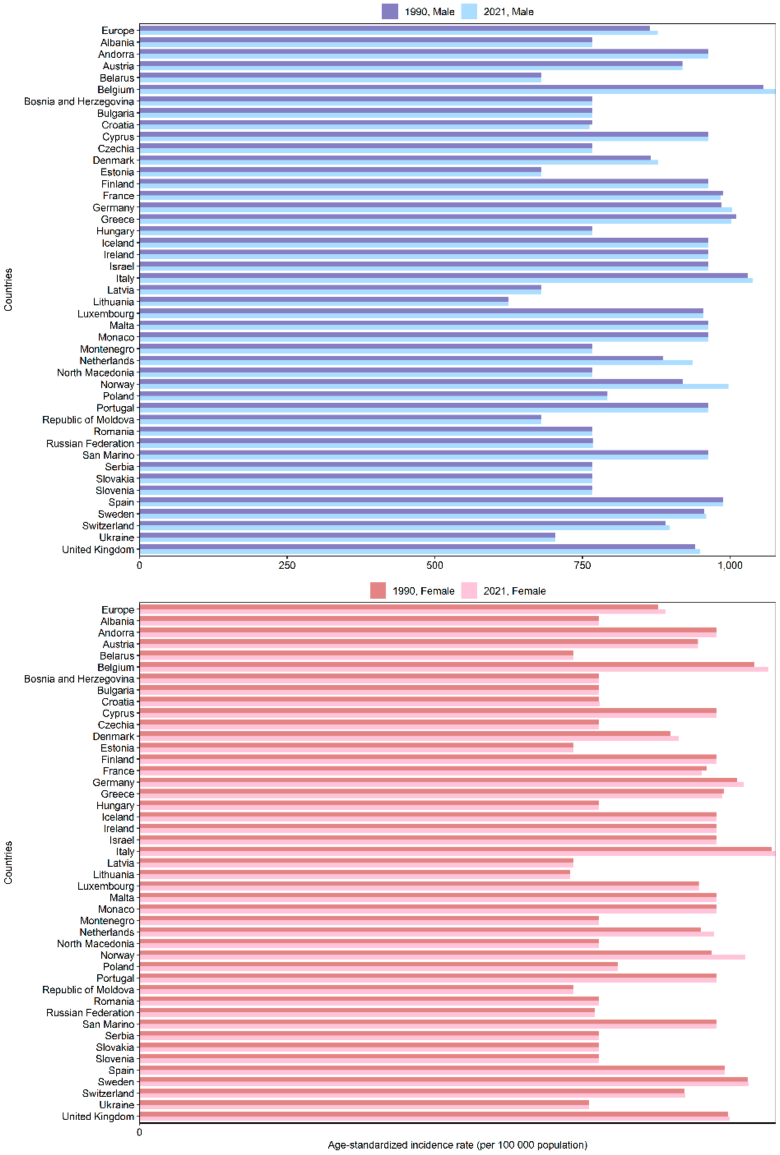

(D) YLDs

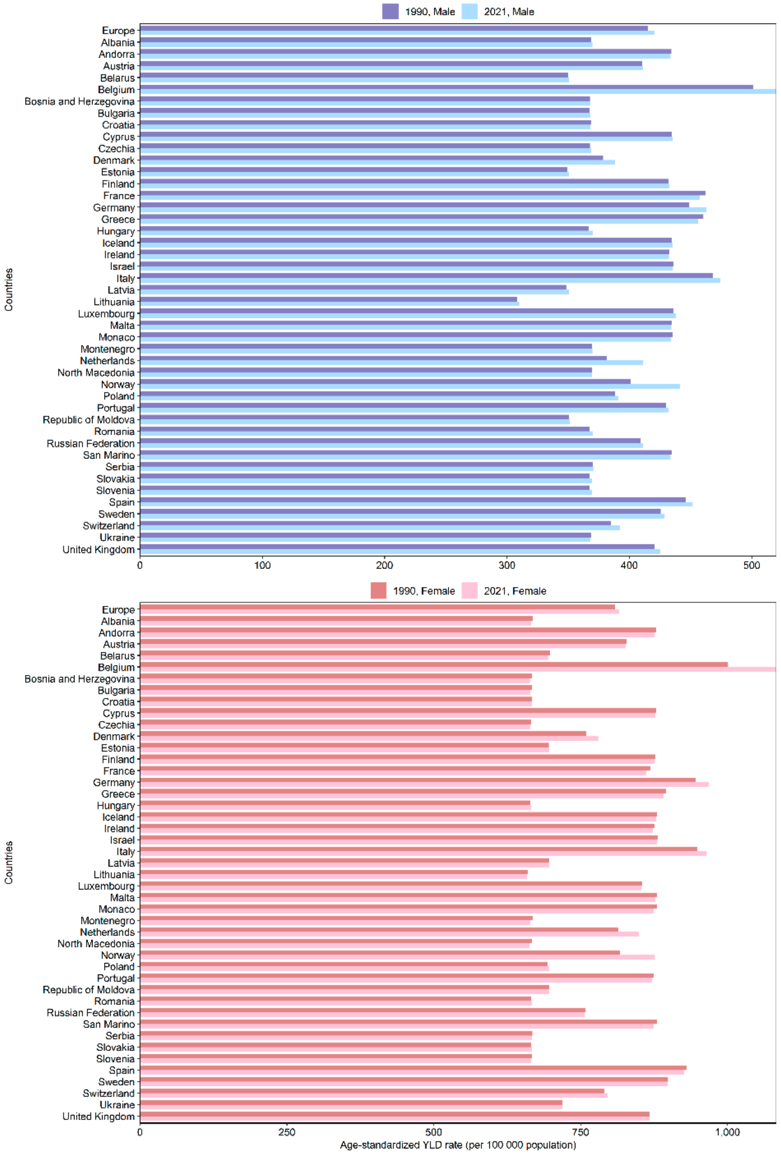

Figure S9b: Age-standardized rates of (A) prevalence, (B) DALY, (C) incidence, and (D) YLD of migraine, by country and sex, 1990–2021

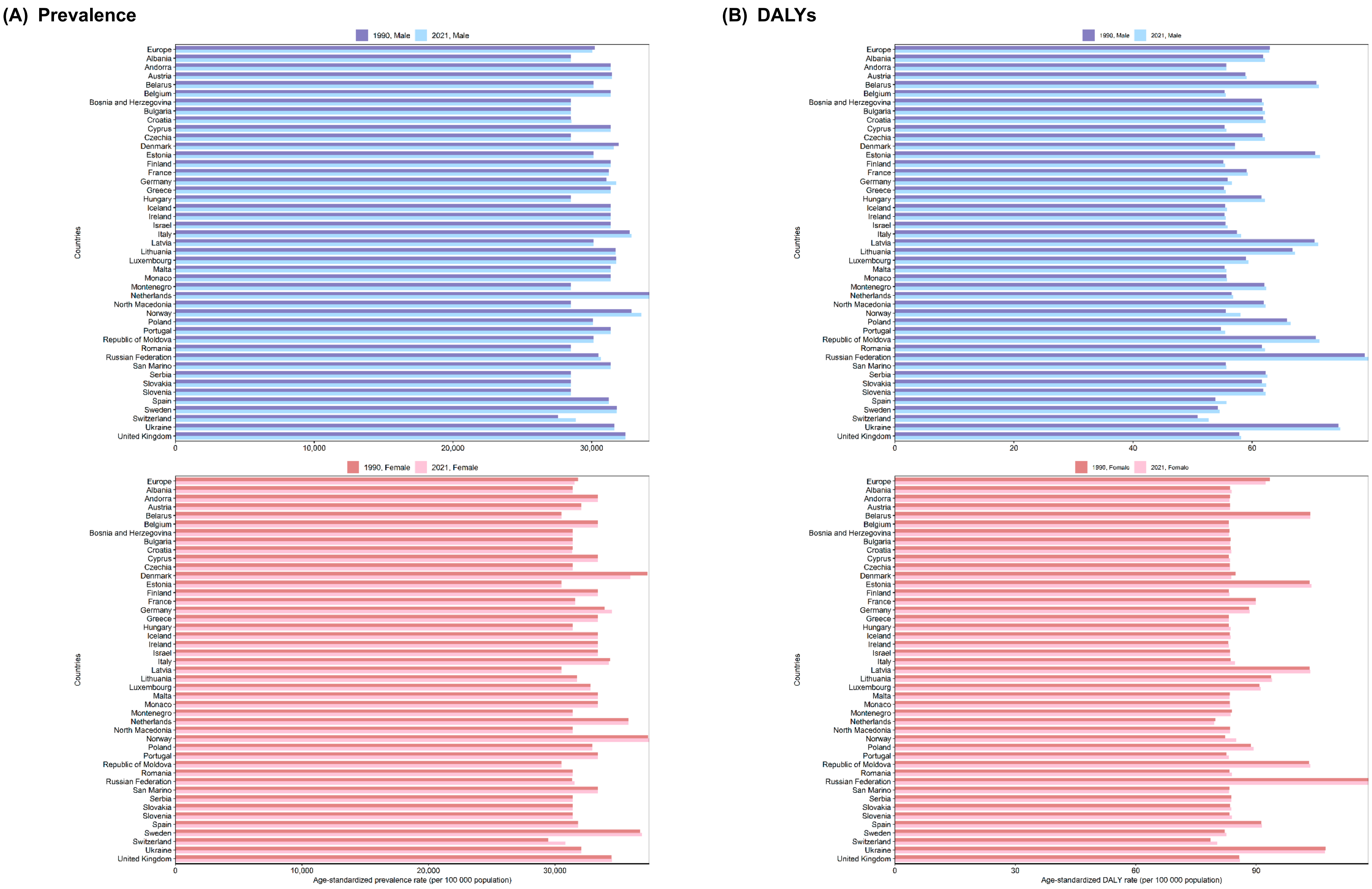
(A) Prevalence
(B) DALYs
1990, Male
2021, Male
1990, Female
2021, Female
Countries
Europe
Albania
Andorra
Austria
Belarus
Belgium
Bosnia and Herzegovina
Bulgaria
Croatia
Cyprus
Czechia
Denmark
Estonia
Finland
France
Germany
Greece
Hungary
Iceland
Ireland
Israel
Italy
Latvia
Lithuania
Luxembourg
Malta
Monaco
Montenegro
Netherlands
North Macedonia
Norway
Poland
Portugal
Republic of Moldova
Romania
Russian Federation
San Marino
Serbia
Slovakia
Slovenia
Spain
Sweden
Switzerland
Ukraine
United Kingdom
0
10,000
20,000
30,000
20
40
60
90
Age-standardized prevalence rate (per 100 000 population)
Age-standardized DALY rate (per 100 000 population)

(C) Incidence

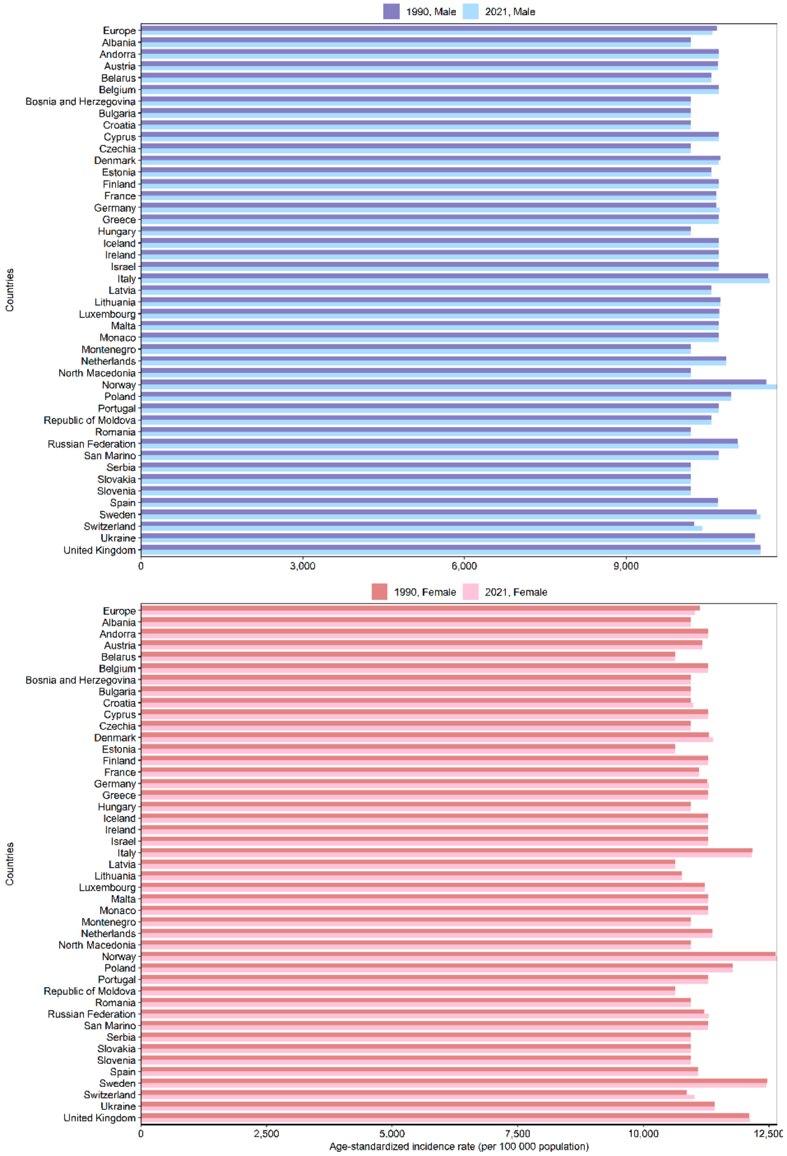

(D) YLDs

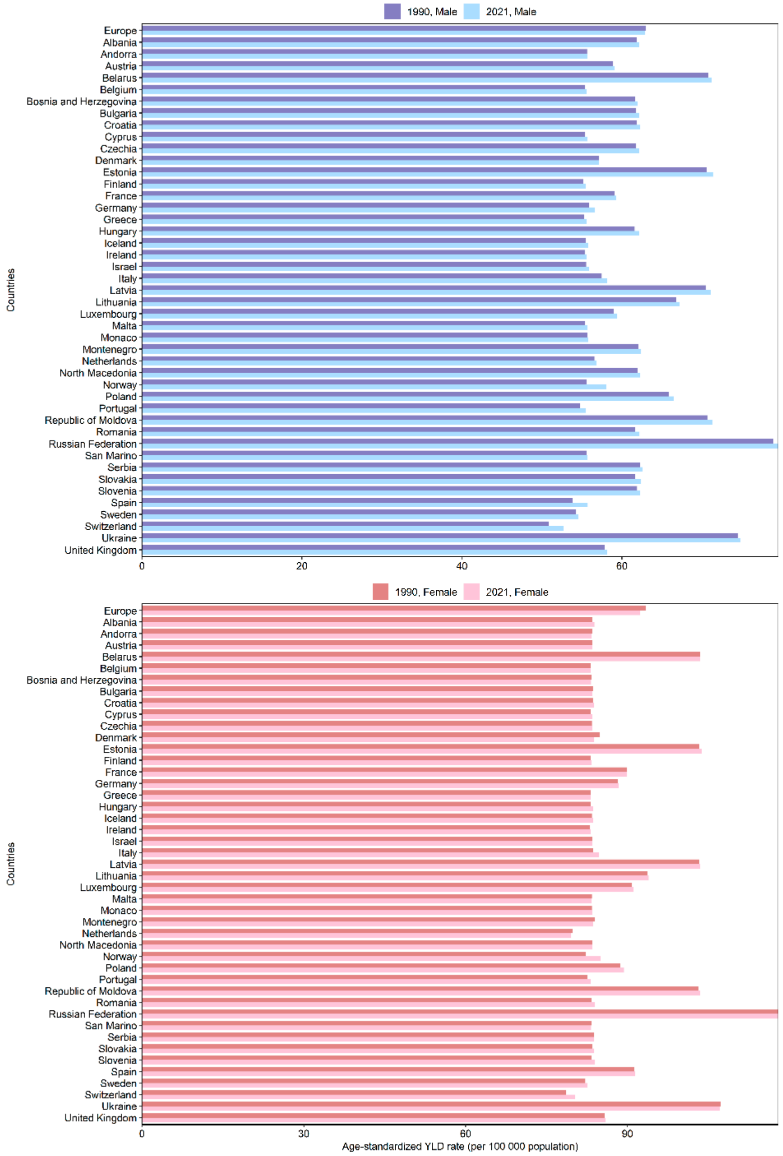

Figure S9c: Age-standardized rates of (A) prevalence, (B) DALY, (C) incidence, and (D) YLD of tension-type headache, by country and sex, 1990–2021

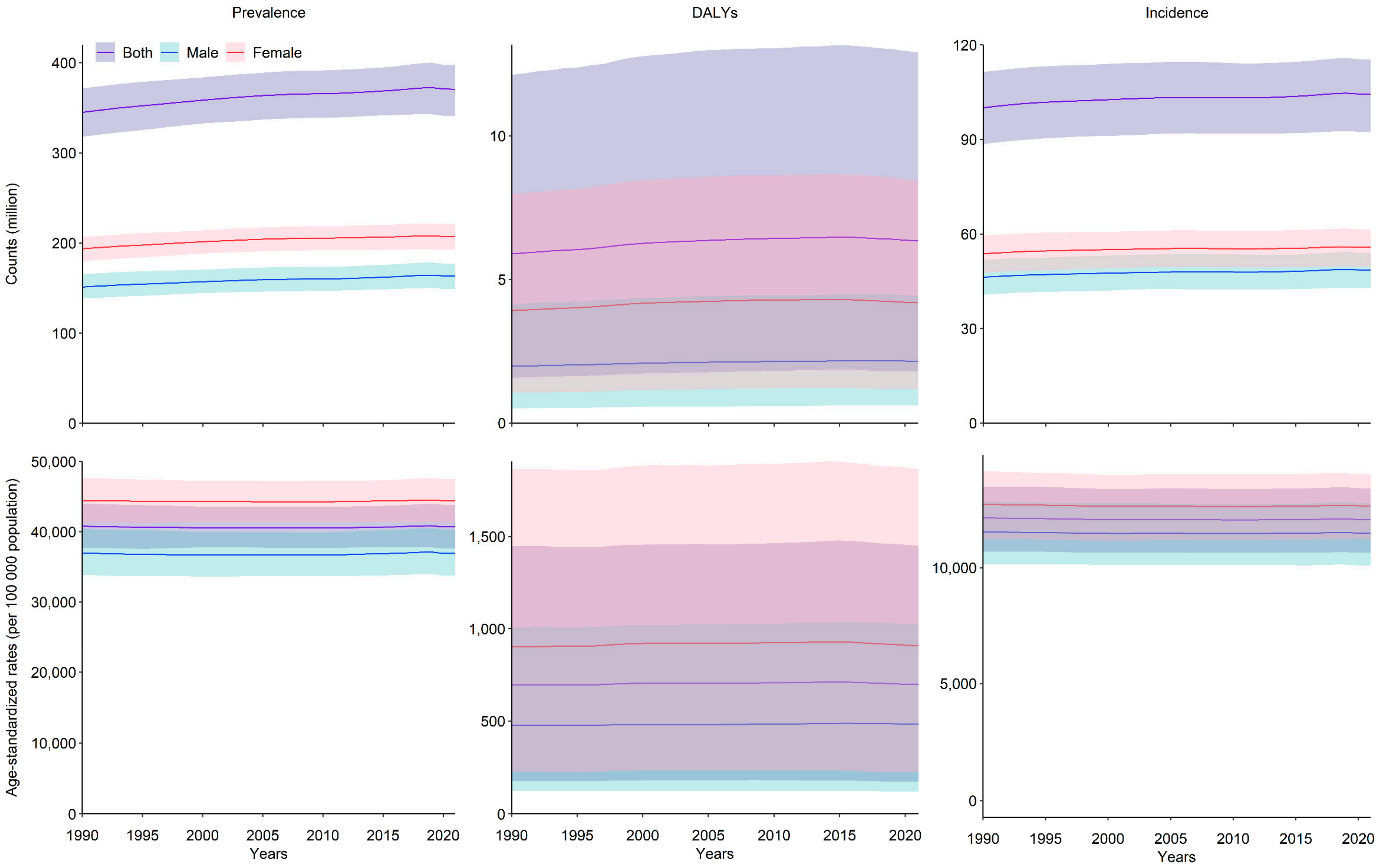

Figure S10a: Age-standardized prevalence, DALY, and incidence rates and counts of headache disorders, by sex, 1990-2021

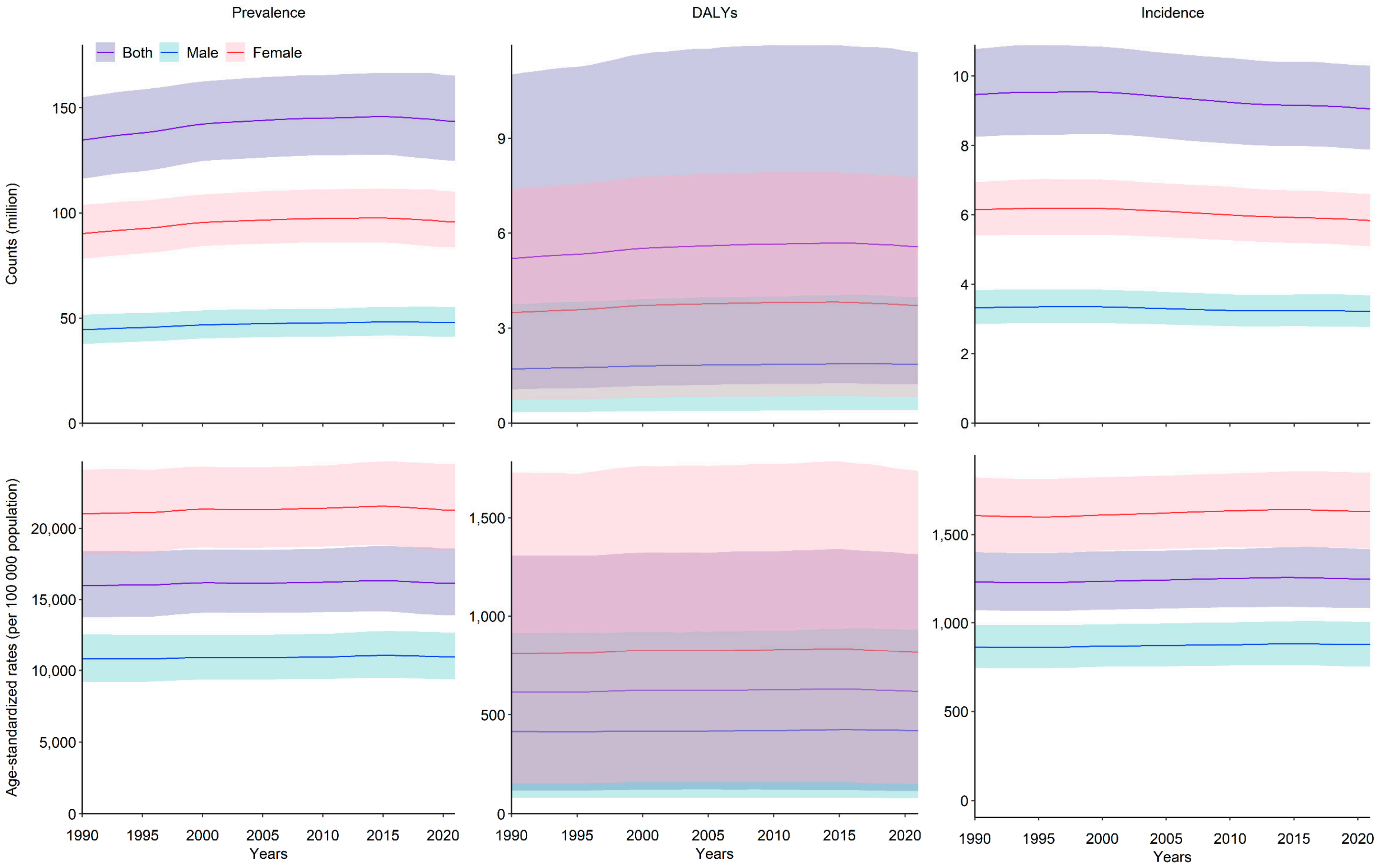

Figure S10b: Age-standardized prevalence, DALY, and incidence rates and counts of migraine, by sex, 1990-2021

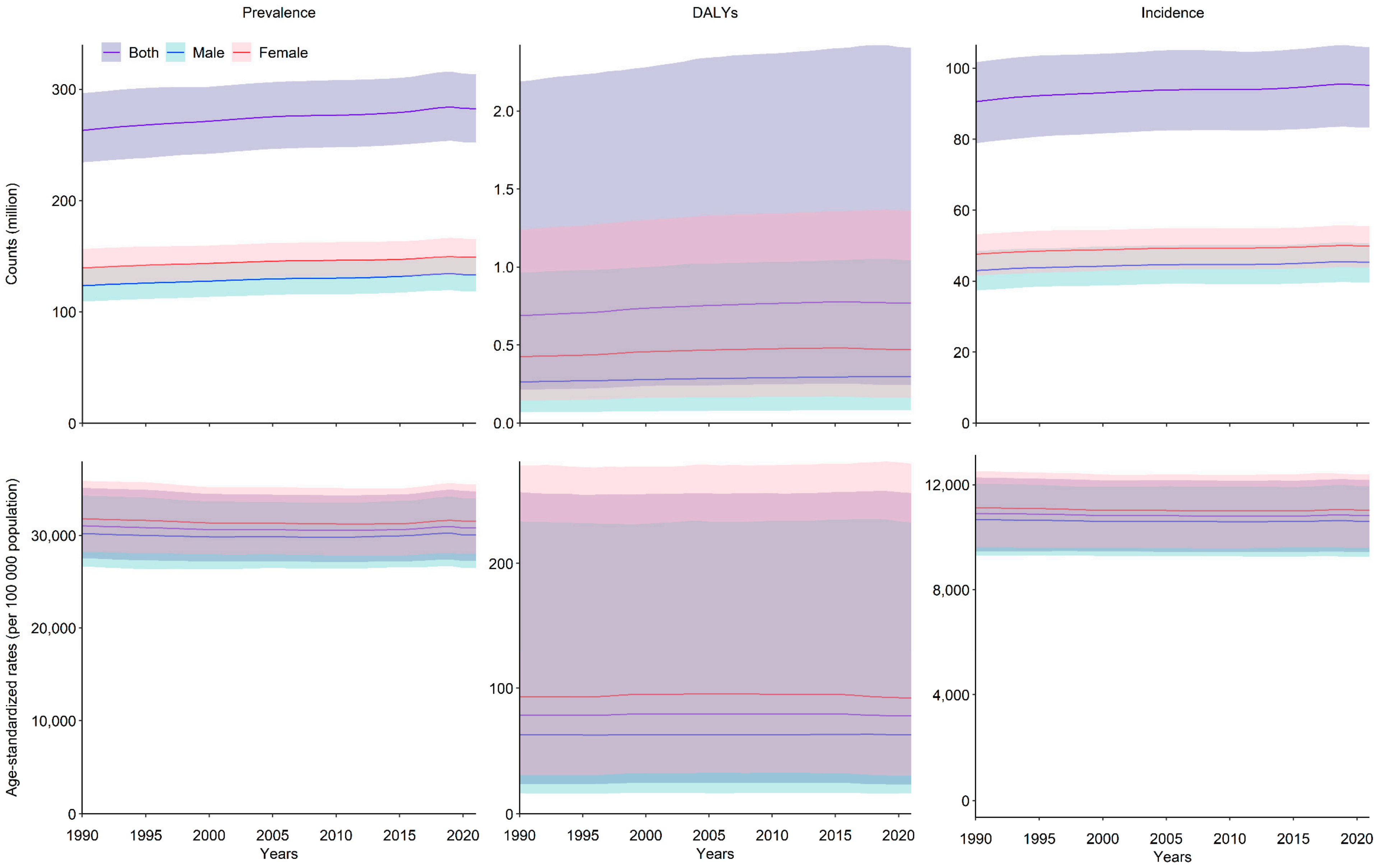

Figure S10c: Age-standardized prevalence, DALY, and incidence rates and counts of tension-type headache, by sex, 1990-2021

(A) Prevalence and DALYs

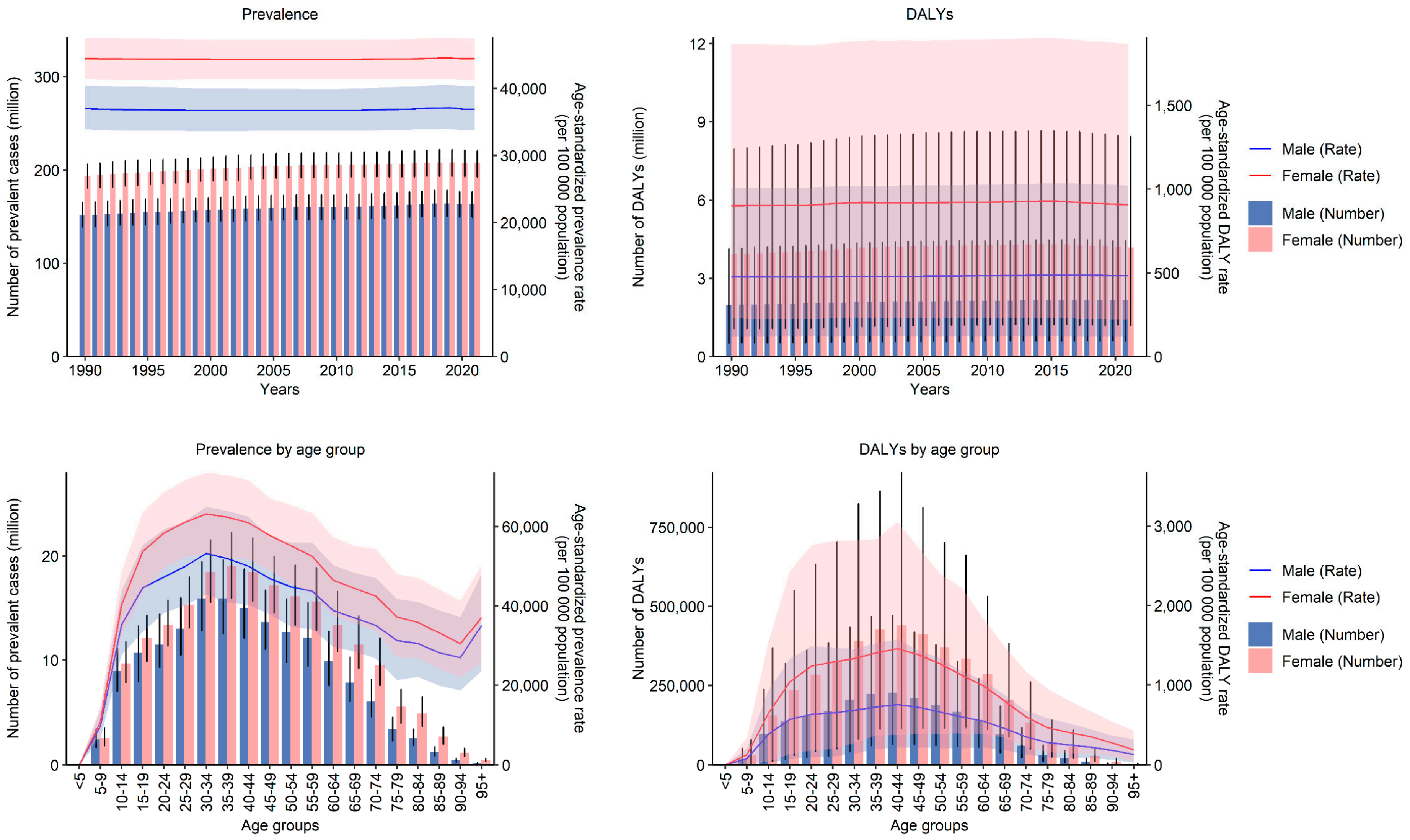

(B) Incidence and YLDs

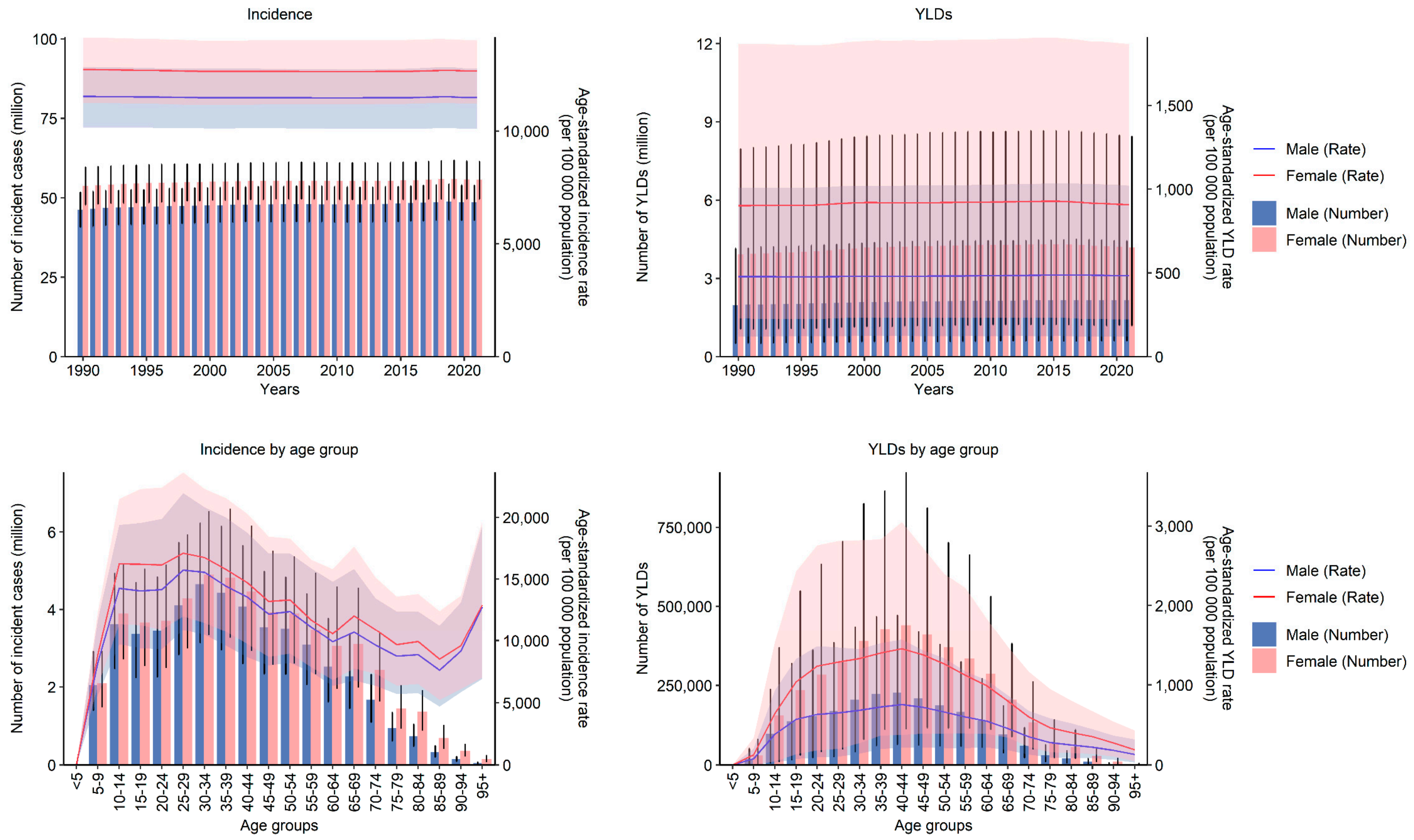

Figure S11a: Counts and rates of (A) prevalence and DALY, and (B) incidence and YLD of headache disorders, by year, age group, and sex

(A) Prevalence and DALYs

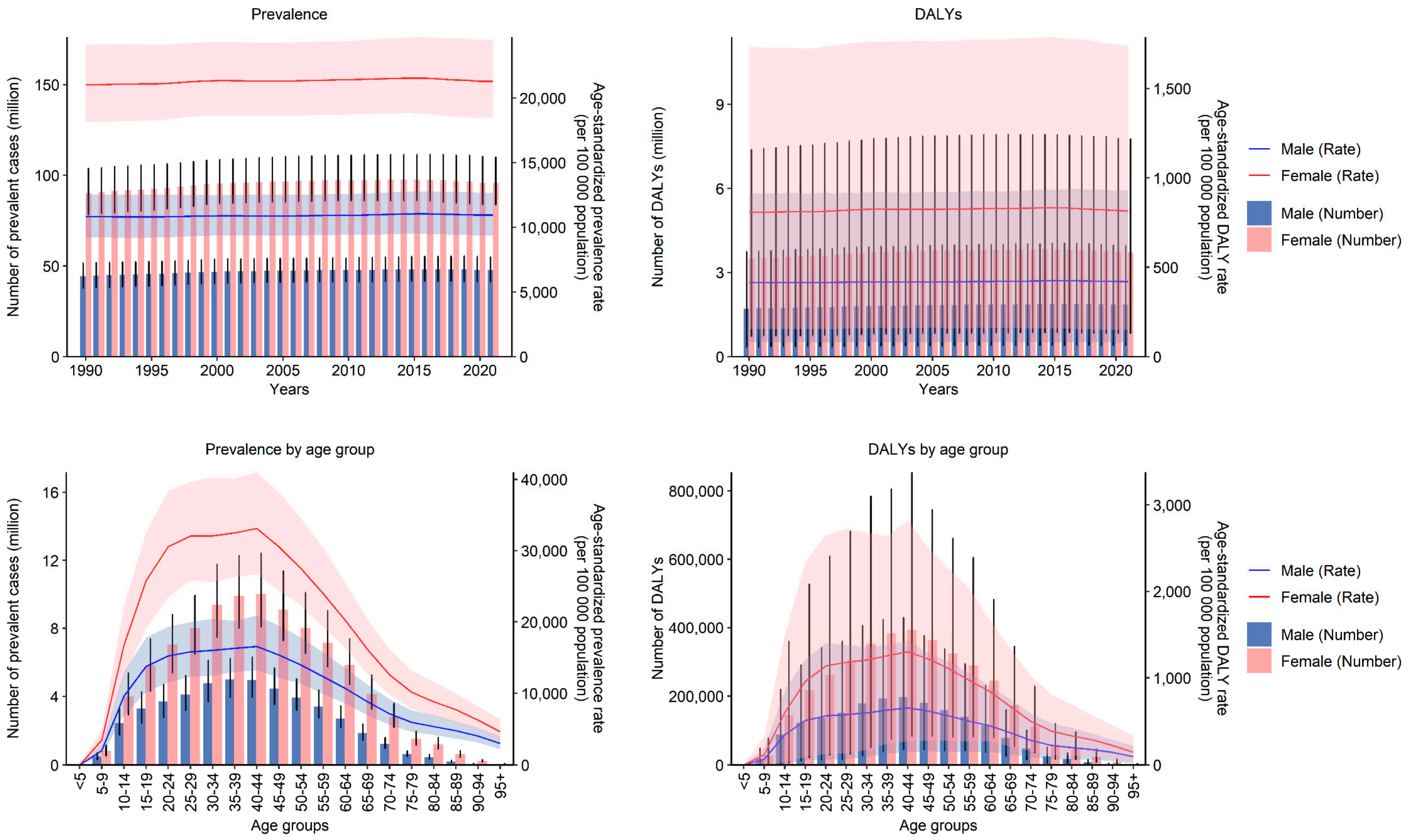

(B) Incidence and YLDs

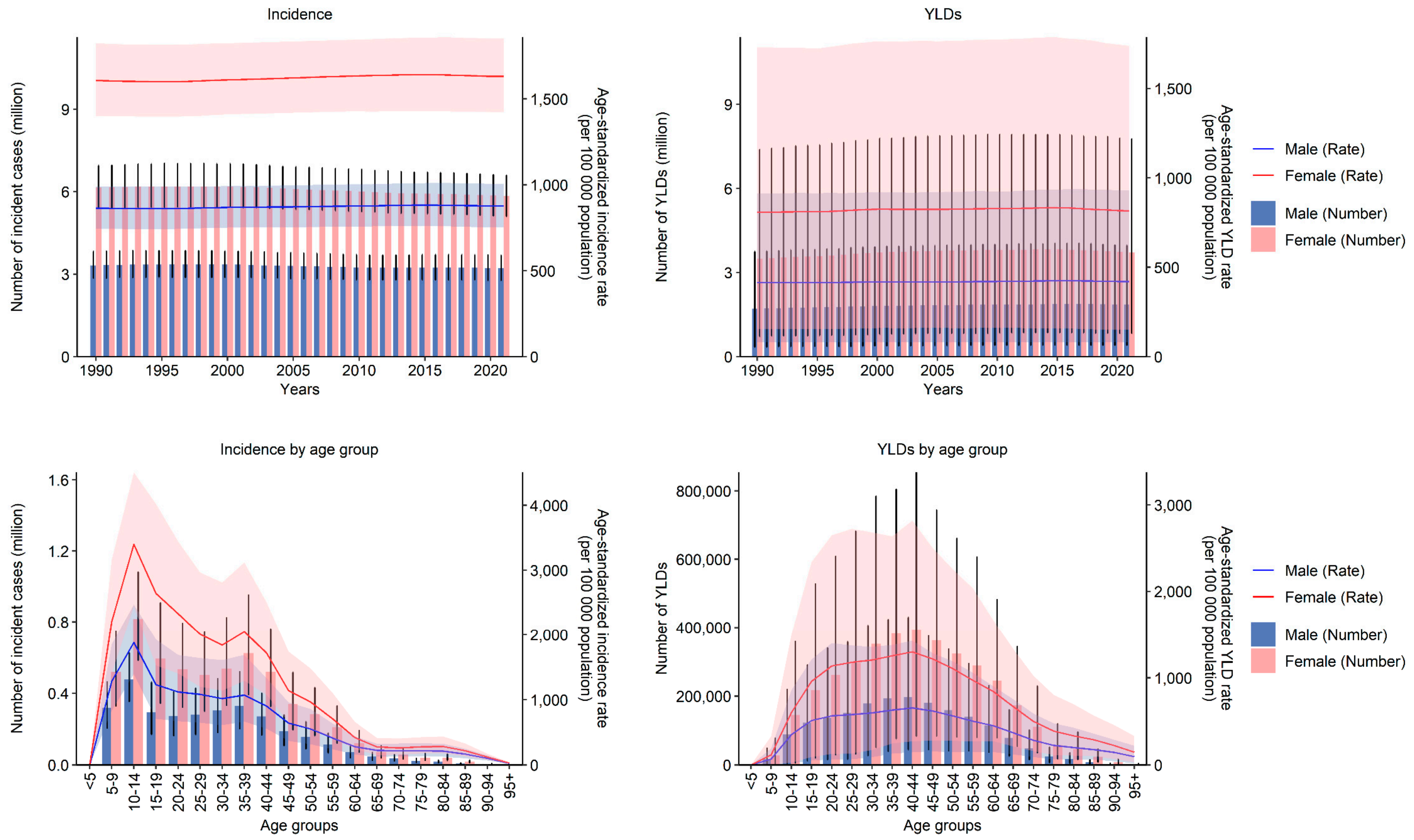

Figure S11b: Counts and rates of (A) prevalence and DALY, and (B) incidence and YLD of migraine, by year, age group, and sex

(A) Prevalence and DALYs

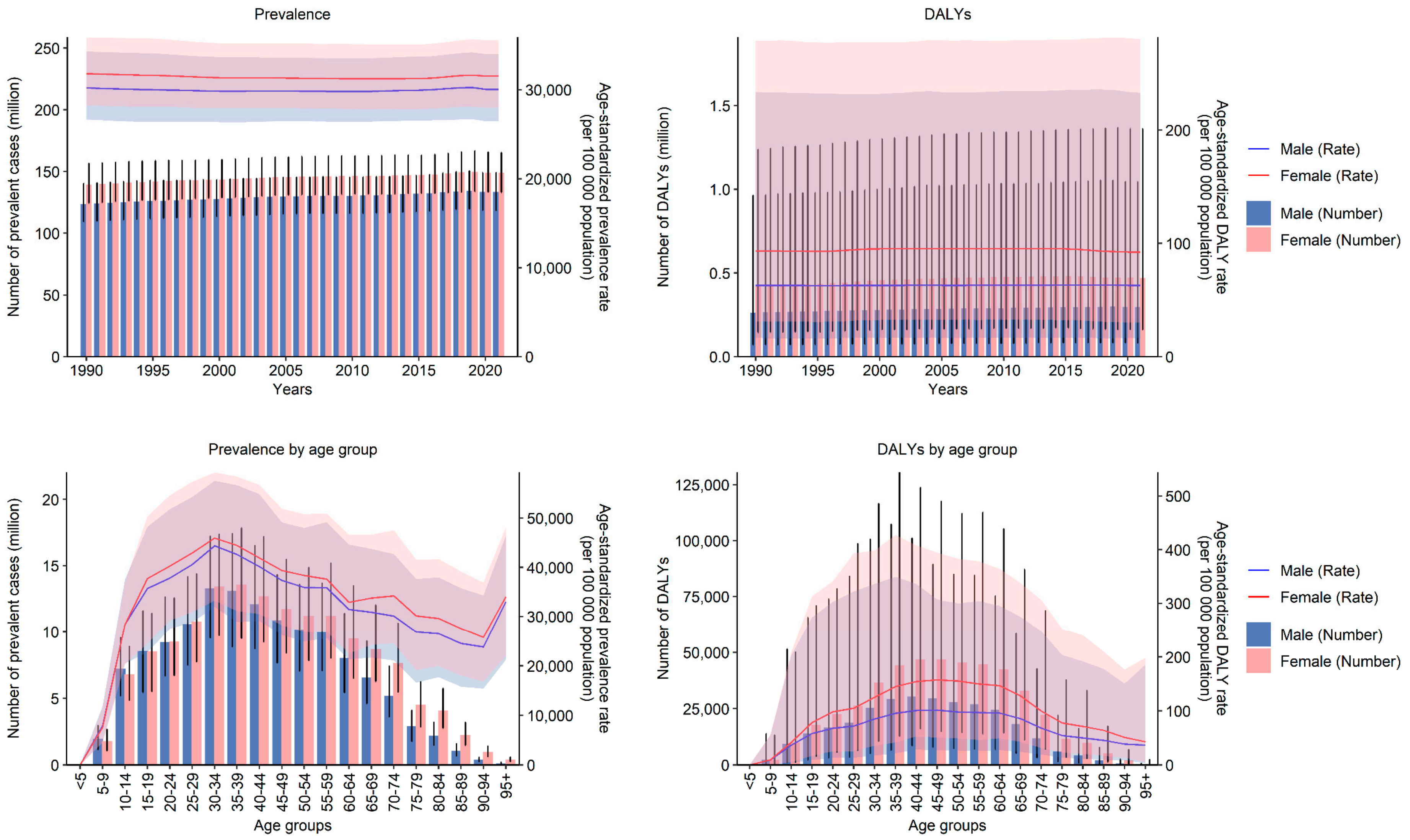

(B) Incidence and YLDs

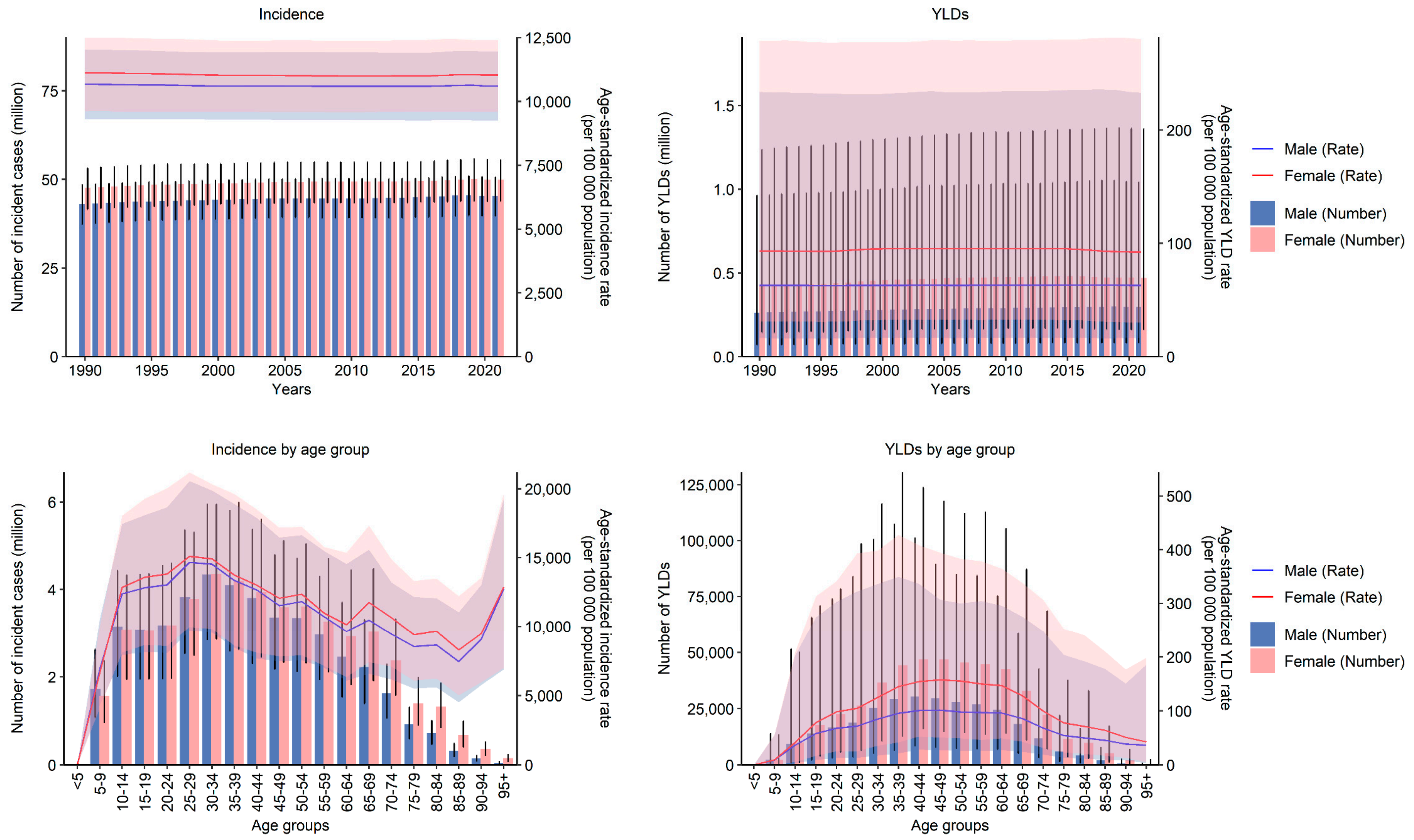

Figure S11c: Counts and rates of (A) prevalence and DALY, and (B) incidence and YLD of tension-type headache, by year, age group, and sex

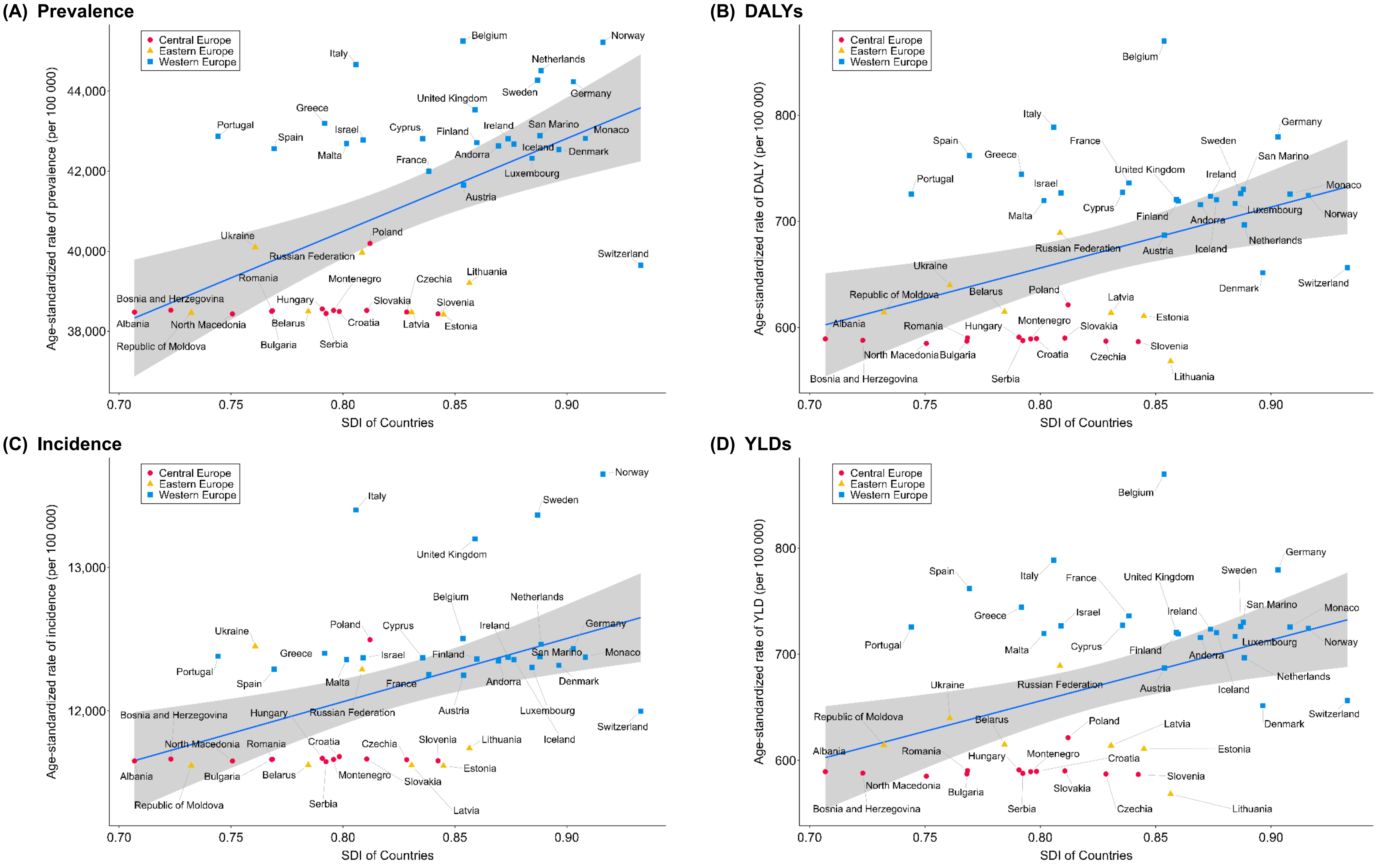

Figure S12a: Age-standardized (A) prevalence, (B) DALY, (C) incidence, and (D) YLD rates for headache disorders in Europe, by socio-demographic index (SDI), 2021

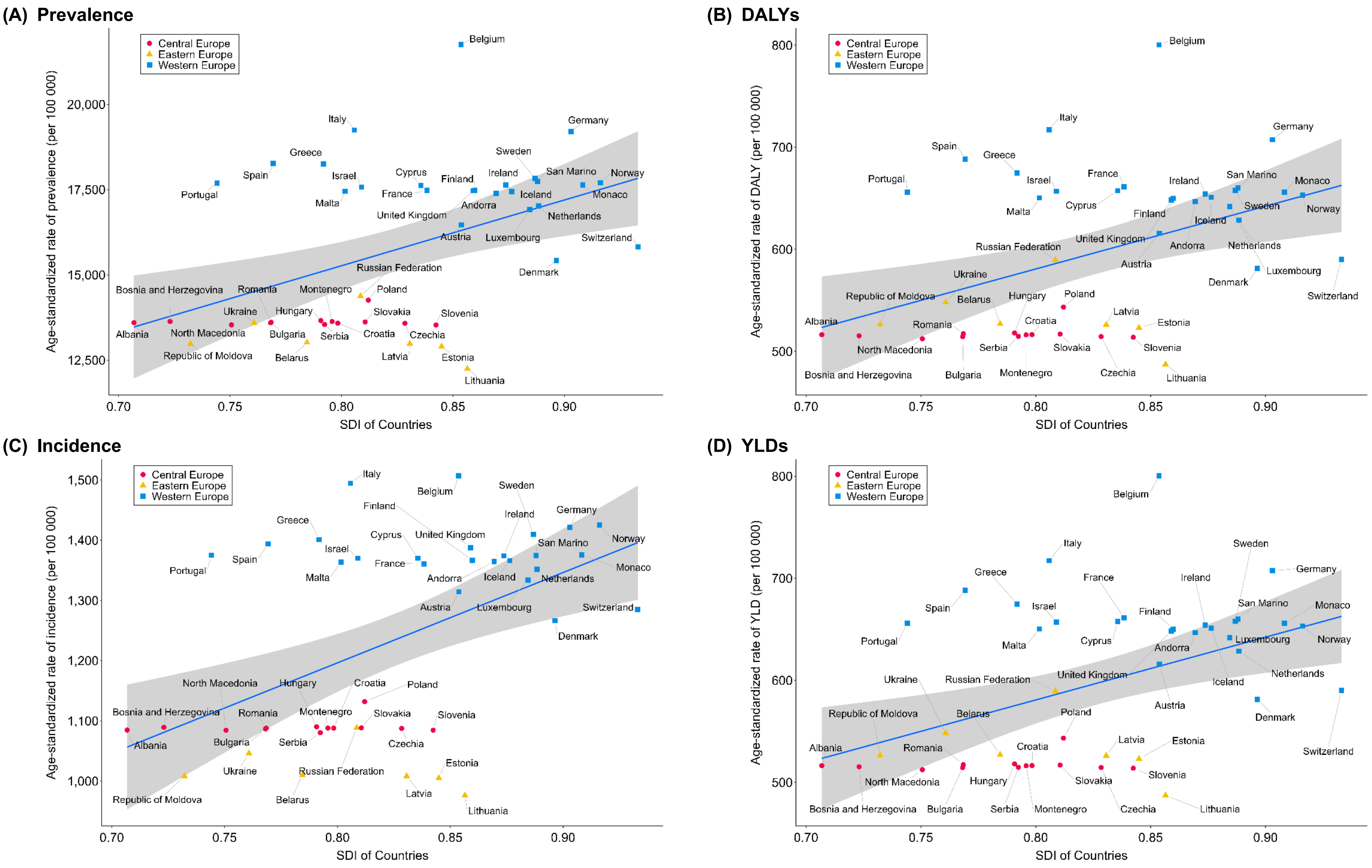

Figure S12b: Age-standardized (A) prevalence, (B) DALY, (C) incidence, and (D) YLD rates for migraine in Europe, by socio-demographic index (SDI), 2021

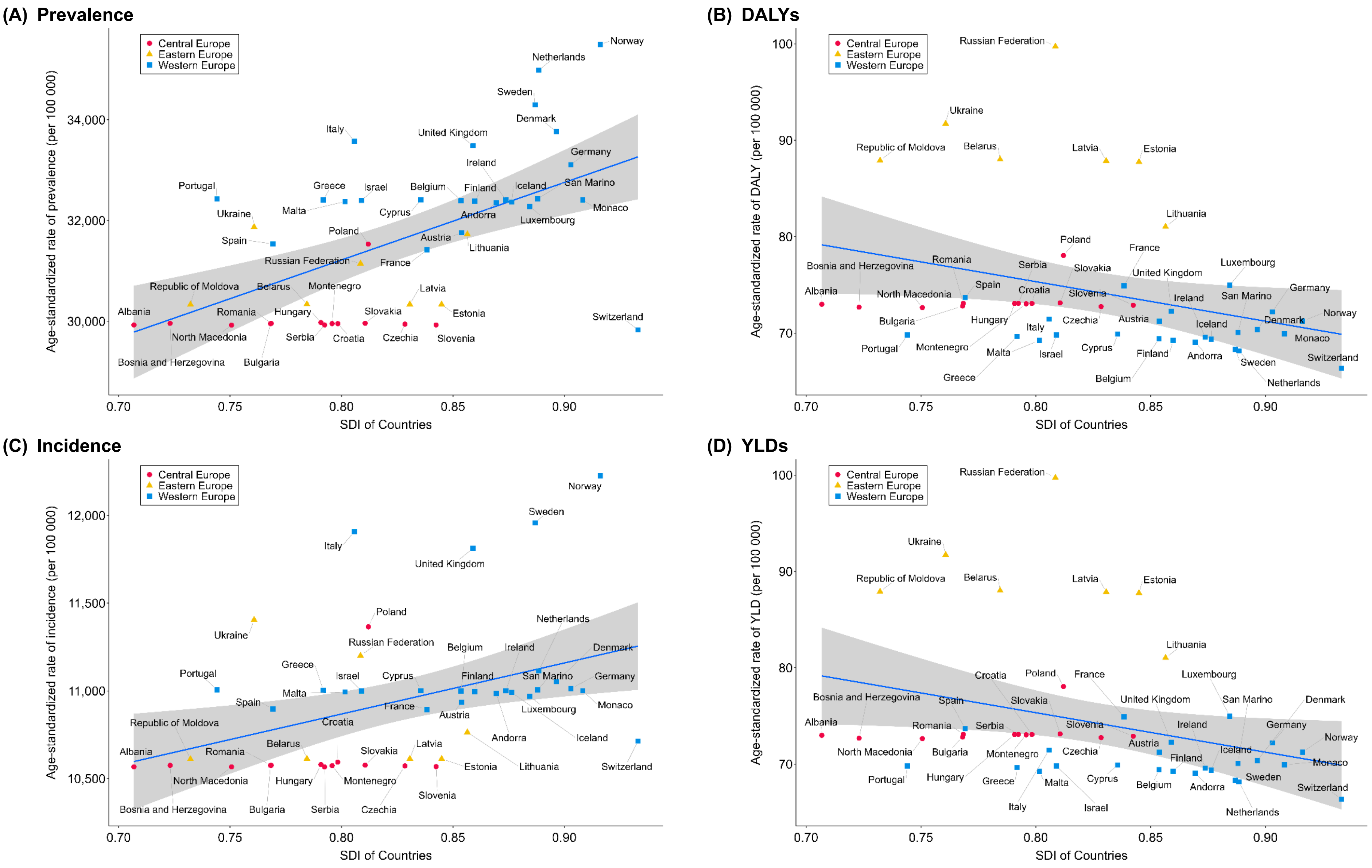

Figure S12c: Age-standardized (A) prevalence, (B) DALY, (C) incidence, and (D) YLD rates for tension-type headache in Europe, by socio-demographic index (SDI), 2021
